# The proteomics of roadside hawk (*Rupornis magnirostris*), broad-snouted caiman (*Caiman latirostris*) and loggerhead sea turtle (*Caretta caretta*) tears

**DOI:** 10.1186/s12917-020-02495-0

**Published:** 2020-08-07

**Authors:** A. C. Raposo, C. B. Lebrilla, R. W. Portela, E. Goonatilleke, F. A. Dórea Neto, A. P. Oriá

**Affiliations:** 1 School of Veterinary Medicine, Federal University of Bahia, Salvador, 40110-060 Brazil; 2 Chemistry Department, Mass Spectrometry Facilities Campus, University of California, Davis, CA 95616–8585 USA; 3 Institute of Health Sciences, Federal University of Bahia, Salvador, 40110-100 Brazil

**Keywords:** Bird, Clinical biochemistry, Ocular surface, Reptile, Tear film

## Abstract

**Background:**

Tears play an important role in ocular surface protection, and help wild animals maintain visual acuity in the face of air and water friction. The proteomics of tears has only been described for mammals. The knowledge of the proteomics of wild animal tears can aid not only in the setting of normal standards for ocular disease studies in these animals, but also to base the search for new molecules to be used in ophthalmology therapeutics. We therefore set out to describe the proteomic profile of roadside hawk (*Rupornis magnirostris*), broad-snouted caiman (*Caiman latirostris*) and loggerhead sea turtle (*Caretta caretta*) tears. Tears were collected from healthy animals, their spectral profiles were obtained with an LTQ Orbitrap XL mass spectrometer, and the dataset was analyzed against reference taxa.

**Results:**

For roadside hawk, 446 proteins were identified, the most abundant being albumin, transferrin, globulin and actin. For broad-snouted caiman and loggerhead sea turtle, 1358 and 163 proteins were identified, respectively. Uncharacterized proteins and transferrin were highly abundant in both species. The roadside hawk tear components and their properties were similar to those described for humans, but with a higher albumin concentration. Broad-snouted caiman tears presented a wide diversity of ontological functions, with an abundant presence of enzymatic compounds. In loggerhead sea turtle tears, the predominance of proteins with ion-transport functions was consistent with possible osmolality-maintenance mechanisms.

**Conclusion:**

These data enhance our understanding of birds and reptiles’ tears microcomposition and may be used to base the discovery of new molecules with high biotechnological potential.

## Background

Biotechnological advances have contributed to the description of microcomponents present in fluids, their properties, and the presence of health and disease biomarkers [1–3]. Among these technologies, proteomics has proven to be a powerful method for obtaining information on polypeptides, via comparison of the expressed profile to post-translational modifications [4–6].

Numerous organic components can provide information on biological pathways and activities, or even clinical applications [6, 7]. Among the fluids, tears are of great interest due to easy and noninvasive sampling, a composition that is less complex than that of blood serum, and an immediate response to environmental challenges and changes in humans and others animals [3, 7–9]. The tear fluid contains electrolytes, proteins, lipids, mucins, metabolites and small molecules [10]. Among its functions, the most important are lubrication, protection, nutrition of the ocular surface, and modulation of the eye’s optical properties [11, 12].

Non-human mammals’ tear studies have been conducted for the establishment of experimental or clinically relevant models [13–15]. However, there is no information on the proteomic components and dynamics of tears in non-mammalian species. Information on other classes is often extrapolated and may not be valid, mainly because tears are the body fluid that is most exposed to the environment,[9] and conditions such as friction with air and water may cause differences in tear composition.

Vision is the main sense in birds and is essential for many basic habits, such as flying, mate selection, predator avoidance and prey [16–18]. With the objective to promote an ideal light refraction and a satisfactory visual acuity, the tear and cornea must be healthy [11] Reptiles are found in varied ecological niches, with varied lifestyles. Consequently, their organic fluids and metabolism present differences that reflect their adaptation to these environments [19, 20]. Thus, to elucidate the composition of non-mammalian species’ tear fluids, we determined the proteomic profiles of tear fluid from roadside hawk (*Rupornis magnirostris*), broad-snouted caiman (*Caiman latirostris*) and loggerhead sea turtle (*Caretta caretta*). The composition of these wild animals’ tears can provide information on modifications due to the environment or the evolutionary process, serve as a base for studies on biomarkers of ophthalmic diseases, and reveal molecules of potential biotechnological importance.

## Results

**Fig. 1:**
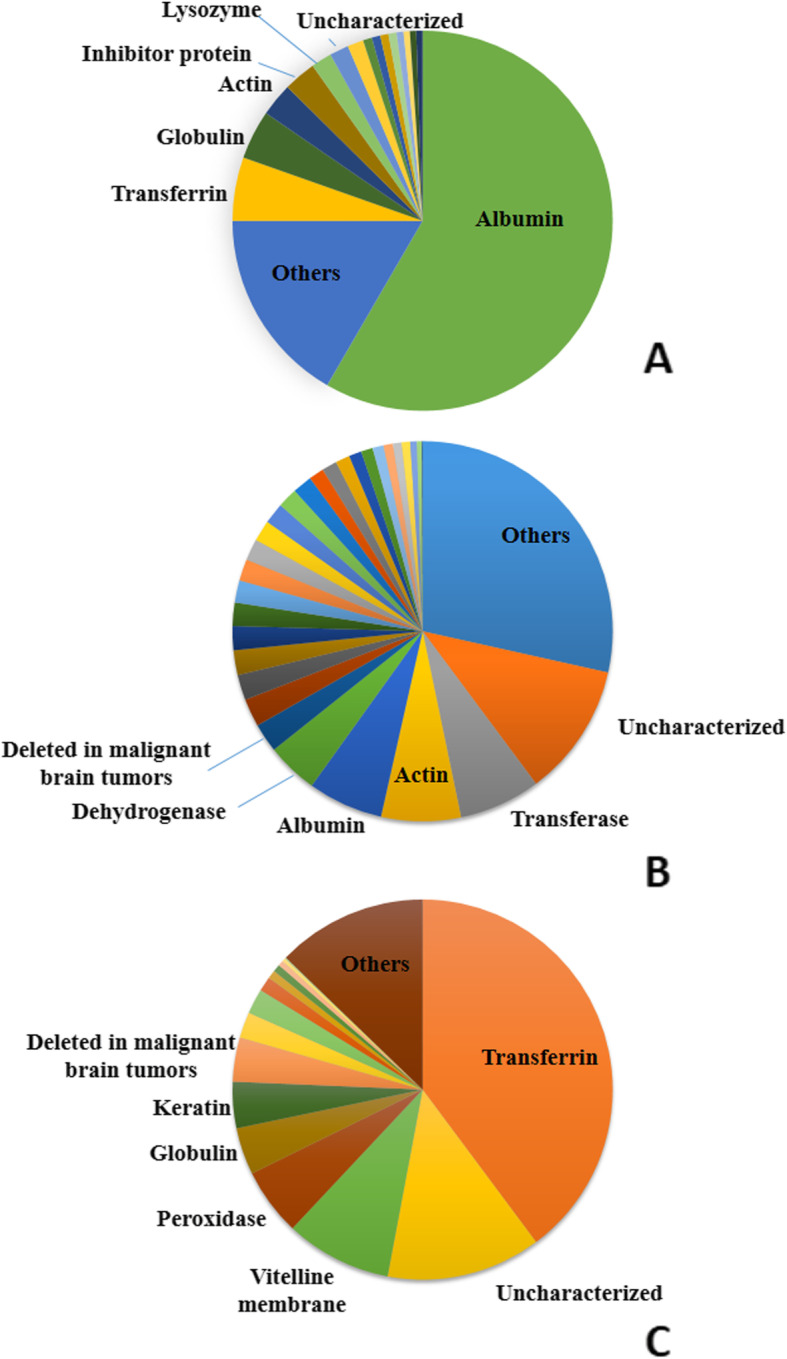
Distribution of abundance of proteins identified in roadside hawk **(a)**, broad-snouted caiman **(b)** and loggerhead sea turtle **(c)** tears. Different shades of the same color do not correspond to proximity or similarity among proteins

We identified 446 proteins (Additional file 1) in the roadside hawk tear fluid according to the UniProtKB database; among the most abundant were albumin (53.4%), transferrin (4.9%), globulins (3.8%), actin (2.6%), inhibitory proteins (2.5%), lysozymes (1.5%), apolipoproteins (1.2%), transferases (0.7%), dehydrogenases (0.7%), proteins of the complement system (6%), kinases (0.63%), reductases (0.5%), aldolases (0.5%) and isomerases (0.5%). Other proteins occurred at a lower frequency, together making up about 17% of the total protein abundance (Fig. 1a).

The spectral profile obtained with the LTQ Orbitrap XL enabled the identification of 1358 broad-snouted caiman tear proteins (Additional file 2). The most abundant proteins were: uncharacterized proteins (11.3%), transferases (6.9%), actin (6.8%), albumin (6.4%), dehydrogenases (4.4%), deleted in malignant brain tumors (2.5%), kinase (2.4%), myosin (2.2%), aldolases (2.1%), enolases (2.0%) and peroxidases (2.0%). Other proteins, such as olfactomedin, heat-shock proteins, isomerases, RNA ligase, inhibitors, proteases, transferrins, globulins, ribosomal proteins, annexin, tubulins, complement system proteins, synthases, reductases, phosphorylases, GTPases, keratin and mucins were present at less than 2%. The group “others” contained proteins present at less than 0.06% (Fig. 1b).

We found 163 proteins in the tears of loggerhead sea turtles (Additional file 3). Transferrin (39.5%) was the most abundant protein, followed by uncharacterized proteins (13.0%), vitelline membrane proteins (9.0%), peroxidase (5.7%), globulin (4.0%), keratin (3.9%), tumor markers (3.7%), actin (2.2%) and transferase (2.1%). Albumin, dehydrogenases, kinases, histones, RNA ligase and mucins were observed at frequencies lower than 2%. Proteins with frequency less than 0.01% were included in the “others” group (Fig. 1c).

Serum albumin and ovotransferrin were found in all animal tears with high abundance, and it were able to detect immunoglobulins in the tears of roadside hawk and loggerhead sea turtles. However, there were some differences among the ten proteins with the highest abundance in the tears of the animals studied herein (Table 1).

**Table 1:** Proteins with the highest abundance in roadside hawk, broad-snouted caiman, loggerhead sea turtle and human tears

Rank	Roadside hawk	Broad-snouted caiman	Loggerhead sea turtle	Human^3^
1	Serum albumin	Serum albumin isoform	Ovotransferrin	Human lactoferrin
2	Ovotransferrin	Beta-actin	Uncharacterized protein	Serum albumin
3	Ig lambda-1 chain	Uncharacterized protein	Vitelline membrane outer layer protein 1	Complement C3
4	Ig heavy chain V	Glutathione S-transferase	Myeloperoxidase	Heparan sulfate proteoglycan
5	Actin, cytoplasmic 2	Deleted in malignant brain tumors 1 protein-like	Deleted in malignant brain tumors 1 protein	Myosin
6	Ovoinhibitor	Protein-glutamine gamma-glutamyltransferase	Ig mu chain C region	Zinc-alpha-2-glycoprotein
7	Ig lambda chain V-1	Alpha-enolase	Actin, cytoplasmic 2	Lipocalin-1
8	Nesprin-1	Olfactomedin-4	Protein-glutamine gamma-glutamyltransferase	Keratin, type I cytoskeletal
9	Lysozyme g	Ovotransferrin	Serum albumin	Isoform alpha-enolase of Alpha-enolase
10	Alpha-enolase	Lysine--tRNA ligase	Alpha-enolase	Keratin, type II cytoskeletal

**Fig. 2:**
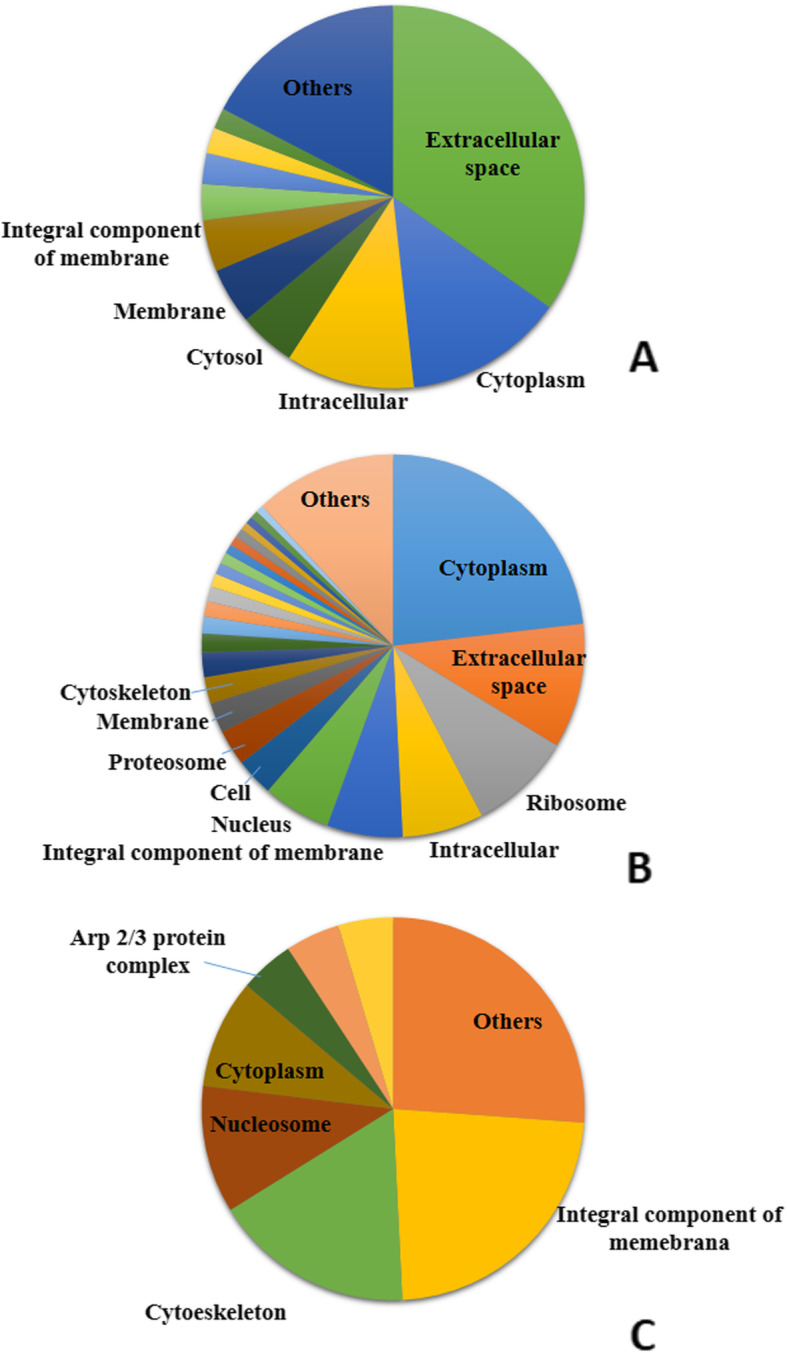
Frequency distribution of the cellular components identified in roadside hawk **(a)**, broad-snouted caiman **(b)** and loggerhead sea turtle **(c)** tears. Different shades of the same color do not correspond to proximity or similarity among proteins. The presented data are not necessarily correlated to proteins of greater abundance

Approximately 230 cellular components were identified in the roadside hawk tears. The identified proteins were most frequently associated with the extracellular region (albumin, transferrin and globulin – 34.8%), cytoplasm (13.5%), cell fraction (10.9%), cytosol (4.8%), integral membrane components (4.3%), cytoskeleton (actin – 3.0%); other associations, with lower frequency, were with the endoplasmic reticulum, hemoglobin complex, and nucleus (Fig. 2a). Overall, 626 and 65 cellular components were observed in the tears of broad-snouted caimans and loggerhead sea turtles, respectively. Broad-snouted caiman proteins were most frequently associated with the cytoplasm, extracellular space, ribosomes, intracellular space, membrane components and nucleus, making up about 55.6% of this ontology classification (Fig. 2b). Some broad-snouted caiman tear proteins were classified as intracellular content. Loggerhead sea turtle tear proteins were characterized as cellular components associated with integral membrane components (23%), cytoskeleton (16.9%), nucleosomes (10.8%), cytoplasm (9.2%), Arp 2/3 protein complex (4.6%), intracellular region (4.6%) and nucleus (4.6%) (Fig. 2c).

**Fig. 3:**
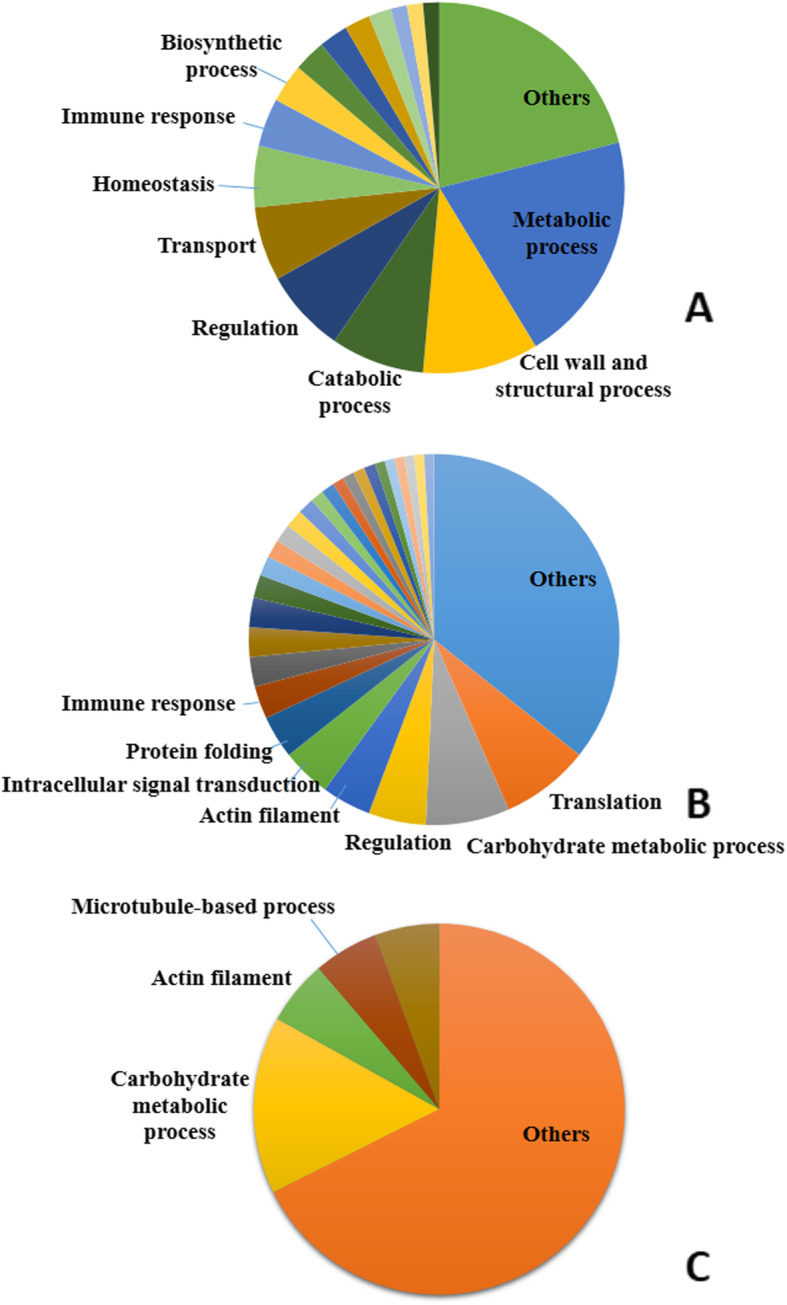
Frequency distribution of the biological process components identified in roadside hawk **(a)**, broad-snouted caiman **(b)** and loggerhead sea turtle **(c)** tears. Different shades of the same color do not correspond to proximity or similarity among proteins. The data presented are not necessarily correlated to the proteins of greater abundance

The most frequent biological processes of the roadside hawk tear proteins were metabolic processes (20.2%), cell wall component and structural process (10.1%), catabolic processes (8.1%), regulation (7.3%), transport (6.5%), homeostasis regulation (5.3%), immune response (4.2%) and, less frequently, biosynthesis, redox processes, microtubule-based processes, protein folding, cellular response, oxidative stress and glucogenesis (Fig. 3a). In broad-snouted caimans, proteins were classified into the following biological processes: translation (7.7%), carbohydrate metabolic process (7.3%), regulation (5.0%), actin filament (4.3%), intracellular signal transduction (4.3%), protein folding (3.7%), defense response (2.9%), cell redox (2.6%), microtubule-based process (2.6%) and ubiquitin-dependent protein catabolic process (2%); the other biological processes had a frequency of less than 2% (Fig. 3b). Only four biological processes had percentages above 5.5% in the loggerhead sea turtle tear: carbohydrate metabolic process (15.5%), actin filament (6.3%), microtubule-based (6.3%) and nucleosome assembly (6.3%) (Fig. 3c).

**Fig. 4:**
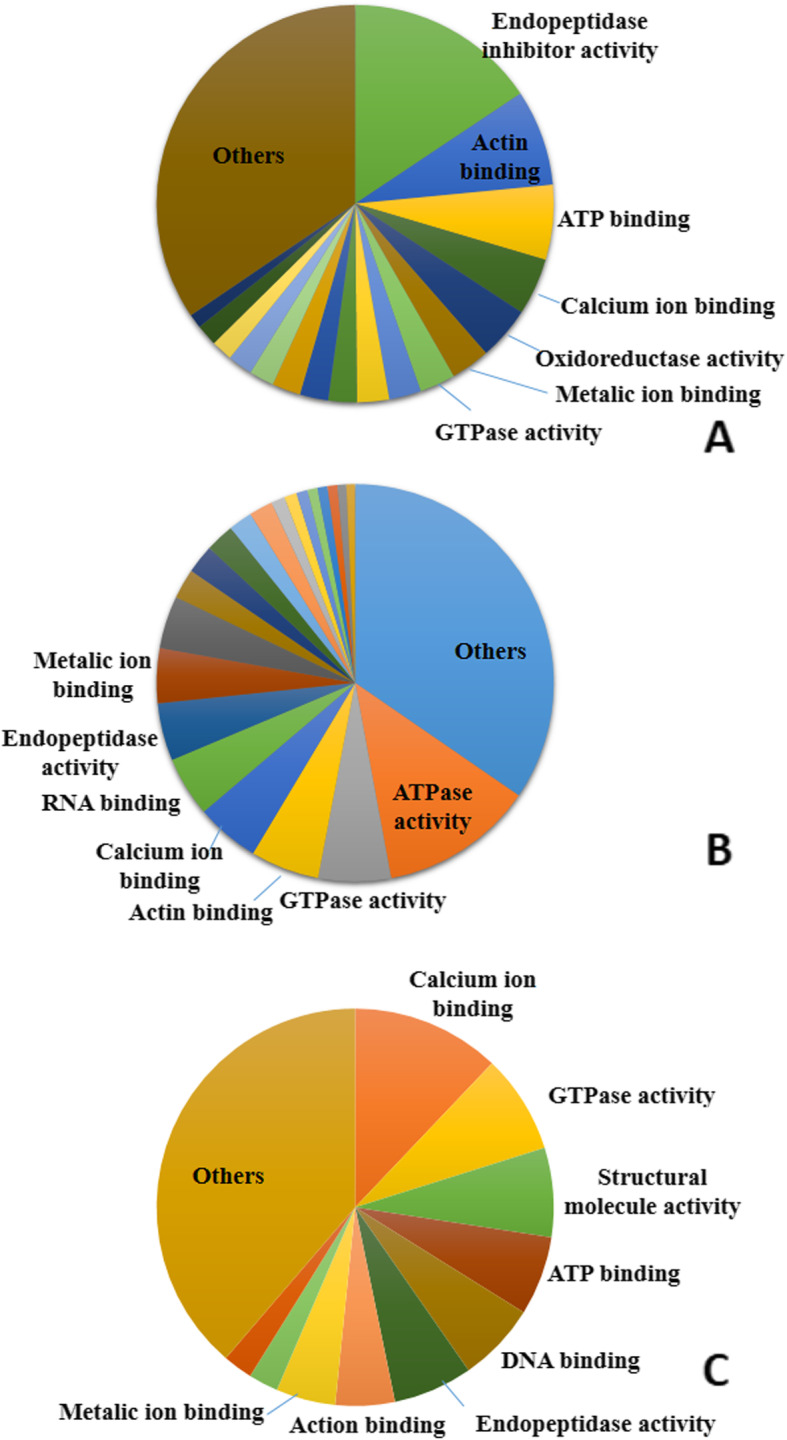
Frequency distribution of the molecular functions identified in roadside hawk **(a)**, broad-snouted caiman **(b)** and loggerhead sea turtle **(c)** tears. Different shades of the same color do not correspond to proximity or similarity among proteins. The presented data are not necessarily correlated to the proteins of greater abundance

The molecular functions of the roadside hawk tear proteins were classified as endopeptidase inhibitor activity (15.6%) associated with the amino acids serine, cysteine ​​and threonine. Other functions were detected at lower frequency, such as actin binding (7.8%), ATP binding (6.1%), calcium-ion binding (4.6%), oxidoreductase activity (4.3%), and metal-ion binding (3.2%) (Fig. 4a). The broad-snouted caiman tears showed a wide diversity of molecular functions (~ 983), with prevalent enzymatic functions: ATPase activity (12.5%), GTPase activity (5.9%), actin binding (5.6%), calcium-ion binding (5.2%), RNA binding (4.9%), endopeptidase activity (4.7%), metal-ion binding (4.5%), and structural constituent of the ribosome (4.3%). Other functions, such as oxidoreductase activity, DNA binding, translation, initiation factor activity and hydrolase activity were detected at up to 2% of the total molecular functions (Fig. 4b). Calcium-ion binding was the most frequent molecular function for the loggerhead sea turtle tear proteins (12.1%), followed by GTPase activity (8.1%), structural molecule activity (7.2%), ATP binding (6.4%), DNA binding (6.4%) and endopeptidase activity (6.4%). The other functions, occurring at frequencies lower than 5%, were actin binding, metal-ion binding, peroxidase activity and protein domain-specific binding (Fig. 4c).

## Discussion

The use of MS to evaluate tear fluid enables the description of a larger number of proteins and promotes studies on the identification of biomarkers, especially those indicating different physiological conditions or environmental influences [2, 4, 7, 21]. To expand this knowledge to nonmammalian species, the present study describes tear protein profiles that can reflect differences in environmental adaptation or different interactions with the ocular surface [6, 9, 22]. Our results provide the first description of the protein components of these wild animals’ tears.

Birds of prey and caimans generally produce less tear fluid than humans or dogs [23–25]. As this fluid can only be collected in small quantities, the collection method must be precise. Despite these difficulties, use of the LTQ Orbitrap XL enabled the identification of 446 proteins in roadside hawk tears, 1358 proteins in broad-snouted caiman tears and 163 proteins in loggerhead sea turtle tears. Studies of human tears using a similar methodology have reported the presence of 491 and 1526 proteins,[26, 27] with the difference being attributed to the methodology of tear collection,[27] i.e., possible retention of polypeptides on the Schirmer strip [28]. However, in this study, the difference in collection methods and consequent tear extraction did not seem to be the key to explaining the large differences in detected proteins, since the strip was used in roadside hawks and broad-snouted caimans, and the latter presented a greater abundance of proteins.

Research into animals that fly prompts the question of what mechanisms are used to keep their tear fluid stable, so that it will not dissipate under friction of the air with the ocular surface [9]. Similarly, the projections of the corneal epithelium in freshwater and marine animals can be maintained and nourished by tears [9, 29]. Indeed, this interface with the environment followed the adaptation process with the objective to maintain homeostasis, osmotic balance and metabolite transport,[9, 11] as already showed by previous studies on the tear composition of animals from the same Classes that live in different environments [22, 30].

A comparative study on human tear elements showed that a polluted air (extrinsic factor) and the age (intrinsic factor) can change the tear composition [31, 32]. For animals, investigations like these are not available until now; however, it is reported that free-living and domiciliated turtles present differences in the composition of the eye microbiota, and there are differences in the hematological parameters of turtles with different ages,[33, 34] and these situations can possibly influence the microcomponent concentrations in tears.

Although the application of MS in this research provided some important results, the absence of a database including the specific species, or taxonomically close species’ genomes could present an obstacle to result interpretation. Comparing our MS data for roadside hawk tears to prior descriptions of Aves taxa, for broad-snouted caiman tears to prior descriptions of Alligatoridae, and for loggerhead sea turtle tears to prior descriptions of Testudines, may reduce the accuracy of some findings, and may have influenced the information obtained for ontology categories, but these were the only data available for consideration.

Zhou and Beuerman [2] classified the polypeptides found in human lacrimal fluid according to their abundance; they found that proteins associated with the innate immune response make up the largest group, defining the main function of tears in humans. Our protein profile of roadside hawk tears differed in terms of abundance compared to mammalian tears, but in general, the same proteins were identified in both. Compared to primates,[3, 22] a higher abundance of albumin was observed in the roadside hawk tear fluid, which may reflect its importance in the maintenance of osmolarity, thereby reducing possible damage caused by flight.

Comparing the protein profiles of the two reptile species’ tears in this study, we observed a greater abundance of proteins in the broad-snouted caiman and a higher frequency of transferrin in the loggerhead sea turtle. Therefore, transferrin may be key to the mechanism of ionic balance in the corneal epithelium of loggerhead sea turtles, since the high salinity in their environment can result in osmotic stress at the ocular surface [29]. Similarly, the remarkable presence of uncharacterized proteins in both reptiles indicates significant gaps in our understanding of their tears’ main functions, especially for broad-snouted caimans. The other proteins, except for the vitelline membrane proteins of the loggerhead sea turtle tear, have also been found in human tears, albeit in different amounts [2].

Transferrin and other proteins associated with ion transport are described as an immunological barrier in tears. Together with the abundance of globulin, these proteins might serve as a defense mechanism for ocular surface protection in birds and sea turtles, as has been previously shown in mammals [3, 15, 26, 35]. In the broad-snouted caiman tear profile, proteins with immunological function were not identified in the clusters of greater abundance; in contrast, in the loggerhead sea turtle, 4% of the tear was composed of globulins. However, the high presence of proteins with enzymatic action in broad-snouted caiman tears suggests that, because this animal has a more primitive immune system—as verified by the absence of IgA in broad-snouted caiman tears,[36] these enzymatic compounds may play a crucial role in the protection of these reptiles’ eyes against microbes.

Actin has also been observed in the tears of other species. It is a major component of the ocular surface epithelium microprojections, and an important component of the cytoskeleton [3, 9, 15, 26]. Together with actin, the proteins found in smaller abundance might be foreign proteins, attributable to rupture of the adjacent tissue during sample collection. The problem of tissue lesions was strongly considered at the time of sampling, but even microlesions can lead to the detection of these proteins because of the high sensitivity of the methods used [2]. As the exfoliative process of the corneal epithelium is constant in any species, and the ocular surface epithelium has been shown to undergo total renovation in a period of 7 days,[37] these cellular proteins can be considered normal components of the tear fluid.

The freshwater broad-snouted caiman’s tear proteins were most frequently associated with the cytoplasm, extracellular space, ribosomes, intracellular space, membrane components and nucleus, making up about 55.6% of this ontology classification. Taking into consideration the particularities of this species, the high protein presence may promote a stable tear film, despite wide intervals between palpebral incursions [25] and the caiman’s unhealthy environmental niche, i.e., muddy swamps (which can cause intense and constant friction at the ocular surface) or even polluted waters. Therefore, the cell-renewal rate of this species is expected to be high, as a compensatory mechanism for these conditions. On the other hand, loggerhead sea turtles presented a predominance of proteins associated with membranes and cytoskeleton (about 44.6%), which may be associated with the high density of corneal epithelial cell microprojections reported in these animals [9, 29].

The concentrations of microcomponents in organic fluids, including tears, are correlated to homeostatic and metabolic processes. As an example, studies in humans showed differences in the tears of healthy patients and those with systemic diseases, such as kidney disease and diabetes [38, 39]. However, in others species, information on the permeability of the lacrimal gland/plasma interface are not available and direct correlations between blood and tear protein composition are scarce [30].

Albumin is an important serum component among vertebrates, presenting a role in ion transport and osmolarity maintenance [22, 40]. It is noteworthy that this component is among the most abundant ones in the tears of the three species studied herein and humans. Ovotransferrin, belonging to the transferrin protein family, was abundantly found in the tears of the three species and humans. These proteins are an important component of body fluids, and their presence in different tissues of birds and reptiles shows its importance in ion transport and regulation, as well as in the immune response [41]. Immunoglobulins have particularities between Avian and Reptile Classes, with species-specific differences, such as the possible absence of IgA in some reptiles; indeed, these proteins are present in tears and have an immunological role [42].

Some proteins that were identified in the tears studied herein were previoulsy detected only in specific tissues, such as vitelline, described in birds and reptile eggs and with a possible antimicrobial function,[43] and olfactomedin-4, described as a biomarker of intestinal inflammatory processes in humans [44] and in the venom of snakes [45]. The presence of these proteins in the tear suggests that further studies should be carried out to understand the tear composition of these animals, or even if these proteins present a high similarity with other proteins that were already described, but with specific differences in its composition.

In proteomics studies of humans and dogs tears, the ontology classification of biological processes acts as a guide for tear function, with an emphasis on processes correlated to the immune system [2, 15, 26]. In roadside hawk, metabolic processes and transport of substances associated with albumin were among the most frequent biological processes, which might be a result of the previously described characteristic of homeostasis maintenance during flight [9]. Carbohydrate metabolic process and actin filament were two important processes in reptile tears, and have also been described in humans, but in smaller percentages [2, 26].

In relation to the molecular functions of the proteins, Winiarczyk et al. [15] identified activities related to hydrolases, lysozymes and immunological response as the main molecular functions in dog tears, similar to that found in human tears [3]. The main molecular function cluster found in birds was endopeptidase inhibitor activity which, according to Souza et al.,[26] can be used as a biomarker, since protease imbalances can be correlated to disease. According to the same authors,[26] this mechanism can also be attributed to the tears’ role in immune defense.

Calcium-ion binding was the most frequent molecular function in loggerhead sea turtle tears. The strong presence of functions correlated to ion transport associated with transferrin frequency reinforces the notion that the main function of the tear in loggerhead sea turtles is maintenance of stability against the osmotic pressure exerted by the medium [9]. Another significant point was the presence of proteins with structural molecule activity function which, when associated with the main cellular components, further confirms the fact that the tear provides a favorable medium for the microprojections of the corneal epithelium [9, 29].

## Conclusion

This study offers the first description of bird and reptile tear proteomics, with a focus on species inhabiting different ecosystems (aerial, marine and freshwater). A total of 446, 1358 and 163 proteins were identified in roadside hawk, broad-snouted caiman and loggerhead sea turtle tears, respectively. The proteomic profiles of these fluids differed from those of mammalian tears, suggesting that adaptative processes directly influence the composition of tear polypeptides. In reptiles, there was a large number of uncharacterized proteins. The components of roadside hawk tears were primarily destined to molecules and ion transport, indicating the constant need for maintenance of osmolarity and stability. The protein profile of the broad-snouted caiman tears was diverse, with a high abundance of enzymes, a situation that can be related to a close contact with a hypotonic freshwater environment or even to a specific defense role. The ontological classification of proteins in loggerhead sea turtle tears revealed a strong mechanism of osmotic-pressure maintenance.

## Methods

### Ethics approval and consent to participate

This study was approved by and registered at the System of Authorization and Information on Biodiversity (protocol no. 27489) and by the National System of Management of Genetic Heritage (protocol no. A1F8C27), both part of the Brazilian Ministry of the Environment, and by the Ethics Committee on Animal Experimentation of the School of Veterinary Medicine and Zootechnology of UFBA (protocol no. 72/2016) who allowed to use the animals.. All procedures were conducted in compliance with the Association for Vision and Ophthalmology Research (ARVO) and the National Institutes of Health (NIH) for the use of animals in eye and ophthalmic research. In addition, at all stages involving contact with the animal, minimally invasive maneuvers were performed to reduce stress and pain.

### Materials

The following materials were obtained from Sigma-Aldrich (Saint Louis, MO, USA): ACN, TFA, ammonium bicarbonate, DTT, IAA, and trypsin (0.1 μg/μL). Nanopure water was used throughout.

### Animal species included in the experiment

The roadside hawk (*Rupornis magnirostris*) is a hawk with a wide geographical distribution in the America s[46]. This bird is easily handled and tear collection is possible using methods that are employed for domestic animals. The broad-snouted caiman (*Caiman latirostris*) is a medium-sized crocodile, bred for commercial purposes, which lives in freshwater environments and can be found in eastern and central South America. The loggerhead sea turtle (*Caretta caretta*) is considered a vulnerable species according to the current IUCN (International Union for Conservation of Nature’s) Red List Criteria; it is globally distributed throughout the subtropical and temperate regions of the Mediterranean Sea and Pacific, Indian, and Atlantic Ocean s[35]. These wild animal species were chosen because each occupies a different ecological niche, and was available for sampling and clinical evaluation. In addition, they are species routinely found in rescue centers and zoos, and which can be eventually afflicted by diseases that cause damage to the ocular surface.

A total of 10 healthy adult roadside hawks of unknown sex were screened in this study. All birds were kept at the Center for Triage of Wild Animals (Salvador, Brazil) and housed in an outdoor enclosure, with an environmental enrichment like that found in the Atlantic Rainforest. The birds’ diet was based on meat and chicken or slaughtered prey (mice). We screened 70 healthy adult broad-snouted caimans, males and females, kept in a commercial breeding center (Alagoas, Brazil). The animals were kept in freshwater tanks with a water supply without pollutants, and a supervised diet based on chicken and meat. A total of 10 healthy juvenile loggerhead sea turtles of unknown sex were also screened. These animals were kept at a wild animal conservation center (Mata de São João, Brazil), and the tank water supply was originated directly from the sea and filtered using sand filters. The pH of water tanks was around 7.5–8.5. The diet of the sea turtles were based on fish, algae and vegetables. The choice of captive animals was based in the fact that they are used to human handling, making the tear collection ease and with less chances of contamination. All animals were submitted to periodical hematologic exams and had no history of disease. In addition, it complies with national regulations (SISBIO law requirements) and these institutions allowed to use the captive animals.

### Tear collection and sample preparation

The collection procedures were conducted under manual restraint, without the use of anesthetics, and with previous cleaning of the eye with sterile water to avoid contamination. Before sample collection, the institute staff and veterinarian ophthalmologists performed a routine physical examination, and evaluated the eyes and periocular regions using light and a magnifying lens. Other ophthalmic evaluations (such as biomicroscopy and fluorescein test) were performed after acquisition of the samples, as these techniques can influence tear production. If diseases or morphological changes were found, the sample was excluded from the evaluation. Stress factors, such as intense sounds and light stimuli or physical restraints for a long time, and procedures that can induce pain, such as an excessive manipulation of the eyelids and eyelashes, were prevented and reduced with the objective to preserve animal welfare during the sampling process.

All collections were performed in the morning (0800–1130 h), in both eyes. For birds and caimans, the collection was performed by Schirmer strip (Ophthalmos®, São Paulo, Brazil), as previously describe d[5]. The viscous sea turtle tears were collected with a 3-mL disposable syringe (BD®, São Paulo, Brazil). The amount of individual tear samples that can be collected from these animals is very small (around 2 μL) and because of this, the samples were pooled. After this, the pooled samples of each species was frozen at − 80 °C until processing.

### LC-MS analysis

The protein concentration of each of the tear pools was determined, and then they were eluted in buffer solution (30 μL of 50 mM ammonium bicarbonate, 4 μL of 100 mM DTT, 8 μL IAA) and incubated in trypsin at 37 °C for 18 h (overnight). HPLC was performed on a C18 column (ZORBAX Extend C18–80 Å 180 m^2^/g, Agilent, Santa Clara, CA, USA) to separate and collect the peptides. The column was washed and activated with 0.1% (v/v) TFA solution in water, and then ACN solution (80% v/v) and 0.1% TFA were added sequentially. After this step, the sample was reintroduced into the column and washing was performed with 0.1% TFA solution and water; then the sample was eluted with 80% ACN solution and 0.1% TFA in water. The collected peptides were concentrated under vacuum (− 26 °C) for 18 h prior to performing MS.

A high-resolution mass spectrum profile was acquired using an LTQ Orbitrap XL mass spectrometer with electrospray ionization source (ThermoFisher, San Jose, CA, USA) operated in positive mode. Samples were introduced to the source by direct infusion at 10 μL/min and MS was performed through collision-induced dissociation. The precursor ions were isolated from 3 Da, activated from 25 to 35% of the normalized collision energy per 100 ms, and the *qz* value was maintained at 0.250. The ions that were subjected to collision-induced dissociation were transferred to the Orbitrap for MS measurements and spectra were acquired, with a mean 16–53 scans.

### Data analysis

The MS results were evaluated using Byonic Protein Metrics software (Cupertino, CA, USA); polypeptides with a score above log 2 were included, and those below this score were considered foreign proteins. The data were evaluated using the UniProtKB database, with the research criterion set to the taxa Aves (class), Alligatoridae (family) and Testudines (order) for roadside hawk, broad-snouted caiman and loggerhead sea turtle, respectively. The total abundance was obtained, and protein frequencies were described as percentages (relative abundance). The results were organized into clusters, based on the frequency of the protein and it gene ontology category (cellular components, biological processes and molecular functions), as previously describe d[3].

## Supplementary information

**Additional file 1 **Table 1**.** Proteins identified in roadside hawk (*Rupornis magnirostris*) tears. An Orbitrap platform was used to identify the peptides, and the results were analyzed using the taxum Aves database

**Additional file 2 Table 2.** Proteins identified in caiman (*Caiman latirostris*) tears. An Orbitrap platform was used to identify the peptides, and the results were analyzed using the Alligatoridae family database .

**Additional file 3 Table 2.** Proteins identified in caiman (*Caiman latirostris*) tears. An Orbitrap platform was used to identify the peptides, and the results were analyzed using the Alligatoridae family database .

## Data Availability

The datasets used and/or analyzed during the current study are available from the corresponding author on reasonable request.

## Abbreviations

ACN: Acetronile
TFA: Trifluroacetic acid
DTT: Dithiothreitol
IAA: Iodoacetamide
