# Supplementary material for: The proteomics of roadside hawk (Rupornis magnirostris), broad-snouted caiman (Caiman latirostris) and loggerhead sea turtle (Caretta caretta) tears
Source: BMC Vet Res. 2020 Aug 7;16:276. doi: 10.1186/s12917-020-02495-0 (PMC7412644; doi:10.1186/s12917-020-02495-0)
Supplement: Supplementary file 1 — Additional file 1 Table 1. Proteins identified in roadside hawk (Rupornis magnirostris) tears. An Orbitrap platform was used to identify the peptides, and the results were analyzed using the taxum Aves database [file 12917_2020_2495_MOESM1_ESM.docx]

**Table 1. Proteins identified in roadside hawk (*Rupornis magnirostris*) tears.** An Orbitrap platform was used to identify the peptides, and the results were analyzed using the taxum Aves database (UNIPROT KB).

| **n.** | **Description protein** | **Total intensity** | **n. of spectra** | **n. of unique peptides** |
| --- | --- | --- | --- | --- |
| 1 | \|A0A0A0AN62\|A0A0A0AN62_CHAVO Serum albumin OS=Charadrius vociferus GN=N301_09369 PE=3 SV=1 | 32410552678,0 | 915 | 51 |
| 2 | \|U3K0R0\|U3K0R0_FICAL Albumin OS=Ficedula albicollis GN=ALB PE=3 SV=1 | 11124063878,4 | 136 | 9 |
| 3 | \|A0A093GZX5\|A0A093GZX5_GAVST Ovotransferrin (Fragment) OS=Gavia stellata GN=N328_12749 PE=4 SV=1 | 3490955292,7 | 172 | 21 |
| 4 | \|A0A091UTV5\|A0A091UTV5_NIPNI Ig lambda-1 chain C regions (Fragment) OS=Nipponia nippon GN=Y956_06980 PE=4 SV=1 | 2437494224,6 | 45 | 4 |
| 5 | \|A0A091TMQ3\|A0A091TMQ3_PHALP Ig heavy chain V-III region VH26 (Fragment) OS=Phaethon lepturus GN=N335_05354 PE=4 SV=1 | 2387439089,1 | 20 | 1 |
| 6 | \|Q5ZMQ2\|ACTG_CHICK Actin, cytoplasmic 2 OS=Gallus gallus GN=ACTG1 PE=1 SV=1 | 1753053914,5 | 177 | 38 |
| 7 | \|A0A099ZXZ8\|A0A099ZXZ8_CHAVO Ovoinhibitor (Fragment) OS=Charadrius vociferus GN=N301_00610 PE=4 SV=1 | 1517749097,0 | 65 | 12 |
| 8 | \|A0A093P4F7\|A0A093P4F7_PYGAD Ig lambda chain V-1 region (Fragment) OS=Pygoscelis adeliae GN=AS28_08483 PE=4 SV=1 | 1311776425,1 | 47 | 6 |
| 9 | \|A0A093FBG5\|A0A093FBG5_GAVST Ig heavy chain V-III region VH26 (Fragment) OS=Gavia stellata GN=N328_00712 PE=4 SV=1 | 1238986022,7 | 98 | 4 |
| 10 | \|A0A093FHI9\|A0A093FHI9_GAVST Serum albumin OS=Gavia stellata GN=N328_06103 PE=3 SV=1 | 1096271008,2 | 34 | 5 |
| 11 | \|A0A099Z6D9\|A0A099Z6D9_TINGU Nesprin-1 (Fragment) OS=Tinamus guttatus GN=N309_13349 PE=4 SV=1 | 1093174530,4 | 35 | 12 |
| 12 | \|A0A091WH83\|A0A091WH83_NIPNI Serum albumin OS=Nipponia nippon GN=Y956_03391 PE=3 SV=1 | 1088564308,6 | 43 | 2 |
| 13 | \|A0A091J8H4\|A0A091J8H4_9AVES Lysozyme g (Fragment) OS=Egretta garzetta GN=Z169_11799 PE=4 SV=1 | 960926891,5 | 56 | 8 |
| 14 | \|A0A0A0ASU7\|A0A0A0ASU7_CHAVO Alpha-enolase OS=Charadrius vociferus GN=N301_11379 PE=3 SV=1 | 847498957,1 | 160 | 31 |
| 15 | \|A0A091PG89\|A0A091PG89_LEPDC Uncharacterized protein OS=Leptosomus discolor GN=N330_14178 PE=4 SV=1 | 729846523,2 | 76 | 13 |
| 16 | \|A0A093CFV7\|A0A093CFV7_9AVES Ig heavy chain V-III region CAM (Fragment) OS=Pterocles gutturalis GN=N339_05564 PE=4 SV=1 | 727033837,7 | 79 | 3 |
| 17 | \|A0A0Q3LVM5\|A0A0Q3LVM5_AMAAE Apolipoprotein A-I OS=Amazona aestiva GN=AAES_252467 PE=3 SV=1 | 698635163,2 | 87 | 23 |
| 18 | \|A0A091IU87\|A0A091IU87_9AVES Gelsolin (Fragment) OS=Egretta garzetta GN=Z169_04393 PE=4 SV=1 | 496384831,8 | 103 | 26 |
| 19 | \|A0A093NJT8\|A0A093NJT8_PYGAD Ovostatin (Fragment) OS=Pygoscelis adeliae GN=AS28_11983 PE=4 SV=1 | 491626582,1 | 98 | 44 |
| 20 | \|P19121\|ALBU_CHICK Serum albumin OS=Gallus gallus GN=ALB PE=1 SV=2 | 387706180,8 | 37 | 5 |
| 21 | \|A0A091IHJ3\|A0A091IHJ3_CALAN Ig lambda chain V-1 region (Fragment) OS=Calypte anna GN=N300_08157 PE=4 SV=1 | 381298546,4 | 24 | 3 |
| 22 | \|A0A093I422\|A0A093I422_STRCA Serum albumin (Fragment) OS=Struthio camelus australis GN=N308_00941 PE=3 SV=1 | 377938608,0 | 29 | 5 |
| 23 | \|A0A093KM83\|A0A093KM83_FULGA Ovotransferrin (Fragment) OS=Fulmarus glacialis GN=N327_12390 PE=4 SV=1 | 376051219,6 | 39 | 9 |
| 24 | \|A0A093N8L5\|A0A093N8L5_PYGAD Plasminogen (Fragment) OS=Pygoscelis adeliae GN=AS28_07462 PE=3 SV=1 | 369813373,2 | 73 | 19 |
| 25 | \|A0A091Q5U4\|A0A091Q5U4_LEPDC Glutathione S-transferase (Fragment) OS=Leptosomus discolor GN=N330_06991 PE=3 SV=1 | 360972371,3 | 40 | 15 |
| 26 | \|A0A0Q3PLB4\|A0A0Q3PLB4_AMAAE Antithrombin-III OS=Amazona aestiva GN=AAES_130795 PE=3 SV=1 | 349211447,1 | 67 | 19 |
| 27 | \|A0A0A0ADU8\|A0A0A0ADU8_CHAVO Alpha-2-macroglobulin-like 1 (Fragment) OS=Charadrius vociferus GN=N301_11515 PE=4 SV=1 | 334370134,7 | 57 | 15 |
| 28 | \|A0A091WKF3\|A0A091WKF3_NIPNI Pyruvate kinase (Fragment) OS=Nipponia nippon GN=Y956_05437 PE=3 SV=1 | 328065408,3 | 101 | 37 |
| 29 | \|A0PA16\|A0PA16_COTJA Heat shock protein 70kDa OS=Coturnix japonica GN=HSPA8 PE=2 SV=1 | 323496535,0 | 99 | 29 |
| 30 | \|Q5ZMD1\|1433T_CHICK 14-3-3 protein theta OS=Gallus gallus GN=YWHAQ PE=1 SV=1 | 311013553,7 | 63 | 17 |
| 31 | \|A0A093SFT9\|A0A093SFT9_9PASS Ig lambda chain V-1 region (Fragment) OS=Manacus vitellinus GN=N305_06010 PE=4 SV=1 | 286078099,1 | 19 | 1 |
| 32 | \|A0A091I8D1\|A0A091I8D1_CALAN Fatty acid-binding protein, heart (Fragment) OS=Calypte anna GN=N300_09425 PE=3 SV=1 | 278383485,7 | 25 | 7 |
| 33 | \|A0A091QSZ2\|A0A091QSZ2_LEPDC Vitamin D-binding protein (Fragment) OS=Leptosomus discolor GN=N330_03780 PE=3 SV=1 | 276927493,4 | 46 | 13 |
| 34 | \|A0A091TMZ0\|A0A091TMZ0_PHALP Aldo-keto reductase family 1 member B10 (Fragment) OS=Phaethon lepturus GN=N335_01469 PE=4 SV=1 | 261029477,4 | 46 | 13 |
| 35 | \|A0A091VJ06\|A0A091VJ06_NIPNI Alpha-actinin-4 (Fragment) OS=Nipponia nippon GN=Y956_07286 PE=4 SV=1 | 254737028,1 | 67 | 29 |
| 36 | \|Q5ZMT0\|1433E_CHICK 14-3-3 protein epsilon OS=Gallus gallus GN=YWHAE PE=1 SV=1 | 244691955,7 | 48 | 21 |
| 37 | \|A0A0A0AE51\|A0A0A0AE51_CHAVO Lysozyme g (Fragment) OS=Charadrius vociferus GN=N301_16526 PE=4 SV=1 | 244046492,5 | 12 | 3 |
| 38 | \|A0A093NMA3\|A0A093NMA3_PYGAD Ig lambda chain V-1 region (Fragment) OS=Pygoscelis adeliae GN=AS28_03496 PE=4 SV=1 | 234501040,0 | 18 | 3 |
| 39 | \|A0A091J8G3\|A0A091J8G3_9AVES Peroxiredoxin-1 (Fragment) OS=Egretta garzetta GN=Z169_02194 PE=4 SV=1 | 230768624,8 | 58 | 14 |
| 40 | \|A0A091IBA6\|A0A091IBA6_CALAN Apolipoprotein A-I (Fragment) OS=Calypte anna GN=N300_01377 PE=3 SV=1 | 229557240,8 | 5 | 2 |
| 41 | \|Q9DET5\|TB15A_COTJA Thymosin beta-15A homolog OS=Coturnix japonica PE=3 SV=3 | 224311020,7 | 16 | 4 |
| 42 | \|A0A0A0AKU1\|A0A0A0AKU1_CHAVO Vitamin D-binding protein (Fragment) OS=Charadrius vociferus GN=N301_14254 PE=3 SV=1 | 223523582,5 | 16 | 4 |
| 43 | \|A0A0Q3TQY2\|A0A0Q3TQY2_AMAAE Transcription initiation factor TFIID subunit 1 isoform X1 OS=Amazona aestiva GN=AAES_26050 PE=4 SV=1 | 219233448,8 | 6 | 3 |
| 44 | \|A0A093FRQ2\|A0A093FRQ2_GAVST Ezrin (Fragment) OS=Gavia stellata GN=N328_11245 PE=4 SV=1 | 206762943,4 | 56 | 21 |
| 45 | \|R7VRC4\|R7VRC4_COLLI Complement C3 OS=Columba livia GN=A306_14901 PE=4 SV=1 | 206757796,4 | 46 | 16 |
| 46 | \|A0A093G4R8\|A0A093G4R8_PICPB Beta-microseminoprotein (Fragment) OS=Picoides pubescens GN=N307_06108 PE=4 SV=1 | 203099468,1 | 7 | 1 |
| 47 | \|A0A091UPZ3\|A0A091UPZ3_PHALP Serum albumin OS=Phaethon lepturus GN=N335_06786 PE=3 SV=1 | 201741804,6 | 26 | 6 |
| 48 | \|A0A091UEL8\|A0A091UEL8_PHORB Ovotransferrin (Fragment) OS=Phoenicopterus ruber ruber GN=N337_09335 PE=4 SV=1 | 199040289,8 | 7 | 1 |
| 49 | \|A0A091HBG2\|A0A091HBG2_BUCRH Ovotransferrin (Fragment) OS=Buceros rhinoceros silvestris GN=N320_04456 PE=4 SV=1 | 198868669,4 | 30 | 7 |
| 50 | \|A0A091I8G9\|A0A091I8G9_CALAN Serum albumin OS=Calypte anna GN=N300_02121 PE=3 SV=1 | 198818465,0 | 10 | 3 |
| 51 | \|A0A093GJM8\|A0A093GJM8_PICPB Ovoinhibitor (Fragment) OS=Picoides pubescens GN=N307_13738 PE=4 SV=1 | 194927788,5 | 10 | 2 |
| 52 | \|A0A091ULQ8\|A0A091ULQ8_NIPNI Beta-microseminoprotein (Fragment) OS=Nipponia nippon GN=Y956_01553 PE=4 SV=1 | 193678123,1 | 7 | 2 |
| 53 | \|A0A093FK84\|A0A093FK84_GAVST Ig lambda chain V-1 region (Fragment) OS=Gavia stellata GN=N328_05684 PE=4 SV=1 | 192720259,6 | 24 | 2 |
| 54 | \|U3K163\|U3K163_FICAL Joining chain of multimeric IgA and IgM OS=Ficedula albicollis GN=JCHAIN PE=4 SV=1 | 190336184,9 | 10 | 2 |
| 55 | \|A0A093FKN9\|A0A093FKN9_GAVST Heparin cofactor 2 (Fragment) OS=Gavia stellata GN=N328_02668 PE=3 SV=1 | 187047341,8 | 38 | 12 |
| 56 | \|A0A0Q3US23\|A0A0Q3US23_AMAAE Kininogen-1 OS=Amazona aestiva GN=AAES_100461 PE=4 SV=1 | 186488549,2 | 24 | 7 |
| 57 | \|P68059\|HBAD_AEGMO Hemoglobin subunit alpha-D OS=Aegypius monachus GN=HBAD PE=1 SV=1 | 184975966,5 | 30 | 6 |
| 58 | \|A0A093FV96\|A0A093FV96_GAVST Ig lambda chain V-1 region (Fragment) OS=Gavia stellata GN=N328_07412 PE=4 SV=1 | 184183321,6 | 28 | 1 |
| 59 | \|A0A091NPM5\|A0A091NPM5_APAVI Serum albumin (Fragment) OS=Apaloderma vittatum GN=N311_04327 PE=3 SV=1 | 181206429,6 | 8 | 3 |
| 60 | \|A0A093F8T4\|A0A093F8T4_GAVST Ig heavy chain V region 5A (Fragment) OS=Gavia stellata GN=N328_03139 PE=4 SV=1 | 180227005,7 | 45 | 2 |
| 61 | \|A0A091V150\|A0A091V150_NIPNI Fructose-bisphosphate aldolase (Fragment) OS=Nipponia nippon GN=Y956_14710 PE=3 SV=1 | 172886738,0 | 55 | 19 |
| 62 | \|A0A091HYW8\|A0A091HYW8_CALAN Inter-alpha-trypsin inhibitor heavy chain H2 (Fragment) OS=Calypte anna GN=N300_00514 PE=4 SV=1 | 172032938,7 | 41 | 18 |
| 63 | \|A0A091HI50\|A0A091HI50_BUCRH Glyceraldehyde-3-phosphate dehydrogenase (Fragment) OS=Buceros rhinoceros silvestris GN=N320_05893 PE=3 SV=1 | 162738093,6 | 30 | 11 |
| 64 | \|U3K8K1\|U3K8K1_FICAL Transaldolase OS=Ficedula albicollis GN=TALDO1 PE=3 SV=1 | 157111416,8 | 27 | 12 |
| 65 | \|A0A093SFV1\|A0A093SFV1_9PASS Ig lambda chain C region (Fragment) OS=Manacus vitellinus GN=N305_06021 PE=4 SV=1 | 156366476,4 | 6 | 2 |
| 66 | \|Q5ZKC9\|1433Z_CHICK 14-3-3 protein zeta OS=Gallus gallus GN=YWHAZ PE=2 SV=1 | 153674552,8 | 34 | 12 |
| 67 | \|A0A0Q3T9U3\|A0A0Q3T9U3_AMAAE Uncharacterized protein OS=Amazona aestiva GN=AAES_183830 PE=4 SV=1 | 150872028,6 | 33 | 13 |
| 68 | \|A0A093QUA9\|A0A093QUA9_PYGAD Ceruloplasmin (Fragment) OS=Pygoscelis adeliae GN=AS28_05814 PE=4 SV=1 | 147072708,2 | 43 | 16 |
| 69 | \|A0A093PH41\|A0A093PH41_PYGAD Deleted in malignant brain tumors 1 protein (Fragment) OS=Pygoscelis adeliae GN=AS28_08025 PE=4 SV=1 | 146325727,9 | 49 | 10 |
| 70 | \|A0A093DDT4\|A0A093DDT4_CHAPE Peptidyl-prolyl cis-trans isomerase (Fragment) OS=Chaetura pelagica GN=M959_06853 PE=3 SV=1 | 143630388,9 | 61 | 15 |
| 71 | \|A0A091NH36\|A0A091NH36_APAVI Ig lambda chain V-1 region (Fragment) OS=Apaloderma vittatum GN=N311_05416 PE=4 SV=1 | 142930040,0 | 18 | 3 |
| 72 | \|A0A093P9B6\|A0A093P9B6_PYGAD Alpha-1-antiproteinase 2 (Fragment) OS=Pygoscelis adeliae GN=AS28_10539 PE=3 SV=1 | 137648015,6 | 28 | 11 |
| 73 | \|A0A091VQC7\|A0A091VQC7_NIPNI Lysozyme g (Fragment) OS=Nipponia nippon GN=Y956_02766 PE=4 SV=1 | 136516630,0 | 18 | 3 |
| 74 | \|A0A0A0A1J2\|A0A0A0A1J2_CHAVO Alpha-2-macroglobulin (Fragment) OS=Charadrius vociferus GN=N301_12834 PE=4 SV=1 | 134290653,0 | 66 | 18 |
| 75 | \|A0A091HVD5\|A0A091HVD5_CALAN Gelsolin (Fragment) OS=Calypte anna GN=N300_09373 PE=4 SV=1 | 133704455,8 | 4 | 1 |
| 76 | \|A0A091Q3D7\|A0A091Q3D7_LEPDC Aldo-keto reductase family 1 member B10 OS=Leptosomus discolor GN=N330_02348 PE=4 SV=1 | 130270370,9 | 11 | 4 |
| 77 | \|Q5ZLQ6\|1433B_CHICK 14-3-3 protein beta/alpha OS=Gallus gallus GN=YWHAB PE=2 SV=1 | 126728023,8 | 21 | 8 |
| 78 | \|A0A091V999\|A0A091V999_NIPNI Heat shock protein HSP 90-alpha OS=Nipponia nippon GN=Y956_11615 PE=3 SV=1 | 123395345,2 | 39 | 20 |
| 79 | \|A0A0Q3PMK9\|A0A0Q3PMK9_AMAAE Phosphoglycerate mutase OS=Amazona aestiva GN=AAES_129103 PE=3 SV=1 | 120621745,6 | 28 | 11 |
| 80 | \|A0A091VZ15\|A0A091VZ15_NIPNI Nucleoside diphosphate kinase (Fragment) OS=Nipponia nippon GN=Y956_12156 PE=3 SV=1 | 114694898,1 | 27 | 9 |
| 81 | \|A0A091WPP2\|A0A091WPP2_NIPNI 14-3-3 protein sigma (Fragment) OS=Nipponia nippon GN=Y956_14887 PE=3 SV=1 | 114694864,8 | 23 | 9 |
| 82 | \|P68063\|HBB_GYPRU Hemoglobin subunit beta OS=Gyps rueppellii GN=HBB PE=1 SV=1 | 108604738,4 | 18 | 6 |
| 83 | \|A0A0A0B3E6\|A0A0A0B3E6_CHAVO Ig lambda chain V-1 region (Fragment) OS=Charadrius vociferus GN=N301_16811 PE=4 SV=1 | 108505896,8 | 17 | 3 |
| 84 | \|Q90835\|EF1A_CHICK Elongation factor 1-alpha 1 OS=Gallus gallus GN=EEF1A PE=2 SV=1 | 107723219,5 | 40 | 20 |
| 85 | \|A0A091VQ86\|A0A091VQ86_NIPNI Elongation factor 2 (Fragment) OS=Nipponia nippon GN=Y956_14260 PE=4 SV=1 | 107397588,8 | 39 | 20 |
| 86 | \|A0A091GQJ0\|A0A091GQJ0_BUCRH Uncharacterized protein (Fragment) OS=Buceros rhinoceros silvestris GN=N320_04888 PE=4 SV=1 | 106062966,0 | 12 | 1 |
| 87 | \|A0A091Q7L5\|A0A091Q7L5_LEPDC Myeloperoxidase (Fragment) OS=Leptosomus discolor GN=N330_08999 PE=4 SV=1 | 105804527,4 | 24 | 7 |
| 88 | \|R0LRM7\|R0LRM7_ANAPL Destrin (Fragment) OS=Anas platyrhynchos GN=DSTN PE=3 SV=1 | 104743555,3 | 25 | 9 |
| 89 | \|A0A0A0A3R1\|A0A0A0A3R1_CHAVO Apolipoprotein A-I (Fragment) OS=Charadrius vociferus GN=N301_03716 PE=3 SV=1 | 99068894,6 | 16 | 3 |
| 90 | \|A0A093FNM0\|A0A093FNM0_GAVST Annexin OS=Gavia stellata GN=N328_11448 PE=3 SV=1 | 98151563,3 | 25 | 11 |
| 91 | \|P12003\|VINC_CHICK Vinculin OS=Gallus gallus GN=VCL PE=1 SV=4 | 96107939,4 | 18 | 12 |
| 92 | \|A0A091NSR5\|A0A091NSR5_APAVI Deleted in malignant brain tumors 1 protein (Fragment) OS=Apaloderma vittatum GN=N311_12463 PE=4 SV=1 | 94613541,4 | 15 | 2 |
| 93 | \|P62149\|CALM_CHICK Calmodulin OS=Gallus gallus GN=CALM PE=1 SV=2 | 94439481,5 | 21 | 7 |
| 94 | \|A0A091UAT7\|A0A091UAT7_PHORB Glycerol-3-phosphate dehydrogenase [NAD(+)] (Fragment) OS=Phoenicopterus ruber ruber GN=N337_13306 PE=3 SV=1 | 91973942,6 | 16 | 7 |
| 95 | \|A0A093QM44\|A0A093QM44_9PASS Annexin (Fragment) OS=Manacus vitellinus GN=N305_12184 PE=3 SV=1 | 89676445,1 | 26 | 9 |
| 96 | \|Q5ZMU9\|Q5ZMU9_CHICK Valosin containing protein OS=Gallus gallus GN=VCP PE=1 SV=1 | 89608051,7 | 34 | 18 |
| 97 | \|A0A093BJI5\|A0A093BJI5_9AVES Complement C3 (Fragment) OS=Pterocles gutturalis GN=N339_04591 PE=4 SV=1 | 89486025,0 | 10 | 3 |
| 98 | \|A0A0Q3X8S6\|A0A0Q3X8S6_AMAAE L-lactate dehydrogenase OS=Amazona aestiva GN=AAES_27127 PE=3 SV=1 | 88915839,8 | 24 | 13 |
| 99 | \|P07404\|HBA_VULGR Hemoglobin subunit alpha-A OS=Vultur gryphus GN=HBAA PE=1 SV=1 | 88807996,0 | 8 | 1 |
| 100 | \|A0A093RKW8\|A0A093RKW8_PYGAD Alpha-2-macroglobulin (Fragment) OS=Pygoscelis adeliae GN=AS28_11805 PE=4 SV=1 | 88190431,8 | 20 | 5 |
| 101 | \|A0A091UAC8\|A0A091UAC8_PHALP Transketolase (Fragment) OS=Phaethon lepturus GN=N335_06008 PE=4 SV=1 | 87588589,4 | 42 | 13 |
| 102 | \|A0A091H219\|A0A091H219_BUCRH Alpha-2-macroglobulin-like 1 (Fragment) OS=Buceros rhinoceros silvestris GN=N320_12354 PE=4 SV=1 | 81325158,8 | 9 | 2 |
| 103 | \|F1NPG2\|F1NPG2_CHICK Isocitrate dehydrogenase [NADP] OS=Gallus gallus GN=IDH1 PE=3 SV=2 | 81037708,2 | 22 | 12 |
| 104 | \|A0A091FZ47\|A0A091FZ47_9AVES Alpha-2-HS-glycoprotein OS=Cuculus canorus GN=N303_10297 PE=3 SV=1 | 80129725,7 | 9 | 3 |
| 105 | \|R0LC19\|R0LC19_ANAPL Heat shock 70 kDa protein (Fragment) OS=Anas platyrhynchos GN=HSPA2 PE=3 SV=1 | 79798243,0 | 38 | 16 |
| 106 | \|Q2TK69\|Q2TK69_TYMCU Beta-actin (Fragment) OS=Tympanuchus cupido PE=4 SV=1 | 79315777,2 | 4 | 2 |
| 107 | \|A0A091V9M7\|A0A091V9M7_NIPNI Hemopexin (Fragment) OS=Nipponia nippon GN=Y956_09520 PE=4 SV=1 | 78924152,8 | 20 | 2 |
| 108 | \|U3JYU6\|U3JYU6_FICAL Prothymosin, alpha OS=Ficedula albicollis GN=PTMA PE=4 SV=1 | 78067344,1 | 20 | 4 |
| 109 | \|A0A091TTJ2\|A0A091TTJ2_PHALP Thioredoxin (Fragment) OS=Phaethon lepturus GN=N335_12583 PE=4 SV=1 | 77564485,1 | 12 | 6 |
| 110 | \|A0A091G8N5\|A0A091G8N5_9AVES Glutathione S-transferase Mu 3 (Fragment) OS=Cuculus canorus GN=N303_12930 PE=4 SV=1 | 77108980,8 | 24 | 12 |
| 111 | \|A0A091HUJ9\|A0A091HUJ9_CALAN Adenosylhomocysteinase (Fragment) OS=Calypte anna GN=N300_10797 PE=3 SV=1 | 72518228,4 | 32 | 13 |
| 112 | \|A0A091W6T3\|A0A091W6T3_NIPNI Glucose-6-phosphate isomerase (Fragment) OS=Nipponia nippon GN=Y956_02624 PE=3 SV=1 | 72484150,7 | 33 | 14 |
| 113 | \|A0A0Q3XCN4\|A0A0Q3XCN4_AMAAE Mesothelin isoform X4 OS=Amazona aestiva GN=AAES_12491 PE=4 SV=1 | 70859329,3 | 19 | 4 |
| 114 | \|A0A091UX80\|A0A091UX80_NIPNI Rab GDP dissociation inhibitor (Fragment) OS=Nipponia nippon GN=Y956_06188 PE=3 SV=1 | 70164828,0 | 39 | 21 |
| 115 | \|P00940\|TPIS_CHICK Triosephosphate isomerase OS=Gallus gallus GN=TPI1 PE=1 SV=2 | 69566347,5 | 31 | 10 |
| 116 | \|U3KGY5\|U3KGY5_FICAL Uncharacterized protein OS=Ficedula albicollis GN=LOC101809215 PE=4 SV=1 | 68201445,1 | 11 | 2 |
| 117 | \|A0A091TEE3\|A0A091TEE3_PHALP Beta-microseminoprotein (Fragment) OS=Phaethon lepturus GN=N335_13751 PE=4 SV=1 | 66011237,4 | 28 | 8 |
| 118 | \|U3K852\|U3K852_FICAL Guanine deaminase OS=Ficedula albicollis GN=GDA PE=4 SV=1 | 65247873,0 | 12 | 5 |
| 119 | \|A0A091GLR6\|A0A091GLR6_9AVES Fibronectin (Fragment) OS=Cuculus canorus GN=N303_04602 PE=4 SV=1 | 65128558,5 | 24 | 18 |
| 120 | \|A0A091W380\|A0A091W380_NIPNI Serpin B5 OS=Nipponia nippon GN=Y956_11926 PE=3 SV=1 | 64064282,8 | 21 | 10 |
| 121 | \|P18625\|FOSL2_CHICK Fos-related antigen 2 OS=Gallus gallus GN=FOSL2 PE=1 SV=1 | 63592055,5 | 9 | 3 |
| 122 | \|A0A093NCN3\|A0A093NCN3_PYGAD Cytosolic non-specific dipeptidase (Fragment) OS=Pygoscelis adeliae GN=AS28_02553 PE=4 SV=1 | 60909584,3 | 30 | 11 |
| 123 | \|A0A091IJP1\|A0A091IJP1_CALAN Ig lambda chain V-1 region (Fragment) OS=Calypte anna GN=N300_08158 PE=4 SV=1 | 60425523,4 | 16 | 2 |
| 124 | \|A0A0A0A5U4\|A0A0A0A5U4_CHAVO Pantetheinase (Fragment) OS=Charadrius vociferus GN=N301_15620 PE=4 SV=1 | 59940324,7 | 13 | 4 |
| 125 | \|A0A091N6E4\|A0A091N6E4_9PASS Ovoinhibitor (Fragment) OS=Acanthisitta chloris GN=N310_12682 PE=4 SV=1 | 59656494,3 | 9 | 2 |
| 126 | \|Q8JFP1\|IF4A2_CHICK Eukaryotic initiation factor 4A-II OS=Gallus gallus GN=EIF4A2 PE=2 SV=1 | 59360414,9 | 25 | 12 |
| 127 | \|A0A0A0AG24\|A0A0A0AG24_CHAVO Vitronectin (Fragment) OS=Charadrius vociferus GN=N301_12375 PE=4 SV=1 | 58863592,1 | 21 | 8 |
| 128 | \|A0A093Q8F2\|A0A093Q8F2_9PASS Protein S100-A4 OS=Manacus vitellinus GN=N305_14382 PE=4 SV=1 | 57582125,8 | 17 | 5 |
| 129 | \|P09244\|TBB7_CHICK Tubulin beta-7 chain OS=Gallus gallus PE=2 SV=1 | 57506287,1 | 23 | 11 |
| 130 | \|A0A091V2L9\|A0A091V2L9_NIPNI Bleomycin hydrolase (Fragment) OS=Nipponia nippon GN=Y956_11599 PE=4 SV=1 | 57005361,3 | 22 | 8 |
| 131 | \|A0A091W0N8\|A0A091W0N8_NIPNI Transketolase OS=Nipponia nippon GN=Y956_15231 PE=4 SV=1 | 56896966,1 | 6 | 3 |
| 132 | \|A0A091W4S3\|A0A091W4S3_NIPNI Myeloperoxidase (Fragment) OS=Nipponia nippon GN=Y956_15277 PE=4 SV=1 | 56770677,0 | 28 | 12 |
| 133 | \|A0A093PB72\|A0A093PB72_9PASS Pyridoxal kinase (Fragment) OS=Manacus vitellinus GN=N305_07655 PE=4 SV=1 | 56670838,6 | 19 | 9 |
| 134 | \|A0A093FFN5\|A0A093FFN5_GAVST Ovostatin (Fragment) OS=Gavia stellata GN=N328_11435 PE=4 SV=1 | 56174286,8 | 7 | 2 |
| 135 | \|A0A091UV65\|A0A091UV65_NIPNI 6-phosphogluconate dehydrogenase, decarboxylating (Fragment) OS=Nipponia nippon GN=Y956_10722 PE=3 SV=1 | 54288593,9 | 30 | 13 |
| 136 | \|U3K9W1\|U3K9W1_FICAL Complement C3 OS=Ficedula albicollis GN=C3 PE=4 SV=1 | 53717743,3 | 24 | 8 |
| 137 | \|L1JFU9\|L1JFU9_GUITH Fructose-bisphosphate aldolase OS=Guillardia theta CCMP2712 GN=GUITHDRAFT_86602 PE=3 SV=1 | 53139861,1 | 7 | 1 |
| 138 | \|A0A091VHD0\|A0A091VHD0_NIPNI Protein disulfide-isomerase (Fragment) OS=Nipponia nippon GN=Y956_09608 PE=3 SV=1 | 52571714,1 | 15 | 8 |
| 139 | \|A0A0A0ABQ0\|A0A0A0ABQ0_CHAVO Glutathione S-transferase 3 OS=Charadrius vociferus GN=N301_01082 PE=4 SV=1 | 51019320,6 | 7 | 3 |
| 140 | \|A0A093NLF3\|A0A093NLF3_PYGAD Beta-microseminoprotein (Fragment) OS=Pygoscelis adeliae GN=AS28_04497 PE=4 SV=1 | 49979259,7 | 5 | 2 |
| 141 | \|A0A093FN72\|A0A093FN72_GAVST Beta-microseminoprotein (Fragment) OS=Gavia stellata GN=N328_06402 PE=4 SV=1 | 49979259,7 | 5 | 2 |
| 142 | \|A0A091VYS2\|A0A091VYS2_NIPNI Complement factor I (Fragment) OS=Nipponia nippon GN=Y956_00911 PE=3 SV=1 | 49720744,2 | 10 | 5 |
| 143 | \|A0A091I6F7\|A0A091I6F7_CALAN Lysozyme g (Fragment) OS=Calypte anna GN=N300_03186 PE=4 SV=1 | 48884634,1 | 3 | 1 |
| 144 | \|P92493\|CYB_APHCE Cytochrome b OS=Aphelocoma coerulescens GN=MT-CYB PE=3 SV=1 | 48196392,9 | 4 | 1 |
| 145 | \|A0A091JW70\|A0A091JW70_COLST Vitamin D-binding protein (Fragment) OS=Colius striatus GN=N325_11871 PE=3 SV=1 | 47980182,0 | 7 | 2 |
| 146 | \|A0A091UIM2\|A0A091UIM2_NIPNI Uncharacterized protein OS=Nipponia nippon GN=Y956_15915 PE=4 SV=1 | 47219618,0 | 10 | 5 |
| 147 | \|A0A093IG35\|A0A093IG35_FULGA Angiotensinogen (Fragment) OS=Fulmarus glacialis GN=N327_03026 PE=3 SV=1 | 46892782,3 | 19 | 7 |
| 148 | \|A0A091VZK3\|A0A091VZK3_NIPNI Homeodomain-only protein OS=Nipponia nippon GN=Y956_01000 PE=4 SV=1 | 46833585,2 | 13 | 3 |
| 149 | \|A0A091VNE3\|A0A091VNE3_NIPNI Protein S100-A4 OS=Nipponia nippon GN=Y956_08539 PE=4 SV=1 | 46663734,3 | 18 | 6 |
| 150 | \|R7VWS2\|R7VWS2_COLLI Tubulin alpha chain (Fragment) OS=Columba livia GN=A306_07239 PE=3 SV=1 | 46639755,1 | 29 | 10 |
| 151 | \|A0A091U489\|A0A091U489_PHORB Prothrombin (Fragment) OS=Phoenicopterus ruber ruber GN=N337_11693 PE=4 SV=1 | 45102766,7 | 12 | 6 |
| 152 | \|A0A093BW72\|A0A093BW72_TAUER Anterior gradient protein 2 OS=Tauraco erythrolophus GN=N340_14833 PE=4 SV=1 | 44966138,9 | 17 | 5 |
| 153 | \|Q90WD0\|ARP3_CHICK Actin-related protein 3 OS=Gallus gallus GN=ACTR3 PE=2 SV=1 | 44516472,7 | 28 | 15 |
| 154 | \|R0KA42\|R0KA42_ANAPL Rho GDP dissociation inhibitor alpha (Fragment) OS=Anas platyrhynchos GN=ARHGDIA PE=4 SV=1 | 44396180,5 | 17 | 6 |
| 155 | \|A0A093PAA1\|A0A093PAA1_PYGAD Aldo-keto reductase family 1 member B10 (Fragment) OS=Pygoscelis adeliae GN=AS28_09060 PE=4 SV=1 | 44358993,9 | 15 | 8 |
| 156 | \|A0A1V4KKD7\|A0A1V4KKD7_PATFA Uncharacterized protein OS=Patagioenas fasciata monilis GN=AV530_007055 PE=4 SV=1 | 44188175,1 | 8 | 3 |
| 157 | \|A0A091UZ54\|A0A091UZ54_NIPNI Phosphatidylethanolamine-binding protein 1 OS=Nipponia nippon GN=Y956_15794 PE=4 SV=1 | 44187033,1 | 21 | 8 |
| 158 | \|A0A093HAG3\|A0A093HAG3_STRCA Uncharacterized protein (Fragment) OS=Struthio camelus australis GN=N308_15696 PE=3 SV=1 | 44169002,6 | 15 | 6 |
| 159 | \|A0A091H639\|A0A091H639_BUCRH Retinol-binding protein 4 (Fragment) OS=Buceros rhinoceros silvestris GN=N320_02923 PE=4 SV=1 | 43550598,3 | 41 | 9 |
| 160 | \|A0A0A0ADA9\|A0A0A0ADA9_CHAVO Adenylyl cyclase-associated protein (Fragment) OS=Charadrius vociferus GN=N301_15281 PE=3 SV=1 | 42668962,4 | 18 | 10 |
| 161 | \|A0A093PYY7\|A0A093PYY7_9PASS Ig lambda chain V-1 region (Fragment) OS=Manacus vitellinus GN=N305_06017 PE=4 SV=1 | 42600113,4 | 6 | 2 |
| 162 | \|A0A093NP35\|A0A093NP35_PYGAD Stromelysin-1 (Fragment) OS=Pygoscelis adeliae GN=AS28_09250 PE=3 SV=1 | 41928731,3 | 16 | 5 |
| 163 | \|A0A091SCH1\|A0A091SCH1_NESNO Ovotransferrin (Fragment) OS=Nestor notabilis GN=N333_06896 PE=4 SV=1 | 41646283,3 | 9 | 3 |
| 164 | \|A0A091JHM6\|A0A091JHM6_9AVES Ezrin (Fragment) OS=Egretta garzetta GN=Z169_09587 PE=4 SV=1 | 41565300,3 | 6 | 2 |
| 165 | \|A0A091IQQ7\|A0A091IQQ7_9AVES Protein disulfide-isomerase (Fragment) OS=Egretta garzetta GN=Z169_09302 PE=4 SV=1 | 41468468,5 | 17 | 9 |
| 166 | \|A0A091T667\|A0A091T667_PHALP Ovostatin (Fragment) OS=Phaethon lepturus GN=N335_05379 PE=4 SV=1 | 40829929,1 | 16 | 8 |
| 167 | \|B5G2R5\|B5G2R5_TAEGU Peptidylprolyl isomerase OS=Taeniopygia guttata PE=2 SV=1 | 40557365,0 | 8 | 3 |
| 168 | \|A0A093NV14\|A0A093NV14_PYGAD Complement factor H (Fragment) OS=Pygoscelis adeliae GN=AS28_11201 PE=4 SV=1 | 38934951,7 | 18 | 9 |
| 169 | \|A0A091URA2\|A0A091URA2_NIPNI Deleted in malignant brain tumors 1 protein (Fragment) OS=Nipponia nippon GN=Y956_10011 PE=4 SV=1 | 38784095,8 | 12 | 2 |
| 170 | \|U3JY76\|U3JY76_FICAL Uncharacterized protein OS=Ficedula albicollis GN=LOC107603309 PE=4 SV=1 | 38507550,1 | 6 | 2 |
| 171 | \|A0A091QSE0\|A0A091QSE0_LEPDC Vitelline membrane outer layer protein 1 (Fragment) OS=Leptosomus discolor GN=N330_12483 PE=4 SV=1 | 38067922,5 | 6 | 2 |
| 172 | \|Q5ZME2\|MDHC_CHICK Malate dehydrogenase, cytoplasmic OS=Gallus gallus GN=MDH1 PE=2 SV=1 | 37065014,0 | 32 | 10 |
| 173 | \|A0A091VTR8\|A0A091VTR8_NIPNI 78 kDa glucose-regulated protein (Fragment) OS=Nipponia nippon GN=Y956_02195 PE=3 SV=1 | 36512330,2 | 18 | 9 |
| 174 | \|U3JK93\|U3JK93_FICAL Ovotransferrin OS=Ficedula albicollis GN=TF PE=3 SV=1 | 36508852,9 | 27 | 4 |
| 175 | \|A0A091T5V5\|A0A091T5V5_NESNO Plasminogen (Fragment) OS=Nestor notabilis GN=N333_00817 PE=3 SV=1 | 36422011,2 | 9 | 3 |
| 176 | \|A0A091UMI1\|A0A091UMI1_PHALP Beta-2-glycoprotein 1 (Fragment) OS=Phaethon lepturus GN=N335_06383 PE=4 SV=1 | 35717699,7 | 16 | 6 |
| 177 | \|A0A091V8N3\|A0A091V8N3_NIPNI Alpha-1-antiproteinase 2 (Fragment) OS=Nipponia nippon GN=Y956_05068 PE=3 SV=1 | 35690439,7 | 10 | 3 |
| 178 | \|B5G4T1\|B5G4T1_TAEGU Putative ubiquitin-conjugating enzyme E2N OS=Taeniopygia guttata PE=2 SV=1 | 35431083,0 | 7 | 5 |
| 179 | \|A0A091IVB0\|A0A091IVB0_9AVES Uncharacterized protein (Fragment) OS=Egretta garzetta GN=Z169_12208 PE=4 SV=1 | 34673732,6 | 15 | 7 |
| 180 | \|B5G350\|B5G350_TAEGU Putative calreticulin variant 2 (Fragment) OS=Taeniopygia guttata PE=2 SV=1 | 34469834,8 | 15 | 8 |
| 181 | \|A0A091VMW6\|A0A091VMW6_NIPNI Nucleobindin-2 OS=Nipponia nippon GN=Y956_06773 PE=4 SV=1 | 34370924,9 | 15 | 9 |
| 182 | \|A0A0A0B2C1\|A0A0A0B2C1_CHAVO Deleted in malignant brain tumors 1 protein (Fragment) OS=Charadrius vociferus GN=N301_09469 PE=4 SV=1 | 33391733,5 | 8 | 1 |
| 183 | \|P31696\|AGRIN_CHICK Agrin OS=Gallus gallus GN=AGRN PE=1 SV=3 | 32831800,7 | 14 | 7 |
| 184 | \|P41263\|RET4_CHICK Retinol-binding protein 4 OS=Gallus gallus GN=RBP4 PE=1 SV=1 | 32584385,7 | 4 | 2 |
| 185 | \|A0A093CAR3\|A0A093CAR3_9AVES Complement receptor type 2 (Fragment) OS=Pterocles gutturalis GN=N339_08123 PE=4 SV=1 | 31831332,1 | 9 | 3 |
| 186 | \|P16580\|GLNA_CHICK Glutamine synthetase OS=Gallus gallus GN=GLUL PE=1 SV=1 | 31453639,5 | 5 | 3 |
| 187 | \|A0A091Q3I8\|A0A091Q3I8_LEPDC Actin, alpha skeletal muscle B OS=Leptosomus discolor GN=N330_12027 PE=3 SV=1 | 31430276,5 | 18 | 2 |
| 188 | \|A0A091VVM2\|A0A091VVM2_NIPNI Protein DJ-1 OS=Nipponia nippon GN=Y956_10608 PE=4 SV=1 | 30865271,3 | 18 | 8 |
| 189 | \|A0A091H222\|A0A091H222_BUCRH Gelsolin (Fragment) OS=Buceros rhinoceros silvestris GN=N320_02544 PE=4 SV=1 | 30683908,9 | 10 | 2 |
| 190 | \|A0A093C130\|A0A093C130_9AVES Inter-alpha-trypsin inhibitor heavy chain H3 (Fragment) OS=Pterocles gutturalis GN=N339_10952 PE=4 SV=1 | 30486626,7 | 19 | 7 |
| 191 | \|Q5ZKJ2\|Q5ZKJ2_CHICK Tyrosine 3-monooxygenase/tryptophan 5-monooxygenase activation protein eta OS=Gallus gallus GN=YWHAH PE=2 SV=1 | 30022613,9 | 13 | 7 |
| 192 | \|A0A091K9Q0\|A0A091K9Q0_COLST Lysozyme g (Fragment) OS=Colius striatus GN=N325_03722 PE=4 SV=1 | 29635582,2 | 11 | 2 |
| 193 | \|A0A093PXV8\|A0A093PXV8_9PASS Ig lambda chain V-1 region (Fragment) OS=Manacus vitellinus GN=N305_06014 PE=4 SV=1 | 29450908,2 | 18 | 2 |
| 194 | \|A0A093EU69\|A0A093EU69_GAVST Complement component C7 (Fragment) OS=Gavia stellata GN=N328_07373 PE=4 SV=1 | 28853456,7 | 17 | 10 |
| 195 | \|A0A091Q1P7\|A0A091Q1P7_LEPDC Phosphoglycerate kinase (Fragment) OS=Leptosomus discolor GN=N330_06447 PE=3 SV=1 | 28478300,6 | 23 | 10 |
| 196 | \|Q5ZKN7\|Q5ZKN7_CHICK Ubiquitin conjugating enzyme E2 L5, pseudogene OS=Gallus gallus GN=UBE2L3 PE=2 SV=1 | 27781159,9 | 5 | 2 |
| 197 | \|A0A091HCY2\|A0A091HCY2_BUCRH Ovoinhibitor (Fragment) OS=Buceros rhinoceros silvestris GN=N320_07235 PE=4 SV=1 | 27177373,6 | 5 | 1 |
| 198 | \|A0A091FIK1\|A0A091FIK1_9AVES Apolipoprotein A-I (Fragment) OS=Cuculus canorus GN=N303_12975 PE=3 SV=1 | 27062447,3 | 10 | 1 |
| 199 | \|A0A091UJZ1\|A0A091UJZ1_PHALP Cystatin (Fragment) OS=Phaethon lepturus GN=N335_10834 PE=3 SV=1 | 27049633,8 | 10 | 3 |
| 200 | \|R7VNZ2\|R7VNZ2_COLLI Fructose-bisphosphate aldolase A OS=Columba livia GN=A306_12840 PE=4 SV=1 | 26350263,6 | 8 | 2 |
| 201 | \|A0A091G7N9\|A0A091G7N9_9AVES Alpha-2-macroglobulin (Fragment) OS=Cuculus canorus GN=N303_01487 PE=4 SV=1 | 26128259,2 | 15 | 3 |
| 202 | \|Q90694\|CDC42_CHICK Cell division control protein 42 homolog OS=Gallus gallus GN=CDC42 PE=2 SV=1 | 25988529,1 | 9 | 7 |
| 203 | \|Q5ZLJ7\|Q5ZLJ7_CHICK Tropomyosin 3 OS=Gallus gallus GN=TPM3 PE=2 SV=1 | 25568996,1 | 17 | 11 |
| 204 | \|A0A091P6E7\|A0A091P6E7_LEPDC Inter-alpha-trypsin inhibitor heavy chain H2 (Fragment) OS=Leptosomus discolor GN=N330_01477 PE=4 SV=1 | 25395086,8 | 13 | 4 |
| 205 | \|A0A093PB96\|A0A093PB96_PYGAD Villin-1 (Fragment) OS=Pygoscelis adeliae GN=AS28_14715 PE=4 SV=1 | 25321203,6 | 14 | 11 |
| 206 | \|A0A091U2N0\|A0A091U2N0_PHORB Ovostatin (Fragment) OS=Phoenicopterus ruber ruber GN=N337_04551 PE=4 SV=1 | 25074162,5 | 5 | 3 |
| 207 | \|P53488\|ARP2_CHICK Actin-related protein 2 OS=Gallus gallus GN=ACTR2 PE=2 SV=1 | 25018532,6 | 18 | 10 |
| 208 | \|A0A093HP97\|A0A093HP97_STRCA Alcohol dehydrogenase [NADP(+)] (Fragment) OS=Struthio camelus australis GN=N308_02878 PE=4 SV=1 | 24965942,8 | 14 | 8 |
| 209 | \|A0A091GN73\|A0A091GN73_9AVES Deleted in malignant brain tumors 1 protein (Fragment) OS=Cuculus canorus GN=N303_11657 PE=4 SV=1 | 24655023,9 | 7 | 3 |
| 210 | \|A0A093EBM6\|A0A093EBM6_TAUER Inter-alpha-trypsin inhibitor heavy chain H2 (Fragment) OS=Tauraco erythrolophus GN=N340_04479 PE=4 SV=1 | 24461292,1 | 6 | 1 |
| 211 | \|R7VU42\|R7VU42_COLLI Ig gamma chain C region (Fragment) OS=Columba livia GN=A306_11075 PE=4 SV=1 | 24314070,0 | 9 | 2 |
| 212 | \|A0A091TAV5\|A0A091TAV5_PHALP Leukotriene A(4) hydrolase (Fragment) OS=Phaethon lepturus GN=N335_03156 PE=3 SV=1 | 24094771,9 | 24 | 10 |
| 213 | \|B5FZ67\|B5FZ67_TAEGU Actin-related protein 2/3 complex subunit 3 OS=Taeniopygia guttata PE=2 SV=1 | 24045055,7 | 7 | 5 |
| 214 | \|Q9PTR5\|LIS1_CHICK Lissencephaly-1 homolog OS=Gallus gallus GN=PAFAH1B1 PE=2 SV=3 | 23820463,0 | 8 | 4 |
| 215 | \|A0A0Q3X6U4\|A0A0Q3X6U4_AMAAE Glutathione S-transferase omega-1 isoform X1 OS=Amazona aestiva GN=AAES_20343 PE=4 SV=1 | 23774109,3 | 13 | 4 |
| 216 | \|A0A0Q3U0C5\|A0A0Q3U0C5_AMAAE Alpha-tectorin-like protein OS=Amazona aestiva GN=AAES_34862 PE=4 SV=1 | 23663449,1 | 5 | 2 |
| 217 | \|U3JSQ8\|U3JSQ8_FICAL Apolipoprotein A4 OS=Ficedula albicollis GN=APOA4 PE=3 SV=1 | 23562023,3 | 8 | 2 |
| 218 | \|A0A091U600\|A0A091U600_PHALP Tetranectin (Fragment) OS=Phaethon lepturus GN=N335_00596 PE=4 SV=1 | 22902841,7 | 13 | 6 |
| 219 | \|A0A093Q5Q8\|A0A093Q5Q8_9PASS Annexin OS=Manacus vitellinus GN=N305_08261 PE=3 SV=1 | 22438450,6 | 10 | 7 |
| 220 | \|A0A093PI65\|A0A093PI65_PYGAD Ig lambda chain V-1 region (Fragment) OS=Pygoscelis adeliae GN=AS28_08482 PE=4 SV=1 | 21887208,4 | 5 | 1 |
| 221 | \|B5G462\|B5G462_TAEGU Putative actin-capping protein Z beta subunit variant 1 OS=Taeniopygia guttata PE=2 SV=1 | 21845886,9 | 15 | 9 |
| 222 | \|U5SAB8\|U5SAB8_FALPE Beta-actin (Fragment) OS=Falco peregrinus PE=2 SV=1 | 21802974,3 | 7 | 2 |
| 223 | \|U3JJZ3\|U3JJZ3_FICAL Serpin family C member 1 OS=Ficedula albicollis GN=SERPINC1 PE=3 SV=1 | 21731384,4 | 9 | 3 |
| 224 | \|U3KED6\|U3KED6_FICAL Galectin OS=Ficedula albicollis GN=LGALS1 PE=4 SV=1 | 21705774,6 | 6 | 3 |
| 225 | \|B5G0T6\|B5G0T6_TAEGU Putative thymosin beta 4 OS=Taeniopygia guttata GN=TMSB4X PE=2 SV=1 | 21447351,1 | 6 | 2 |
| 226 | \|A0A091WD78\|A0A091WD78_NIPNI UTP--glucose-1-phosphate uridylyltransferase (Fragment) OS=Nipponia nippon GN=Y956_07295 PE=4 SV=1 | 21359075,9 | 17 | 8 |
| 227 | \|A0A091USN0\|A0A091USN0_NIPNI Quinone oxidoreductase (Fragment) OS=Nipponia nippon GN=Y956_08769 PE=4 SV=1 | 21353747,5 | 10 | 5 |
| 228 | \|O93601\|O93601_CHICK Apolipoprotein A4 OS=Gallus gallus GN=apoAIV PE=2 SV=1 | 21090105,6 | 6 | 2 |
| 229 | \|A0A0N7J026\|A0A0N7J026_9TURD Beta-globin subunit A (Fragment) OS=Myadestes ralloides GN=HBBA PE=3 SV=1 | 21075751,0 | 4 | 1 |
| 230 | \|A0A093HYC5\|A0A093HYC5_STRCA Glutathione S-transferase theta-1 (Fragment) OS=Struthio camelus australis GN=N308_10009 PE=3 SV=1 | 20757782,0 | 14 | 6 |
| 231 | \|A0A091GI80\|A0A091GI80_9AVES Plasminogen (Fragment) OS=Cuculus canorus GN=N303_01142 PE=3 SV=1 | 19639676,1 | 9 | 5 |
| 232 | \|A0A0A0A3E5\|A0A0A0A3E5_CHAVO Protein-glutamine gamma-glutamyltransferase 4 (Fragment) OS=Charadrius vociferus GN=N301_11957 PE=4 SV=1 | 19342275,9 | 5 | 2 |
| 233 | \|F1NUQ3\|F1NUQ3_CHICK Fatty acid binding protein 3 OS=Gallus gallus GN=FABP3 PE=2 SV=2 | 18938875,4 | 14 | 3 |
| 234 | \|A0A099Z1C2\|A0A099Z1C2_TINGU GRIP and coiled-coil domain-containing protein 2 (Fragment) OS=Tinamus guttatus GN=N309_09442 PE=4 SV=1 | 18776104,7 | 7 | 3 |
| 235 | \|P19140\|ENOA_ANAPL Alpha-enolase OS=Anas platyrhynchos GN=ENO1 PE=2 SV=2 | 18718724,8 | 8 | 3 |
| 236 | \|A0A093I7Q3\|A0A093I7Q3_STRCA SH3 domain-binding glutamic acid-rich-like (Fragment) OS=Struthio camelus australis GN=N308_09394 PE=4 SV=1 | 18509557,0 | 11 | 5 |
| 237 | \|A0A091V329\|A0A091V329_NIPNI Plastin-3 (Fragment) OS=Nipponia nippon GN=Y956_03634 PE=4 SV=1 | 18462718,0 | 15 | 9 |
| 238 | \|E2RUJ8\|E2RUJ8_DRONO Ovotransferrin OS=Dromaius novaehollandiae PE=2 SV=1 | 18290530,2 | 12 | 1 |
| 239 | \|A0A093R918\|A0A093R918_PYGAD Protein Niban (Fragment) OS=Pygoscelis adeliae GN=AS28_01877 PE=4 SV=1 | 18233205,9 | 3 | 2 |
| 240 | \|A0A091V235\|A0A091V235_NIPNI Aconitate hydratase OS=Nipponia nippon GN=Y956_07994 PE=3 SV=1 | 17971422,5 | 10 | 9 |
| 241 | \|A0A091VJX8\|A0A091VJX8_NIPNI Ribonuclease inhibitor OS=Nipponia nippon GN=Y956_06908 PE=4 SV=1 | 17366500,0 | 12 | 6 |
| 242 | \|A0A093NWJ3\|A0A093NWJ3_PYGAD Adseverin (Fragment) OS=Pygoscelis adeliae GN=AS28_02819 PE=4 SV=1 | 17337793,3 | 8 | 5 |
| 243 | \|O43026\|G3P2_SCHPO Glyceraldehyde-3-phosphate dehydrogenase 2 OS=Schizosaccharomyces pombe (strain 972 / ATCC 24843) GN=gpd3 PE=1 SV=1 | 17172992,5 | 3 | 1 |
| 244 | \|Q9PRL8\|ACBP_CHICK Acyl-CoA-binding protein OS=Gallus gallus GN=DBI PE=1 SV=1 | 17127169,6 | 11 | 4 |
| 245 | \|Q5F3W6\|1433G_CHICK 14-3-3 protein gamma OS=Gallus gallus GN=YWHAG PE=1 SV=1 | 17053152,4 | 4 | 3 |
| 246 | \|A0A091VE17\|A0A091VE17_NIPNI Complement component C6 (Fragment) OS=Nipponia nippon GN=Y956_04447 PE=4 SV=1 | 16807115,5 | 10 | 6 |
| 247 | \|A0A091V2M9\|A0A091V2M9_NIPNI Aminopeptidase (Fragment) OS=Nipponia nippon GN=Y956_11188 PE=3 SV=1 | 16253311,3 | 21 | 11 |
| 248 | \|A0A091PUH0\|A0A091PUH0_LEPDC Kininogen-1 (Fragment) OS=Leptosomus discolor GN=N330_05468 PE=4 SV=1 | 16253001,1 | 12 | 3 |
| 249 | \|A0A0Q3MG80\|A0A0Q3MG80_AMAAE Insulin-like growth factor-binding protein 7 isoform X1 OS=Amazona aestiva GN=AAES_83777 PE=4 SV=1 | 16242086,5 | 2 | 1 |
| 250 | \|I6U3W4\|I6U3W4_STRCA Immunonoglobulin heavy chain variable region (Fragment) OS=Struthio camelus PE=2 SV=1 | 16200635,1 | 7 | 2 |
| 251 | \|A7LGI9\|A7LGI9_ELAFO Alpha-enolase (Fragment) OS=Elanoides forficatus PE=4 SV=1 | 15784300,3 | 8 | 2 |
| 252 | \|A0A091VDP5\|A0A091VDP5_NIPNI Thioredoxin domain-containing protein 17 (Fragment) OS=Nipponia nippon GN=Y956_03267 PE=4 SV=1 | 15689752,3 | 4 | 2 |
| 253 | \|A0A0Q3M2E3\|A0A0Q3M2E3_AMAAE Cullin-associated NEDD8-dissociated protein 1 isoform X1 OS=Amazona aestiva GN=AAES_133092 PE=4 SV=1 | 15388766,3 | 5 | 3 |
| 254 | \|A0A091UMC3\|A0A091UMC3_NIPNI Proteasome subunit alpha type (Fragment) OS=Nipponia nippon GN=Y956_06783 PE=3 SV=1 | 15187695,1 | 4 | 3 |
| 255 | \|A0A091H4Q2\|A0A091H4Q2_9AVES Glutathione S-transferase OS=Cuculus canorus GN=N303_04781 PE=4 SV=1 | 15142476,1 | 5 | 3 |
| 256 | \|A0A091KBY8\|A0A091KBY8_COLST Fructose-1,6-bisphosphatase 1 OS=Colius striatus GN=N325_05687 PE=3 SV=1 | 14830321,8 | 14 | 9 |
| 257 | \|B5G418\|B5G418_TAEGU Arp2/3 complex 34 kDa subunit OS=Taeniopygia guttata PE=2 SV=1 | 14671870,1 | 8 | 6 |
| 258 | \|A0A093P651\|A0A093P651_PYGAD Ig lambda chain V-1 region (Fragment) OS=Pygoscelis adeliae GN=AS28_08481 PE=4 SV=1 | 14647805,3 | 4 | 1 |
| 259 | \|A0A091PHV6\|A0A091PHV6_LEPDC Alpha-1-antichymotrypsin (Fragment) OS=Leptosomus discolor GN=N330_03589 PE=3 SV=1 | 14624014,8 | 9 | 3 |
| 260 | \|A0A091UU42\|A0A091UU42_NIPNI Phosphoglucomutase-2 (Fragment) OS=Nipponia nippon GN=Y956_08293 PE=3 SV=1 | 14472364,9 | 8 | 6 |
| 261 | \|A0A091I304\|A0A091I304_CALAN Transgelin OS=Calypte anna GN=N300_14274 PE=3 SV=1 | 14036879,4 | 7 | 6 |
| 262 | \|A0A091UPC5\|A0A091UPC5_NIPNI Glutathione S-transferase omega-1 (Fragment) OS=Nipponia nippon GN=Y956_01340 PE=4 SV=1 | 13944290,6 | 8 | 5 |
| 263 | \|A0A093NFF9\|A0A093NFF9_PYGAD Ester hydrolase C11orf54 (Fragment) OS=Pygoscelis adeliae GN=AS28_02579 PE=4 SV=1 | 13732389,0 | 19 | 8 |
| 264 | \|A0A093PIR8\|A0A093PIR8_9PASS Sulfhydryl oxidase (Fragment) OS=Manacus vitellinus GN=N305_07618 PE=4 SV=1 | 13647590,9 | 10 | 4 |
| 265 | \|A0A0Q3UPW9\|A0A0Q3UPW9_AMAAE Proactivator polypeptide-like protein OS=Amazona aestiva GN=AAES_166919 PE=4 SV=1 | 13376285,7 | 6 | 6 |
| 266 | \|A0A091GFP0\|A0A091GFP0_9AVES Ceruloplasmin (Fragment) OS=Cuculus canorus GN=N303_11031 PE=4 SV=1 | 13303208,9 | 3 | 1 |
| 267 | \|A0A091V5H1\|A0A091V5H1_NIPNI Glutathione peroxidase (Fragment) OS=Nipponia nippon GN=Y956_02357 PE=3 SV=1 | 12982769,3 | 12 | 5 |
| 268 | \|A0A091VH59\|A0A091VH59_NIPNI Proteasome subunit alpha type (Fragment) OS=Nipponia nippon GN=Y956_05506 PE=3 SV=1 | 12982666,6 | 6 | 3 |
| 269 | \|A0A091SET2\|A0A091SET2_NESNO Cystatin-B (Fragment) OS=Nestor notabilis GN=N333_00630 PE=4 SV=1 | 12940802,5 | 6 | 3 |
| 270 | \|A0A0A0A3R4\|A0A0A0A3R4_CHAVO Complement component C7 (Fragment) OS=Charadrius vociferus GN=N301_10859 PE=4 SV=1 | 12595241,9 | 3 | 2 |
| 271 | \|A0A093N8C7\|A0A093N8C7_PYGAD Complement component C6 (Fragment) OS=Pygoscelis adeliae GN=AS28_07942 PE=4 SV=1 | 12276089,9 | 4 | 2 |
| 272 | \|P60706\|ACTB_CHICK Actin, cytoplasmic 1 OS=Gallus gallus GN=ACTB PE=1 SV=1 | 12119615,6 | 4 | 1 |
| 273 | \|A0A0Q3R962\|A0A0Q3R962_AMAAE Beta-2-glycoprotein 1-like protein OS=Amazona aestiva GN=AAES_77464 PE=4 SV=1 | 12100656,9 | 5 | 1 |
| 274 | \|A0A093IGD4\|A0A093IGD4_FULGA Deleted in malignant brain tumors 1 protein (Fragment) OS=Fulmarus glacialis GN=N327_09737 PE=4 SV=1 | 11884837,7 | 7 | 2 |
| 275 | \|Q9I882\|Q9I882_CHICK Protein kinase C inhibitor OS=Gallus gallus GN=chPKCI PE=2 SV=1 | 11826433,9 | 4 | 2 |
| 276 | \|A0A091U869\|A0A091U869_PHALP Keratin, type I cytoskeletal 19 (Fragment) OS=Phaethon lepturus GN=N335_04773 PE=3 SV=1 | 11562814,9 | 7 | 5 |
| 277 | \|A0A093SFU4\|A0A093SFU4_9PASS Ig lambda chain V-1 region (Fragment) OS=Manacus vitellinus GN=N305_06015 PE=4 SV=1 | 11298015,0 | 5 | 1 |
| 278 | \|A0A091URM5\|A0A091URM5_NIPNI Protein S100 (Fragment) OS=Nipponia nippon GN=Y956_04774 PE=3 SV=1 | 11240906,7 | 9 | 4 |
| 279 | \|A0A091WLT5\|A0A091WLT5_NIPNI Aminopeptidase N (Fragment) OS=Nipponia nippon GN=Y956_09625 PE=4 SV=1 | 10910771,6 | 4 | 2 |
| 280 | \|U3JQ23\|U3JQ23_FICAL Ubiquitinyl hydrolase 1 OS=Ficedula albicollis GN=USP5 PE=3 SV=1 | 10898971,4 | 4 | 2 |
| 281 | \|A0A091VKD4\|A0A091VKD4_NIPNI WD repeat-containing protein 1 (Fragment) OS=Nipponia nippon GN=Y956_04783 PE=4 SV=1 | 10638639,8 | 10 | 8 |
| 282 | \|A0A091W0P1\|A0A091W0P1_NIPNI Leukocyte elastase inhibitor OS=Nipponia nippon GN=Y956_11915 PE=3 SV=1 | 10602368,9 | 9 | 3 |
| 283 | \|A0A093IBW8\|A0A093IBW8_FULGA Complement C5 (Fragment) OS=Fulmarus glacialis GN=N327_05448 PE=4 SV=1 | 10538464,5 | 9 | 7 |
| 284 | \|U3JYI8\|U3JYI8_FICAL Uncharacterized protein OS=Ficedula albicollis GN=LOC101817694 PE=4 SV=1 | 10430747,8 | 11 | 3 |
| 285 | \|U3K7W5\|U3K7W5_FICAL Lumican OS=Ficedula albicollis GN=LUM PE=4 SV=1 | 10214610,3 | 9 | 5 |
| 286 | \|A0A099ZM43\|A0A099ZM43_TINGU Serum albumin OS=Tinamus guttatus GN=N309_11365 PE=3 SV=1 | 10086542,0 | 7 | 5 |
| 287 | \|P42558\|RAN_CHICK GTP-binding nuclear protein Ran OS=Gallus gallus GN=RAN PE=2 SV=1 | 10080448,2 | 8 | 4 |
| 288 | \|A0A091G4B1\|A0A091G4B1_9AVES Coagulation factor IX (Fragment) OS=Cuculus canorus GN=N303_10524 PE=3 SV=1 | 10072439,9 | 5 | 3 |
| 289 | \|A0A091VED5\|A0A091VED5_NIPNI Thimet oligopeptidase (Fragment) OS=Nipponia nippon GN=Y956_07015 PE=3 SV=1 | 10020798,5 | 14 | 8 |
| 290 | \|A0A099Z3C3\|A0A099Z3C3_TINGU Fructose-bisphosphate aldolase (Fragment) OS=Tinamus guttatus GN=N309_13513 PE=3 SV=1 | 9825806,2 | 5 | 1 |
| 291 | \|A0A093FCF6\|A0A093FCF6_GAVST Hematopoietic prostaglandin D synthase OS=Gavia stellata GN=N328_08786 PE=4 SV=1 | 9799153,9 | 6 | 3 |
| 292 | \|A0A0L6K307\|A0A0L6K307_9RHIZ Chaperone protein DnaK OS=Agrobacterium sp. SUL3 GN=dnaK PE=2 SV=1 | 9758417,8 | 12 | 3 |
| 293 | \|A0A091PZG2\|A0A091PZG2_LEPDC Programmed cell death 6-interacting protein (Fragment) OS=Leptosomus discolor GN=N330_12215 PE=4 SV=1 | 9510605,5 | 3 | 3 |
| 294 | \|A0A0Q3MLS8\|A0A0Q3MLS8_AMAAE Proteasome subunit alpha type OS=Amazona aestiva GN=AAES_62148 PE=3 SV=1 | 9325280,1 | 8 | 3 |
| 295 | \|Q5ZM35\|TWF2_CHICK Twinfilin-2 OS=Gallus gallus GN=TWF2 PE=1 SV=1 | 9304106,4 | 2 | 1 |
| 296 | \|A0A091SLR3\|A0A091SLR3_NESNO Vitronectin (Fragment) OS=Nestor notabilis GN=N333_04118 PE=4 SV=1 | 9300661,1 | 3 | 1 |
| 297 | \|A0A093FPK1\|A0A093FPK1_GAVST Mesothelin (Fragment) OS=Gavia stellata GN=N328_12082 PE=4 SV=1 | 9003122,7 | 10 | 3 |
| 298 | \|A0A091J8Z6\|A0A091J8Z6_9AVES Ig heavy chain V-III region VH26 (Fragment) OS=Egretta garzetta GN=Z169_01257 PE=4 SV=1 | 8835071,7 | 7 | 2 |
| 299 | \|H9CTT2\|H9CTT2_COLLI Peroxiredoxin 6 OS=Columba livia GN=PRDX6 PE=2 SV=1 | 8831393,3 | 8 | 3 |
| 300 | \|A0A0Q3MG37\|A0A0Q3MG37_AMAAE Multiple inositol polyphosphate phosphatase 1 OS=Amazona aestiva GN=AAES_80077 PE=3 SV=1 | 8647826,0 | 3 | 2 |
| 301 | \|A0A093PG38\|A0A093PG38_9PASS Ig lambda chain V-1 region (Fragment) OS=Manacus vitellinus GN=N305_01667 PE=4 SV=1 | 8589976,1 | 5 | 1 |
| 302 | \|P79781\|RS27A_CHICK Ubiquitin-40S ribosomal protein S27a OS=Gallus gallus GN=RPS27A PE=2 SV=3 | 8539964,7 | 4 | 2 |
| 303 | \|A0A093CBC5\|A0A093CBC5_9AVES Inorganic pyrophosphatase (Fragment) OS=Pterocles gutturalis GN=N339_10872 PE=4 SV=1 | 8458506,6 | 10 | 6 |
| 304 | \|A0A091ML87\|A0A091ML87_9PASS Ig lambda chain V-1 region (Fragment) OS=Acanthisitta chloris GN=N310_10505 PE=4 SV=1 | 8285803,3 | 5 | 1 |
| 305 | \|A0A091UIM1\|A0A091UIM1_PHORB Ovocalyxin-32 (Fragment) OS=Phoenicopterus ruber ruber GN=N337_08580 PE=4 SV=1 | 8271827,5 | 4 | 4 |
| 306 | \|A0A093R3B0\|A0A093R3B0_PYGAD Fetuin-B (Fragment) OS=Pygoscelis adeliae GN=AS28_05393 PE=4 SV=1 | 8263204,2 | 3 | 1 |
| 307 | \|B5FYC6\|B5FYC6_TAEGU Putative inhibitor-1 of protein phosphatase type 2A variant 2 OS=Taeniopygia guttata GN=ANP32A PE=2 SV=1 | 7978304,4 | 8 | 5 |
| 308 | \|A0A091VWZ6\|A0A091VWZ6_NIPNI Aldose reductase (Fragment) OS=Nipponia nippon GN=Y956_04891 PE=4 SV=1 | 7923194,6 | 3 | 1 |
| 309 | \|Q92007\|Q92007_CHICK Aldolase A (Fragment) OS=Gallus gallus GN=aldolase C PE=2 SV=1 | 7855861,1 | 3 | 1 |
| 310 | \|O42395\|CNBP_CHICK Cellular nucleic acid-binding protein OS=Gallus gallus GN=CNBP PE=2 SV=1 | 7586541,7 | 1 | 1 |
| 311 | \|A0A091TPZ4\|A0A091TPZ4_PHALP Lambda-crystallin (Fragment) OS=Phaethon lepturus GN=N335_13508 PE=4 SV=1 | 7109380,2 | 3 | 2 |
| 312 | \|A0A091HEM5\|A0A091HEM5_BUCRH Ovostatin (Fragment) OS=Buceros rhinoceros silvestris GN=N320_07265 PE=4 SV=1 | 7107739,7 | 3 | 1 |
| 313 | \|A0A091PL52\|A0A091PL52_LEPDC Extracellular superoxide dismutase [Cu-Zn] (Fragment) OS=Leptosomus discolor GN=N330_09036 PE=4 SV=1 | 6747764,3 | 4 | 3 |
| 314 | \|A0A093FPZ6\|A0A093FPZ6_GAVST Kininogen-1 (Fragment) OS=Gavia stellata GN=N328_02740 PE=4 SV=1 | 6727344,5 | 6 | 2 |
| 315 | \|A0A0Q3WVG4\|A0A0Q3WVG4_AMAAE Uncharacterized protein OS=Amazona aestiva GN=AAES_32336 PE=3 SV=1 | 6629684,1 | 6 | 3 |
| 316 | \|A0A091UMT3\|A0A091UMT3_NIPNI Aspartate aminotransferase (Fragment) OS=Nipponia nippon GN=Y956_01462 PE=4 SV=1 | 6551196,4 | 9 | 5 |
| 317 | \|U3K9V5\|U3K9V5_FICAL Dihydropyrimidinase like 2 OS=Ficedula albicollis GN=DPYSL2 PE=4 SV=1 | 6262496,7 | 6 | 5 |
| 318 | \|A0A091I9F8\|A0A091I9F8_CALAN NSFL1 cofactor p47 (Fragment) OS=Calypte anna GN=N300_15656 PE=4 SV=1 | 6223783,3 | 7 | 3 |
| 319 | \|A0A091I962\|A0A091I962_CALAN Aminopeptidase B (Fragment) OS=Calypte anna GN=N300_03709 PE=4 SV=1 | 6219913,1 | 4 | 3 |
| 320 | \|A0A0Q3X9Y9\|A0A0Q3X9Y9_AMAAE SET translocation OS=Amazona aestiva GN=AAES_09421 PE=3 SV=1 | 6201423,5 | 5 | 4 |
| 321 | \|A0A091UZE6\|A0A091UZE6_NIPNI ADP-ribosyl cyclase 2 (Fragment) OS=Nipponia nippon GN=Y956_10569 PE=4 SV=1 | 6127585,9 | 3 | 1 |
| 322 | \|A0A091UN46\|A0A091UN46_NIPNI LIM and SH3 domain protein 1 (Fragment) OS=Nipponia nippon GN=Y956_16168 PE=4 SV=1 | 6115742,8 | 3 | 2 |
| 323 | \|A0A0Q3PZX3\|A0A0Q3PZX3_AMAAE Uncharacterized protein OS=Amazona aestiva GN=AAES_83371 PE=4 SV=1 | 5989414,9 | 5 | 4 |
| 324 | \|R7VV38\|R7VV38_COLLI Serine/threonine-protein phosphatase 2A 65 kDa regulatory subunit A alpha isoform (Fragment) OS=Columba livia GN=A306_02287 PE=4 SV=1 | 5980938,1 | 9 | 5 |
| 325 | \|A0A091W370\|A0A091W370_NIPNI Serpin B6 OS=Nipponia nippon GN=Y956_11916 PE=3 SV=1 | 5860816,1 | 5 | 3 |
| 326 | \|A0A091UYE9\|A0A091UYE9_NIPNI S-formylglutathione hydrolase OS=Nipponia nippon GN=Y956_16125 PE=3 SV=1 | 5850547,3 | 3 | 2 |
| 327 | \|A0A093GTH7\|A0A093GTH7_PICPB Glutamate--cysteine ligase catalytic subunit (Fragment) OS=Picoides pubescens GN=N307_11714 PE=4 SV=1 | 5697282,5 | 12 | 5 |
| 328 | \|P12902\|HM14A_CHICK Non-histone chromosomal protein HMG-14A OS=Gallus gallus PE=3 SV=2 | 5687900,0 | 3 | 1 |
| 329 | \|A0A1I9XG94\|A0A1I9XG94_TYTAL Furin variant 2 OS=Tyto alba GN=FURIN PE=2 SV=1 | 5652504,9 | 12 | 7 |
| 330 | \|L1K2I6\|L1K2I6_GUITH Translation elongation factor 1-alpha OS=Guillardia theta CCMP2712 GN=sEF1A-1 PE=3 SV=1 | 5620073,9 | 2 | 2 |
| 331 | \|A0A093FKZ6\|A0A093FKZ6_GAVST Complement C4 (Fragment) OS=Gavia stellata GN=N328_02950 PE=4 SV=1 | 5567651,1 | 8 | 7 |
| 332 | \|P08070\|TBA2_CHICK Tubulin alpha-2 chain OS=Gallus gallus PE=2 SV=1 | 5561532,8 | 5 | 3 |
| 333 | \|A0A093HVZ9\|A0A093HVZ9_STRCA Malate dehydrogenase (Fragment) OS=Struthio camelus australis GN=N308_16042 PE=3 SV=1 | 5514595,7 | 7 | 4 |
| 334 | \|A0A091IFD9\|A0A091IFD9_CALAN Serine/threonine-protein kinase OSR1 (Fragment) OS=Calypte anna GN=N300_12058 PE=4 SV=1 | 5465736,3 | 4 | 3 |
| 335 | \|A0A091TAK8\|A0A091TAK8_PHALP Plasminogen (Fragment) OS=Phaethon lepturus GN=N335_06458 PE=3 SV=1 | 5450674,0 | 2 | 1 |
| 336 | \|A0A091HP34\|A0A091HP34_CALAN Aldo-keto reductase family 1 member B10 OS=Calypte anna GN=N300_06635 PE=4 SV=1 | 5401002,7 | 7 | 2 |
| 337 | \|A0A0A0AWG1\|A0A0A0AWG1_CHAVO Elongation factor 1-beta OS=Charadrius vociferus GN=N301_02493 PE=3 SV=1 | 5335758,0 | 2 | 2 |
| 338 | \|A0A091N0B3\|A0A091N0B3_9PASS L-lactate dehydrogenase (Fragment) OS=Acanthisitta chloris GN=N310_01156 PE=3 SV=1 | 5310514,8 | 2 | 2 |
| 339 | \|A0A093PFK5\|A0A093PFK5_9PASS Uncharacterized protein OS=Manacus vitellinus GN=N305_00489 PE=4 SV=1 | 5250760,1 | 3 | 3 |
| 340 | \|A0A0Q3S4E3\|A0A0Q3S4E3_AMAAE Long-chain fatty acid transport protein 1 OS=Amazona aestiva GN=AAES_34655 PE=4 SV=1 | 5193164,3 | 4 | 1 |
| 341 | \|B5FYD1\|B5FYD1_TAEGU Putative macrophage migration inhibitory factor (MIF) variant 1 OS=Taeniopygia guttata PE=2 SV=1 | 5024571,4 | 5 | 3 |
| 342 | \|A0A0Q3UTW8\|A0A0Q3UTW8_AMAAE Inter-alpha-trypsin inhibitor heavy chain H3 OS=Amazona aestiva GN=AAES_48564 PE=4 SV=1 | 4997731,3 | 4 | 1 |
| 343 | \|Q5ZJX9\|Q5ZJX9_CHICK Proteasome subunit alpha type OS=Gallus gallus GN=PSMA5 PE=2 SV=1 | 4938791,6 | 8 | 6 |
| 344 | \|A0A0Q3WRC2\|A0A0Q3WRC2_AMAAE Uncharacterized protein OS=Amazona aestiva GN=AAES_27573 PE=4 SV=1 | 4892039,6 | 2 | 1 |
| 345 | \|U3JGK3\|U3JGK3_FICAL Inter-alpha-trypsin inhibitor heavy chain 2 OS=Ficedula albicollis GN=ITIH2 PE=4 SV=1 | 4882440,9 | 3 | 2 |
| 346 | \|P43347\|TCTP_CHICK Translationally-controlled tumor protein homolog OS=Gallus gallus GN=TPT1 PE=2 SV=1 | 4718555,8 | 5 | 3 |
| 347 | \|Q90955\|TEBP_CHICK Prostaglandin E synthase 3 (Fragment) OS=Gallus gallus GN=PTGES3 PE=1 SV=1 | 4608487,5 | 5 | 3 |
| 348 | \|A0A091V1M9\|A0A091V1M9_NIPNI Alpha-2-macroglobulin-like 1 (Fragment) OS=Nipponia nippon GN=Y956_11260 PE=4 SV=1 | 4598162,7 | 8 | 2 |
| 349 | \|A0A093GN98\|A0A093GN98_PICPB Angiotensinogen (Fragment) OS=Picoides pubescens GN=N307_04262 PE=3 SV=1 | 4567068,3 | 4 | 2 |
| 350 | \|A0A093GIB9\|A0A093GIB9_PICPB Lambda-crystallin (Fragment) OS=Picoides pubescens GN=N307_06464 PE=4 SV=1 | 4450114,5 | 2 | 2 |
| 351 | \|A0A091TN46\|A0A091TN46_PHALP Fructose-1,6-bisphosphatase 1 (Fragment) OS=Phaethon lepturus GN=N335_03445 PE=3 SV=1 | 4417146,3 | 2 | 1 |
| 352 | \|A0A091IE09\|A0A091IE09_CALAN SH3 domain-binding glutamic acid-rich-like protein OS=Calypte anna GN=N300_05838 PE=3 SV=1 | 4395388,4 | 2 | 2 |
| 353 | \|A0A091V374\|A0A091V374_NIPNI F-actin-capping protein subunit alpha-2 (Fragment) OS=Nipponia nippon GN=Y956_02871 PE=3 SV=1 | 4387184,1 | 7 | 5 |
| 354 | \|A0A093FHB0\|A0A093FHB0_GAVST Dynein light chain 2, cytoplasmic (Fragment) OS=Gavia stellata GN=N328_12187 PE=4 SV=1 | 4332084,7 | 2 | 2 |
| 355 | \|A0A091IZH7\|A0A091IZH7_CALAN Triosephosphate isomerase OS=Calypte anna GN=N300_14133 PE=3 SV=1 | 4268160,4 | 1 | 1 |
| 356 | \|A0A091WXQ7\|A0A091WXQ7_NIPNI 3'(2'),5'-bisphosphate nucleotidase 1 (Fragment) OS=Nipponia nippon GN=Y956_07710 PE=4 SV=1 | 4264870,6 | 4 | 3 |
| 357 | \|A0A093FCT0\|A0A093FCT0_GAVST 4-trimethylaminobutyraldehyde dehydrogenase (Fragment) OS=Gavia stellata GN=N328_01327 PE=3 SV=1 | 4256699,5 | 3 | 2 |
| 358 | \|Q5ZJM9\|SUMO2_CHICK Small ubiquitin-related modifier 2 OS=Gallus gallus GN=SUMO2 PE=3 SV=1 | 4217192,3 | 3 | 2 |
| 359 | \|A0A091J320\|A0A091J320_9AVES Alpha-aminoadipic semialdehyde dehydrogenase (Fragment) OS=Egretta garzetta GN=Z169_11435 PE=3 SV=1 | 4187208,1 | 4 | 4 |
| 360 | \|A0A093PM99\|A0A093PM99_9PASS Ig lambda chain V-1 region (Fragment) OS=Manacus vitellinus GN=N305_01665 PE=4 SV=1 | 4170795,9 | 2 | 1 |
| 361 | \|A0A093H3L8\|A0A093H3L8_STRCA Proteasome subunit beta type (Fragment) OS=Struthio camelus australis GN=N308_14110 PE=3 SV=1 | 4154369,3 | 3 | 3 |
| 362 | \|A0A093BQP4\|A0A093BQP4_CHAPE Myeloperoxidase (Fragment) OS=Chaetura pelagica GN=M959_11292 PE=4 SV=1 | 4110187,2 | 6 | 2 |
| 363 | \|P41887\|HSP90_SCHPO Heat shock protein 90 homolog OS=Schizosaccharomyces pombe (strain 972 / ATCC 24843) GN=swo1 PE=1 SV=2 | 4107881,4 | 8 | 2 |
| 364 | \|A0A093P0B5\|A0A093P0B5_PYGAD Superoxide dismutase [Cu-Zn] (Fragment) OS=Pygoscelis adeliae GN=AS28_02897 PE=4 SV=1 | 3800532,8 | 5 | 3 |
| 365 | \|A0A091UMI3\|A0A091UMI3_NIPNI Serine/threonine-protein phosphatase (Fragment) OS=Nipponia nippon GN=Y956_07238 PE=3 SV=1 | 3743865,7 | 4 | 4 |
| 366 | \|Q5ZHZ0\|DX39B_CHICK Spliceosome RNA helicase DDX39B OS=Gallus gallus GN=DDX39B PE=2 SV=1 | 3742749,3 | 5 | 5 |
| 367 | \|A0A091UY06\|A0A091UY06_NIPNI Vitamin D-binding protein (Fragment) OS=Nipponia nippon GN=Y956_01828 PE=3 SV=1 | 3702181,7 | 6 | 2 |
| 368 | \|A0A091TUK9\|A0A091TUK9_PHORB Acetyl-CoA acetyltransferase, cytosolic (Fragment) OS=Phoenicopterus ruber ruber GN=N337_04274 PE=3 SV=1 | 3484235,0 | 1 | 1 |
| 369 | \|P08284\|H101_CHICK Histone H1.01 OS=Gallus gallus PE=1 SV=2 | 3442523,3 | 1 | 1 |
| 370 | \|A0A0Q3LXU3\|A0A0Q3LXU3_AMAAE Uncharacterized protein OS=Amazona aestiva GN=AAES_145653 PE=4 SV=1 | 3425145,5 | 3 | 2 |
| 371 | \|I6YNU4\|I6YNU4_CROMA Complement component 3d (Fragment) OS=Crossoptilon mantchuricum PE=4 SV=1 | 3293493,4 | 6 | 2 |
| 372 | \|P16527\|MARCS_CHICK Myristoylated alanine-rich C-kinase substrate OS=Gallus gallus GN=MARCKS PE=1 SV=2 | 3278414,7 | 3 | 3 |
| 373 | \|A0A091T801\|A0A091T801_PHALP Fructose-bisphosphate aldolase OS=Phaethon lepturus GN=N335_07156 PE=3 SV=1 | 3187149,6 | 6 | 4 |
| 374 | \|A0A093I0S8\|A0A093I0S8_STRCA Ovostatin (Fragment) OS=Struthio camelus australis GN=N308_05415 PE=4 SV=1 | 3050700,8 | 4 | 2 |
| 375 | \|P05122\|KCRB_CHICK Creatine kinase B-type OS=Gallus gallus GN=CKB PE=1 SV=1 | 3027366,1 | 8 | 5 |
| 376 | \|A0A091TLF3\|A0A091TLF3_PHALP Uncharacterized protein (Fragment) OS=Phaethon lepturus GN=N335_05435 PE=4 SV=1 | 2980549,6 | 4 | 1 |
| 377 | \|A0A093EAK1\|A0A093EAK1_9AVES Phosphoglycerate kinase (Fragment) OS=Pterocles gutturalis GN=N339_04514 PE=3 SV=1 | 2804123,5 | 6 | 2 |
| 378 | \|A0A091TNQ5\|A0A091TNQ5_PHALP Complement component C9 (Fragment) OS=Phaethon lepturus GN=N335_03407 PE=4 SV=1 | 2719095,5 | 5 | 3 |
| 379 | \|A0A099ZAG1\|A0A099ZAG1_TINGU Ceruloplasmin (Fragment) OS=Tinamus guttatus GN=N309_02557 PE=4 SV=1 | 2711265,1 | 6 | 2 |
| 380 | \|A0A091WR23\|A0A091WR23_NIPNI Ubiquitin thioesterase OTUB1 (Fragment) OS=Nipponia nippon GN=Y956_10291 PE=4 SV=1 | 2663384,3 | 3 | 1 |
| 381 | \|A0A099ZSL1\|A0A099ZSL1_TINGU Superoxide dismutase [Cu-Zn] (Fragment) OS=Tinamus guttatus GN=N309_04416 PE=4 SV=1 | 2642006,6 | 3 | 1 |
| 382 | \|A0A182C605\|A0A182C605_CHICK Z-linked thioredoxin like 1 OS=Gallus gallus gallus PE=4 SV=1 | 2632155,9 | 5 | 3 |
| 383 | \|A0A091FMX8\|A0A091FMX8_9AVES Katanin p60 ATPase-containing subunit A-like 2 OS=Cuculus canorus GN=KATNAL2 PE=3 SV=1 | 2623347,9 | 3 | 1 |
| 384 | \|A0A0A0B025\|A0A0A0B025_CHAVO Peroxiredoxin-6 (Fragment) OS=Charadrius vociferus GN=N301_09650 PE=4 SV=1 | 2585854,8 | 8 | 3 |
| 385 | \|A0A091U378\|A0A091U378_PHORB Aldo-keto reductase family 1 member B10 (Fragment) OS=Phoenicopterus ruber ruber GN=N337_04788 PE=4 SV=1 | 2423701,3 | 3 | 2 |
| 386 | \|A0A0A0AG62\|A0A0A0AG62_CHAVO Serpin B6 OS=Charadrius vociferus GN=N301_12933 PE=3 SV=1 | 2372515,5 | 4 | 2 |
| 387 | \|A0A093HIS2\|A0A093HIS2_STRCA Ras GTPase-activating-like IQGAP1 (Fragment) OS=Struthio camelus australis GN=N308_08415 PE=4 SV=1 | 2351282,7 | 8 | 7 |
| 388 | \|P09206\|TBB3_CHICK Tubulin beta-3 chain OS=Gallus gallus PE=2 SV=1 | 2348662,8 | 3 | 1 |
| 389 | \|A0A093JI04\|A0A093JI04_STRCA Actin-related protein 2/3 complex subunit 4 (Fragment) OS=Struthio camelus australis GN=N308_03808 PE=4 SV=1 | 2343397,3 | 5 | 2 |
| 390 | \|Q5ZMJ0\|Q5ZMJ0_CHICK Peptidyl-prolyl cis-trans isomerase OS=Gallus gallus GN=PPIF PE=2 SV=1 | 2275120,5 | 4 | 1 |
| 391 | \|A0A093H9X9\|A0A093H9X9_STRCA Syndecan (Fragment) OS=Struthio camelus australis GN=N308_10716 PE=3 SV=1 | 2255923,9 | 4 | 2 |
| 392 | \|A0A091PZX6\|A0A091PZX6_LEPDC Gallinacin-9 (Fragment) OS=Leptosomus discolor GN=N330_12160 PE=4 SV=1 | 2193294,3 | 4 | 1 |
| 393 | \|A0A093BMN2\|A0A093BMN2_CHAPE Tubulin alpha chain (Fragment) OS=Chaetura pelagica GN=M959_06782 PE=3 SV=1 | 2109156,7 | 5 | 1 |
| 394 | \|A0A099ZL48\|A0A099ZL48_TINGU Transketolase (Fragment) OS=Tinamus guttatus GN=N309_14056 PE=4 SV=1 | 2091202,9 | 1 | 1 |
| 395 | \|A0A099ZYW0\|A0A099ZYW0_CHAVO Uncharacterized protein (Fragment) OS=Charadrius vociferus GN=N301_13361 PE=4 SV=1 | 2071233,7 | 6 | 2 |
| 396 | \|A0A091PBL0\|A0A091PBL0_LEPDC CMP-N-acetylneuraminate-beta-galactosamide-alpha-2, 3-sialyltransferase 1 (Fragment) OS=Leptosomus discolor GN=N330_00930 PE=3 SV=1 | 1979577,4 | 1 | 1 |
| 397 | \|A0A091VWS9\|A0A091VWS9_NIPNI Carbonyl reductase [NADPH] 1 OS=Nipponia nippon GN=Y956_04405 PE=3 SV=1 | 1972091,0 | 5 | 4 |
| 398 | \|A0A093NL91\|A0A093NL91_PYGAD 14-3-3 protein sigma (Fragment) OS=Pygoscelis adeliae GN=AS28_10438 PE=3 SV=1 | 1929379,5 | 2 | 2 |
| 399 | \|B0LJS6\|B0LJS6_PASAF Beta-actin (Fragment) OS=Passerina amoena PE=4 SV=1 | 1924879,6 | 3 | 3 |
| 400 | \|S5N721\|S5N721_9PASE Complement component 3d (Fragment) OS=Melanocorypha mongolica PE=4 SV=1 | 1906319,9 | 1 | 1 |
| 401 | \|U3JXN8\|U3JXN8_FICAL 6-phosphogluconate dehydrogenase, decarboxylating OS=Ficedula albicollis GN=PGD PE=3 SV=1 | 1840089,5 | 3 | 1 |
| 402 | \|Q91955\|MTPN_CHICK Myotrophin OS=Gallus gallus GN=MTPN PE=3 SV=1 | 1840060,8 | 4 | 2 |
| 403 | \|P12274\|HMGN1_CHICK Non-histone chromosomal protein HMG-14B OS=Gallus gallus GN=HMG14 PE=3 SV=2 | 1829433,3 | 2 | 1 |
| 404 | \|A0A099ZUP1\|A0A099ZUP1_CHAVO Mesothelin (Fragment) OS=Charadrius vociferus GN=N301_13360 PE=4 SV=1 | 1826491,4 | 5 | 2 |
| 405 | \|P05094\|ACTN1_CHICK Alpha-actinin-1 OS=Gallus gallus GN=ACTN1 PE=1 SV=3 | 1775737,5 | 2 | 2 |
| 406 | \|A0A093GS00\|A0A093GS00_PICPB Alpha-1-antiproteinase 2 (Fragment) OS=Picoides pubescens GN=N307_08460 PE=3 SV=1 | 1753488,9 | 2 | 1 |
| 407 | \|P43233\|CATB_CHICK Cathepsin B OS=Gallus gallus GN=CTSB PE=2 SV=1 | 1696605,8 | 2 | 2 |
| 408 | \|A0A093HC19\|A0A093HC19_STRCA Clathrin heavy chain 1 (Fragment) OS=Struthio camelus australis GN=N308_07024 PE=4 SV=1 | 1651754,7 | 4 | 4 |
| 409 | \|A0A0Q3UT59\|A0A0Q3UT59_AMAAE Prostaglandin-H2 D-isomerase OS=Amazona aestiva GN=AAES_61196 PE=3 SV=1 | 1556414,7 | 3 | 3 |
| 410 | \|A0A099ZBC1\|A0A099ZBC1_TINGU Aldo-keto reductase family 1 member B10 OS=Tinamus guttatus GN=N309_01636 PE=4 SV=1 | 1550863,9 | 8 | 2 |
| 411 | \|Q5ZIL2\|VPS29_CHICK Vacuolar protein sorting-associated protein 29 OS=Gallus gallus GN=VPS29 PE=2 SV=1 | 1549978,0 | 1 | 1 |
| 412 | \|A0A091VZR1\|A0A091VZR1_NIPNI Galectin (Fragment) OS=Nipponia nippon GN=Y956_01193 PE=4 SV=1 | 1531327,6 | 5 | 2 |
| 413 | \|A0A091UNR0\|A0A091UNR0_NIPNI Glyoxalase domain-containing protein 4 (Fragment) OS=Nipponia nippon GN=Y956_06560 PE=4 SV=1 | 1502962,9 | 8 | 3 |
| 414 | \|A0A091FS53\|A0A091FS53_9AVES Tetranectin (Fragment) OS=Cuculus canorus GN=N303_02197 PE=4 SV=1 | 1389928,0 | 1 | 1 |
| 415 | \|A0A091PF53\|A0A091PF53_LEPDC Xaa-Pro aminopeptidase 1 OS=Leptosomus discolor GN=N330_02526 PE=3 SV=1 | 1349947,9 | 4 | 4 |
| 416 | \|A0A091UWH6\|A0A091UWH6_NIPNI Calmodulin, striated muscle OS=Nipponia nippon GN=Y956_06192 PE=4 SV=1 | 1330646,2 | 4 | 3 |
| 417 | \|A0A091G220\|A0A091G220_9AVES Proteasome subunit beta type-6 (Fragment) OS=Cuculus canorus GN=N303_00693 PE=4 SV=1 | 1328430,6 | 3 | 2 |
| 418 | \|Q58I02\|Q58I02_CHICK Nicotinamide phosphoribosyltransferase OS=Gallus gallus GN=PBEF1 PE=2 SV=1 | 1323185,8 | 3 | 2 |
| 419 | \|Q5ZK84\|AK1A1_CHICK Alcohol dehydrogenase [NADP(+)] OS=Gallus gallus GN=AKR1A1 PE=2 SV=1 | 1293295,4 | 4 | 1 |
| 420 | \|A0A091VDP7\|A0A091VDP7_NIPNI 26S proteasome non-ATPase regulatory subunit 2 (Fragment) OS=Nipponia nippon GN=Y956_07128 PE=4 SV=1 | 1223599,9 | 1 | 1 |
| 421 | \|A0A093PPU2\|A0A093PPU2_9PASS Phosphoglucomutase-1 (Fragment) OS=Manacus vitellinus GN=N305_05300 PE=3 SV=1 | 1212042,8 | 2 | 2 |
| 422 | \|A0A0Q3M077\|A0A0Q3M077_AMAAE Uncharacterized protein OS=Amazona aestiva GN=AAES_139032 PE=4 SV=1 | 1181489,1 | 3 | 2 |
| 423 | \|A0A091UTV9\|A0A091UTV9_NIPNI Glutathione S-transferase Mu 1 (Fragment) OS=Nipponia nippon GN=Y956_08671 PE=4 SV=1 | 1165970,4 | 1 | 1 |
| 424 | \|A0A093P7Y7\|A0A093P7Y7_PYGAD Bleomycin hydrolase (Fragment) OS=Pygoscelis adeliae GN=AS28_08331 PE=4 SV=1 | 1155084,3 | 1 | 1 |
| 425 | \|A0A091VZ87\|A0A091VZ87_NIPNI Glucosamine-6-phosphate isomerase (Fragment) OS=Nipponia nippon GN=Y956_08096 PE=3 SV=1 | 1038478,3 | 2 | 2 |
| 426 | \|A0A091PNG0\|A0A091PNG0_LEPDC Ovostatin (Fragment) OS=Leptosomus discolor GN=N330_04823 PE=4 SV=1 | 1025700,9 | 1 | 1 |
| 427 | \|Q5ZIQ3\|HNRPK_CHICK Heterogeneous nuclear ribonucleoprotein K OS=Gallus gallus GN=HNRNPK PE=2 SV=1 | 1002462,0 | 2 | 2 |
| 428 | \|R0LJA8\|R0LJA8_ANAPL EGF-containing fibulin-like extracellular matrix protein 1 (Fragment) OS=Anas platyrhynchos GN=EFEMP1 PE=4 SV=1 | 874370,1 | 2 | 2 |
| 429 | \|Q9W719\|HPRT_CHICK Hypoxanthine-guanine phosphoribosyltransferase OS=Gallus gallus GN=HPRT1 PE=2 SV=1 | 851136,6 | 1 | 1 |
| 430 | \|A0A091VLE1\|A0A091VLE1_NIPNI UDP-glucose 4-epimerase (Fragment) OS=Nipponia nippon GN=Y956_14935 PE=4 SV=1 | 850510,7 | 1 | 1 |
| 431 | \|A0A0Q3X9J6\|A0A0Q3X9J6_AMAAE Glutathione S-transferase theta-1 OS=Amazona aestiva GN=AAES_16900 PE=4 SV=1 | 817448,3 | 1 | 1 |
| 432 | \|P13127\|CAZA1_CHICK F-actin-capping protein subunit alpha-1 OS=Gallus gallus GN=CAPZA1 PE=1 SV=1 | 778062,8 | 1 | 1 |
| 433 | \|A0A093HG29\|A0A093HG29_STRCA Nucleobindin-2 OS=Struthio camelus australis GN=N308_14228 PE=4 SV=1 | 744898,2 | 1 | 1 |
| 434 | \|P15787\|MT2_COLLI Metallothionein-2 OS=Columba livia PE=1 SV=2 | 710732,8 | 4 | 2 |
| 435 | \|P15164\|HBAD_APUAP Hemoglobin subunit alpha-D OS=Apus apus GN=HBAD PE=1 SV=1 | 687758,0 | 3 | 1 |
| 436 | \|A0A099ZML5\|A0A099ZML5_TINGU Gelsolin (Fragment) OS=Tinamus guttatus GN=N309_11224 PE=4 SV=1 | 687729,7 | 4 | 1 |
| 437 | \|P15165\|HBB_APUAP Hemoglobin subunit beta OS=Apus apus GN=HBB PE=1 SV=1 | 664438,9 | 1 | 1 |
| 438 | \|A0A091T959\|A0A091T959_PHALP Glyceraldehyde-3-phosphate dehydrogenase (Fragment) OS=Phaethon lepturus GN=N335_11600 PE=3 SV=1 | 616477,7 | 1 | 1 |
| 439 | \|A0A093I174\|A0A093I174_STRCA Tubulin alpha chain (Fragment) OS=Struthio camelus australis GN=N308_04558 PE=3 SV=1 | 442901,1 | 2 | 1 |
| 440 | \|G3XDD8\|LYG_DRONO Lysozyme G OS=Dromaius novaehollandiae PE=2 SV=1 | 392440,8 | 2 | 2 |
| 441 | \|A0A091TLQ1\|A0A091TLQ1_PHALP Alpha-1,4 glucan phosphorylase (Fragment) OS=Phaethon lepturus GN=N335_13746 PE=3 SV=1 | 388340,6 | 1 | 1 |
| 442 | \|A0A093FDM2\|A0A093FDM2_GAVST Leukocyte elastase inhibitor OS=Gavia stellata GN=N328_00648 PE=3 SV=1 | 384422,6 | 1 | 1 |
| 443 | \|A0A091TGS8\|A0A091TGS8_PHALP Retinol-binding protein 4 (Fragment) OS=Phaethon lepturus GN=N335_12260 PE=4 SV=1 | 360741,1 | 2 | 1 |
| 444 | \|A0A091VX00\|A0A091VX00_NIPNI BH3-interacting domain death agonist (Fragment) OS=Nipponia nippon GN=Y956_04899 PE=4 SV=1 | 321798,1 | 1 | 1 |
| 445 | \|A0A0A0A455\|A0A0A0A455_CHAVO Aldo-keto reductase family 1 member B10 (Fragment) OS=Charadrius vociferus GN=N301_16181 PE=4 SV=1 | 292332,1 | 1 | 1 |
| 446 | \|A0A0Q3M184\|A0A0Q3M184_AMAAE Galectin OS=Amazona aestiva GN=AAES_136726 PE=4 SV=1 | 213525,4 | 2 | 1 |
