# Supplementary material for: The proteomics of roadside hawk (Rupornis magnirostris), broad-snouted caiman (Caiman latirostris) and loggerhead sea turtle (Caretta caretta) tears
Source: BMC Vet Res. 2020 Aug 7;16:276. doi: 10.1186/s12917-020-02495-0 (PMC7412644; doi:10.1186/s12917-020-02495-0)
Supplement: Supplementary file 2 — Additional file 2 Table 2. Proteins identified in caiman (Caiman latirostris) tears. An Orbitrap platform was used to identify the peptides, and the results were analyzed using the Alligatoridae family database . [file 12917_2020_2495_MOESM2_ESM.docx]

**Table 2. Proteins identified in caiman (*Caiman latirostris*) tears.** An Orbitrap platform was used to identify the peptides, and the results were analyzed using the Alligatoridae family database (UNIPROT KB).

| **n.** | **Description** | **Total Intensity** | **n. of spectra** | **n. of unique peptides** |
| --- | --- | --- | --- | --- |
| 1 | \|A0A1U8CYA2\|A0A1U8CYA2_ALLSI serum albumin isoform X2 OS=Alligator sinensis GN=ALB PE=3 SV=1 | 9661398686,8 | 144 | 20 |
| 2 | \|J7H0T4\|J7H0T4_ALLSI Beta-actin OS=Alligator sinensis GN=ACTB PE=2 SV=1 | 7221749999,9 | 272 | 42 |
| 3 | \|A0A151NFL2\|A0A151NFL2_ALLMI Uncharacterized protein OS=Alligator mississippiensis GN=Y1Q_0018185 PE=4 SV=1 | 5422702270,8 | 348 | 65 |
| 4 | \|A0A1U8DH24\|A0A1U8DH24_ALLSI glutathione S-transferase Mu 3-like isoform X1 OS=Alligator sinensis GN=LOC102376491 PE=4 SV=1 | 4009086509,1 | 185 | 37 |
| 5 | \|A0A151P521\|A0A151P521_ALLMI Deleted in malignant brain tumors 1 protein-like OS=Alligator mississippiensis GN=Y1Q_0012829 PE=4 SV=1 | 3641803614,0 | 53 | 9 |
| 6 | \|A0A1U7RUW6\|A0A1U7RUW6_ALLSI protein-glutamine gamma-glutamyltransferase 4 OS=Alligator sinensis GN=TGM4 PE=4 SV=1 | 3387966969,0 | 203 | 39 |
| 7 | \|A0A1U7SGY2\|A0A1U7SGY2_ALLSI alpha-enolase OS=Alligator sinensis GN=ENO1 PE=3 SV=1 | 3038922359,7 | 211 | 31 |
| 8 | \|A0A1U8DSS5\|A0A1U8DSS5_ALLSI olfactomedin-4 OS=Alligator sinensis GN=OLFM4 PE=4 SV=1 | 2840087600,3 | 161 | 33 |
| 9 | \|A0A151MFZ6\|A0A151MFZ6_ALLMI Uncharacterized protein OS=Alligator mississippiensis GN=Y1Q_0005808 PE=4 SV=1 | 2510180976,3 | 178 | 36 |
| 10 | \|A0A1U8DL94\|A0A1U8DL94_ALLSI Ovotransferrin OS=Alligator sinensis GN=LOC102385079 PE=3 SV=1 | 2443747876,5 | 119 | 23 |
| 11 | \|A0A1U7RZ30\|A0A1U7RZ30_ALLSI Lysine--tRNA ligase OS=Alligator sinensis GN=LOC102368375 PE=3 SV=1 | 1868635477,6 | 177 | 39 |
| 12 | \|A0A1U7R1Q4\|A0A1U7R1Q4_ALLSI peroxiredoxin-1 OS=Alligator sinensis GN=PRDX1 PE=4 SV=1 | 1639857764,5 | 110 | 19 |
| 13 | \|A0A1U7SQL2\|A0A1U7SQL2_ALLSI protein-arginine deiminase type-3 OS=Alligator sinensis GN=LOC102373429 PE=4 SV=1 | 1628859547,8 | 150 | 40 |
| 14 | \|A0A1U7S0T0\|A0A1U7S0T0_ALLSI alpha-2-macroglobulin isoform X2 OS=Alligator sinensis GN=LOC102375395 PE=4 SV=1 | 1543810455,9 | 111 | 28 |
| 15 | \|A0A1U7S862\|A0A1U7S862_ALLSI myosin-9 OS=Alligator sinensis GN=MYH9 PE=3 SV=1 | 1478322918,6 | 229 | 111 |
| 16 | \|A0A151MLN4\|A0A151MLN4_ALLMI Fructose-bisphosphate aldolase OS=Alligator mississippiensis GN=Y1Q_0016059 PE=3 SV=1 | 1422207925,3 | 93 | 26 |
| 17 | \|A0A1U8DE51\|A0A1U8DE51_ALLSI Pyruvate kinase OS=Alligator sinensis GN=PKM PE=3 SV=1 | 1407949992,0 | 184 | 53 |
| 18 | \|A0A1U7SGL7\|A0A1U7SGL7_ALLSI alpha-actinin-4 OS=Alligator sinensis GN=ACTN4 PE=4 SV=1 | 1365445318,6 | 238 | 66 |
| 19 | \|A0A151NL74\|A0A151NL74_ALLMI Uncharacterized protein OS=Alligator mississippiensis GN=Y1Q_0015779 PE=4 SV=1 | 1176668852,8 | 206 | 72 |
| 20 | \|A9CPG1\|A9CPG1_ALLMI Heat shock protein 90a OS=Alligator mississippiensis GN=HSP90a PE=2 SV=1 | 1160253839,1 | 140 | 45 |
| 21 | \|A0A1U7RUK8\|A0A1U7RUK8_ALLSI 14-3-3 protein sigma OS=Alligator sinensis GN=SFN PE=3 SV=1 | 1021276485,8 | 98 | 27 |
| 22 | \|A0A151MZG8\|A0A151MZG8_ALLMI Transketolase OS=Alligator mississippiensis GN=TKT PE=4 SV=1 | 989837376,6 | 119 | 31 |
| 23 | >sp\|Q9PW06\|LDHA_ALLMI L-lactate dehydrogenase A chain OS=Alligator mississippiensis GN=LDHA PE=2 SV=3 | 983855420,1 | 101 | 27 |
| 24 | \|A9CPF4\|A9CPF4_ALLMI Heat shock protein 70B OS=Alligator mississippiensis GN=HSP70B PE=2 SV=1 | 980912240,3 | 157 | 45 |
| 25 | \|A0A151MCM1\|A0A151MCM1_ALLMI Glyceraldehyde-3-phosphate dehydrogenase OS=Alligator mississippiensis GN=GAPDH PE=3 SV=1 | 980232123,7 | 102 | 23 |
| 26 | \|A0A151N9I2\|A0A151N9I2_ALLMI Glutathione S-transferase Mu 1 OS=Alligator mississippiensis GN=Y1Q_0014943 PE=4 SV=1 | 976753056,9 | 68 | 15 |
| 27 | \|A0A151MCS3\|A0A151MCS3_ALLMI Triosephosphate isomerase OS=Alligator mississippiensis GN=TPI1 PE=3 SV=1 | 973441812,6 | 82 | 16 |
| 28 | \|A0A151MU53\|A0A151MU53_ALLMI Elongation factor 1-alpha OS=Alligator mississippiensis GN=EEF1A2 PE=3 SV=1 | 957499165,6 | 100 | 27 |
| 29 | \|A0A151MZH9\|A0A151MZH9_ALLMI Tubulin beta chain OS=Alligator mississippiensis GN=Y1Q_0022913 PE=3 SV=1 | 911082034,5 | 134 | 34 |
| 30 | \|A0A1U7RSA6\|A0A1U7RSA6_ALLSI Alpha-1,4 glucan phosphorylase OS=Alligator sinensis GN=PYGL PE=3 SV=1 | 908067962,0 | 138 | 46 |
| 31 | \|A0A151NFA0\|A0A151NFA0_ALLMI Leukocyte elastase inhibitor isoform A OS=Alligator mississippiensis GN=SERPINB1 PE=3 SV=1 | 860473537,1 | 101 | 30 |
| 32 | \|A0A1U8D492\|A0A1U8D492_ALLSI villin-like protein OS=Alligator sinensis GN=VILL PE=4 SV=1 | 854931639,1 | 121 | 38 |
| 33 | \|A0A1U7R8Q2\|A0A1U7R8Q2_ALLSI Transaldolase OS=Alligator sinensis GN=TALDO1 PE=3 SV=1 | 837747780,0 | 102 | 25 |
| 34 | \|A0A1U7SCC5\|A0A1U7SCC5_ALLSI 14-3-3 protein zeta/delta OS=Alligator sinensis GN=YWHAZ PE=3 SV=1 | 766435318,1 | 77 | 17 |
| 35 | \|A0A1U7QZQ6\|A0A1U7QZQ6_ALLSI Annexin OS=Alligator sinensis GN=ANXA8L1 PE=3 SV=1 | 751800164,0 | 62 | 15 |
| 36 | \|A0A151NPS7\|A0A151NPS7_ALLMI Rab GDP dissociation inhibitor OS=Alligator mississippiensis GN=GDI2 PE=3 SV=1 | 749400597,9 | 126 | 38 |
| 37 | \|A0A1U7SCP6\|A0A1U7SCP6_ALLSI Glucose-6-phosphate isomerase OS=Alligator sinensis GN=GPI PE=3 SV=1 | 720011068,5 | 92 | 25 |
| 38 | \|A0A151MLG9\|A0A151MLG9_ALLMI 14-3-3 protein epsilon OS=Alligator mississippiensis GN=YWHAE PE=3 SV=1 | 664492411,9 | 80 | 24 |
| 39 | \|A0A1U7RLN0\|A0A1U7RLN0_ALLSI GDP-mannose 4,6 dehydratase OS=Alligator sinensis GN=GMDS PE=3 SV=1 | 660621589,4 | 80 | 21 |
| 40 | \|A0A151NNU5\|A0A151NNU5_ALLMI NAD(P)H dehydrogenase [quinone] 1 OS=Alligator mississippiensis GN=NQO1 PE=4 SV=1 | 658447101,1 | 71 | 13 |
| 41 | \|A0A1U7S835\|A0A1U7S835_ALLSI unconventional myosin-Ic isoform X1 OS=Alligator sinensis GN=MYO1C PE=3 SV=1 | 637133048,0 | 32 | 8 |
| 42 | \|A0A1U8DBN5\|A0A1U8DBN5_ALLSI Fructose-bisphosphate aldolase OS=Alligator sinensis GN=ALDOB PE=3 SV=1 | 634397760,5 | 61 | 15 |
| 43 | \|A0A1U7S2F0\|A0A1U7S2F0_ALLSI argininosuccinate synthase OS=Alligator sinensis GN=ASS1 PE=3 SV=1 | 626177704,1 | 96 | 28 |
| 44 | \|A0A151M8E2\|A0A151M8E2_ALLMI Retinal dehydrogenase 1 OS=Alligator mississippiensis GN=ALDH1A1 PE=3 SV=1 | 624449715,3 | 124 | 30 |
| 45 | \|A0A151N3V9\|A0A151N3V9_ALLMI 6-phosphogluconate dehydrogenase, decarboxylating OS=Alligator mississippiensis GN=PGD PE=3 SV=1 | 618083245,5 | 107 | 33 |
| 46 | \|A0A151MDZ9\|A0A151MDZ9_ALLMI Superoxide dismutase [Cu-Zn] OS=Alligator mississippiensis GN=SOD1 PE=3 SV=1 | 612018518,2 | 39 | 6 |
| 47 | \|A0A1U7S9N4\|A0A1U7S9N4_ALLSI filamin-B isoform X3 OS=Alligator sinensis GN=FLNB PE=4 SV=1 | 604222987,6 | 181 | 79 |
| 48 | \|A0A151M299\|A0A151M299_ALLMI Uncharacterized protein OS=Alligator mississippiensis GN=Y1Q_0009110 PE=4 SV=1 | 544733763,3 | 121 | 45 |
| 49 | \|A0A151MIW1\|A0A151MIW1_ALLMI Antithrombin-III OS=Alligator mississippiensis GN=SERPINC1 PE=3 SV=1 | 528062122,6 | 76 | 27 |
| 50 | \|A0A1U8DDU3\|A0A1U8DDU3_ALLSI LOW QUALITY PROTEIN: C-factor-like OS=Alligator sinensis GN=LOC102370007 PE=3 SV=1 | 526555240,4 | 90 | 21 |
| 51 | \|A0A1U7RH57\|A0A1U7RH57_ALLSI Glycerol-3-phosphate dehydrogenase [NAD(+)] OS=Alligator sinensis GN=LOC102371629 PE=3 SV=1 | 522503805,3 | 57 | 21 |
| 52 | \|A0A151MKE5\|A0A151MKE5_ALLMI 26S proteasome non-ATPase regulatory subunit 6 OS=Alligator mississippiensis GN=PSMD6 PE=4 SV=1 | 517962742,7 | 25 | 11 |
| 53 | \|A0A151NM72\|A0A151NM72_ALLMI Complement factor H OS=Alligator mississippiensis GN=CFHR2 PE=4 SV=1 | 504240694,1 | 43 | 11 |
| 54 | \|A0A151N832\|A0A151N832_ALLMI 14-3-3 protein theta OS=Alligator mississippiensis GN=YWHAQ PE=3 SV=1 | 503922612,7 | 54 | 15 |
| 55 | \|A0A151MWJ6\|A0A151MWJ6_ALLMI Uncharacterized protein OS=Alligator mississippiensis GN=Y1Q_0009722 PE=4 SV=1 | 486520760,1 | 101 | 40 |
| 56 | \|A0A151MPW6\|A0A151MPW6_ALLMI Annexin OS=Alligator mississippiensis GN=ANXA3 PE=3 SV=1 | 482084269,6 | 105 | 27 |
| 57 | \|A0A151NJF4\|A0A151NJF4_ALLMI Uncharacterized protein OS=Alligator mississippiensis GN=Y1Q_0020931 PE=3 SV=1 | 478579134,9 | 97 | 34 |
| 58 | \|A0A1U8DMI0\|A0A1U8DMI0_ALLSI ubiquitin-like modifier-activating enzyme 1 OS=Alligator sinensis GN=UBA1 PE=3 SV=1 | 473210996,4 | 106 | 38 |
| 59 | \|A0A151MXB0\|A0A151MXB0_ALLMI Uncharacterized protein OS=Alligator mississippiensis GN=Y1Q_0009929 PE=3 SV=1 | 469381260,6 | 19 | 2 |
| 60 | \|A0A151NSI2\|A0A151NSI2_ALLMI Peroxiredoxin-6 OS=Alligator mississippiensis GN=PRDX6 PE=4 SV=1 | 465954886,1 | 65 | 16 |
| 61 | \|A0A1U7SEJ5\|A0A1U7SEJ5_ALLSI selenium-binding protein 1 OS=Alligator sinensis GN=SELENBP1 PE=4 SV=1 | 461895328,9 | 105 | 29 |
| 62 | \|A0A151NIL0\|A0A151NIL0_ALLMI Aldo-keto reductase family 1, member B1-like OS=Alligator mississippiensis GN=AKR1B1L PE=4 SV=1 | 456066850,5 | 52 | 13 |
| 63 | \|A0A1U7RUP1\|A0A1U7RUP1_ALLSI Adenylyl cyclase-associated protein OS=Alligator sinensis GN=CAP1 PE=3 SV=1 | 438824639,4 | 80 | 27 |
| 64 | \|A0A1U7RT30\|A0A1U7RT30_ALLSI Nucleoside diphosphate kinase OS=Alligator sinensis GN=LOC102374469 PE=3 SV=1 | 434256025,8 | 55 | 17 |
| 65 | \|A0A151NU00\|A0A151NU00_ALLMI Nicotinamide N-methyltransferase OS=Alligator mississippiensis GN=NNMT PE=4 SV=1 | 426174216,3 | 34 | 7 |
| 66 | \|A0A151N6T2\|A0A151N6T2_ALLMI Clathrin heavy chain OS=Alligator mississippiensis GN=Y1Q_0007208 PE=3 SV=1 | 413860490,0 | 119 | 59 |
| 67 | \|A0A1U7REG0\|A0A1U7REG0_ALLSI fructose-1,6-bisphosphatase 1 OS=Alligator sinensis GN=FBP1 PE=3 SV=1 | 409895223,8 | 68 | 19 |
| 68 | \|A0A151NXF1\|A0A151NXF1_ALLMI Ribonuclease inhibitor OS=Alligator mississippiensis GN=RNH1 PE=4 SV=1 | 400156610,7 | 72 | 19 |
| 69 | \|A0A1U8DI15\|A0A1U8DI15_ALLSI neuroblast differentiation-associated protein AHNAK isoform X2 OS=Alligator sinensis GN=AHNAK PE=4 SV=1 | 397334659,3 | 98 | 47 |
| 70 | \|A0A151P9Q4\|A0A151P9Q4_ALLMI WD repeat-containing protein 1 OS=Alligator mississippiensis GN=WDR1 PE=4 SV=1 | 396517525,0 | 62 | 23 |
| 71 | \|A0A151PJ40\|A0A151PJ40_ALLMI Tropomyosin alpha-3 chain isoform C OS=Alligator mississippiensis GN=TPM3-1 PE=3 SV=1 | 394937901,9 | 60 | 26 |
| 72 | \|A0A151MU30\|A0A151MU30_ALLMI Malic enzyme OS=Alligator mississippiensis GN=ME1 PE=3 SV=1 | 394201626,9 | 76 | 26 |
| 73 | \|A0A151MTJ7\|A0A151MTJ7_ALLMI Phosphoglycerate mutase OS=Alligator mississippiensis GN=PGAM1 PE=3 SV=1 | 389791304,1 | 38 | 11 |
| 74 | >sp\|Q98SL1\|LDHB_CAICA L-lactate dehydrogenase B chain OS=Caiman crocodilus apaporiensis GN=LDHB PE=2 SV=3 | 382002994,9 | 49 | 14 |
| 75 | \|A0A1U7S4E8\|A0A1U7S4E8_ALLSI Pyruvate kinase OS=Alligator sinensis GN=PKLR PE=3 SV=1 | 371999639,8 | 109 | 42 |
| 76 | \|A0A1U7SGG9\|A0A1U7SGG9_ALLSI Tubulin alpha chain OS=Alligator sinensis GN=LOC102384891 PE=3 SV=1 | 370358002,4 | 90 | 24 |
| 77 | \|A0A1U7RYA5\|A0A1U7RYA5_ALLSI ras GTPase-activating protein-binding protein 1 isoform X1 OS=Alligator sinensis GN=G3BP1 PE=4 SV=1 | 369456513,2 | 5 | 3 |
| 78 | \|A0A1U7S0W2\|A0A1U7S0W2_ALLSI ovostatin-like OS=Alligator sinensis GN=LOC102388541 PE=4 SV=1 | 363962717,5 | 39 | 8 |
| 79 | \|A0A1U7SXY4\|A0A1U7SXY4_ALLSI rho GDP-dissociation inhibitor 1 OS=Alligator sinensis GN=ARHGDIA PE=4 SV=1 | 360125777,4 | 55 | 12 |
| 80 | \|A0A1U7S1A4\|A0A1U7S1A4_ALLSI glutamine--fructose-6-phosphate aminotransferase [isomerizing] 1 OS=Alligator sinensis GN=GFPT1 PE=4 SV=1 | 359031634,3 | 83 | 36 |
| 81 | \|A0A1U7RXV5\|A0A1U7RXV5_ALLSI prostaglandin reductase 1 OS=Alligator sinensis GN=PTGR1 PE=4 SV=1 | 355591906,9 | 39 | 16 |
| 82 | \|A0A1U8DHP2\|A0A1U8DHP2_ALLSI thymosin beta-15B OS=Alligator sinensis GN=TMSB15B PE=4 SV=1 | 351829919,0 | 25 | 6 |
| 83 | \|A0A1U7SI08\|A0A1U7SI08_ALLSI ras GTPase-activating-like protein IQGAP1 OS=Alligator sinensis GN=IQGAP1 PE=4 SV=1 | 351797819,5 | 137 | 56 |
| 84 | \|A0A151MGK7\|A0A151MGK7_ALLMI Malate dehydrogenase OS=Alligator mississippiensis GN=MDH1 PE=3 SV=1 | 349742270,7 | 39 | 14 |
| 85 | >sp\|P02131\|HBB_CAICR Hemoglobin subunit beta OS=Caiman crocodilus GN=HBB PE=1 SV=1 | 341857936,9 | 43 | 11 |
| 86 | \|A0A151PC06\|A0A151PC06_ALLMI Fibrinogen beta chain OS=Alligator mississippiensis GN=FGB PE=4 SV=1 | 341496823,9 | 70 | 18 |
| 87 | \|A0A151NGB2\|A0A151NGB2_ALLMI Hemopexin OS=Alligator mississippiensis GN=HPX PE=4 SV=1 | 337412563,8 | 66 | 12 |
| 88 | \|A0A151NNS5\|A0A151NNS5_ALLMI Phosphoglycerate kinase OS=Alligator mississippiensis GN=PGK1 PE=3 SV=1 | 335699911,8 | 91 | 27 |
| 89 | \|A0A1U8D6C6\|A0A1U8D6C6_ALLSI LOW QUALITY PROTEIN: myosin-10-like OS=Alligator sinensis GN=LOC102371226 PE=3 SV=1 | 332001986,6 | 106 | 57 |
| 90 | \|A0A1U8DBR6\|A0A1U8DBR6_ALLSI Plasminogen OS=Alligator sinensis GN=LOC102377482 PE=3 SV=1 | 324648033,6 | 61 | 24 |
| 91 | \|A0A151N583\|A0A151N583_ALLMI Uncharacterized protein OS=Alligator mississippiensis GN=Y1Q_0012178 PE=3 SV=1 | 324479958,1 | 63 | 19 |
| 92 | \|A0A151NPX3\|A0A151NPX3_ALLMI Calmodulin-alpha-like OS=Alligator mississippiensis GN=Y1Q_0023495 PE=4 SV=1 | 324378478,1 | 36 | 10 |
| 93 | \|A0A151NTQ4\|A0A151NTQ4_ALLMI Uncharacterized protein OS=Alligator mississippiensis GN=Y1Q_0019431 PE=3 SV=1 | 318089622,5 | 89 | 38 |
| 94 | \|A9CPF2\|A9CPF2_ALLMI Heat shock protein 70A OS=Alligator mississippiensis GN=HSP70A PE=2 SV=1 | 315826224,9 | 78 | 27 |
| 95 | \|A0A151PC64\|A0A151PC64_ALLMI Keratin, type I cytoskeletal 19 OS=Alligator mississippiensis GN=KRT19 PE=3 SV=1 | 311872599,6 | 60 | 21 |
| 96 | \|A0A151NE24\|A0A151NE24_ALLMI Destrin OS=Alligator mississippiensis GN=DSTN PE=3 SV=1 | 299296449,8 | 43 | 15 |
| 97 | \|A0A151N9I7\|A0A151N9I7_ALLMI Isocitrate dehydrogenase [NADP] OS=Alligator mississippiensis GN=Y1Q_0008660 PE=3 SV=1 | 296346583,4 | 62 | 22 |
| 98 | \|A0A1U8CZN4\|A0A1U8CZN4_ALLSI fatty acid-binding protein, epidermal OS=Alligator sinensis GN=LOC102381973 PE=3 SV=1 | 290430495,4 | 22 | 6 |
| 99 | \|A0A1U7RBI2\|A0A1U7RBI2_ALLSI gelsolin isoform X3 OS=Alligator sinensis GN=GSN PE=4 SV=1 | 287488762,7 | 30 | 6 |
| 100 | \|A0A151MF96\|A0A151MF96_ALLMI Uncharacterized protein OS=Alligator mississippiensis GN=Y1Q_0005628 PE=4 SV=1 | 287048835,5 | 18 | 5 |
| 101 | \|A0A1U8DWS5\|A0A1U8DWS5_ALLSI complement factor B OS=Alligator sinensis GN=CFB PE=4 SV=1 | 284771203,5 | 39 | 18 |
| 102 | \|A0A151PG92\|A0A151PG92_ALLMI Alcohol dehydrogenase [NADP(+)] isoform A OS=Alligator mississippiensis GN=AKR1A1-1 PE=4 SV=1 | 281231430,1 | 47 | 12 |
| 103 | \|A0A1U8CUS1\|A0A1U8CUS1_ALLSI aspartate--tRNA ligase, cytoplasmic isoform X2 OS=Alligator sinensis GN=DARS PE=3 SV=1 | 278374057,5 | 50 | 21 |
| 104 | \|A0A151NLY8\|A0A151NLY8_ALLMI Proteasome activator complex subunit 2 OS=Alligator mississippiensis GN=PSME2 PE=4 SV=1 | 275992827,9 | 64 | 17 |
| 105 | \|A0A1U8DJP2\|A0A1U8DJP2_ALLSI von Willebrand factor A domain-containing protein 7 OS=Alligator sinensis GN=VWA7 PE=4 SV=1 | 274994194,5 | 24 | 7 |
| 106 | \|A0A151N686\|A0A151N686_ALLMI Myosin light polypeptide 6 isoform B OS=Alligator mississippiensis GN=MYL6-1 PE=4 SV=1 | 269425517,3 | 43 | 12 |
| 107 | \|A0A1U7SST1\|A0A1U7SST1_ALLSI hemopexin OS=Alligator sinensis GN=HPX PE=4 SV=1 | 268709453,5 | 17 | 3 |
| 108 | \|A0A1U7SWQ7\|A0A1U7SWQ7_ALLSI Aminopeptidase OS=Alligator sinensis GN=NPEPPS PE=3 SV=1 | 268100965,4 | 90 | 33 |
| 109 | \|A0A1U8DT30\|A0A1U8DT30_ALLSI von Willebrand factor A domain-containing protein 5A-like OS=Alligator sinensis GN=LOC102369335 PE=4 SV=1 | 263284379,0 | 47 | 14 |
| 110 | \|A0A1U8DC15\|A0A1U8DC15_ALLSI phosphoglucomutase-2 OS=Alligator sinensis GN=PGM2 PE=4 SV=1 | 260137465,9 | 42 | 16 |
| 111 | \|A0A151NFJ9\|A0A151NFJ9_ALLMI Uncharacterized protein OS=Alligator mississippiensis GN=Y1Q_0018187 PE=4 SV=1 | 258558606,6 | 13 | 3 |
| 112 | \|A0A1U7RSI5\|A0A1U7RSI5_ALLSI cell division control protein 42 homolog isoform X1 OS=Alligator sinensis GN=CDC42 PE=3 SV=1 | 258129562,6 | 34 | 9 |
| 113 | \|A0A1U8D644\|A0A1U8D644_ALLSI riboflavin-binding protein-like OS=Alligator sinensis GN=LOC102380528 PE=4 SV=1 | 255277651,1 | 16 | 6 |
| 114 | \|A0A151MZ81\|A0A151MZ81_ALLMI Protein disulfide-isomerase OS=Alligator mississippiensis GN=P4HB PE=3 SV=1 | 254897138,9 | 55 | 21 |
| 115 | \|A0A151P3C9\|A0A151P3C9_ALLMI 3'(2'),5'-bisphosphate nucleotidase 1 isoform B OS=Alligator mississippiensis GN=BPNT1-1 PE=4 SV=1 | 252660895,6 | 40 | 14 |
| 116 | \|A0A1U7RTA3\|A0A1U7RTA3_ALLSI alpha-1-antitrypsin-like OS=Alligator sinensis GN=LOC102370152 PE=3 SV=1 | 250670580,8 | 25 | 8 |
| 117 | \|A0A1U7SAD2\|A0A1U7SAD2_ALLSI fibrinogen gamma chain OS=Alligator sinensis GN=FGG PE=4 SV=1 | 247556410,3 | 53 | 20 |
| 118 | \|A0A1U7SC00\|A0A1U7SC00_ALLSI adseverin OS=Alligator sinensis GN=SCIN PE=4 SV=1 | 244988913,3 | 43 | 24 |
| 119 | \|A0A151P8J8\|A0A151P8J8_ALLMI Uncharacterized protein OS=Alligator mississippiensis GN=Y1Q_0015079 PE=4 SV=1 | 244763283,0 | 107 | 68 |
| 120 | \|A0A151M2M5\|A0A151M2M5_ALLMI Uncharacterized protein OS=Alligator mississippiensis GN=Y1Q_0009210 PE=3 SV=1 | 242995159,4 | 22 | 4 |
| 121 | \|A0A151M8F4\|A0A151M8F4_ALLMI Annexin OS=Alligator mississippiensis GN=ANXA1 PE=3 SV=1 | 239966261,3 | 42 | 13 |
| 122 | \|A0A1U8D2D5\|A0A1U8D2D5_ALLSI LOW QUALITY PROTEIN: programmed cell death 6-interacting protein OS=Alligator sinensis GN=PDCD6IP PE=4 SV=1 | 232132271,4 | 66 | 27 |
| 123 | \|A0A151MYJ9\|A0A151MYJ9_ALLMI Peroxiredoxin-5, mitochondrial OS=Alligator mississippiensis GN=PRDX5 PE=4 SV=1 | 231067163,0 | 9 | 4 |
| 124 | \|A0A1U7R7Q4\|A0A1U7R7Q4_ALLSI Galectin OS=Alligator sinensis GN=LGALS3 PE=4 SV=1 | 227716787,4 | 27 | 8 |
| 125 | \|A0A1U7SG38\|A0A1U7SG38_ALLSI LOW QUALITY PROTEIN: niban-like protein 1 OS=Alligator sinensis GN=FAM129B PE=4 SV=1 | 225773857,2 | 45 | 22 |
| 126 | \|A0A1U8DP95\|A0A1U8DP95_ALLSI F-actin-capping protein subunit beta isoform X2 OS=Alligator sinensis GN=CAPZB PE=4 SV=1 | 223174323,4 | 43 | 19 |
| 127 | \|A0A151N4I2\|A0A151N4I2_ALLMI Phosphatidylethanolamine-binding protein 1 OS=Alligator mississippiensis GN=PEBP1 PE=4 SV=1 | 222884153,7 | 28 | 10 |
| 128 | \|A0A1U7SCG4\|A0A1U7SCG4_ALLSI Glucose-6-phosphate 1-dehydrogenase OS=Alligator sinensis GN=G6PD PE=3 SV=1 | 220164468,7 | 55 | 25 |
| 129 | \|A0A1U8D3J0\|A0A1U8D3J0_ALLSI polymeric immunoglobulin receptor OS=Alligator sinensis GN=PIGR PE=4 SV=1 | 220162549,4 | 23 | 9 |
| 130 | \|A0A1U7SCL3\|A0A1U7SCL3_ALLSI tubulin polymerization-promoting protein family member 3 OS=Alligator sinensis GN=TPPP3 PE=4 SV=1 | 218127288,2 | 26 | 9 |
| 131 | \|A0A151N795\|A0A151N795_ALLMI Glucose-6-phosphate isomerase OS=Alligator mississippiensis GN=GPI PE=3 SV=1 | 217128165,2 | 19 | 7 |
| 132 | \|A0A1U8DEX6\|A0A1U8DEX6_ALLSI GDP-L-fucose synthase OS=Alligator sinensis GN=TSTA3 PE=3 SV=1 | 216993278,0 | 41 | 16 |
| 133 | \|A0A151MA32\|A0A151MA32_ALLMI Annexin OS=Alligator mississippiensis GN=ICE2 PE=3 SV=1 | 212613039,0 | 38 | 16 |
| 134 | \|A0A1U7RB22\|A0A1U7RB22_ALLSI 14-3-3 protein beta/alpha OS=Alligator sinensis GN=YWHAB PE=3 SV=1 | 212361428,5 | 29 | 12 |
| 135 | \|A0A151NEI0\|A0A151NEI0_ALLMI Actin-like protein 7A isoform A OS=Alligator mississippiensis GN=ACTL7A-1 PE=3 SV=1 | 211868509,6 | 9 | 4 |
| 136 | \|A0A151MC38\|A0A151MC38_ALLMI Proteasome subunit alpha type OS=Alligator mississippiensis GN=PSMA1 PE=3 SV=1 | 208747489,3 | 56 | 23 |
| 137 | >sp\|P02000\|HBA_CAICR Hemoglobin subunit alpha OS=Caiman crocodilus GN=HBA PE=1 SV=1 | 207726829,1 | 36 | 7 |
| 138 | \|A0A151PBF3\|A0A151PBF3_ALLMI Fibrinogen alpha chain OS=Alligator mississippiensis GN=FGA PE=4 SV=1 | 207598933,3 | 50 | 18 |
| 139 | \|A0A1U7SBE6\|A0A1U7SBE6_ALLSI Sulfotransferase OS=Alligator sinensis GN=LOC102375066 PE=3 SV=1 | 207473826,0 | 26 | 7 |
| 140 | \|A0A151LZJ0\|A0A151LZJ0_ALLMI Uncharacterized protein OS=Alligator mississippiensis GN=Y1Q_0014258 PE=3 SV=1 | 204180268,7 | 31 | 13 |
| 141 | \|A0A1U7S4G2\|A0A1U7S4G2_ALLSI serine/threonine-protein phosphatase 2A 65 kDa regulatory subunit A alpha isoform OS=Alligator sinensis GN=PPP2R1A PE=4 SV=1 | 203764673,9 | 58 | 28 |
| 142 | \|A0A151N1E3\|A0A151N1E3_ALLMI Phosphomannomutase OS=Alligator mississippiensis GN=PMM2 PE=3 SV=1 | 198213680,1 | 37 | 11 |
| 143 | \|A0A1U7SCG5\|A0A1U7SCG5_ALLSI Peptidylprolyl isomerase OS=Alligator sinensis GN=FKBP4 PE=4 SV=1 | 197198445,0 | 55 | 20 |
| 144 | \|A0A151PIC2\|A0A151PIC2_ALLMI Calpastatin isoform B OS=Alligator mississippiensis GN=CAST PE=4 SV=1 | 195170983,5 | 48 | 18 |
| 145 | \|A0A1U7SRC7\|A0A1U7SRC7_ALLSI TRPM8 channel-associated factor 2 isoform X1 OS=Alligator sinensis GN=TCAF2 PE=4 SV=1 | 193503496,8 | 46 | 18 |
| 146 | \|A0A151M7F5\|A0A151M7F5_ALLMI Heterogeneous nuclear ribonucleoprotein H isoform A OS=Alligator mississippiensis GN=HNRNPH1 PE=4 SV=1 | 190669755,6 | 37 | 12 |
| 147 | \|A0A1U7S5V9\|A0A1U7S5V9_ALLSI keratin, type II cytoskeletal cochleal-like OS=Alligator sinensis GN=LOC102371330 PE=3 SV=1 | 186610662,0 | 41 | 17 |
| 148 | \|A0A1U7RF13\|A0A1U7RF13_ALLSI vitamin D-binding protein isoform X2 OS=Alligator sinensis GN=GC PE=3 SV=1 | 186104125,6 | 44 | 13 |
| 149 | \|A0A151MTG6\|A0A151MTG6_ALLMI Transforming protein RhoA OS=Alligator mississippiensis GN=RHOA PE=3 SV=1 | 184332688,6 | 36 | 12 |
| 150 | \|A0A1U7SUL9\|A0A1U7SUL9_ALLSI stress-induced-phosphoprotein 1 OS=Alligator sinensis GN=STIP1 PE=4 SV=1 | 182200238,2 | 43 | 21 |
| 151 | \|A0A151P8U9\|A0A151P8U9_ALLMI Alpha-1-antitrypsin OS=Alligator mississippiensis GN=SERPINA1 PE=3 SV=1 | 181835214,5 | 14 | 5 |
| 152 | \|A0A151NZV6\|A0A151NZV6_ALLMI Inorganic pyrophosphatase OS=Alligator mississippiensis GN=PPA1 PE=4 SV=1 | 180191232,9 | 36 | 14 |
| 153 | \|A0A1U7RFN2\|A0A1U7RFN2_ALLSI GTP-binding nuclear protein Ran OS=Alligator sinensis GN=RAN PE=3 SV=1 | 178965465,1 | 24 | 9 |
| 154 | \|A0A151N1X2\|A0A151N1X2_ALLMI Uncharacterized protein OS=Alligator mississippiensis GN=Y1Q_0014343 PE=4 SV=1 | 177977540,5 | 33 | 12 |
| 155 | \|A0A1U7S7E8\|A0A1U7S7E8_ALLSI calpain small subunit 1 OS=Alligator sinensis GN=CAPNS1 PE=4 SV=1 | 175763290,2 | 30 | 8 |
| 156 | \|A0A1U7RHX0\|A0A1U7RHX0_ALLSI envoplakin isoform X2 OS=Alligator sinensis GN=EVPL PE=4 SV=1 | 173403295,2 | 42 | 29 |
| 157 | \|A0A151M101\|A0A151M101_ALLMI Translin OS=Alligator mississippiensis GN=TSN PE=4 SV=1 | 169941266,5 | 33 | 13 |
| 158 | \|A0A1U7S1C8\|A0A1U7S1C8_ALLSI ceruloplasmin OS=Alligator sinensis GN=CP PE=3 SV=1 | 166928117,9 | 39 | 15 |
| 159 | \|A0A151PJB0\|A0A151PJB0_ALLMI Protein disulfide-isomerase OS=Alligator mississippiensis GN=PDIA3 PE=3 SV=1 | 164555520,0 | 48 | 19 |
| 160 | \|A0A1U7SIL4\|A0A1U7SIL4_ALLSI Fructose-bisphosphate aldolase OS=Alligator sinensis GN=ALDOA PE=3 SV=1 | 164031425,2 | 11 | 3 |
| 161 | \|A0A151MUD7\|A0A151MUD7_ALLMI Coronin OS=Alligator mississippiensis GN=Y1Q_0005078 PE=3 SV=1 | 163273088,3 | 39 | 13 |
| 162 | \|A0A151P621\|A0A151P621_ALLMI Aspartate aminotransferase OS=Alligator mississippiensis GN=GOT1 PE=4 SV=1 | 162554141,9 | 40 | 20 |
| 163 | \|A0A151M028\|A0A151M028_ALLMI Lupus La protein OS=Alligator mississippiensis GN=SSB PE=4 SV=1 | 162134098,0 | 45 | 19 |
| 164 | \|A0A151MFF0\|A0A151MFF0_ALLMI Spectrin alpha chain, erythrocytic 1 isoform A OS=Alligator mississippiensis GN=SPTA1 PE=4 SV=1 | 159317128,3 | 60 | 37 |
| 165 | \|A0A151NKR0\|A0A151NKR0_ALLMI Alanine aminotransferase 2 OS=Alligator mississippiensis GN=GPT2 PE=4 SV=1 | 158091568,2 | 34 | 14 |
| 166 | \|A0A1U7S221\|A0A1U7S221_ALLSI argininosuccinate lyase OS=Alligator sinensis GN=ASL PE=3 SV=1 | 157106342,4 | 33 | 10 |
| 167 | \|A0A1U7RL72\|A0A1U7RL72_ALLSI vinculin isoform X1 OS=Alligator sinensis GN=VCL PE=4 SV=1 | 153389351,2 | 51 | 30 |
| 168 | \|A0A151M0S1\|A0A151M0S1_ALLMI Actin-related protein 3 OS=Alligator mississippiensis GN=ACTR3 PE=3 SV=1 | 151748084,0 | 51 | 18 |
| 169 | \|A0A151N5M3\|A0A151N5M3_ALLMI Lissencephaly-1 homolog OS=Alligator mississippiensis GN=PAFAH1B1 PE=3 SV=1 | 150276487,4 | 30 | 19 |
| 170 | \|A0A151NUK3\|A0A151NUK3_ALLMI Glutathione S-transferase omega-1 OS=Alligator mississippiensis GN=GSTO1 PE=4 SV=1 | 150087013,5 | 39 | 12 |
| 171 | \|A0A151N7H6\|A0A151N7H6_ALLMI Uncharacterized protein OS=Alligator mississippiensis GN=Y1Q_0009322 PE=4 SV=1 | 150021933,8 | 55 | 28 |
| 172 | \|A0A1U7R7I8\|A0A1U7R7I8_ALLSI heat shock protein HSP 90-beta OS=Alligator sinensis GN=HSP90AB1 PE=3 SV=1 | 149950042,7 | 47 | 18 |
| 173 | \|A0A151NSN2\|A0A151NSN2_ALLMI Actin-related protein 2/3 complex subunit 3 OS=Alligator mississippiensis GN=ARPC3 PE=3 SV=1 | 148907984,0 | 35 | 12 |
| 174 | \|A0A151MHF9\|A0A151MHF9_ALLMI Uncharacterized protein OS=Alligator mississippiensis GN=Y1Q_0015916 PE=3 SV=1 | 148543753,6 | 34 | 13 |
| 175 | \|A0A151NZC3\|A0A151NZC3_ALLMI Adenosylhomocysteinase OS=Alligator mississippiensis GN=AHCY PE=3 SV=1 | 147886259,8 | 31 | 12 |
| 176 | \|A0A151P5V6\|A0A151P5V6_ALLMI Uncharacterized protein OS=Alligator mississippiensis GN=Y1Q_0016759 PE=3 SV=1 | 147559567,4 | 33 | 11 |
| 177 | \|A0A151NFI2\|A0A151NFI2_ALLMI Serpin B5 isoform B OS=Alligator mississippiensis GN=SERPINB5-1 PE=3 SV=1 | 145901317,5 | 56 | 15 |
| 178 | \|A0A1U8D0F8\|A0A1U8D0F8_ALLSI heterogeneous nuclear ribonucleoprotein K isoform X4 OS=Alligator sinensis GN=HNRNPK PE=4 SV=1 | 145806363,1 | 40 | 13 |
| 179 | \|A0A151M8D7\|A0A151M8D7_ALLMI Guanine deaminase OS=Alligator mississippiensis GN=GDA PE=4 SV=1 | 144884654,3 | 39 | 20 |
| 180 | \|A0A1U8D7B4\|A0A1U8D7B4_ALLSI alpha-1-antiproteinase-like OS=Alligator sinensis GN=LOC102375061 PE=3 SV=1 | 144650897,8 | 5 | 2 |
| 181 | \|A0A1U7SXQ0\|A0A1U7SXQ0_ALLSI apolipoprotein E OS=Alligator sinensis GN=APOE PE=3 SV=1 | 143578161,2 | 24 | 12 |
| 182 | \|A0A151MYL4\|A0A151MYL4_ALLMI T-complex protein 1 subunit eta OS=Alligator mississippiensis GN=CCT7 PE=3 SV=1 | 142955825,4 | 37 | 14 |
| 183 | \|A0A1U8DQE8\|A0A1U8DQE8_ALLSI ATPase family AAA domain-containing protein 5 OS=Alligator sinensis GN=ATAD5 PE=4 SV=1 | 142285535,5 | 9 | 5 |
| 184 | \|A0A151P2V2\|A0A151P2V2_ALLMI Arp2/3 complex 34 kDa subunit OS=Alligator mississippiensis GN=ARPC2 PE=3 SV=1 | 141406062,8 | 40 | 16 |
| 185 | \|A0A151N1N8\|A0A151N1N8_ALLMI Ras-related C3 botulinum toxin substrate 1 OS=Alligator mississippiensis GN=RAC1 PE=3 SV=1 | 139813969,0 | 29 | 10 |
| 186 | \|A0A1U8CZ74\|A0A1U8CZ74_ALLSI T-complex protein 1 subunit theta isoform X1 OS=Alligator sinensis GN=CCT8 PE=3 SV=1 | 137547497,4 | 40 | 20 |
| 187 | \|A0A1U7SH29\|A0A1U7SH29_ALLSI Mitogen-activated protein kinase OS=Alligator sinensis GN=LOC102375250 PE=3 SV=1 | 136422798,2 | 30 | 11 |
| 188 | \|A0A151NYA3\|A0A151NYA3_ALLMI T-complex protein 1 subunit gamma OS=Alligator mississippiensis GN=CCT3 PE=3 SV=1 | 135297240,6 | 41 | 22 |
| 189 | \|A0A151M7D0\|A0A151M7D0_ALLMI Coatomer subunit alpha OS=Alligator mississippiensis GN=COPA PE=4 SV=1 | 135197468,8 | 43 | 23 |
| 190 | \|A0A151MIQ2\|A0A151MIQ2_ALLMI Complement factor I OS=Alligator mississippiensis GN=CFI PE=3 SV=1 | 134312093,4 | 30 | 13 |
| 191 | \|A0A151MHG0\|A0A151MHG0_ALLMI Uncharacterized protein OS=Alligator mississippiensis GN=Y1Q_0004565 PE=4 SV=1 | 132538233,8 | 18 | 8 |
| 192 | \|A0A151MMG3\|A0A151MMG3_ALLMI Calreticulin OS=Alligator mississippiensis GN=CALR PE=3 SV=1 | 132078992,0 | 30 | 14 |
| 193 | \|A0A1U8DRB1\|A0A1U8DRB1_ALLSI alpha-2-antiplasmin OS=Alligator sinensis GN=SERPINF2 PE=3 SV=1 | 131886697,6 | 29 | 10 |
| 194 | \|A0A151M916\|A0A151M916_ALLMI Aldehyde dehydrogenase OS=Alligator mississippiensis GN=Y1Q_0001324 PE=3 SV=1 | 131519907,1 | 41 | 14 |
| 195 | \|A0A1U7SLU7\|A0A1U7SLU7_ALLSI ATP-citrate synthase OS=Alligator sinensis GN=ACLY PE=3 SV=1 | 131158054,1 | 51 | 28 |
| 196 | \|A0A1U7SGJ7\|A0A1U7SGJ7_ALLSI eukaryotic initiation factor 4A-I OS=Alligator sinensis GN=EIF4A1 PE=3 SV=1 | 130033071,1 | 36 | 14 |
| 197 | \|A0A1U7SVD9\|A0A1U7SVD9_ALLSI Eukaryotic translation initiation factor 5A OS=Alligator sinensis GN=EIF5A PE=3 SV=1 | 129940296,1 | 33 | 10 |
| 198 | \|A0A151NDX3\|A0A151NDX3_ALLMI Sialic acid synthase OS=Alligator mississippiensis GN=NANS PE=4 SV=1 | 129301789,8 | 28 | 10 |
| 199 | \|A0A1U7S9S5\|A0A1U7S9S5_ALLSI 3-hydroxybutyrate dehydrogenase type 2 OS=Alligator sinensis GN=BDH2 PE=4 SV=1 | 128161498,7 | 13 | 7 |
| 200 | \|A0A1U8DFV4\|A0A1U8DFV4_ALLSI staphylococcal nuclease domain-containing protein 1 OS=Alligator sinensis GN=SND1 PE=4 SV=1 | 127029348,9 | 22 | 11 |
| 201 | \|A0A1U7SFM2\|A0A1U7SFM2_ALLSI Profilin OS=Alligator sinensis GN=PFN1 PE=3 SV=1 | 126188967,8 | 25 | 8 |
| 202 | \|A0A1U7RXJ0\|A0A1U7RXJ0_ALLSI 40S ribosomal protein S3 OS=Alligator sinensis GN=RPS3 PE=3 SV=1 | 125870157,6 | 34 | 16 |
| 203 | \|A0A151PIT0\|A0A151PIT0_ALLMI Complement factor B OS=Alligator mississippiensis GN=CFB PE=4 SV=1 | 124583740,8 | 16 | 5 |
| 204 | \|A0A1U8DIP0\|A0A1U8DIP0_ALLSI cytosolic non-specific dipeptidase OS=Alligator sinensis GN=LOC102368140 PE=4 SV=1 | 121316507,5 | 58 | 24 |
| 205 | \|A0A151MEP3\|A0A151MEP3_ALLMI Alpha/beta hydrolase domain-containing protein 14B OS=Alligator mississippiensis GN=ABHD14B PE=4 SV=1 | 121280423,9 | 15 | 8 |
| 206 | \|A0A151NM15\|A0A151NM15_ALLMI Proteasome activator complex subunit 1 OS=Alligator mississippiensis GN=PSME1 PE=4 SV=1 | 119065647,4 | 31 | 7 |
| 207 | \|A0A151N0I5\|A0A151N0I5_ALLMI Eukaryotic translation initiation factor 2 subunit 3 OS=Alligator mississippiensis GN=EIF2S3 PE=4 SV=1 | 118488364,7 | 32 | 14 |
| 208 | \|A0A151MGP3\|A0A151MGP3_ALLMI Actin-related protein 2 OS=Alligator mississippiensis GN=ACTR2 PE=3 SV=1 | 117701041,8 | 34 | 17 |
| 209 | \|A0A151NQV3\|A0A151NQV3_ALLMI Ribosomal RNA processing 1-like protein B OS=Alligator mississippiensis GN=RRP1B PE=4 SV=1 | 117160246,1 | 31 | 11 |
| 210 | \|A0A1U7SVZ4\|A0A1U7SVZ4_ALLSI endonuclease domain-containing 1 protein-like OS=Alligator sinensis GN=LOC102383670 PE=4 SV=1 | 114931585,5 | 26 | 6 |
| 211 | \|A0A151N034\|A0A151N034_ALLMI Uncharacterized protein OS=Alligator mississippiensis GN=Y1Q_0022428 PE=3 SV=1 | 112918012,2 | 27 | 13 |
| 212 | \|A0A151M2D6\|A0A151M2D6_ALLMI T-complex protein 1 subunit alpha OS=Alligator mississippiensis GN=TCP1 PE=3 SV=1 | 112520158,4 | 28 | 12 |
| 213 | \|A0A1U7RA06\|A0A1U7RA06_ALLSI uncharacterized protein LOC102376444 OS=Alligator sinensis GN=LOC102376444 PE=4 SV=1 | 112232428,4 | 7 | 2 |
| 214 | \|A0A151MGL9\|A0A151MGL9_ALLMI UTP--glucose-1-phosphate uridylyltransferase isoform A OS=Alligator mississippiensis GN=UGP2 PE=4 SV=1 | 111361551,9 | 32 | 15 |
| 215 | \|A0A151MZE8\|A0A151MZE8_ALLMI Proliferation-associated protein 2G4 OS=Alligator mississippiensis GN=PA2G4 PE=4 SV=1 | 111235566,7 | 40 | 20 |
| 216 | \|A0A1U8DHD8\|A0A1U8DHD8_ALLSI LOW QUALITY PROTEIN: junctional adhesion molecule A-like OS=Alligator sinensis GN=LOC102383480 PE=4 SV=1 | 110666515,4 | 10 | 3 |
| 217 | \|A0A151PIP5\|A0A151PIP5_ALLMI Cytosolic 5'-nucleotidase 1B isoform B OS=Alligator mississippiensis GN=NT5C1B-1 PE=4 SV=1 | 110441526,3 | 28 | 9 |
| 218 | \|A0A151N1C0\|A0A151N1C0_ALLMI Serine/threonine-protein kinase SMG1 isoform A OS=Alligator mississippiensis GN=SMG1 PE=3 SV=1 | 110037038,2 | 7 | 3 |
| 219 | \|A0A151NZD8\|A0A151NZD8_ALLMI Proteasome subunit alpha type OS=Alligator mississippiensis GN=PSMA7 PE=3 SV=1 | 107199335,8 | 33 | 15 |
| 220 | \|A0A151M7G3\|A0A151M7G3_ALLMI Protein S100 OS=Alligator mississippiensis GN=S100A11 PE=3 SV=1 | 106452459,0 | 6 | 2 |
| 221 | \|A0A151P5P7\|A0A151P5P7_ALLMI Uncharacterized protein OS=Alligator mississippiensis GN=Y1Q_0012141 PE=4 SV=1 | 106345366,5 | 19 | 8 |
| 222 | \|A0A151N2J0\|A0A151N2J0_ALLMI cAMP-dependent protein kinase type I-alpha regulatory subunit OS=Alligator mississippiensis GN=PRKAR1A PE=4 SV=1 | 106152965,3 | 14 | 6 |
| 223 | \|A0A151NHC2\|A0A151NHC2_ALLMI 26S protease regulatory subunit 6B OS=Alligator mississippiensis GN=PSMC4 PE=3 SV=1 | 106094847,3 | 21 | 11 |
| 224 | \|A0A151MK66\|A0A151MK66_ALLMI Uncharacterized protein OS=Alligator mississippiensis GN=Y1Q_0023736 PE=3 SV=1 | 105984197,9 | 34 | 20 |
| 225 | \|A0A1U8DJI1\|A0A1U8DJI1_ALLSI vegetative cell wall protein gp1-like isoform X1 OS=Alligator sinensis GN=LOC102385071 PE=4 SV=1 | 105368547,3 | 25 | 5 |
| 226 | \|A0A1U7RPH9\|A0A1U7RPH9_ALLSI T-complex protein 1 subunit beta OS=Alligator sinensis GN=CCT2 PE=3 SV=1 | 104551930,0 | 31 | 17 |
| 227 | \|A0A151P6R3\|A0A151P6R3_ALLMI Uncharacterized protein OS=Alligator mississippiensis GN=Y1Q_0016874 PE=4 SV=1 | 103977732,9 | 22 | 12 |
| 228 | \|A0A151NQ86\|A0A151NQ86_ALLMI 26S protease regulatory subunit 7 OS=Alligator mississippiensis GN=PSMC2 PE=3 SV=1 | 103545047,9 | 35 | 18 |
| 229 | \|A0A1U7S8G6\|A0A1U7S8G6_ALLSI uromodulin OS=Alligator sinensis GN=UMOD PE=4 SV=1 | 103150507,6 | 21 | 7 |
| 230 | \|A0A151NJ08\|A0A151NJ08_ALLMI Fructose-bisphosphate aldolase OS=Alligator mississippiensis GN=Y1Q_0006767 PE=3 SV=1 | 103071479,5 | 15 | 6 |
| 231 | \|A0A151N2D1\|A0A151N2D1_ALLMI Beta-2-glycoprotein 1 OS=Alligator mississippiensis GN=APOH PE=4 SV=1 | 102467409,7 | 15 | 5 |
| 232 | \|A0A151MIZ9\|A0A151MIZ9_ALLMI Bifunctional 3'-phosphoadenosine 5'-phosphosulfate synthase 1 OS=Alligator mississippiensis GN=PAPSS1 PE=3 SV=1 | 102091148,2 | 22 | 11 |
| 233 | \|A0A1U7RQQ3\|A0A1U7RQQ3_ALLSI rho GDP-dissociation inhibitor 2 OS=Alligator sinensis GN=ARHGDIB PE=4 SV=1 | 102052146,4 | 25 | 11 |
| 234 | \|A0A151M1V3\|A0A151M1V3_ALLMI Proteasome subunit alpha type OS=Alligator mississippiensis GN=PSMA3 PE=3 SV=1 | 101784431,7 | 29 | 11 |
| 235 | \|A0A1U8DMW0\|A0A1U8DMW0_ALLSI high mobility group protein B1 OS=Alligator sinensis GN=HMGB1 PE=4 SV=1 | 101542588,4 | 32 | 11 |
| 236 | \|A0A1U7RKS9\|A0A1U7RKS9_ALLSI prolyl endopeptidase OS=Alligator sinensis GN=PREP PE=4 SV=1 | 101404192,6 | 34 | 20 |
| 237 | \|A0A1U7S1U4\|A0A1U7S1U4_ALLSI aminopeptidase B OS=Alligator sinensis GN=RNPEP PE=4 SV=1 | 100606069,8 | 48 | 19 |
| 238 | \|A0A1U8CVY1\|A0A1U8CVY1_ALLSI LOW QUALITY PROTEIN: asparagine--tRNA ligase, cytoplasmic OS=Alligator sinensis GN=NARS PE=4 SV=1 | 98791316,6 | 27 | 13 |
| 239 | \|A0A151MZ64\|A0A151MZ64_ALLMI Uncharacterized protein OS=Alligator mississippiensis GN=Y1Q_0005956 PE=4 SV=1 | 97513889,1 | 17 | 5 |
| 240 | \|A0A1U7RWU7\|A0A1U7RWU7_ALLSI poly(RC)-binding protein 2 isoform X2 OS=Alligator sinensis GN=PCBP2 PE=4 SV=1 | 97419583,9 | 21 | 10 |
| 241 | \|A0A151P6X1\|A0A151P6X1_ALLMI Uncharacterized protein OS=Alligator mississippiensis GN=Y1Q_0006982 PE=4 SV=1 | 97384670,1 | 5 | 2 |
| 242 | \|A0A151N4N6\|A0A151N4N6_ALLMI 14-3-3 protein eta OS=Alligator mississippiensis GN=YWHAH PE=3 SV=1 | 97314920,2 | 16 | 6 |
| 243 | \|A0A151PFF9\|A0A151PFF9_ALLMI Cullin-associated NEDD8-dissociated protein 1 OS=Alligator mississippiensis GN=CAND1 PE=4 SV=1 | 96442155,1 | 42 | 23 |
| 244 | \|A0A151NMH1\|A0A151NMH1_ALLMI Alcohol dehydrogenase class-3 OS=Alligator mississippiensis GN=ADH5 PE=4 SV=1 | 96333618,7 | 47 | 18 |
| 245 | \|A0A151N6Y0\|A0A151N6Y0_ALLMI Uncharacterized protein OS=Alligator mississippiensis GN=Y1Q_0020436 PE=4 SV=1 | 94677403,1 | 40 | 28 |
| 246 | \|A0A1U8DMQ6\|A0A1U8DMQ6_ALLSI protein deglycase DJ-1 OS=Alligator sinensis GN=PARK7 PE=4 SV=1 | 94213692,0 | 25 | 9 |
| 247 | \|A0A1U8CWH2\|A0A1U8CWH2_ALLSI perilipin-3 isoform X1 OS=Alligator sinensis GN=LOC102369356 PE=4 SV=1 | 94008848,7 | 23 | 13 |
| 248 | \|A0A151NE79\|A0A151NE79_ALLMI UV excision repair RAD23-like protein B OS=Alligator mississippiensis GN=RAD23B PE=4 SV=1 | 93009014,3 | 11 | 4 |
| 249 | \|A0A151M0V0\|A0A151M0V0_ALLMI Acyl-CoA-binding protein OS=Alligator mississippiensis GN=DBI PE=4 SV=1 | 92890685,1 | 19 | 6 |
| 250 | \|A0A1U7RAN3\|A0A1U7RAN3_ALLSI latexin OS=Alligator sinensis GN=LXN PE=4 SV=1 | 92716380,2 | 7 | 1 |
| 251 | \|A0A1U7S342\|A0A1U7S342_ALLSI SH3 domain-binding glutamic acid-rich-like protein OS=Alligator sinensis GN=SH3BGRL PE=3 SV=1 | 92406097,2 | 33 | 12 |
| 252 | \|A0A1U7S0C0\|A0A1U7S0C0_ALLSI complement C3 OS=Alligator sinensis GN=C3 PE=4 SV=1 | 91738413,7 | 10 | 3 |
| 253 | \|A0A151M599\|A0A151M599_ALLMI Proteasome subunit beta OS=Alligator mississippiensis GN=PSMB4 PE=3 SV=1 | 91228483,6 | 26 | 10 |
| 254 | \|A0A151N258\|A0A151N258_ALLMI 40S ribosomal protein S2 OS=Alligator mississippiensis GN=RPS2 PE=3 SV=1 | 90802896,2 | 21 | 7 |
| 255 | \|A0A1U8DNZ2\|A0A1U8DNZ2_ALLSI 78 kDa glucose-regulated protein OS=Alligator sinensis GN=HSPA5 PE=3 SV=1 | 90534106,1 | 27 | 15 |
| 256 | \|A0A1U7RQ17\|A0A1U7RQ17_ALLSI Proteasome subunit alpha type OS=Alligator sinensis GN=PSMA4 PE=3 SV=1 | 90250152,5 | 31 | 11 |
| 257 | \|A0A1U7RV10\|A0A1U7RV10_ALLSI alpha-aminoadipic semialdehyde dehydrogenase OS=Alligator sinensis GN=ALDH7A1 PE=3 SV=1 | 89422802,1 | 29 | 16 |
| 258 | \|A0A151P5J8\|A0A151P5J8_ALLMI LIM and SH3 domain protein 1 isoform B OS=Alligator mississippiensis GN=LASP1-1 PE=4 SV=1 | 89414123,0 | 15 | 5 |
| 259 | \|A0A151PBB7\|A0A151PBB7_ALLMI 40S ribosomal protein S3a OS=Alligator mississippiensis GN=RPS3A PE=3 SV=1 | 89068660,2 | 20 | 10 |
| 260 | \|A0A151MLG5\|A0A151MLG5_ALLMI Acetyl-coenzyme A synthetase, cytoplasmic isoform A OS=Alligator mississippiensis GN=ACSS2 PE=4 SV=1 | 88726486,5 | 18 | 9 |
| 261 | \|A0A151NS50\|A0A151NS50_ALLMI Uncharacterized protein OS=Alligator mississippiensis GN=Y1Q_0018735 PE=3 SV=1 | 88469289,7 | 32 | 16 |
| 262 | \|A0A151M589\|A0A151M589_ALLMI Selenium-binding protein 1 OS=Alligator mississippiensis GN=SELENBP1 PE=4 SV=1 | 87891815,8 | 16 | 6 |
| 263 | \|A0A1U7S7F2\|A0A1U7S7F2_ALLSI Protein S100 OS=Alligator sinensis GN=S100A11 PE=3 SV=1 | 86106095,5 | 12 | 3 |
| 264 | \|A0A151MJ91\|A0A151MJ91_ALLMI Protein-arginine deiminase type-3 OS=Alligator mississippiensis GN=PADI3 PE=4 SV=1 | 86027621,1 | 13 | 3 |
| 265 | \|A0A151M8E4\|A0A151M8E4_ALLMI Osteoclast-stimulating factor 1 OS=Alligator mississippiensis GN=OSTF1 PE=4 SV=1 | 85786088,7 | 24 | 9 |
| 266 | \|A0A151N9J3\|A0A151N9J3_ALLMI Proteasome subunit alpha type OS=Alligator mississippiensis GN=PSMA5 PE=3 SV=1 | 85068697,9 | 27 | 12 |
| 267 | \|A0A151P1L2\|A0A151P1L2_ALLMI Lysine--tRNA ligase OS=Alligator mississippiensis GN=KARS PE=3 SV=1 | 84940918,9 | 27 | 11 |
| 268 | \|A0A151PIZ6\|A0A151PIZ6_ALLMI Protein S100-A16 OS=Alligator mississippiensis GN=S100A16 PE=4 SV=1 | 82686746,2 | 11 | 4 |
| 269 | \|A0A151N708\|A0A151N708_ALLMI Xaa-Pro dipeptidase OS=Alligator mississippiensis GN=PEPD PE=3 SV=1 | 82111352,4 | 24 | 11 |
| 270 | \|A0A151PDY8\|A0A151PDY8_ALLMI 26S protease regulatory subunit 10B OS=Alligator mississippiensis GN=PSMC6 PE=3 SV=1 | 81695800,8 | 13 | 6 |
| 271 | \|A0A151N5B9\|A0A151N5B9_ALLMI 14-3-3 protein gamma OS=Alligator mississippiensis GN=YWHAG PE=3 SV=1 | 81403908,5 | 18 | 8 |
| 272 | \|A0A151MB44\|A0A151MB44_ALLMI Eukaryotic translation initiation factor 2 subunit 1 OS=Alligator mississippiensis GN=EIF2S1 PE=4 SV=1 | 81335801,0 | 13 | 9 |
| 273 | \|A0A1U7SC06\|A0A1U7SC06_ALLSI vigilin OS=Alligator sinensis GN=HDLBP PE=4 SV=1 | 81231133,6 | 31 | 15 |
| 274 | \|A0A151NKD7\|A0A151NKD7_ALLMI Proteasome subunit alpha type OS=Alligator mississippiensis GN=PSMA6 PE=3 SV=1 | 81075776,4 | 18 | 9 |
| 275 | \|A0A1U8CTL9\|A0A1U8CTL9_ALLSI 4-trimethylaminobutyraldehyde dehydrogenase OS=Alligator sinensis GN=ALDH9A1 PE=3 SV=1 | 81060521,1 | 24 | 11 |
| 276 | \|A0A151LYU3\|A0A151LYU3_ALLMI Lumican OS=Alligator mississippiensis GN=LUM PE=4 SV=1 | 81020411,5 | 19 | 7 |
| 277 | \|A0A151P2X6\|A0A151P2X6_ALLMI Lactoylglutathione lyase OS=Alligator mississippiensis GN=GLO1 PE=3 SV=1 | 80821832,3 | 25 | 9 |
| 278 | \|A0A1U8DHY7\|A0A1U8DHY7_ALLSI Spectrin beta chain OS=Alligator sinensis GN=SPTBN2 PE=3 SV=1 | 80749499,4 | 19 | 14 |
| 279 | \|A0A151NRW5\|A0A151NRW5_ALLMI Acetoacetyl-CoA synthetase OS=Alligator mississippiensis GN=AACS PE=4 SV=1 | 80160058,8 | 33 | 17 |
| 280 | \|A0A151M7Z5\|A0A151M7Z5_ALLMI Histidine triad nucleotide-binding protein 1 OS=Alligator mississippiensis GN=HINT1 PE=4 SV=1 | 79964761,1 | 12 | 5 |
| 281 | \|A0A151M033\|A0A151M033_ALLMI Obg-like ATPase 1 OS=Alligator mississippiensis GN=OLA1 PE=3 SV=1 | 79604105,8 | 17 | 10 |
| 282 | \|A0A151NWL1\|A0A151NWL1_ALLMI Mucin-16 OS=Alligator mississippiensis GN=MUC16 PE=4 SV=1 | 79500717,9 | 27 | 11 |
| 283 | \|A0A151MAB6\|A0A151MAB6_ALLMI Tropomyosin alpha-1 chain isoform D OS=Alligator mississippiensis GN=TPM1-1 PE=3 SV=1 | 78228373,8 | 10 | 5 |
| 284 | \|A0A1U8D7H2\|A0A1U8D7H2_ALLSI Sulfotransferase OS=Alligator sinensis GN=LOC102374654 PE=3 SV=1 | 77448804,1 | 15 | 7 |
| 285 | \|A0A1U8DX32\|A0A1U8DX32_ALLSI chloride intracellular channel protein 1 OS=Alligator sinensis GN=CLIC1 PE=4 SV=1 | 77417200,2 | 17 | 9 |
| 286 | \|A0A1U7SBC6\|A0A1U7SBC6_ALLSI charged multivesicular body protein 2a OS=Alligator sinensis GN=CHMP2A PE=3 SV=1 | 77058248,1 | 13 | 6 |
| 287 | \|A0A151MI25\|A0A151MI25_ALLMI Microtubule-associated protein RP/EB family member 1 OS=Alligator mississippiensis GN=MAPRE1 PE=4 SV=1 | 76544336,1 | 14 | 6 |
| 288 | \|A0A1U8DHF0\|A0A1U8DHF0_ALLSI L-lactate dehydrogenase OS=Alligator sinensis GN=LDHB PE=3 SV=1 | 76060980,5 | 3 | 1 |
| 289 | \|A0A1U8E129\|A0A1U8E129_ALLSI alpha-2-macroglobulin-like protein 1 OS=Alligator sinensis GN=LOC102379338 PE=4 SV=1 | 76028273,0 | 26 | 10 |
| 290 | \|A0A151MYV2\|A0A151MYV2_ALLMI Uncharacterized protein OS=Alligator mississippiensis GN=Y1Q_0005949 PE=4 SV=1 | 74513377,7 | 8 | 3 |
| 291 | \|A0A151MMY5\|A0A151MMY5_ALLMI Tyrosine--tRNA ligase OS=Alligator mississippiensis GN=YARS PE=3 SV=1 | 74453692,2 | 23 | 11 |
| 292 | \|A0A151N8U8\|A0A151N8U8_ALLMI 40S ribosomal protein S7 OS=Alligator mississippiensis GN=RPS7 PE=4 SV=1 | 74259995,1 | 11 | 5 |
| 293 | \|A0A151NVZ2\|A0A151NVZ2_ALLMI Xaa-Pro aminopeptidase 1 isoform A OS=Alligator mississippiensis GN=XPNPEP1 PE=3 SV=1 | 74048719,4 | 18 | 11 |
| 294 | \|A0A151MT59\|A0A151MT59_ALLMI Uncharacterized protein OS=Alligator mississippiensis GN=Y1Q_0005249 PE=4 SV=1 | 73874500,8 | 6 | 1 |
| 295 | \|A0A1U8D6W1\|A0A1U8D6W1_ALLSI LOW QUALITY PROTEIN: deleted in malignant brain tumors 1 protein OS=Alligator sinensis GN=DMBT1 PE=4 SV=1 | 72882698,1 | 16 | 9 |
| 296 | \|A0A151M9Z9\|A0A151M9Z9_ALLMI Mannose-6-phosphate isomerase OS=Alligator mississippiensis GN=MPI PE=3 SV=1 | 72147063,8 | 20 | 8 |
| 297 | \|A0A151MCT8\|A0A151MCT8_ALLMI Ubiquitinyl hydrolase 1 OS=Alligator mississippiensis GN=Y1Q_0002914 PE=3 SV=1 | 71782157,2 | 36 | 18 |
| 298 | \|A0A151N6D4\|A0A151N6D4_ALLMI Protein NLRC3 OS=Alligator mississippiensis GN=NLRC3 PE=4 SV=1 | 71605212,0 | 11 | 4 |
| 299 | \|A0A1U7RVV3\|A0A1U7RVV3_ALLSI 60S ribosomal protein L18 OS=Alligator sinensis GN=RPL18 PE=4 SV=1 | 71524313,3 | 12 | 6 |
| 300 | \|A0A151MYU6\|A0A151MYU6_ALLMI Protein AHNAK2 OS=Alligator mississippiensis GN=AHNAK2L PE=4 SV=1 | 71326779,2 | 25 | 16 |
| 301 | \|A0A151P356\|A0A151P356_ALLMI Proteasome endopeptidase complex OS=Alligator mississippiensis GN=Y1Q_0017503 PE=3 SV=1 | 71216325,3 | 25 | 8 |
| 302 | \|A0A1U8DRL5\|A0A1U8DRL5_ALLSI retinoic acid receptor responder protein 1 OS=Alligator sinensis GN=RARRES1 PE=4 SV=1 | 70755156,2 | 12 | 8 |
| 303 | \|A0A151NHN3\|A0A151NHN3_ALLMI Uncharacterized protein OS=Alligator mississippiensis GN=Y1Q_0024073 PE=3 SV=1 | 70529825,2 | 20 | 10 |
| 304 | \|A0A151P255\|A0A151P255_ALLMI Biliverdin reductase A OS=Alligator mississippiensis GN=BLVRA PE=4 SV=1 | 70488315,2 | 19 | 8 |
| 305 | \|A0A151MMY8\|A0A151MMY8_ALLMI Spectrin beta chain OS=Alligator mississippiensis GN=Y1Q_0012258 PE=3 SV=1 | 70281291,2 | 8 | 3 |
| 306 | \|A0A1U7SQS6\|A0A1U7SQS6_ALLSI Sulfhydryl oxidase OS=Alligator sinensis GN=QSOX1 PE=4 SV=1 | 70115058,3 | 15 | 7 |
| 307 | \|A0A151MYX4\|A0A151MYX4_ALLMI Uncharacterized protein OS=Alligator mississippiensis GN=Y1Q_0017648 PE=4 SV=1 | 70112253,7 | 14 | 6 |
| 308 | \|A0A151MGY7\|A0A151MGY7_ALLMI T-complex protein 1 subunit delta OS=Alligator mississippiensis GN=CCT4 PE=3 SV=1 | 69838354,1 | 40 | 18 |
| 309 | \|A0A1U7RLD9\|A0A1U7RLD9_ALLSI glycogen debranching enzyme isoform X2 OS=Alligator sinensis GN=AGL PE=4 SV=1 | 69812377,1 | 22 | 13 |
| 310 | \|A0A1U7R1L7\|A0A1U7R1L7_ALLSI LOW QUALITY PROTEIN: catenin alpha-1 OS=Alligator sinensis GN=CTNNA1 PE=4 SV=1 | 69660726,3 | 20 | 16 |
| 311 | \|A0A151N4M1\|A0A151N4M1_ALLMI 60S acidic ribosomal protein P0 OS=Alligator mississippiensis GN=RPLP0 PE=4 SV=1 | 69586105,7 | 23 | 9 |
| 312 | \|A0A1U7SI23\|A0A1U7SI23_ALLSI ubiquitin-like domain-containing CTD phosphatase 1 OS=Alligator sinensis GN=UBLCP1 PE=4 SV=1 | 69334560,0 | 8 | 5 |
| 313 | \|A0A151ND33\|A0A151ND33_ALLMI T-complex protein 1 subunit epsilon OS=Alligator mississippiensis GN=CCT5 PE=3 SV=1 | 69007603,1 | 30 | 14 |
| 314 | \|A0A151MYS3\|A0A151MYS3_ALLMI Uncharacterized protein OS=Alligator mississippiensis GN=Y1Q_0005954 PE=4 SV=1 | 68963505,2 | 17 | 6 |
| 315 | \|A0A151NLJ0\|A0A151NLJ0_ALLMI Uncharacterized protein OS=Alligator mississippiensis GN=Y1Q_0021989 PE=4 SV=1 | 68879964,4 | 20 | 13 |
| 316 | \|A0A151NM44\|A0A151NM44_ALLMI Uncharacterized protein OS=Alligator mississippiensis GN=Y1Q_0015778 PE=4 SV=1 | 68708592,0 | 37 | 20 |
| 317 | \|A0A151MQE1\|A0A151MQE1_ALLMI Anterior gradient 2-like protein OS=Alligator mississippiensis GN=AGR2 PE=4 SV=1 | 68588585,2 | 13 | 3 |
| 318 | \|A0A151NR50\|A0A151NR50_ALLMI ATP-dependent RNA helicase DDX3X isoform A OS=Alligator mississippiensis GN=DDX3X PE=3 SV=1 | 68108476,9 | 18 | 11 |
| 319 | \|A0A1U7S780\|A0A1U7S780_ALLSI alpha-1-antitrypsin-like OS=Alligator sinensis GN=LOC102371045 PE=3 SV=1 | 67991484,5 | 21 | 9 |
| 320 | \|A0A151MB02\|A0A151MB02_ALLMI Uncharacterized protein OS=Alligator mississippiensis GN=Y1Q_0000417 PE=4 SV=1 | 67613256,2 | 25 | 13 |
| 321 | \|A0A151NB24\|A0A151NB24_ALLMI Latexin OS=Alligator mississippiensis GN=LXN PE=4 SV=1 | 67409638,2 | 13 | 5 |
| 322 | \|A0A151NWR9\|A0A151NWR9_ALLMI Epithelial cell adhesion molecule OS=Alligator mississippiensis GN=EPCAM PE=4 SV=1 | 67394614,9 | 16 | 7 |
| 323 | \|A0A151MFZ3\|A0A151MFZ3_ALLMI Uncharacterized protein OS=Alligator mississippiensis GN=Y1Q_0005805 PE=4 SV=1 | 67392110,9 | 24 | 14 |
| 324 | \|A0A151NNY8\|A0A151NNY8_ALLMI Cofilin-1 OS=Alligator mississippiensis GN=CFL1 PE=3 SV=1 | 67366208,0 | 15 | 5 |
| 325 | \|U3U7A5\|U3U7A5_CAICR Proteasome subunit beta type (Fragment) OS=Caiman crocodilus GN=PSMB8 PE=2 SV=1 | 66552746,6 | 13 | 6 |
| 326 | \|A0A1U7STC9\|A0A1U7STC9_ALLSI V-type proton ATPase catalytic subunit A OS=Alligator sinensis GN=ATP6V1A PE=3 SV=1 | 66533810,1 | 26 | 17 |
| 327 | \|A0A1U7SCX3\|A0A1U7SCX3_ALLSI Tubulin alpha chain OS=Alligator sinensis GN=TUBA4A PE=3 SV=1 | 66453896,2 | 18 | 5 |
| 328 | \|A0A151M678\|A0A151M678_ALLMI Kininogen-1 OS=Alligator mississippiensis GN=KNG1 PE=4 SV=1 | 66060338,5 | 17 | 7 |
| 329 | \|A0A1U7SNP0\|A0A1U7SNP0_ALLSI apoptosis-associated speck-like protein containing a CARD OS=Alligator sinensis GN=LOC102373125 PE=4 SV=1 | 66036881,9 | 9 | 3 |
| 330 | \|A0A151N5P4\|A0A151N5P4_ALLMI T-complex protein 1 subunit zeta OS=Alligator mississippiensis GN=CCT6A PE=3 SV=1 | 65567133,4 | 21 | 11 |
| 331 | \|A9CPG5\|A9CPG5_ALLMI Heat shock protein 108 OS=Alligator mississippiensis GN=HSP108 PE=2 SV=1 | 65456307,1 | 24 | 13 |
| 332 | \|A0A1U8D227\|A0A1U8D227_ALLSI bifunctional UDP-N-acetylglucosamine 2-epimerase/N-acetylmannosamine kinase isoform X1 OS=Alligator sinensis GN=GNE PE=4 SV=1 | 64330173,4 | 23 | 12 |
| 333 | \|A0A1U7S7K3\|A0A1U7S7K3_ALLSI Prothrombin OS=Alligator sinensis GN=F2 PE=3 SV=1 | 64228919,7 | 21 | 10 |
| 334 | \|A0A151M606\|A0A151M606_ALLMI Coatomer subunit beta' OS=Alligator mississippiensis GN=COPB2 PE=3 SV=1 | 64155566,5 | 28 | 17 |
| 335 | \|A0A151N3C0\|A0A151N3C0_ALLMI Alpha-1,4 glucan phosphorylase OS=Alligator mississippiensis GN=Y1Q_0002565 PE=3 SV=1 | 63835467,4 | 8 | 3 |
| 336 | \|A0A151NSU6\|A0A151NSU6_ALLMI 60S ribosomal protein L6 OS=Alligator mississippiensis GN=RPL6 PE=3 SV=1 | 63670445,6 | 17 | 7 |
| 337 | \|A0A1U7S5V2\|A0A1U7S5V2_ALLSI Glutamine synthetase OS=Alligator sinensis GN=LOC102376806 PE=3 SV=1 | 63450018,3 | 12 | 6 |
| 338 | \|A0A1U7SHM5\|A0A1U7SHM5_ALLSI 40S ribosomal protein SA OS=Alligator sinensis GN=RPSA PE=3 SV=1 | 62735878,3 | 16 | 9 |
| 339 | \|A0A151MGG2\|A0A151MGG2_ALLMI Ubiquitin-40S ribosomal protein S27a OS=Alligator mississippiensis GN=RPS27A PE=4 SV=1 | 62342851,0 | 13 | 6 |
| 340 | \|A0A151PC95\|A0A151PC95_ALLMI Keratin, type I cytoskeletal 20 OS=Alligator mississippiensis GN=Y1Q_0018455 PE=3 SV=1 | 62113265,4 | 14 | 4 |
| 341 | \|A0A151MTN3\|A0A151MTN3_ALLMI F-actin-capping protein subunit alpha-1 OS=Alligator mississippiensis GN=CAPZA1-2 PE=3 SV=1 | 61739576,5 | 24 | 9 |
| 342 | \|A0A151MHP4\|A0A151MHP4_ALLMI Translationally-controlled tumor protein OS=Alligator mississippiensis GN=TPT1 PE=3 SV=1 | 61402037,7 | 12 | 3 |
| 343 | \|A0A1U7SEX2\|A0A1U7SEX2_ALLSI BPI fold-containing family B member 3 OS=Alligator sinensis GN=LOC102376719 PE=4 SV=1 | 61108188,1 | 10 | 3 |
| 344 | \|A0A1U7RYI4\|A0A1U7RYI4_ALLSI 26S proteasome non-ATPase regulatory subunit 1 OS=Alligator sinensis GN=PSMD1 PE=4 SV=1 | 61069635,1 | 30 | 17 |
| 345 | \|A0A151MMN6\|A0A151MMN6_ALLMI UDP-glucose 4-epimerase OS=Alligator mississippiensis GN=GALE PE=3 SV=1 | 60724302,3 | 16 | 7 |
| 346 | \|A0A151LZP3\|A0A151LZP3_ALLMI Platelet-activating factor acetylhydrolase IB subunit beta OS=Alligator mississippiensis GN=PAFAH1B2 PE=4 SV=1 | 60705001,7 | 16 | 6 |
| 347 | \|A0A1U7RZ21\|A0A1U7RZ21_ALLSI ubiquitin-conjugating enzyme E2 L3 OS=Alligator sinensis GN=UBE2L3 PE=3 SV=1 | 60624185,2 | 14 | 7 |
| 348 | \|A0A151P114\|A0A151P114_ALLMI Uridine phosphorylase OS=Alligator mississippiensis GN=UPP1 PE=3 SV=1 | 60624007,4 | 17 | 9 |
| 349 | \|A0A151P644\|A0A151P644_ALLMI 40S ribosomal protein S9 OS=Alligator mississippiensis GN=RPS9 PE=3 SV=1 | 60368611,3 | 16 | 7 |
| 350 | \|A0A151NW17\|A0A151NW17_ALLMI Phosphoglycolate phosphatase OS=Alligator mississippiensis GN=PGP PE=4 SV=1 | 60286126,6 | 17 | 8 |
| 351 | \|A0A151MVY5\|A0A151MVY5_ALLMI V-set domain-containing T-cell activation inhibitor 1-like OS=Alligator mississippiensis GN=Y1Q_0000819 PE=4 SV=1 | 60061256,5 | 8 | 4 |
| 352 | \|A0A151MGM4\|A0A151MGM4_ALLMI Ras-related protein Rab-1A OS=Alligator mississippiensis GN=RAB1A PE=4 SV=1 | 60023300,3 | 23 | 8 |
| 353 | \|A0A151MNM1\|A0A151MNM1_ALLMI Ubiquitin-conjugating enzyme E2 variant 2 OS=Alligator mississippiensis GN=UBE2V2 PE=3 SV=1 | 59996214,6 | 15 | 6 |
| 354 | \|A0A1U7RYL5\|A0A1U7RYL5_ALLSI STE20-like serine/threonine-protein kinase isoform X2 OS=Alligator sinensis GN=SLK PE=4 SV=1 | 59900427,4 | 30 | 18 |
| 355 | \|A0A151PK40\|A0A151PK40_ALLMI Protein NDRG2 isoform B OS=Alligator mississippiensis GN=NDRG2 PE=4 SV=1 | 59521677,1 | 22 | 10 |
| 356 | \|A0A1U7RTS0\|A0A1U7RTS0_ALLSI Annexin OS=Alligator sinensis GN=ANXA3 PE=3 SV=1 | 59519406,7 | 5 | 1 |
| 357 | \|A0A151PBL0\|A0A151PBL0_ALLMI 40S ribosomal protein S8 OS=Alligator mississippiensis GN=RPS8 PE=3 SV=1 | 59518008,7 | 15 | 7 |
| 358 | \|A0A151MHK7\|A0A151MHK7_ALLMI S-formylglutathione hydrolase OS=Alligator mississippiensis GN=ESD PE=3 SV=1 | 58848272,2 | 19 | 7 |
| 359 | \|A0A151MBI4\|A0A151MBI4_ALLMI Src substrate protein p85 isoform D OS=Alligator mississippiensis GN=CTTN PE=4 SV=1 | 58801630,5 | 17 | 8 |
| 360 | \|A0A151NBA5\|A0A151NBA5_ALLMI Glyoxalase domain-containing protein 4 OS=Alligator mississippiensis GN=GLOD4 PE=4 SV=1 | 58746774,8 | 19 | 13 |
| 361 | \|A0A151N1Q2\|A0A151N1Q2_ALLMI Uncharacterized protein OS=Alligator mississippiensis GN=Y1Q_0008358 PE=3 SV=1 | 58488240,3 | 24 | 13 |
| 362 | \|A0A1U8DCA5\|A0A1U8DCA5_ALLSI bifunctional glutamate/proline--tRNA ligase isoform X2 OS=Alligator sinensis GN=EPRS PE=3 SV=1 | 58455908,6 | 19 | 13 |
| 363 | \|A0A151NUW4\|A0A151NUW4_ALLMI Alpha-centractin OS=Alligator mississippiensis GN=ACTR1A PE=3 SV=1 | 58455376,3 | 22 | 9 |
| 364 | \|A0A1U7SDF2\|A0A1U7SDF2_ALLSI putative V-set and immunoglobulin domain-containing-like protein IGHV4OR15-8 OS=Alligator sinensis GN=LOC102384924 PE=4 SV=1 | 58442623,6 | 10 | 3 |
| 365 | \|A0A1U7RMB2\|A0A1U7RMB2_ALLSI AP complex subunit beta OS=Alligator sinensis GN=AP1B1 PE=3 SV=1 | 58414450,5 | 27 | 19 |
| 366 | \|Q1EL67\|Q1EL67_CRONI Ovotransferrin OS=Crocodylus niloticus GN=trfe PE=2 SV=1 | 58327736,7 | 7 | 1 |
| 367 | \|A0A1U7RWH5\|A0A1U7RWH5_ALLSI ubiquitin-conjugating enzyme E2 N OS=Alligator sinensis GN=UBE2N PE=3 SV=1 | 58296506,1 | 17 | 7 |
| 368 | \|A0A151P1G4\|A0A151P1G4_ALLMI NSFL1 cofactor p47 OS=Alligator mississippiensis GN=NSFL1C PE=4 SV=1 | 58279420,9 | 18 | 8 |
| 369 | \|A0A151MQ50\|A0A151MQ50_ALLMI Phosphotriesterase-related protein OS=Alligator mississippiensis GN=PTER PE=3 SV=1 | 58160877,2 | 12 | 6 |
| 370 | \|A0A1U7RSY5\|A0A1U7RSY5_ALLSI dynamin-2 isoform X2 OS=Alligator sinensis GN=DNM2 PE=3 SV=1 | 57913968,6 | 16 | 12 |
| 371 | \|A0A151NFZ0\|A0A151NFZ0_ALLMI Transcription elongation factor B polypeptide 2 OS=Alligator mississippiensis GN=TCEB2 PE=4 SV=1 | 57858278,2 | 16 | 5 |
| 372 | \|A0A151N359\|A0A151N359_ALLMI Glutathione reductase, mitochondrial OS=Alligator mississippiensis GN=GSR PE=3 SV=1 | 57674521,3 | 33 | 16 |
| 373 | \|A0A151PFM8\|A0A151PFM8_ALLMI Coatomer subunit delta OS=Alligator mississippiensis GN=ARCN1 PE=3 SV=1 | 57653412,6 | 18 | 9 |
| 374 | \|A0A1U7RFF6\|A0A1U7RFF6_ALLSI tight junction protein ZO-2 isoform X1 OS=Alligator sinensis GN=TJP2 PE=4 SV=1 | 56725911,9 | 24 | 13 |
| 375 | \|A0A151LYE9\|A0A151LYE9_ALLMI Eukaryotic translation initiation factor 4B OS=Alligator mississippiensis GN=Y1Q_0017731 PE=4 SV=1 | 56627077,8 | 13 | 4 |
| 376 | \|A0A1U8CUG7\|A0A1U8CUG7_ALLSI breast carcinoma-amplified sequence 1 isoform X4 OS=Alligator sinensis GN=BCAS1 PE=4 SV=1 | 56370158,2 | 22 | 12 |
| 377 | \|A0A1U7S632\|A0A1U7S632_ALLSI serpin B6 OS=Alligator sinensis GN=LOC102384945 PE=3 SV=1 | 56101234,8 | 26 | 9 |
| 378 | \|A0A1U7S365\|A0A1U7S365_ALLSI heat shock 70 kDa protein 4 OS=Alligator sinensis GN=HSPA4 PE=3 SV=1 | 55888069,5 | 34 | 21 |
| 379 | \|A0A151NTW1\|A0A151NTW1_ALLMI Far upstream element-binding protein 2 isoform B OS=Alligator mississippiensis GN=KHSRP PE=4 SV=1 | 55807705,7 | 13 | 10 |
| 380 | \|A0A151M6Y2\|A0A151M6Y2_ALLMI Serine/threonine-protein phosphatase OS=Alligator mississippiensis GN=PPP2CA PE=3 SV=1 | 55705832,4 | 30 | 12 |
| 381 | \|A0A1U8D6B5\|A0A1U8D6B5_ALLSI LOW QUALITY PROTEIN: lipopolysaccharide-responsive and beige-like anchor protein OS=Alligator sinensis GN=LRBA PE=4 SV=1 | 55627563,9 | 28 | 16 |
| 382 | \|A0A151P0U5\|A0A151P0U5_ALLMI Complement factor D OS=Alligator mississippiensis GN=CFD PE=3 SV=1 | 55563390,1 | 16 | 6 |
| 383 | \|A0A151LZM8\|A0A151LZM8_ALLMI Heterogeneous nuclear ribonucleoprotein U-like protein 2 OS=Alligator mississippiensis GN=HNRNPUL2 PE=4 SV=1 | 55115129,8 | 14 | 10 |
| 384 | \|A0A1U8DCL6\|A0A1U8DCL6_ALLSI soluble scavenger receptor cysteine-rich domain-containing protein SSC5D OS=Alligator sinensis GN=SSC5D PE=4 SV=1 | 55069453,7 | 7 | 6 |
| 385 | \|A0A1U7S992\|A0A1U7S992_ALLSI heterogeneous nuclear ribonucleoproteins A2/B1 OS=Alligator sinensis GN=HNRNPA2B1 PE=4 SV=1 | 55062417,5 | 20 | 12 |
| 386 | \|A0A151MJ73\|A0A151MJ73_ALLMI Aflatoxin B1 aldehyde reductase member 2 OS=Alligator mississippiensis GN=AKR7A2 PE=4 SV=1 | 55008334,1 | 15 | 7 |
| 387 | \|A0A151MJY4\|A0A151MJY4_ALLMI Ras-related protein Rab-7a OS=Alligator mississippiensis GN=RAB7A PE=4 SV=1 | 54997151,0 | 11 | 5 |
| 388 | \|A0A1U7RFC0\|A0A1U7RFC0_ALLSI Proteasome subunit beta type OS=Alligator sinensis GN=PSMB3 PE=3 SV=1 | 54880408,6 | 13 | 6 |
| 389 | \|A0A151P336\|A0A151P336_ALLMI Serine/threonine-protein phosphatase OS=Alligator mississippiensis GN=PPP1CA PE=3 SV=1 | 54778680,8 | 17 | 9 |
| 390 | \|A0A151ME84\|A0A151ME84_ALLMI Carbonyl reductase [NADPH] 1 OS=Alligator mississippiensis GN=CBR1 PE=3 SV=1 | 54659196,0 | 26 | 11 |
| 391 | \|A0A151NYX2\|A0A151NYX2_ALLMI Ras-related protein Rab-11A-like OS=Alligator mississippiensis GN=RAB2BL PE=4 SV=1 | 53656428,1 | 16 | 5 |
| 392 | \|A0A1U8DEX9\|A0A1U8DEX9_ALLSI elongation factor 1-delta isoform X3 OS=Alligator sinensis GN=EEF1D PE=3 SV=1 | 53293565,0 | 18 | 8 |
| 393 | \|A0A1U8CY18\|A0A1U8CY18_ALLSI kinectin isoform X5 OS=Alligator sinensis GN=KTN1 PE=4 SV=1 | 53288120,1 | 16 | 12 |
| 394 | \|A0A151N7Y8\|A0A151N7Y8_ALLMI 40S ribosomal protein S18 OS=Alligator mississippiensis GN=RPS18 PE=3 SV=1 | 53193156,9 | 12 | 4 |
| 395 | \|A0A151P1M1\|A0A151P1M1_ALLMI Uncharacterized protein OS=Alligator mississippiensis GN=Y1Q_0017155 PE=4 SV=1 | 53149048,9 | 4 | 2 |
| 396 | \|A0A1U8DQB0\|A0A1U8DQB0_ALLSI zinc finger protein 185 OS=Alligator sinensis GN=ZNF185 PE=4 SV=1 | 52992915,3 | 7 | 4 |
| 397 | \|A0A151PCX0\|A0A151PCX0_ALLMI ATP-dependent 6-phosphofructokinase OS=Alligator mississippiensis GN=Y1Q_0014478 PE=3 SV=1 | 52838871,0 | 20 | 11 |
| 398 | \|Q9W6C9\|Q9W6C9_CAICR Alpha enolase (Fragment) OS=Caiman crocodilus PE=2 SV=1 | 52801582,6 | 10 | 3 |
| 399 | \|A0A151NND9\|A0A151NND9_ALLMI Moesin OS=Alligator mississippiensis GN=MSN PE=4 SV=1 | 52796778,8 | 26 | 13 |
| 400 | \|A0A151P8L7\|A0A151P8L7_ALLMI Ig lambda chain V-1 region OS=Alligator mississippiensis GN=IGLL1 PE=4 SV=1 | 52743327,3 | 5 | 1 |
| 401 | \|A0A151MZ83\|A0A151MZ83_ALLMI Uncharacterized protein OS=Alligator mississippiensis GN=Y1Q_0023132 PE=4 SV=1 | 52374509,8 | 8 | 4 |
| 402 | \|A0A1U7SYV6\|A0A1U7SYV6_ALLSI complement factor H-like OS=Alligator sinensis GN=LOC102372212 PE=4 SV=1 | 52362175,6 | 15 | 8 |
| 403 | \|A0A1U7RBX7\|A0A1U7RBX7_ALLSI 26S proteasome non-ATPase regulatory subunit 2 OS=Alligator sinensis GN=PSMD2 PE=4 SV=1 | 52106191,7 | 23 | 17 |
| 404 | \|A0A1U8DQI7\|A0A1U8DQI7_ALLSI verrucotoxin subunit beta-like OS=Alligator sinensis GN=LOC106723204 PE=4 SV=1 | 51991407,7 | 17 | 11 |
| 405 | \|A0A151MC27\|A0A151MC27_ALLMI Coatomer subunit beta OS=Alligator mississippiensis GN=COPB1 PE=4 SV=1 | 51902722,2 | 22 | 15 |
| 406 | \|A0A151PDD7\|A0A151PDD7_ALLMI Phosphoinositide phospholipase C OS=Alligator mississippiensis GN=PLCD1 PE=4 SV=1 | 51890949,1 | 18 | 10 |
| 407 | \|A0A1U7RRU3\|A0A1U7RRU3_ALLSI thioredoxin reductase 3 OS=Alligator sinensis GN=TXNRD3 PE=3 SV=1 | 51581398,6 | 4 | 1 |
| 408 | \|A0A1U7ST99\|A0A1U7ST99_ALLSI heterogeneous nuclear ribonucleoprotein A3 isoform X4 OS=Alligator sinensis GN=HNRNPA3 PE=4 SV=1 | 51483104,7 | 14 | 9 |
| 409 | \|A0A151PE27\|A0A151PE27_ALLMI ERO1-like protein beta OS=Alligator mississippiensis GN=ERO1B PE=4 SV=1 | 51342949,5 | 6 | 2 |
| 410 | \|A0A151NC26\|A0A151NC26_ALLMI Uncharacterized protein OS=Alligator mississippiensis GN=Y1Q_0002478 PE=3 SV=1 | 50758441,9 | 16 | 7 |
| 411 | \|A0A1U7SUH9\|A0A1U7SUH9_ALLSI fatty acid synthase OS=Alligator sinensis GN=FASN PE=4 SV=1 | 50507330,2 | 28 | 20 |
| 412 | \|A0A1U7RU76\|A0A1U7RU76_ALLSI ras-related C3 botulinum toxin substrate 2 OS=Alligator sinensis GN=RAC2 PE=3 SV=1 | 50456714,9 | 12 | 4 |
| 413 | \|A0A151MVT9\|A0A151MVT9_ALLMI Calpain small subunit 1 OS=Alligator mississippiensis GN=CAPNS1 PE=4 SV=1 | 50261016,5 | 16 | 5 |
| 414 | \|A0A151NU83\|A0A151NU83_ALLMI Uncharacterized protein OS=Alligator mississippiensis GN=Y1Q_0008988 PE=4 SV=1 | 50167715,4 | 24 | 17 |
| 415 | \|A0A151MU32\|A0A151MU32_ALLMI Phosphoacetylglucosamine mutase OS=Alligator mississippiensis GN=PGM3 PE=3 SV=1 | 49575956,5 | 4 | 2 |
| 416 | \|A0A1U7S1T7\|A0A1U7S1T7_ALLSI mesothelin-like protein OS=Alligator sinensis GN=LOC102368358 PE=4 SV=1 | 49497884,4 | 16 | 7 |
| 417 | \|A0A151MTI5\|A0A151MTI5_ALLMI Mannose-1-phosphate guanyltransferase beta OS=Alligator mississippiensis GN=GMPPB PE=4 SV=1 | 49398841,2 | 19 | 6 |
| 418 | \|A0A1U8DDL6\|A0A1U8DDL6_ALLSI LOW QUALITY PROTEIN: E3 ubiquitin-protein ligase HUWE1 OS=Alligator sinensis GN=HUWE1 PE=4 SV=1 | 49325332,2 | 22 | 14 |
| 419 | \|A0A1U7S0D5\|A0A1U7S0D5_ALLSI complement component C9 OS=Alligator sinensis GN=C9 PE=4 SV=1 | 48918134,8 | 6 | 4 |
| 420 | \|A0A1U7RJL4\|A0A1U7RJL4_ALLSI Ribosomal protein L15 OS=Alligator sinensis GN=RPL15 PE=3 SV=1 | 48549620,4 | 7 | 4 |
| 421 | \|A0A1U8DJJ5\|A0A1U8DJJ5_ALLSI EH domain-containing protein 3 isoform X1 OS=Alligator sinensis GN=EHD3 PE=3 SV=1 | 48532712,8 | 14 | 8 |
| 422 | \|A0A151P8X0\|A0A151P8X0_ALLMI 26S protease regulatory subunit 4 OS=Alligator mississippiensis GN=PSMC1 PE=3 SV=1 | 48510085,6 | 17 | 10 |
| 423 | \|A0A151N5A4\|A0A151N5A4_ALLMI Uncharacterized protein OS=Alligator mississippiensis GN=Y1Q_0007049 PE=4 SV=1 | 48458167,1 | 4 | 3 |
| 424 | \|A0A1U8DKB4\|A0A1U8DKB4_ALLSI actin, cytoplasmic 2 isoform X1 OS=Alligator sinensis GN=ACTG1 PE=3 SV=1 | 47960381,2 | 10 | 2 |
| 425 | \|A0A151NDT5\|A0A151NDT5_ALLMI Mannose-1-phosphate guanyltransferase alpha OS=Alligator mississippiensis GN=GMPPA PE=4 SV=1 | 47927519,7 | 11 | 5 |
| 426 | \|A0A1U7SJ23\|A0A1U7SJ23_ALLSI spliceosome RNA helicase DDX39B OS=Alligator sinensis GN=LOC102378570 PE=4 SV=1 | 47924220,2 | 23 | 12 |
| 427 | \|A0A151MML3\|A0A151MML3_ALLMI 60S ribosomal protein L11 isoform A OS=Alligator mississippiensis GN=RPL11-1 PE=3 SV=1 | 47883929,6 | 11 | 6 |
| 428 | \|A0A151NM23\|A0A151NM23_ALLMI Eukaryotic translation initiation factor 4 gamma 3 OS=Alligator mississippiensis GN=EIF4G3 PE=4 SV=1 | 47877442,3 | 19 | 10 |
| 429 | \|A0A151MLU5\|A0A151MLU5_ALLMI Glutathione synthetase OS=Alligator mississippiensis GN=GSS PE=3 SV=1 | 47487865,1 | 12 | 6 |
| 430 | \|A0A151N8R4\|A0A151N8R4_ALLMI Adenosylhomocysteinase OS=Alligator mississippiensis GN=AHCYL1 PE=3 SV=1 | 47476839,9 | 15 | 10 |
| 431 | \|A0A151LYF4\|A0A151LYF4_ALLMI Uncharacterized protein OS=Alligator mississippiensis GN=Y1Q_0017726 PE=3 SV=1 | 47355293,7 | 22 | 12 |
| 432 | \|A0A151M2T4\|A0A151M2T4_ALLMI Translin-associated protein X OS=Alligator mississippiensis GN=TSNAX PE=4 SV=1 | 47141770,6 | 23 | 12 |
| 433 | \|A0A151N3K9\|A0A151N3K9_ALLMI Glia maturation factor beta OS=Alligator mississippiensis GN=GMFB PE=4 SV=1 | 47117423,3 | 10 | 4 |
| 434 | \|A0A151MUT0\|A0A151MUT0_ALLMI Guanine nucleotide-binding protein subunit beta-2-like 1 OS=Alligator mississippiensis GN=GNB2L1 PE=4 SV=1 | 46658722,6 | 21 | 10 |
| 435 | \|A0A1U8D3T9\|A0A1U8D3T9_ALLSI leucine-rich alpha-2-glycoprotein OS=Alligator sinensis GN=LRG1 PE=4 SV=1 | 46605628,2 | 12 | 4 |
| 436 | \|A0A151NED0\|A0A151NED0_ALLMI Putative ATP-dependent RNA helicase DDX58 OS=Alligator mississippiensis GN=DDX58 PE=4 SV=1 | 46018546,2 | 17 | 10 |
| 437 | \|A0A151NJD3\|A0A151NJD3_ALLMI Adenylate kinase 4, mitochondrial OS=Alligator mississippiensis GN=AK4 PE=3 SV=1 | 45916394,3 | 13 | 5 |
| 438 | \|A0A151P0E0\|A0A151P0E0_ALLMI Uncharacterized protein OS=Alligator mississippiensis GN=Y1Q_0022223 PE=3 SV=1 | 45770912,0 | 16 | 9 |
| 439 | \|A0A1U7RI13\|A0A1U7RI13_ALLSI ATP-binding cassette sub-family E member 1 OS=Alligator sinensis GN=ABCE1 PE=4 SV=1 | 45741724,5 | 21 | 9 |
| 440 | \|A0A151N0I1\|A0A151N0I1_ALLMI Uncharacterized protein OS=Alligator mississippiensis GN=Y1Q_0018487 PE=3 SV=1 | 45639564,4 | 10 | 7 |
| 441 | \|A0A151P440\|A0A151P440_ALLMI Brain-specific angiogenesis inhibitor 1-associated protein 2 isoform A OS=Alligator mississippiensis GN=BAIAP2 PE=4 SV=1 | 45501874,6 | 5 | 4 |
| 442 | \|A0A151M8V4\|A0A151M8V4_ALLMI Acidic leucine-rich nuclear phosphoprotein 32 family member A OS=Alligator mississippiensis GN=ANP32A PE=4 SV=1 | 45424582,2 | 15 | 9 |
| 443 | \|A0A1U8D405\|A0A1U8D405_ALLSI acylamino-acid-releasing enzyme OS=Alligator sinensis GN=APEH PE=4 SV=1 | 45371920,6 | 12 | 6 |
| 444 | \|A0A1U7RBP4\|A0A1U7RBP4_ALLSI Proteasome subunit beta type OS=Alligator sinensis GN=PSMB1 PE=3 SV=1 | 45251423,7 | 21 | 10 |
| 445 | \|A0A151P7J1\|A0A151P7J1_ALLMI ES1 protein-like protein, mitochondrial OS=Alligator mississippiensis GN=Y1Q_0007363 PE=4 SV=1 | 45134724,7 | 18 | 5 |
| 446 | \|A0A151MLD7\|A0A151MLD7_ALLMI Prothymosin alpha OS=Alligator mississippiensis GN=PTMA PE=4 SV=1 | 45032746,6 | 33 | 5 |
| 447 | \|A0A151PIN7\|A0A151PIN7_ALLMI CD2-associated protein isoform B OS=Alligator mississippiensis GN=CD2AP PE=4 SV=1 | 44937327,0 | 16 | 8 |
| 448 | \|A0A1U7SII6\|A0A1U7SII6_ALLSI C-factor-like OS=Alligator sinensis GN=LOC102378992 PE=3 SV=1 | 44778199,4 | 17 | 9 |
| 449 | \|A0A151P586\|A0A151P586_ALLMI 26S proteasome non-ATPase regulatory subunit 3 OS=Alligator mississippiensis GN=PSMD3 PE=4 SV=1 | 44352056,1 | 20 | 11 |
| 450 | \|A0A151NXU0\|A0A151NXU0_ALLMI 26S proteasome non-ATPase regulatory subunit 13 OS=Alligator mississippiensis GN=PSMD13 PE=4 SV=1 | 44339661,5 | 20 | 10 |
| 451 | \|A0A151NB21\|A0A151NB21_ALLMI Profilin OS=Alligator mississippiensis GN=PFN2-1 PE=3 SV=1 | 44302366,1 | 10 | 4 |
| 452 | \|A0A151MFF2\|A0A151MFF2_ALLMI Protein SET OS=Alligator mississippiensis GN=SET PE=3 SV=1 | 44263052,8 | 13 | 6 |
| 453 | \|A0A151LZA8\|A0A151LZA8_ALLMI S-methyl-5'-thioadenosine phosphorylase OS=Alligator mississippiensis GN=MTAP PE=3 SV=1 | 44245131,0 | 15 | 6 |
| 454 | \|A0A1U7RRA2\|A0A1U7RRA2_ALLSI WD repeat-containing protein 44 OS=Alligator sinensis GN=WDR44 PE=4 SV=1 | 44005476,9 | 17 | 9 |
| 455 | \|A0A151MZP7\|A0A151MZP7_ALLMI Nucleolin OS=Alligator mississippiensis GN=NCL PE=4 SV=1 | 43988915,8 | 28 | 12 |
| 456 | \|A0A151MFP7\|A0A151MFP7_ALLMI 60S ribosomal protein L7a OS=Alligator mississippiensis GN=RPL7A PE=4 SV=1 | 43975155,3 | 17 | 9 |
| 457 | \|A0A151MB32\|A0A151MB32_ALLMI 26S protease regulatory subunit 6A OS=Alligator mississippiensis GN=PSMC3 PE=3 SV=1 | 43868024,5 | 20 | 11 |
| 458 | \|A0A151MZ12\|A0A151MZ12_ALLMI Uncharacterized protein OS=Alligator mississippiensis GN=Y1Q_0005955 PE=4 SV=1 | 43469963,6 | 5 | 4 |
| 459 | \|A0A1U8DRS9\|A0A1U8DRS9_ALLSI uncharacterized protein LOC102380967 OS=Alligator sinensis GN=LOC102380967 PE=4 SV=1 | 43412190,0 | 12 | 4 |
| 460 | \|A0A151N2I9\|A0A151N2I9_ALLMI 26S proteasome non-ATPase regulatory subunit 12 OS=Alligator mississippiensis GN=PSMD12 PE=4 SV=1 | 43329511,9 | 16 | 9 |
| 461 | \|A0A151PBS0\|A0A151PBS0_ALLMI Prostaglandin E synthase 3 OS=Alligator mississippiensis GN=PTGES3 PE=4 SV=1 | 43056328,2 | 11 | 6 |
| 462 | \|A0A151NFR0\|A0A151NFR0_ALLMI Glutamine--tRNA ligase OS=Alligator mississippiensis GN=QARS PE=3 SV=1 | 43013666,8 | 20 | 14 |
| 463 | \|A0A151NU24\|A0A151NU24_ALLMI Dynactin subunit 2 OS=Alligator mississippiensis GN=DCTN2 PE=4 SV=1 | 42888293,2 | 12 | 5 |
| 464 | \|A0A151NWY6\|A0A151NWY6_ALLMI U5 small nuclear ribonucleoprotein component OS=Alligator mississippiensis GN=EFTUD2 PE=4 SV=1 | 42767269,8 | 18 | 9 |
| 465 | \|A0A1U8DJ17\|A0A1U8DJ17_ALLSI nuclear GTPase SLIP-GC-like OS=Alligator sinensis GN=LOC106722788 PE=4 SV=1 | 42493185,7 | 10 | 3 |
| 466 | \|A0A151P605\|A0A151P605_ALLMI Uncharacterized protein OS=Alligator mississippiensis GN=Y1Q_0016752 PE=4 SV=1 | 42404255,8 | 24 | 14 |
| 467 | \|A0A151NGA3\|A0A151NGA3_ALLMI M7GpppX diphosphatase OS=Alligator mississippiensis GN=DCPS PE=4 SV=1 | 41915070,1 | 13 | 9 |
| 468 | \|A0A151NJ11\|A0A151NJ11_ALLMI Serine-threonine kinase receptor-associated protein OS=Alligator mississippiensis GN=STRAP PE=4 SV=1 | 41838360,7 | 6 | 4 |
| 469 | \|A0A151N9V9\|A0A151N9V9_ALLMI Uncharacterized protein OS=Alligator mississippiensis GN=Y1Q_0008772 PE=4 SV=1 | 41654097,2 | 13 | 9 |
| 470 | \|A0A1U7RLW0\|A0A1U7RLW0_ALLSI uncharacterized protein LOC102375295 OS=Alligator sinensis GN=LOC102375295 PE=3 SV=1 | 41596562,7 | 7 | 3 |
| 471 | \|A0A1U8CWA8\|A0A1U8CWA8_ALLSI microtubule-actin cross-linking factor 1 OS=Alligator sinensis GN=MACF1 PE=4 SV=1 | 41570704,2 | 8 | 5 |
| 472 | \|A0A151N9U1\|A0A151N9U1_ALLMI Proteasome (Prosome, macropain) 26S subunit, ATPase, 5 OS=Alligator mississippiensis GN=PSMC5 PE=3 SV=1 | 41280635,6 | 18 | 11 |
| 473 | \|A0A151PHU1\|A0A151PHU1_ALLMI Uncharacterized protein OS=Alligator mississippiensis GN=Y1Q_0003986 PE=4 SV=1 | 41165805,9 | 15 | 7 |
| 474 | \|A0A151NIZ1\|A0A151NIZ1_ALLMI V-type proton ATPase subunit E 1 OS=Alligator mississippiensis GN=ATP6V1E1 PE=3 SV=1 | 41161794,2 | 12 | 7 |
| 475 | \|A0A151MLU1\|A0A151MLU1_ALLMI Proteasome subunit beta type OS=Alligator mississippiensis GN=PSMB9 PE=3 SV=1 | 40988141,4 | 9 | 3 |
| 476 | \|A0A0Q3ZXL6\|A0A0Q3ZXL6_ALLMI Calmodulin OS=Alligator mississippiensis GN=CALM1 PE=4 SV=1 | 40912818,6 | 13 | 5 |
| 477 | \|A0A151NXH9\|A0A151NXH9_ALLMI Uncharacterized protein OS=Alligator mississippiensis GN=Y1Q_0006281 PE=4 SV=1 | 40777502,3 | 17 | 11 |
| 478 | \|A0A1U7S7U1\|A0A1U7S7U1_ALLSI LOW QUALITY PROTEIN: alanine--tRNA ligase, cytoplasmic OS=Alligator sinensis GN=AARS PE=3 SV=1 | 40647909,8 | 15 | 10 |
| 479 | \|A0A151NRQ1\|A0A151NRQ1_ALLMI Transgelin OS=Alligator mississippiensis GN=IGSF9B PE=3 SV=1 | 40625555,7 | 13 | 8 |
| 480 | \|A0A1U7S923\|A0A1U7S923_ALLSI 26S proteasome non-ATPase regulatory subunit 11 OS=Alligator sinensis GN=PSMD11 PE=4 SV=1 | 40540733,3 | 22 | 13 |
| 481 | \|A0A1U8DMZ1\|A0A1U8DMZ1_ALLSI PCTP-like protein OS=Alligator sinensis GN=STARD10 PE=4 SV=1 | 40427678,9 | 10 | 6 |
| 482 | \|A0A151M1P9\|A0A151M1P9_ALLMI Cystatin B (Stefin B) OS=Alligator mississippiensis GN=CSTB PE=4 SV=1 | 40302773,8 | 6 | 2 |
| 483 | \|A0A151PDE9\|A0A151PDE9_ALLMI Uncharacterized protein OS=Alligator mississippiensis GN=Y1Q_0014548 PE=4 SV=1 | 40098388,5 | 11 | 4 |
| 484 | \|A0A151MNL8\|A0A151MNL8_ALLMI V-type proton ATPase subunit H OS=Alligator mississippiensis GN=ATP6V1H PE=4 SV=1 | 40022263,3 | 4 | 3 |
| 485 | \|A0A151N491\|A0A151N491_ALLMI Beta-ureidopropionase OS=Alligator mississippiensis GN=UPB1 PE=4 SV=1 | 39970222,1 | 15 | 8 |
| 486 | \|A0A151MG02\|A0A151MG02_ALLMI 26S proteasome non-ATPase regulatory subunit 5 isoform B OS=Alligator mississippiensis GN=PSMD5-1 PE=4 SV=1 | 39820032,0 | 17 | 8 |
| 487 | \|A0A151N8W8\|A0A151N8W8_ALLMI Voltage-dependent L-type calcium channel subunit alpha OS=Alligator mississippiensis GN=CACNA1E PE=3 SV=1 | 39809984,2 | 3 | 1 |
| 488 | \|A0A151MIB6\|A0A151MIB6_ALLMI Actin-related protein 2/3 complex subunit 5 OS=Alligator mississippiensis GN=ARPC5 PE=3 SV=1 | 39695333,8 | 21 | 7 |
| 489 | \|A0A151NYS1\|A0A151NYS1_ALLMI Ras-related protein Rab-25 OS=Alligator mississippiensis GN=RAB25 PE=4 SV=1 | 39641654,8 | 9 | 4 |
| 490 | \|A0A151NXF7\|A0A151NXF7_ALLMI Brain acid soluble protein 1 OS=Alligator mississippiensis GN=BASP1 PE=4 SV=1 | 39536487,2 | 16 | 8 |
| 491 | \|A0A151M8X2\|A0A151M8X2_ALLMI 60S ribosomal protein L4 OS=Alligator mississippiensis GN=RPL4 PE=4 SV=1 | 39324807,2 | 18 | 9 |
| 492 | \|A0A1U7SB30\|A0A1U7SB30_ALLSI importin subunit beta-1 OS=Alligator sinensis GN=KPNB1 PE=4 SV=1 | 39314436,4 | 14 | 8 |
| 493 | \|A0A1U8CZ22\|A0A1U8CZ22_ALLSI ficolin-3 isoform X3 OS=Alligator sinensis GN=FCN3 PE=4 SV=1 | 39294719,0 | 6 | 2 |
| 494 | \|A0A1U7SS44\|A0A1U7SS44_ALLSI Peptidyl-prolyl cis-trans isomerase OS=Alligator sinensis GN=PPIA PE=3 SV=1 | 39248208,5 | 9 | 6 |
| 495 | \|A0A151P0N0\|A0A151P0N0_ALLMI Coatomer subunit epsilon OS=Alligator mississippiensis GN=COPE PE=3 SV=1 | 39102145,4 | 11 | 6 |
| 496 | \|A0A1U7RK44\|A0A1U7RK44_ALLSI C4b-binding protein alpha chain-like isoform X1 OS=Alligator sinensis GN=LOC102381982 PE=4 SV=1 | 39091860,7 | 9 | 3 |
| 497 | \|A0A1U7SD81\|A0A1U7SD81_ALLSI ficolin-2-like OS=Alligator sinensis GN=LOC102377990 PE=4 SV=1 | 39018614,2 | 14 | 3 |
| 498 | \|A0A151MTP1\|A0A151MTP1_ALLMI Uncharacterized protein OS=Alligator mississippiensis GN=Y1Q_0006897 PE=3 SV=1 | 38995320,8 | 23 | 14 |
| 499 | \|A0A151PCM3\|A0A151PCM3_ALLMI Kinesin-like protein OS=Alligator mississippiensis GN=Y1Q_0014452 PE=3 SV=1 | 38969057,2 | 26 | 17 |
| 500 | \|A0A1U8DCG8\|A0A1U8DCG8_ALLSI UDP-glucose 6-dehydrogenase OS=Alligator sinensis GN=UGDH PE=3 SV=1 | 38789893,8 | 5 | 3 |
| 501 | \|A0A151NJ50\|A0A151NJ50_ALLMI Far upstream element-binding protein 1 isoform A OS=Alligator mississippiensis GN=FUBP1 PE=4 SV=1 | 38765718,4 | 8 | 4 |
| 502 | \|A0A1U7R1E4\|A0A1U7R1E4_ALLSI Annexin OS=Alligator sinensis GN=ANXA5 PE=3 SV=1 | 38731404,1 | 8 | 4 |
| 503 | \|A0A151M5P0\|A0A151M5P0_ALLMI Uncharacterized protein OS=Alligator mississippiensis GN=Y1Q_0003944 PE=4 SV=1 | 38630770,7 | 9 | 4 |
| 504 | \|A0A151N8Q9\|A0A151N8Q9_ALLMI Plakophilin-1 isoform B OS=Alligator mississippiensis GN=PKP1 PE=4 SV=1 | 38520557,4 | 5 | 2 |
| 505 | \|A0A151MH10\|A0A151MH10_ALLMI Galectin OS=Alligator mississippiensis GN=LGALSL PE=4 SV=1 | 38126883,5 | 9 | 4 |
| 506 | \|A0A151PEB7\|A0A151PEB7_ALLMI Hepatoma-derived growth factor OS=Alligator mississippiensis GN=HDGF PE=4 SV=1 | 38110125,8 | 16 | 11 |
| 507 | \|A0A151N5Z3\|A0A151N5Z3_ALLMI Uncharacterized protein OS=Alligator mississippiensis GN=Y1Q_0021921 PE=4 SV=1 | 37742566,2 | 9 | 4 |
| 508 | \|A0A1U7S542\|A0A1U7S542_ALLSI 60S ribosomal protein L10 OS=Alligator sinensis GN=RPL10 PE=4 SV=1 | 37713711,1 | 13 | 6 |
| 509 | \|A0A151MC71\|A0A151MC71_ALLMI Cysteine--tRNA ligase, cytoplasmic isoform B OS=Alligator mississippiensis GN=CARS PE=3 SV=1 | 37541468,0 | 13 | 9 |
| 510 | \|A0A1U8DKE3\|A0A1U8DKE3_ALLSI vitelline membrane outer layer protein 1 homolog OS=Alligator sinensis GN=VMO1 PE=4 SV=1 | 37456043,1 | 11 | 5 |
| 511 | \|A0A151N5L0\|A0A151N5L0_ALLMI Thioredoxin domain-containing protein 17 OS=Alligator mississippiensis GN=TXNDC17 PE=4 SV=1 | 37369506,9 | 11 | 4 |
| 512 | \|A0A1U8DG33\|A0A1U8DG33_ALLSI unconventional myosin-Id OS=Alligator sinensis GN=MYO1D PE=3 SV=1 | 37081498,6 | 15 | 11 |
| 513 | \|A0A151MG15\|A0A151MG15_ALLMI Uncharacterized protein OS=Alligator mississippiensis GN=Y1Q_0005826 PE=4 SV=1 | 36780986,7 | 10 | 7 |
| 514 | \|A0A151PGB7\|A0A151PGB7_ALLMI Proteasome subunit beta type OS=Alligator mississippiensis GN=PSMB2 PE=3 SV=1 | 36730058,2 | 18 | 8 |
| 515 | \|A0A1U8DK13\|A0A1U8DK13_ALLSI LOW QUALITY PROTEIN: protein AHNAK2 OS=Alligator sinensis GN=AHNAK2 PE=4 SV=1 | 36676604,6 | 16 | 10 |
| 516 | \|A0A151NQ27\|A0A151NQ27_ALLMI ADP-sugar pyrophosphatase OS=Alligator mississippiensis GN=NUDT5 PE=3 SV=1 | 36587661,6 | 12 | 6 |
| 517 | \|A0A151NKU2\|A0A151NKU2_ALLMI Ubiquitin carboxyl-terminal hydrolase OS=Alligator mississippiensis GN=UCHL3-1 PE=3 SV=1 | 36487411,7 | 18 | 7 |
| 518 | \|A0A1U8DKD1\|A0A1U8DKD1_ALLSI LOW QUALITY PROTEIN: complement C4 OS=Alligator sinensis GN=LOC102383747 PE=4 SV=1 | 36478297,7 | 20 | 14 |
| 519 | \|A9CPG7\|A9CPG7_ALLMI Cold-inducible RNA binding protein OS=Alligator mississippiensis GN=CIRBP PE=2 SV=1 | 36394588,5 | 10 | 3 |
| 520 | \|A0A151M3X9\|A0A151M3X9_ALLMI Ubiquitin thioesterase OS=Alligator mississippiensis GN=OTUB1 PE=3 SV=1 | 36369929,8 | 14 | 8 |
| 521 | \|A0A1U8D1C0\|A0A1U8D1C0_ALLSI septin-7 OS=Alligator sinensis GN=SEPT7 PE=3 SV=1 | 36311501,2 | 20 | 10 |
| 522 | \|A0A1U7S483\|A0A1U7S483_ALLSI uncharacterized protein LOC102368608 OS=Alligator sinensis GN=LOC102368608 PE=4 SV=1 | 36185188,4 | 16 | 7 |
| 523 | \|A0A151N0P7\|A0A151N0P7_ALLMI Pirin OS=Alligator mississippiensis GN=PIR PE=3 SV=1 | 35892611,1 | 15 | 8 |
| 524 | \|A0A1U8DSP4\|A0A1U8DSP4_ALLSI vitellogenin-2-like OS=Alligator sinensis GN=LOC102371934 PE=4 SV=1 | 35696158,0 | 10 | 8 |
| 525 | \|A0A151PD78\|A0A151PD78_ALLMI Glycine--tRNA ligase OS=Alligator mississippiensis GN=GARS PE=4 SV=1 | 35567268,7 | 16 | 9 |
| 526 | \|A0A151NYS5\|A0A151NYS5_ALLMI Putative aminopeptidase NPEPL1 OS=Alligator mississippiensis GN=NPEPL1 PE=4 SV=1 | 35364768,0 | 13 | 9 |
| 527 | \|A0A151N033\|A0A151N033_ALLMI Tumor protein D54 isoform B OS=Alligator mississippiensis GN=TPD52L2-1 PE=4 SV=1 | 35283551,3 | 10 | 4 |
| 528 | \|A0A1U8DN06\|A0A1U8DN06_ALLSI Sulfotransferase OS=Alligator sinensis GN=LOC102370036 PE=3 SV=1 | 35281060,5 | 11 | 5 |
| 529 | \|A0A1U8D4E7\|A0A1U8D4E7_ALLSI Ribosomal protein OS=Alligator sinensis GN=RPL10A PE=3 SV=1 | 35240896,6 | 8 | 5 |
| 530 | \|A0A151MZS4\|A0A151MZS4_ALLMI Tyrosine-protein kinase OS=Alligator mississippiensis GN=PTK6 PE=3 SV=1 | 35230115,1 | 10 | 6 |
| 531 | \|A0A151P727\|A0A151P727_ALLMI 40S ribosomal protein S11 isoform A OS=Alligator mississippiensis GN=RPS11 PE=3 SV=1 | 35206527,6 | 13 | 5 |
| 532 | \|V9TTB7\|V9TTB7_CAICR Complement component 3 (Fragment) OS=Caiman crocodilus GN=C3 PE=2 SV=1 | 35165823,9 | 15 | 9 |
| 533 | \|A0A1U8DEP6\|A0A1U8DEP6_ALLSI uncharacterized protein LOC102387935 OS=Alligator sinensis GN=LOC102387935 PE=3 SV=1 | 34681072,0 | 21 | 13 |
| 534 | \|A0A151NPB3\|A0A151NPB3_ALLMI Nicotinamide phosphoribosyltransferase OS=Alligator mississippiensis GN=NAMPT PE=4 SV=1 | 34642684,7 | 16 | 9 |
| 535 | \|A0A151MZK6\|A0A151MZK6_ALLMI Proteasome subunit beta type OS=Alligator mississippiensis GN=Y1Q_0005128 PE=3 SV=1 | 34477210,0 | 8 | 4 |
| 536 | \|A0A1U7SLN9\|A0A1U7SLN9_ALLSI ovostatin-like OS=Alligator sinensis GN=LOC102385863 PE=4 SV=1 | 34334620,3 | 17 | 12 |
| 537 | \|A0A151MZ86\|A0A151MZ86_ALLMI Constitutive coactivator of PPAR-gamma-like protein 1 OS=Alligator mississippiensis GN=FAM120A PE=4 SV=1 | 34324099,3 | 8 | 5 |
| 538 | \|A0A1U7RIN0\|A0A1U7RIN0_ALLSI C-1-tetrahydrofolate synthase, cytoplasmic OS=Alligator sinensis GN=MTHFD1 PE=3 SV=1 | 34190789,1 | 15 | 10 |
| 539 | \|A0A151P5D8\|A0A151P5D8_ALLMI Ribosomal protein L19 OS=Alligator mississippiensis GN=RPL19 PE=3 SV=1 | 34102358,3 | 5 | 3 |
| 540 | \|A0A1U7SLJ6\|A0A1U7SLJ6_ALLSI lamin-A-like OS=Alligator sinensis GN=LOC102370910 PE=3 SV=1 | 34082836,4 | 12 | 8 |
| 541 | \|A0A151NGE2\|A0A151NGE2_ALLMI Uncharacterized protein OS=Alligator mississippiensis GN=Y1Q_0007578 PE=4 SV=1 | 34065980,4 | 13 | 6 |
| 542 | \|A0A151P2J3\|A0A151P2J3_ALLMI Programmed cell death 6-interacting protein OS=Alligator mississippiensis GN=PDCD6IP PE=4 SV=1 | 33723713,5 | 9 | 3 |
| 543 | \|A0A151LYY3\|A0A151LYY3_ALLMI LUC7-like 2 OS=Alligator mississippiensis GN=LUC7L2 PE=4 SV=1 | 33500395,8 | 5 | 4 |
| 544 | \|A0A1U7SMK2\|A0A1U7SMK2_ALLSI talin-1 OS=Alligator sinensis GN=TLN1 PE=4 SV=1 | 33093009,8 | 28 | 13 |
| 545 | \|A0A1U7S134\|A0A1U7S134_ALLSI N-acylneuraminate cytidylyltransferase OS=Alligator sinensis GN=CMAS PE=4 SV=1 | 33067306,6 | 9 | 4 |
| 546 | \|A0A151NB22\|A0A151NB22_ALLMI Signal transducer and activator of transcription OS=Alligator mississippiensis GN=Y1Q_0008828 PE=3 SV=1 | 32998908,7 | 23 | 15 |
| 547 | \|A0A1U8DLX9\|A0A1U8DLX9_ALLSI 40S ribosomal protein S25 OS=Alligator sinensis GN=RPS25 PE=4 SV=1 | 32934290,3 | 7 | 3 |
| 548 | \|G8DV23\|G8DV23_GAVGA Beta-actin (Fragment) OS=Gavialis gangeticus GN=ACTB PE=4 SV=1 | 32875527,8 | 5 | 3 |
| 549 | \|A0A1U7SI74\|A0A1U7SI74_ALLSI L-xylulose reductase OS=Alligator sinensis GN=DCXR PE=4 SV=1 | 32814750,8 | 11 | 6 |
| 550 | \|A0A151NUG6\|A0A151NUG6_ALLMI EF-hand domain-containing protein D1 OS=Alligator mississippiensis GN=EFHD1 PE=4 SV=1 | 32647850,5 | 6 | 3 |
| 551 | \|A0A151NXJ6\|A0A151NXJ6_ALLMI 60S acidic ribosomal protein P2 isoform B OS=Alligator mississippiensis GN=RPLP2 PE=3 SV=1 | 32608646,7 | 7 | 3 |
| 552 | \|A0A151NIM0\|A0A151NIM0_ALLMI Uncharacterized protein OS=Alligator mississippiensis GN=Y1Q_0024331 PE=4 SV=1 | 32501053,7 | 8 | 5 |
| 553 | \|A0A1U7S642\|A0A1U7S642_ALLSI Aconitate hydratase OS=Alligator sinensis GN=ACO1 PE=3 SV=1 | 32442472,5 | 18 | 13 |
| 554 | \|A0A151P337\|A0A151P337_ALLMI Protein disulfide-isomerase OS=Alligator mississippiensis GN=PDIA4 PE=3 SV=1 | 32371924,4 | 9 | 7 |
| 555 | \|A0A1U7SKP6\|A0A1U7SKP6_ALLSI Phosphoacetylglucosamine mutase OS=Alligator sinensis GN=PGM3 PE=3 SV=1 | 32276595,4 | 8 | 4 |
| 556 | \|A0A1U7SF33\|A0A1U7SF33_ALLSI keratin, type II cytoskeletal 8 OS=Alligator sinensis GN=KRT8 PE=3 SV=1 | 32166788,6 | 12 | 5 |
| 557 | \|A0A1U8DAY5\|A0A1U8DAY5_ALLSI cytosolic purine 5'-nucleotidase isoform X1 OS=Alligator sinensis GN=NT5C2 PE=4 SV=1 | 31829406,1 | 10 | 8 |
| 558 | \|A0A151N2W5\|A0A151N2W5_ALLMI Na(+)/H(+) exchange regulatory cofactor NHE-RF OS=Alligator mississippiensis GN=SLC9A3R1 PE=4 SV=1 | 31828893,0 | 6 | 4 |
| 559 | \|A0A1U7RC19\|A0A1U7RC19_ALLSI myeloid protein 1-like OS=Alligator sinensis GN=LOC102377860 PE=4 SV=1 | 31819401,2 | 6 | 2 |
| 560 | \|A0A151MEY2\|A0A151MEY2_ALLMI Delta-aminolevulinic acid dehydratase OS=Alligator mississippiensis GN=ALAD PE=3 SV=1 | 31531834,3 | 11 | 5 |
| 561 | \|A0A151MIN5\|A0A151MIN5_ALLMI Phosphohistidine phosphatase-like OS=Alligator mississippiensis GN=Y1Q_0004375 PE=4 SV=1 | 31480030,3 | 8 | 3 |
| 562 | \|A0A151NQU4\|A0A151NQU4_ALLMI 60S ribosomal protein L8 OS=Alligator mississippiensis GN=RPL8 PE=3 SV=1 | 31386695,1 | 8 | 3 |
| 563 | \|A0A151P523\|A0A151P523_ALLMI Sulfotransferase OS=Alligator mississippiensis GN=Y1Q_0013888 PE=3 SV=1 | 31360487,6 | 6 | 3 |
| 564 | \|A0A1U7RVN4\|A0A1U7RVN4_ALLSI 60S ribosomal protein L26-like 1 OS=Alligator sinensis GN=RPL26L1 PE=3 SV=1 | 31304871,9 | 6 | 4 |
| 565 | \|A0A1U7S9K4\|A0A1U7S9K4_ALLSI cathelicidin-2-like OS=Alligator sinensis GN=LOC102379244 PE=3 SV=1 | 31195053,2 | 8 | 3 |
| 566 | \|A0A1U8D9F3\|A0A1U8D9F3_ALLSI N-acetylgalactosamine kinase OS=Alligator sinensis GN=GALK2 PE=4 SV=1 | 31156873,2 | 9 | 5 |
| 567 | \|A0A151NGA8\|A0A151NGA8_ALLMI Uncharacterized protein OS=Alligator mississippiensis GN=Y1Q_0015294 PE=3 SV=1 | 31100925,7 | 20 | 11 |
| 568 | \|A0A151NHU5\|A0A151NHU5_ALLMI Uncharacterized protein OS=Alligator mississippiensis GN=Y1Q_0024143 PE=3 SV=1 | 31037368,8 | 3 | 2 |
| 569 | \|A0A151NVN9\|A0A151NVN9_ALLMI Eukaryotic translation initiation factor 3 subunit A OS=Alligator mississippiensis GN=EIF3A PE=3 SV=1 | 31029057,8 | 16 | 10 |
| 570 | \|A0A151MNI6\|A0A151MNI6_ALLMI 40S ribosomal protein S20 OS=Alligator mississippiensis GN=RPS20 PE=3 SV=1 | 30956064,4 | 13 | 5 |
| 571 | \|A0A151MWU0\|A0A151MWU0_ALLMI Thioredoxin-like protein 1 OS=Alligator mississippiensis GN=TXNL1 PE=4 SV=1 | 30908130,7 | 20 | 10 |
| 572 | \|A0A151PDJ2\|A0A151PDJ2_ALLMI Uncharacterized protein OS=Alligator mississippiensis GN=Y1Q_0014554 PE=4 SV=1 | 30893744,0 | 17 | 5 |
| 573 | \|A0A151N739\|A0A151N739_ALLMI NEDD8-activating enzyme E1 regulatory subunit OS=Alligator mississippiensis GN=NAE1 PE=3 SV=1 | 30749660,1 | 9 | 6 |
| 574 | \|A0A1U8D807\|A0A1U8D807_ALLSI epidermal growth factor receptor kinase substrate 8-like protein 2 OS=Alligator sinensis GN=EPS8L2 PE=4 SV=1 | 30661097,0 | 20 | 9 |
| 575 | \|A0A151P0T5\|A0A151P0T5_ALLMI Cadherin-1 OS=Alligator mississippiensis GN=CDH1 PE=4 SV=1 | 30584031,6 | 7 | 3 |
| 576 | \|A0A151N1X5\|A0A151N1X5_ALLMI Uncharacterized protein OS=Alligator mississippiensis GN=Y1Q_0008360 PE=4 SV=1 | 30492554,5 | 7 | 5 |
| 577 | \|A0A151P1W2\|A0A151P1W2_ALLMI Dynactin subunit 1 isoform A OS=Alligator mississippiensis GN=DCTN1 PE=4 SV=1 | 30306244,7 | 17 | 11 |
| 578 | \|A0A151N984\|A0A151N984_ALLMI Vesicle-fusing ATPase OS=Alligator mississippiensis GN=NSF PE=4 SV=1 | 30295034,0 | 10 | 7 |
| 579 | \|A0A1U8DS83\|A0A1U8DS83_ALLSI IgGFc-binding protein-like OS=Alligator sinensis GN=LOC102371424 PE=4 SV=1 | 30253954,7 | 14 | 10 |
| 580 | \|A0A1U7SVR7\|A0A1U7SVR7_ALLSI Sulfhydryl oxidase OS=Alligator sinensis GN=LOC102374951 PE=4 SV=1 | 30134205,5 | 17 | 8 |
| 581 | \|A0A151NQV5\|A0A151NQV5_ALLMI Eukaryotic peptide chain release factor subunit 1 OS=Alligator mississippiensis GN=ETF1 PE=4 SV=1 | 30024888,5 | 17 | 11 |
| 582 | \|A0A151PGC4\|A0A151PGC4_ALLMI Splicing factor, proline-and glutamine-rich isoform A OS=Alligator mississippiensis GN=SFPQ PE=4 SV=1 | 29935127,9 | 7 | 4 |
| 583 | \|A0A151P5U7\|A0A151P5U7_ALLMI Serine/threonine-protein phosphatase 2A 55 kDa regulatory subunit B OS=Alligator mississippiensis GN=Y1Q_0016754 PE=3 SV=1 | 29841838,9 | 7 | 5 |
| 584 | \|A9CPG8\|A9CPG8_ALLMI Heat shock protein 70-binding protein OS=Alligator mississippiensis GN=HSP70-BP PE=2 SV=1 | 29734394,9 | 11 | 4 |
| 585 | \|A0A151MJ32\|A0A151MJ32_ALLMI Uncharacterized protein OS=Alligator mississippiensis GN=Y1Q_0023234 PE=4 SV=1 | 29658298,0 | 14 | 9 |
| 586 | \|A0A151P4L2\|A0A151P4L2_ALLMI 60S ribosomal protein L13 OS=Alligator mississippiensis GN=SPG7 PE=3 SV=1 | 29629292,7 | 12 | 5 |
| 587 | \|A0A151NPS3\|A0A151NPS3_ALLMI Inter-alpha-trypsin inhibitor heavy chain H2 isoform B OS=Alligator mississippiensis GN=ITIH2 PE=4 SV=1 | 29539139,3 | 6 | 5 |
| 588 | \|A0A1U8DID7\|A0A1U8DID7_ALLSI complement component C6-like OS=Alligator sinensis GN=LOC102376566 PE=4 SV=1 | 29499872,2 | 13 | 8 |
| 589 | \|A0A151M7W3\|A0A151M7W3_ALLMI Uncharacterized protein OS=Alligator mississippiensis GN=Y1Q_0023213 PE=4 SV=1 | 29392868,5 | 7 | 2 |
| 590 | \|A0A1U8DP08\|A0A1U8DP08_ALLSI complement factor H-like OS=Alligator sinensis GN=LOC106723316 PE=4 SV=1 | 29384641,4 | 11 | 3 |
| 591 | \|A0A1U8D860\|A0A1U8D860_ALLSI Density-regulated protein OS=Alligator sinensis GN=DENR PE=3 SV=1 | 29292063,3 | 6 | 2 |
| 592 | \|A0A1U7SQA0\|A0A1U7SQA0_ALLSI kinesin light chain 4 isoform X3 OS=Alligator sinensis GN=KLC4 PE=4 SV=1 | 29159554,5 | 10 | 8 |
| 593 | \|A0A151MLS1\|A0A151MLS1_ALLMI Heparin cofactor 2 isoform B OS=Alligator mississippiensis GN=SERPIND1 PE=3 SV=1 | 29104363,7 | 13 | 5 |
| 594 | \|A0A1U7RQC0\|A0A1U7RQC0_ALLSI serine/threonine-protein kinase TAO3 OS=Alligator sinensis GN=TAOK3 PE=4 SV=1 | 29077722,9 | 8 | 4 |
| 595 | \|A0A151PG33\|A0A151PG33_ALLMI Phosphatidylinositol 5-phosphate 4-kinase type-2 gamma OS=Alligator mississippiensis GN=PIP4K2C PE=4 SV=1 | 28728943,8 | 7 | 3 |
| 596 | \|A0A1U8DE14\|A0A1U8DE14_ALLSI serine/threonine-protein phosphatase CPPED1 OS=Alligator sinensis GN=CPPED1 PE=4 SV=1 | 28610348,7 | 10 | 6 |
| 597 | \|A0A151MZ21\|A0A151MZ21_ALLMI ADP-ribosylation factor 4 OS=Alligator mississippiensis GN=ARF4 PE=3 SV=1 | 28560121,3 | 11 | 4 |
| 598 | \|A0A151N753\|A0A151N753_ALLMI Programmed cell death protein 5 OS=Alligator mississippiensis GN=PDCD5 PE=4 SV=1 | 28474008,2 | 10 | 3 |
| 599 | \|A0A151MF37\|A0A151MF37_ALLMI Chloride intracellular channel protein 3 isoform B OS=Alligator mississippiensis GN=CLIC3-1 PE=4 SV=1 | 28460338,1 | 13 | 6 |
| 600 | \|A0A151NFE8\|A0A151NFE8_ALLMI Cytosolic non-specific dipeptidase OS=Alligator mississippiensis GN=CNDP2 PE=4 SV=1 | 28424290,8 | 11 | 9 |
| 601 | \|A0A1U8D1M9\|A0A1U8D1M9_ALLSI copper transport protein ATOX1 OS=Alligator sinensis GN=ATOX1 PE=4 SV=1 | 28393184,3 | 3 | 1 |
| 602 | \|A0A151ML29\|A0A151ML29_ALLMI Annexin OS=Alligator mississippiensis GN=ANXA8L1 PE=3 SV=1 | 28389053,3 | 4 | 1 |
| 603 | \|A0A1U7RWZ1\|A0A1U7RWZ1_ALLSI LOW QUALITY PROTEIN: ran-specific GTPase-activating protein OS=Alligator sinensis GN=RANBP1 PE=4 SV=1 | 28336135,9 | 8 | 6 |
| 604 | \|A0A151NS74\|A0A151NS74_ALLMI Actin-related protein 2/3 complex subunit 4 OS=Alligator mississippiensis GN=ARPC4 PE=4 SV=1 | 28241008,3 | 19 | 5 |
| 605 | \|A0A151MRV4\|A0A151MRV4_ALLMI Vacuolar protein sorting-associated protein 35 OS=Alligator mississippiensis GN=VPS35 PE=3 SV=1 | 28220148,1 | 19 | 12 |
| 606 | \|A0A151M4U7\|A0A151M4U7_ALLMI Hook-like protein 3 OS=Alligator mississippiensis GN=HOOK3 PE=4 SV=1 | 28186571,0 | 8 | 6 |
| 607 | \|A0A151NCF5\|A0A151NCF5_ALLMI Heterogeneous nuclear ribonucleoprotein M OS=Alligator mississippiensis GN=HNRNPM PE=4 SV=1 | 28185356,6 | 7 | 4 |
| 608 | \|A0A0M5KC71\|A0A0M5KC71_CRONI Mitogen-activated protein kinase (Fragment) OS=Crocodylus niloticus GN=MAPK1 PE=2 SV=1 | 28041256,0 | 13 | 5 |
| 609 | \|A0A1U8E0Z2\|A0A1U8E0Z2_ALLSI complement factor H-like OS=Alligator sinensis GN=LOC102378843 PE=4 SV=1 | 28002301,6 | 11 | 5 |
| 610 | \|A0A1U7SYM5\|A0A1U7SYM5_ALLSI 60 kDa SS-A/Ro ribonucleoprotein-like OS=Alligator sinensis GN=LOC102385652 PE=4 SV=1 | 27822378,8 | 15 | 8 |
| 611 | \|A0A151MN05\|A0A151MN05_ALLMI Tumor protein D52 isoform A OS=Alligator mississippiensis GN=TPD52-1 PE=4 SV=1 | 27707052,2 | 15 | 8 |
| 612 | \|A0A151N9K0\|A0A151N9K0_ALLMI Putative methyltransferase OS=Alligator mississippiensis GN=Y1Q_0008688 PE=4 SV=1 | 27542325,0 | 9 | 4 |
| 613 | \|A0A1U8DGX7\|A0A1U8DGX7_ALLSI Peptidyl-prolyl cis-trans isomerase OS=Alligator sinensis GN=PPIL1 PE=3 SV=1 | 27363860,0 | 7 | 4 |
| 614 | \|A0A151MZ29\|A0A151MZ29_ALLMI Prestin isoform C OS=Alligator mississippiensis GN=SLC26A5-1 PE=3 SV=1 | 26984473,4 | 3 | 1 |
| 615 | \|A0A151NBE4\|A0A151NBE4_ALLMI DnaJ-like protein subfamily B member 1 OS=Alligator mississippiensis GN=DNAJB1 PE=4 SV=1 | 26792693,2 | 8 | 6 |
| 616 | \|A0A151NCL1\|A0A151NCL1_ALLMI Vacuolar protein-sorting-associated protein 36 OS=Alligator mississippiensis GN=VPS36 PE=4 SV=1 | 26588412,1 | 3 | 3 |
| 617 | \|A0A1U7RZ45\|A0A1U7RZ45_ALLSI high mobility group nucleosome-binding domain-containing protein 5 OS=Alligator sinensis GN=HMGN5 PE=4 SV=1 | 26506030,2 | 8 | 3 |
| 618 | \|A0A1U7SCN7\|A0A1U7SCN7_ALLSI Malate dehydrogenase OS=Alligator sinensis GN=MDH2 PE=3 SV=1 | 26420708,5 | 17 | 11 |
| 619 | \|A0A151NJU3\|A0A151NJU3_ALLMI Nuclear migration protein nudC isoform B OS=Alligator mississippiensis GN=NUDC-1 PE=4 SV=1 | 26377220,1 | 10 | 6 |
| 620 | \|A0A151N172\|A0A151N172_ALLMI ADP-ribosylation factor-like protein 6-interacting protein 1 OS=Alligator mississippiensis GN=ARL6IP1 PE=3 SV=1 | 26235128,6 | 12 | 7 |
| 621 | \|A0A1U7RVC3\|A0A1U7RVC3_ALLSI protein FAM3D OS=Alligator sinensis GN=FAM3D PE=4 SV=1 | 26155932,2 | 12 | 5 |
| 622 | \|A0A1U7SDR1\|A0A1U7SDR1_ALLSI fatty acid-binding protein, liver OS=Alligator sinensis GN=FABP1 PE=3 SV=1 | 26085404,0 | 4 | 2 |
| 623 | \|A0A151MCU2\|A0A151MCU2_ALLMI TIP41-like protein OS=Alligator mississippiensis GN=TIPRL PE=4 SV=1 | 26077221,1 | 6 | 4 |
| 624 | \|A0A151N2X6\|A0A151N2X6_ALLMI Periplakin OS=Alligator mississippiensis GN=PPL PE=4 SV=1 | 26065870,1 | 5 | 3 |
| 625 | \|A0A1U8E068\|A0A1U8E068_ALLSI GTP-binding protein SAR1b OS=Alligator sinensis GN=SAR1B PE=3 SV=1 | 26014429,2 | 3 | 1 |
| 626 | \|A0A151NJ98\|A0A151NJ98_ALLMI Cystathionine gamma-lyase OS=Alligator mississippiensis GN=CTH PE=3 SV=1 | 26002895,9 | 10 | 6 |
| 627 | \|A0A151PED7\|A0A151PED7_ALLMI Uncharacterized protein OS=Alligator mississippiensis GN=Y1Q_0001185 PE=3 SV=1 | 26000037,5 | 16 | 10 |
| 628 | \|A0A1U7SMU1\|A0A1U7SMU1_ALLSI ras-related protein Rab-5C OS=Alligator sinensis GN=RAB5C PE=4 SV=1 | 25946355,2 | 16 | 7 |
| 629 | \|A0A151M797\|A0A151M797_ALLMI 40S ribosomal protein S14 OS=Alligator mississippiensis GN=Y1Q_0010910 PE=3 SV=1 | 25930615,2 | 10 | 7 |
| 630 | \|A0A151PET1\|A0A151PET1_ALLMI Testin OS=Alligator mississippiensis GN=TES PE=4 SV=1 | 25770752,9 | 8 | 6 |
| 631 | \|A0A151P764\|A0A151P764_ALLMI Paraspeckle component 1 isoform B OS=Alligator mississippiensis GN=PSPC1 PE=4 SV=1 | 25741521,2 | 9 | 7 |
| 632 | \|A0A151NEL7\|A0A151NEL7_ALLMI Myosin regulatory light chain 2, smooth muscle minor isoform OS=Alligator mississippiensis GN=MYL12A PE=4 SV=1 | 25675336,2 | 7 | 4 |
| 633 | \|A0A151NXB5\|A0A151NXB5_ALLMI Eukaryotic translation initiation factor 3 subunit C OS=Alligator mississippiensis GN=EIF3C PE=3 SV=1 | 25513487,5 | 5 | 3 |
| 634 | \|A0A1U8DEU3\|A0A1U8DEU3_ALLSI junction plakoglobin isoform X2 OS=Alligator sinensis GN=JUP PE=4 SV=1 | 25511310,8 | 12 | 7 |
| 635 | \|A0A151MDT4\|A0A151MDT4_ALLMI 1,4-alpha-glucan-branching enzyme OS=Alligator mississippiensis GN=GBE1 PE=4 SV=1 | 25344720,4 | 7 | 5 |
| 636 | \|A0A151NP11\|A0A151NP11_ALLMI 26S proteasome non-ATPase regulatory subunit 7 OS=Alligator mississippiensis GN=PSMD7 PE=4 SV=1 | 25180963,4 | 8 | 5 |
| 637 | \|A0A151P0R9\|A0A151P0R9_ALLMI Uncharacterized protein OS=Alligator mississippiensis GN=Y1Q_0022381 PE=4 SV=1 | 24964130,2 | 8 | 7 |
| 638 | \|A0A151NZK3\|A0A151NZK3_ALLMI Disks large-associated protein 4 OS=Alligator mississippiensis GN=DLGAP4 PE=4 SV=1 | 24815012,7 | 8 | 6 |
| 639 | \|A0A151P5E7\|A0A151P5E7_ALLMI 60S ribosomal protein L23 isoform A OS=Alligator mississippiensis GN=RPL23 PE=3 SV=1 | 24810789,1 | 6 | 3 |
| 640 | \|A0A151MZ93\|A0A151MZ93_ALLMI DCC-interacting protein 13-alpha OS=Alligator mississippiensis GN=APPL1 PE=4 SV=1 | 24539383,1 | 7 | 4 |
| 641 | \|A0A151PA03\|A0A151PA03_ALLMI Dihydropteridine reductase OS=Alligator mississippiensis GN=QDPR PE=4 SV=1 | 24419317,9 | 12 | 7 |
| 642 | \|A0A1U8DJ03\|A0A1U8DJ03_ALLSI cold shock domain-containing protein E1 isoform X1 OS=Alligator sinensis GN=CSDE1 PE=4 SV=1 | 24261122,8 | 10 | 6 |
| 643 | \|A0A151MX90\|A0A151MX90_ALLMI Complement component C7 OS=Alligator mississippiensis GN=C7 PE=4 SV=1 | 24142525,3 | 9 | 7 |
| 644 | \|A0A151P6I6\|A0A151P6I6_ALLMI Protein FAM115C OS=Alligator mississippiensis GN=TCAF2-2 PE=4 SV=1 | 24132034,7 | 3 | 2 |
| 645 | \|A0A151P4U3\|A0A151P4U3_ALLMI Phosphatidylinositol transfer protein beta OS=Alligator mississippiensis GN=PITPNB PE=4 SV=1 | 23980062,7 | 6 | 5 |
| 646 | \|A0A151MJZ7\|A0A151MJZ7_ALLMI Melanotransferrin OS=Alligator mississippiensis GN=MFI2 PE=4 SV=1 | 23816470,4 | 6 | 1 |
| 647 | \|A0A151M0E7\|A0A151M0E7_ALLMI 26S proteasome non-ATPase regulatory subunit 14 OS=Alligator mississippiensis GN=PSMD14 PE=4 SV=1 | 23669775,1 | 11 | 6 |
| 648 | \|A0A151NG14\|A0A151NG14_ALLMI Amyloid-like protein 2 isoform A OS=Alligator mississippiensis GN=APLP2-1 PE=4 SV=1 | 23664958,2 | 6 | 4 |
| 649 | \|A0A1U7SXA4\|A0A1U7SXA4_ALLSI uncharacterized protein LOC102383910 OS=Alligator sinensis GN=LOC102383910 PE=4 SV=1 | 23659003,2 | 10 | 4 |
| 650 | \|A0A151MSU1\|A0A151MSU1_ALLMI PDZ and LIM domain protein 1 OS=Alligator mississippiensis GN=PDLIM1 PE=4 SV=1 | 23630785,0 | 13 | 6 |
| 651 | \|A0A151MSE3\|A0A151MSE3_ALLMI 60S ribosomal protein L21 OS=Alligator mississippiensis GN=RPL21 PE=4 SV=1 | 23603421,0 | 4 | 3 |
| 652 | \|A0A151MNZ5\|A0A151MNZ5_ALLMI Desmocollin-2 isoform B OS=Alligator mississippiensis GN=DSC2 PE=4 SV=1 | 23440334,9 | 14 | 9 |
| 653 | \|A0A1U8D5C2\|A0A1U8D5C2_ALLSI Perilipin OS=Alligator sinensis GN=LOC102371531 PE=3 SV=1 | 23403400,4 | 15 | 7 |
| 654 | \|A0A1U7R1K6\|A0A1U7R1K6_ALLSI transcriptional activator protein Pur-alpha OS=Alligator sinensis GN=PURA PE=4 SV=1 | 23298314,4 | 9 | 5 |
| 655 | \|A0A151NYZ5\|A0A151NYZ5_ALLMI DNA damage-binding protein 1 OS=Alligator mississippiensis GN=DDB1 PE=4 SV=1 | 23063292,8 | 7 | 6 |
| 656 | \|A0A151N1F6\|A0A151N1F6_ALLMI Brain-specific angiogenesis inhibitor 1-associated protein 2-like protein 1 OS=Alligator mississippiensis GN=BAIAP2L1 PE=4 SV=1 | 23038854,1 | 8 | 7 |
| 657 | \|A0A151M641\|A0A151M641_ALLMI Calcium-binding protein 39 OS=Alligator mississippiensis GN=CAB39 PE=4 SV=1 | 22904476,7 | 6 | 6 |
| 658 | \|A0A151N808\|A0A151N808_ALLMI Serine/threonine-protein kinase 26 OS=Alligator mississippiensis GN=STK26 PE=4 SV=1 | 22773234,4 | 10 | 5 |
| 659 | \|A0A151MV57\|A0A151MV57_ALLMI Kunitz-type protease inhibitor 1 OS=Alligator mississippiensis GN=SPINT1 PE=4 SV=1 | 22769863,8 | 8 | 4 |
| 660 | \|A0A151MF59\|A0A151MF59_ALLMI V-type proton ATPase subunit G OS=Alligator mississippiensis GN=ATP6V1G1 PE=3 SV=1 | 22769563,2 | 6 | 4 |
| 661 | \|A0A151PAW5\|A0A151PAW5_ALLMI Caspase-3 OS=Alligator mississippiensis GN=CASP3 PE=3 SV=1 | 22698650,1 | 14 | 7 |
| 662 | \|A0A151NMS3\|A0A151NMS3_ALLMI Uncharacterized protein OS=Alligator mississippiensis GN=Y1Q_0007254 PE=3 SV=1 | 22671158,0 | 5 | 2 |
| 663 | \|A0A1U7SBJ9\|A0A1U7SBJ9_ALLSI aminoacyl tRNA synthase complex-interacting multifunctional protein 1 isoform X2 OS=Alligator sinensis GN=AIMP1 PE=4 SV=1 | 22581176,2 | 7 | 6 |
| 664 | \|A0A151N170\|A0A151N170_ALLMI Ubiquitin carboxyl-terminal hydrolase 7 OS=Alligator mississippiensis GN=USP7 PE=3 SV=1 | 22518822,4 | 12 | 8 |
| 665 | \|A0A1U7RWA4\|A0A1U7RWA4_ALLSI ras-related protein Rap-1b isoform X2 OS=Alligator sinensis GN=RAP1B PE=4 SV=1 | 22441130,7 | 12 | 5 |
| 666 | \|A0A151MD19\|A0A151MD19_ALLMI Sodium/potassium-transporting ATPase subunit alpha OS=Alligator mississippiensis GN=Y1Q_0003007 PE=3 SV=1 | 22417820,1 | 11 | 8 |
| 667 | \|A0A151P593\|A0A151P593_ALLMI Uncharacterized protein OS=Alligator mississippiensis GN=Y1Q_0015398 PE=4 SV=1 | 22199915,4 | 13 | 7 |
| 668 | \|A0A1U8D9W5\|A0A1U8D9W5_ALLSI quinone oxidoreductase PIG3 OS=Alligator sinensis GN=TP53I3 PE=4 SV=1 | 22162933,2 | 7 | 5 |
| 669 | \|A0A151MIU6\|A0A151MIU6_ALLMI 60S ribosomal protein L34 OS=Alligator mississippiensis GN=RPL34 PE=4 SV=1 | 22113104,8 | 3 | 3 |
| 670 | \|A0A1U8CVQ6\|A0A1U8CVQ6_ALLSI carboxymethylenebutenolidase homolog OS=Alligator sinensis GN=LOC106721788 PE=4 SV=1 | 22039823,0 | 12 | 9 |
| 671 | \|A0A1U8D027\|A0A1U8D027_ALLSI nucleosome assembly protein 1-like 4 isoform X3 OS=Alligator sinensis GN=NAP1L4 PE=3 SV=1 | 21985377,4 | 15 | 6 |
| 672 | \|A0A1U7SF20\|A0A1U7SF20_ALLSI proteasome activator complex subunit 2 OS=Alligator sinensis GN=PSME2 PE=4 SV=1 | 21968700,6 | 9 | 4 |
| 673 | \|A0A151MMP4\|A0A151MMP4_ALLMI Adenylate kinase 2, mitochondrial OS=Alligator mississippiensis GN=AK2 PE=3 SV=1 | 21917582,6 | 6 | 4 |
| 674 | >sp\|P81280\|CYC_ALLMI Cytochrome c OS=Alligator mississippiensis PE=1 SV=2 | 21837205,1 | 6 | 3 |
| 675 | \|A0A151P3K2\|A0A151P3K2_ALLMI Protein-glutamine gamma-glutamyltransferase 4 isoform A OS=Alligator mississippiensis GN=TGM4 PE=4 SV=1 | 21837043,7 | 7 | 4 |
| 676 | \|A0A151M2M3\|A0A151M2M3_ALLMI Conserved oligomeric Golgi complex subunit 2 OS=Alligator mississippiensis GN=COG2 PE=4 SV=1 | 21793363,4 | 5 | 3 |
| 677 | \|A0A151M8N3\|A0A151M8N3_ALLMI PH domain leucine-rich repeat-containing protein phosphatase 2 isoform A OS=Alligator mississippiensis GN=PHLPP2 PE=4 SV=1 | 21790939,0 | 13 | 8 |
| 678 | \|A0A1U7S7G9\|A0A1U7S7G9_ALLSI heterogeneous nuclear ribonucleoprotein A/B isoform X1 OS=Alligator sinensis GN=HNRNPAB PE=4 SV=1 | 21701202,8 | 7 | 4 |
| 679 | \|A0A151NHT1\|A0A151NHT1_ALLMI ATP-dependent 6-phosphofructokinase OS=Alligator mississippiensis GN=PFKP PE=3 SV=1 | 21684781,8 | 11 | 6 |
| 680 | \|A0A151NCG5\|A0A151NCG5_ALLMI Ras-related protein Rab-11B OS=Alligator mississippiensis GN=RAB11B PE=4 SV=1 | 21630992,4 | 7 | 3 |
| 681 | \|A0A1U8DSJ3\|A0A1U8DSJ3_ALLSI phosphoglycerate mutase 1 OS=Alligator sinensis GN=PGAM1 PE=4 SV=1 | 21508623,5 | 5 | 2 |
| 682 | \|A0A151MCL0\|A0A151MCL0_ALLMI Uncharacterized protein OS=Alligator mississippiensis GN=Y1Q_0002878 PE=4 SV=1 | 21182901,6 | 9 | 5 |
| 683 | \|A0A151PFY8\|A0A151PFY8_ALLMI Peroxiredoxin-1 OS=Alligator mississippiensis GN=PRDX1 PE=4 SV=1 | 21178638,8 | 13 | 5 |
| 684 | \|A0A151MD24\|A0A151MD24_ALLMI Tetraspanin OS=Alligator mississippiensis GN=UPK1B PE=3 SV=1 | 21101245,2 | 4 | 2 |
| 685 | \|A0A151NMD0\|A0A151NMD0_ALLMI Serine/threonine-protein phosphatase OS=Alligator mississippiensis GN=PPP3CC PE=3 SV=1 | 21089500,0 | 9 | 6 |
| 686 | \|A0A151MAF8\|A0A151MAF8_ALLMI Uncharacterized protein OS=Alligator mississippiensis GN=Y1Q_0001678 PE=4 SV=1 | 20996905,7 | 10 | 6 |
| 687 | \|A0A1U7SEF3\|A0A1U7SEF3_ALLSI histidine--tRNA ligase, cytoplasmic OS=Alligator sinensis GN=HARS PE=3 SV=1 | 20968159,3 | 17 | 10 |
| 688 | \|A0A151N5J4\|A0A151N5J4_ALLMI Zinc finger ZZ-type and EF-hand domain-containing protein 1 OS=Alligator mississippiensis GN=ZZEF1 PE=4 SV=1 | 20872381,5 | 6 | 4 |
| 689 | \|A0A1U7RWN1\|A0A1U7RWN1_ALLSI septin-2 OS=Alligator sinensis GN=LOC102369897 PE=3 SV=1 | 20706720,0 | 10 | 5 |
| 690 | \|A0A1U7SIE1\|A0A1U7SIE1_ALLSI isochorismatase domain-containing protein 1 OS=Alligator sinensis GN=ISOC1 PE=4 SV=1 | 20675665,9 | 8 | 5 |
| 691 | \|A0A151MHP1\|A0A151MHP1_ALLMI Olfactomedin-4 OS=Alligator mississippiensis GN=OLFM4 PE=4 SV=1 | 20609338,4 | 2 | 2 |
| 692 | \|A0A151MHD7\|A0A151MHD7_ALLMI Deoxynucleoside triphosphate triphosphohydrolase SAMHD1 OS=Alligator mississippiensis GN=SAMHD1 PE=4 SV=1 | 20504876,1 | 10 | 7 |
| 693 | \|A0A1U8D8C0\|A0A1U8D8C0_ALLSI glycerol kinase OS=Alligator sinensis GN=GK PE=3 SV=1 | 20481971,6 | 12 | 7 |
| 694 | \|A0A151NJT0\|A0A151NJT0_ALLMI Ribosomal protein S6 kinase OS=Alligator mississippiensis GN=RPS6KA1 PE=3 SV=1 | 20396903,0 | 9 | 7 |
| 695 | \|A0A151MDY6\|A0A151MDY6_ALLMI T-complex protein 1 subunit theta OS=Alligator mississippiensis GN=CCT8 PE=3 SV=1 | 20348784,9 | 6 | 2 |
| 696 | \|A0A1U7S899\|A0A1U7S899_ALLSI transcobalamin-1-like OS=Alligator sinensis GN=LOC102386187 PE=4 SV=1 | 20326835,5 | 4 | 1 |
| 697 | \|A0A151P8V8\|A0A151P8V8_ALLMI Alpha-1-antitrypsin-like OS=Alligator mississippiensis GN=Y1Q_0015124 PE=3 SV=1 | 20222856,4 | 4 | 4 |
| 698 | \|A0A151ND53\|A0A151ND53_ALLMI Desmoplakin OS=Alligator mississippiensis GN=DSP PE=4 SV=1 | 20217742,5 | 10 | 8 |
| 699 | \|A0A151MWR8\|A0A151MWR8_ALLMI Uncharacterized protein OS=Alligator mississippiensis GN=Y1Q_0009801 PE=3 SV=1 | 20141247,5 | 11 | 7 |
| 700 | \|A0A151MTB9\|A0A151MTB9_ALLMI Adipogenesis regulatory factor OS=Alligator mississippiensis GN=ADIRF PE=4 SV=1 | 20050656,2 | 9 | 3 |
| 701 | \|A0A151P4Z7\|A0A151P4Z7_ALLMI von Willebrand factor A domain-containing protein 5A OS=Alligator mississippiensis GN=VWA5A PE=4 SV=1 | 20039564,9 | 1 | 1 |
| 702 | \|A0A151NVR8\|A0A151NVR8_ALLMI Uncharacterized protein OS=Alligator mississippiensis GN=Y1Q_0007656 PE=3 SV=1 | 20035430,0 | 6 | 3 |
| 703 | \|A0A151PJH7\|A0A151PJH7_ALLMI Protein S100 OS=Alligator mississippiensis GN=S100A9 PE=3 SV=1 | 20000123,1 | 6 | 2 |
| 704 | \|A0A1U7S1W8\|A0A1U7S1W8_ALLSI asparagine synthetase [glutamine-hydrolyzing] isoform X1 OS=Alligator sinensis GN=ASNS PE=4 SV=1 | 19979602,9 | 13 | 6 |
| 705 | \|A0A1U7SE44\|A0A1U7SE44_ALLSI IST1 homolog OS=Alligator sinensis GN=IST1 PE=4 SV=1 | 19874310,4 | 7 | 5 |
| 706 | \|A0A151P1X6\|A0A151P1X6_ALLMI Uncharacterized protein OS=Alligator mississippiensis GN=Y1Q_0016672 PE=3 SV=1 | 19870887,1 | 9 | 7 |
| 707 | \|A0A151MLE3\|A0A151MLE3_ALLMI Tubulin-folding cofactor B OS=Alligator mississippiensis GN=TBCB PE=4 SV=1 | 19866454,9 | 5 | 4 |
| 708 | \|A9CPF9\|A9CPF9_ALLMI Heat shock protein 75 OS=Alligator mississippiensis GN=HSP75 PE=2 SV=1 | 19864973,9 | 4 | 1 |
| 709 | \|A0A151MTX0\|A0A151MTX0_ALLMI Heterogeneous nuclear ribonucleoprotein R OS=Alligator mississippiensis GN=HNRNPR PE=4 SV=1 | 19779668,2 | 4 | 3 |
| 710 | \|A0A151M1G9\|A0A151M1G9_ALLMI Alpha-1,4 glucan phosphorylase OS=Alligator mississippiensis GN=Y1Q_0008491 PE=3 SV=1 | 19737352,3 | 9 | 4 |
| 711 | \|A0A151PIQ7\|A0A151PIQ7_ALLMI Protein S100 OS=Alligator mississippiensis GN=S100A6 PE=3 SV=1 | 19493950,5 | 5 | 2 |
| 712 | \|A0A1U7S3G3\|A0A1U7S3G3_ALLSI Sorting nexin OS=Alligator sinensis GN=SNX5 PE=3 SV=1 | 19487497,3 | 9 | 7 |
| 713 | \|A0A151MG37\|A0A151MG37_ALLMI Proteasome subunit beta type OS=Alligator mississippiensis GN=PSMB7 PE=3 SV=1 | 19446649,0 | 4 | 2 |
| 714 | \|A0A151NQQ4\|A0A151NQQ4_ALLMI Prefoldin subunit 1 OS=Alligator mississippiensis GN=PFDN1 PE=4 SV=1 | 19396551,5 | 5 | 4 |
| 715 | \|A0A151N1Z6\|A0A151N1Z6_ALLMI COP9 signalosome complex subunit 3 OS=Alligator mississippiensis GN=COPS3 PE=4 SV=1 | 19319247,1 | 4 | 4 |
| 716 | \|A0A151PFM9\|A0A151PFM9_ALLMI F-actin-capping protein subunit alpha-1 OS=Alligator mississippiensis GN=CAPZA1-1 PE=3 SV=1 | 19315661,5 | 3 | 2 |
| 717 | \|A0A151MTI1\|A0A151MTI1_ALLMI Dystroglycan OS=Alligator mississippiensis GN=DAG1 PE=4 SV=1 | 19267567,6 | 4 | 1 |
| 718 | \|A0A1U7S8N5\|A0A1U7S8N5_ALLSI protein phosphatase 1A OS=Alligator sinensis GN=PPM1A PE=3 SV=1 | 19247496,3 | 8 | 5 |
| 719 | \|A0A151NTL8\|A0A151NTL8_ALLMI Proteasome subunit beta type OS=Alligator mississippiensis GN=PSMB6 PE=3 SV=1 | 19191907,3 | 9 | 6 |
| 720 | \|A0A151N9G1\|A0A151N9G1_ALLMI LanC-like protein 1 OS=Alligator mississippiensis GN=LANCL1 PE=4 SV=1 | 19077856,7 | 12 | 7 |
| 721 | \|A0A151P540\|A0A151P540_ALLMI Prefoldin subunit 5 OS=Alligator mississippiensis GN=PFDN5 PE=3 SV=1 | 19065179,3 | 4 | 2 |
| 722 | \|A0A1U7RJA2\|A0A1U7RJA2_ALLSI transcription elongation factor B polypeptide 1 OS=Alligator sinensis GN=TCEB1 PE=3 SV=1 | 19033384,9 | 8 | 5 |
| 723 | \|A0A151N0Y4\|A0A151N0Y4_ALLMI AP-2 complex subunit alpha-2 isoform A OS=Alligator mississippiensis GN=AP2A2 PE=4 SV=1 | 18982001,9 | 13 | 10 |
| 724 | \|A0A151N6E9\|A0A151N6E9_ALLMI Galectin OS=Alligator mississippiensis GN=LGALS9 PE=4 SV=1 | 18981069,4 | 8 | 3 |
| 725 | \|A0A151NSF8\|A0A151NSF8_ALLMI Vacuolar protein sorting-associated protein 29 OS=Alligator mississippiensis GN=VPS29 PE=3 SV=1 | 18954504,2 | 6 | 3 |
| 726 | \|A0A151M202\|A0A151M202_ALLMI Uncharacterized protein OS=Alligator mississippiensis GN=Y1Q_0014804 PE=3 SV=1 | 18789276,8 | 4 | 1 |
| 727 | \|A0A1U7S2Q0\|A0A1U7S2Q0_ALLSI SUMO-activating enzyme subunit 2 OS=Alligator sinensis GN=UBA2 PE=4 SV=1 | 18624600,9 | 7 | 5 |
| 728 | \|A0A151N6D6\|A0A151N6D6_ALLMI Pyruvate kinase OS=Alligator mississippiensis GN=Y1Q_0000374 PE=3 SV=1 | 18602746,7 | 3 | 1 |
| 729 | \|A0A1U7RDM8\|A0A1U7RDM8_ALLSI Inositol-1-monophosphatase OS=Alligator sinensis GN=IMPA1 PE=3 SV=1 | 18491417,9 | 11 | 5 |
| 730 | \|A0A151PI29\|A0A151PI29_ALLMI 40S ribosomal protein S23 isoform A OS=Alligator mississippiensis GN=RPS23 PE=3 SV=1 | 18417925,8 | 6 | 4 |
| 731 | \|A0A151MK37\|A0A151MK37_ALLMI Coatomer subunit gamma OS=Alligator mississippiensis GN=Y1Q_0023707 PE=3 SV=1 | 18223608,1 | 9 | 7 |
| 732 | \|A0A151NQJ9\|A0A151NQJ9_ALLMI Proteasome subunit beta type-5 OS=Alligator mississippiensis GN=PSMB5 PE=4 SV=1 | 18079124,0 | 4 | 4 |
| 733 | \|A0A1U8DVC9\|A0A1U8DVC9_ALLSI exportin-2 isoform X2 OS=Alligator sinensis GN=CSE1L PE=4 SV=1 | 17949026,0 | 11 | 9 |
| 734 | \|A0A1U7S3T7\|A0A1U7S3T7_ALLSI keratin, type I cytoskeletal 18 OS=Alligator sinensis GN=KRT18 PE=3 SV=1 | 17887586,2 | 4 | 3 |
| 735 | \|A0A1U7SID2\|A0A1U7SID2_ALLSI Vacuolar protein sorting-associated protein 28 homolog OS=Alligator sinensis GN=VPS28 PE=3 SV=1 | 17784226,1 | 4 | 4 |
| 736 | \|A0A151M9U0\|A0A151M9U0_ALLMI Uncharacterized protein OS=Alligator mississippiensis GN=Y1Q_0001479 PE=4 SV=1 | 17775970,1 | 10 | 8 |
| 737 | \|A0A1U7RQB3\|A0A1U7RQB3_ALLSI angiotensinogen OS=Alligator sinensis GN=AGT PE=3 SV=1 | 17751096,8 | 8 | 6 |
| 738 | \|A0A1U8DS59\|A0A1U8DS59_ALLSI putative V-set and immunoglobulin domain-containing-like protein IGHV4OR15-8 OS=Alligator sinensis GN=LOC102382546 PE=4 SV=1 | 17702008,4 | 6 | 2 |
| 739 | \|A0A1U7S5A2\|A0A1U7S5A2_ALLSI flavin reductase (NADPH) OS=Alligator sinensis GN=BLVRB PE=4 SV=1 | 17672553,9 | 10 | 5 |
| 740 | \|A0A1U8DJ93\|A0A1U8DJ93_ALLSI EH domain-binding protein 1 OS=Alligator sinensis GN=EHBP1 PE=4 SV=1 | 17668564,4 | 2 | 2 |
| 741 | \|A0A1U7RU69\|A0A1U7RU69_ALLSI Polyadenylate-binding protein OS=Alligator sinensis GN=PABPC1 PE=3 SV=1 | 17652492,7 | 16 | 11 |
| 742 | \|A0A151P8W5\|A0A151P8W5_ALLMI RNA-binding protein 3 OS=Alligator mississippiensis GN=RBM3 PE=4 SV=1 | 17646256,6 | 13 | 4 |
| 743 | \|A0A151NXN5\|A0A151NXN5_ALLMI Dual specificity mitogen-activated protein kinase kinase 1 OS=Alligator mississippiensis GN=MAP2K1 PE=3 SV=1 | 17561345,5 | 7 | 4 |
| 744 | \|A0A1U7SM42\|A0A1U7SM42_ALLSI 40S ribosomal protein S12 OS=Alligator sinensis GN=RPS12 PE=3 SV=1 | 17555115,0 | 7 | 4 |
| 745 | \|A0A151NNL6\|A0A151NNL6_ALLMI Malignant T-cell-amplified sequence OS=Alligator mississippiensis GN=MCTS1 PE=3 SV=1 | 17523018,8 | 9 | 4 |
| 746 | \|A0A151P0H5\|A0A151P0H5_ALLMI Uncharacterized protein OS=Alligator mississippiensis GN=Y1Q_0000291 PE=4 SV=1 | 17497101,9 | 10 | 7 |
| 747 | \|A0A1U8D920\|A0A1U8D920_ALLSI 60S ribosomal protein L28 OS=Alligator sinensis GN=RPL28 PE=4 SV=1 | 17455558,0 | 5 | 3 |
| 748 | \|A0A151LYZ5\|A0A151LYZ5_ALLMI Heme-binding protein 1 OS=Alligator mississippiensis GN=HEBP1 PE=4 SV=1 | 17448950,2 | 5 | 3 |
| 749 | \|A0A1U8DX19\|A0A1U8DX19_ALLSI hypoxia up-regulated protein 1 OS=Alligator sinensis GN=HYOU1 PE=3 SV=1 | 17415741,6 | 8 | 6 |
| 750 | \|A0A151NKB4\|A0A151NKB4_ALLMI Uncharacterized protein OS=Alligator mississippiensis GN=Y1Q_0008930 PE=4 SV=1 | 17354638,5 | 8 | 6 |
| 751 | \|A0A151NTC3\|A0A151NTC3_ALLMI Caspase-1 OS=Alligator mississippiensis GN=Y1Q_0013318 PE=3 SV=1 | 17260809,9 | 6 | 4 |
| 752 | \|A0A151NIQ7\|A0A151NIQ7_ALLMI Myotrophin OS=Alligator mississippiensis GN=MTPN PE=4 SV=1 | 17142593,3 | 16 | 6 |
| 753 | \|A0A151N7V1\|A0A151N7V1_ALLMI Low molecular weight phosphotyrosine protein phosphatase isoform A OS=Alligator mississippiensis GN=ACP1-1 PE=4 SV=1 | 17139189,6 | 4 | 2 |
| 754 | \|A0A151MR19\|A0A151MR19_ALLMI 60S ribosomal protein L14 OS=Alligator mississippiensis GN=RPL14 PE=4 SV=1 | 17037844,9 | 8 | 3 |
| 755 | \|A0A1U8D573\|A0A1U8D573_ALLSI inter-alpha-trypsin inhibitor heavy chain H3 isoform X3 OS=Alligator sinensis GN=LOC102387098 PE=4 SV=1 | 16989808,8 | 4 | 3 |
| 756 | \|A0A1U7S6L7\|A0A1U7S6L7_ALLSI RNA-binding motif protein, X chromosome isoform X1 OS=Alligator sinensis GN=RBMX PE=4 SV=1 | 16897116,5 | 4 | 2 |
| 757 | \|A0A1U8DL32\|A0A1U8DL32_ALLSI guanylate kinase isoform X2 OS=Alligator sinensis GN=GUK1 PE=4 SV=1 | 16885380,8 | 3 | 2 |
| 758 | \|A0A151MY00\|A0A151MY00_ALLMI 60S ribosomal protein L30 OS=Alligator mississippiensis GN=RPL30 PE=3 SV=1 | 16835864,0 | 8 | 4 |
| 759 | \|A0A151MRJ1\|A0A151MRJ1_ALLMI Prefoldin subunit 2 OS=Alligator mississippiensis GN=PFDN2 PE=4 SV=1 | 16824639,3 | 9 | 3 |
| 760 | \|A0A151PHF6\|A0A151PHF6_ALLMI Endoplasmic reticulum resident protein 44 OS=Alligator mississippiensis GN=ERP44 PE=4 SV=1 | 16789051,8 | 3 | 2 |
| 761 | \|A0A151NQC9\|A0A151NQC9_ALLMI Thymocyte nuclear protein 1 OS=Alligator mississippiensis GN=THYN1 PE=4 SV=1 | 16501929,7 | 14 | 8 |
| 762 | \|A0A151NE88\|A0A151NE88_ALLMI SAP domain-containing ribonucleoprotein OS=Alligator mississippiensis GN=SARNP PE=4 SV=1 | 16488876,2 | 2 | 1 |
| 763 | \|A0A151MRV9\|A0A151MRV9_ALLMI DnaJ-like protein subfamily A member 2 OS=Alligator mississippiensis GN=DNAJA2 PE=3 SV=1 | 16488100,8 | 4 | 3 |
| 764 | \|A0A1U7RMI7\|A0A1U7RMI7_ALLSI triokinase/FMN cyclase OS=Alligator sinensis GN=TKFC PE=4 SV=1 | 16388479,7 | 11 | 5 |
| 765 | \|A0A151NHW8\|A0A151NHW8_ALLMI tRNA-splicing ligase RtcB homolog OS=Alligator mississippiensis GN=RTCB PE=3 SV=1 | 16326521,1 | 12 | 7 |
| 766 | \|A0A151MST8\|A0A151MST8_ALLMI Annexin OS=Alligator mississippiensis GN=ANXA7 PE=3 SV=1 | 16282732,6 | 8 | 8 |
| 767 | \|A0A1U7RYE0\|A0A1U7RYE0_ALLSI transcobalamin-1-like OS=Alligator sinensis GN=LOC102372472 PE=4 SV=1 | 16279950,3 | 5 | 4 |
| 768 | \|A0A1U8DA80\|A0A1U8DA80_ALLSI cordon-bleu protein-like 1 OS=Alligator sinensis GN=COBLL1 PE=4 SV=1 | 16277870,1 | 3 | 3 |
| 769 | \|A0A1U8DBU1\|A0A1U8DBU1_ALLSI LIM domain and actin-binding protein 1 isoform X1 OS=Alligator sinensis GN=LIMA1 PE=4 SV=1 | 16153205,3 | 12 | 8 |
| 770 | \|A0A151NK01\|A0A151NK01_ALLMI Nuclear localization protein 4-like protein OS=Alligator mississippiensis GN=NPLOC4 PE=4 SV=1 | 16147190,8 | 7 | 4 |
| 771 | \|A0A1U7SNP1\|A0A1U7SNP1_ALLSI phosphoglucomutase-1 isoform X2 OS=Alligator sinensis GN=PGM1 PE=3 SV=1 | 16145165,5 | 1 | 1 |
| 772 | \|A0A151P466\|A0A151P466_ALLMI Mitotic checkpoint protein BUB3 OS=Alligator mississippiensis GN=BUB3 PE=4 SV=1 | 16134096,4 | 6 | 4 |
| 773 | \|A0A1U7RPU1\|A0A1U7RPU1_ALLSI protein 4.1 isoform X3 OS=Alligator sinensis GN=EPB41 PE=4 SV=1 | 16106416,0 | 9 | 6 |
| 774 | \|A0A151MP94\|A0A151MP94_ALLMI Uncharacterized protein OS=Alligator mississippiensis GN=Y1Q_0018595 PE=4 SV=1 | 16075078,8 | 7 | 1 |
| 775 | \|A0A1U7SLA8\|A0A1U7SLA8_ALLSI vacuolar protein sorting-associated protein 4B OS=Alligator sinensis GN=VPS4B PE=3 SV=1 | 16061670,3 | 6 | 5 |
| 776 | \|A0A151MZC6\|A0A151MZC6_ALLMI Cellular nucleic acid-binding protein OS=Alligator mississippiensis GN=CNBP PE=4 SV=1 | 15969458,6 | 5 | 3 |
| 777 | \|A0A151NLB0\|A0A151NLB0_ALLMI Uncharacterized protein OS=Alligator mississippiensis GN=Y1Q_0008962 PE=4 SV=1 | 15954689,1 | 5 | 4 |
| 778 | \|A0A151PFJ2\|A0A151PFJ2_ALLMI 60S ribosomal protein L18a OS=Alligator mississippiensis GN=RPL18A PE=3 SV=1 | 15808486,6 | 5 | 4 |
| 779 | \|A0A151NYA5\|A0A151NYA5_ALLMI Ras-related protein Rab-8A isoform B OS=Alligator mississippiensis GN=RAB8A-1 PE=4 SV=1 | 15759036,9 | 3 | 3 |
| 780 | \|A0A1U8D0P2\|A0A1U8D0P2_ALLSI DIS3-like exonuclease 2 OS=Alligator sinensis GN=DIS3L2 PE=3 SV=1 | 15706934,2 | 5 | 4 |
| 781 | \|A0A151MNY7\|A0A151MNY7_ALLMI Sushi, nidogen and EGF-like domain-containing protein 1 OS=Alligator mississippiensis GN=Y1Q_0013078 PE=4 SV=1 | 15663392,3 | 3 | 2 |
| 782 | \|A0A1U7SAN8\|A0A1U7SAN8_ALLSI Oxysterol-binding protein OS=Alligator sinensis GN=OSBP PE=3 SV=1 | 15618440,4 | 9 | 8 |
| 783 | \|A0A1U7S1Q9\|A0A1U7S1Q9_ALLSI caspase-7 isoform X1 OS=Alligator sinensis GN=CASP7 PE=3 SV=1 | 15519565,1 | 18 | 9 |
| 784 | \|A0A1U7RTP4\|A0A1U7RTP4_ALLSI serum albumin-like OS=Alligator sinensis GN=LOC102376438 PE=3 SV=1 | 15397912,3 | 4 | 2 |
| 785 | \|A0A1U7SH80\|A0A1U7SH80_ALLSI Leukotriene A(4) hydrolase OS=Alligator sinensis GN=LTA4H PE=3 SV=1 | 15362504,4 | 9 | 4 |
| 786 | \|A0A151N1D4\|A0A151N1D4_ALLMI Pyridoxal-dependent decarboxylase domain-containing protein 1 OS=Alligator mississippiensis GN=PDXDC1 PE=4 SV=1 | 15281106,5 | 7 | 6 |
| 787 | \|A0A1U8DCT8\|A0A1U8DCT8_ALLSI E3 ubiquitin-protein ligase UBR4 OS=Alligator sinensis GN=UBR4 PE=4 SV=1 | 15268650,2 | 10 | 10 |
| 788 | \|A0A1U7RFE6\|A0A1U7RFE6_ALLSI KN motif and ankyrin repeat domain-containing protein 1 isoform X1 OS=Alligator sinensis GN=KANK1 PE=4 SV=1 | 15185995,0 | 5 | 3 |
| 789 | \|A0A151P1Z7\|A0A151P1Z7_ALLMI Syntenin-2 OS=Alligator mississippiensis GN=SDCBP2 PE=4 SV=1 | 15167668,0 | 3 | 2 |
| 790 | \|A0A151MFH6\|A0A151MFH6_ALLMI Serine/threonine-protein phosphatase 2A activator OS=Alligator mississippiensis GN=PPP2R4 PE=3 SV=1 | 15085986,5 | 16 | 10 |
| 791 | \|A0A151NRH3\|A0A151NRH3_ALLMI Diaphanous-like protein 2 isoform B OS=Alligator mississippiensis GN=DIAPH1-1 PE=4 SV=1 | 15075038,2 | 6 | 4 |
| 792 | \|A0A151MCG3\|A0A151MCG3_ALLMI Cathepsin D OS=Alligator mississippiensis GN=CTSD PE=3 SV=1 | 15066948,8 | 5 | 3 |
| 793 | \|A0A151P3A8\|A0A151P3A8_ALLMI BRO1 domain-containing protein BROX OS=Alligator mississippiensis GN=BROX PE=4 SV=1 | 15064055,7 | 5 | 4 |
| 794 | \|A0A1U8D5U4\|A0A1U8D5U4_ALLSI Signal transducer and activator of transcription OS=Alligator sinensis GN=LOC102380420 PE=3 SV=1 | 15035655,2 | 6 | 3 |
| 795 | \|A0A151MF35\|A0A151MF35_ALLMI Aminoacylase-1 OS=Alligator mississippiensis GN=ACY1 PE=4 SV=1 | 15026431,3 | 4 | 2 |
| 796 | \|A0A151PK60\|A0A151PK60_ALLMI 26S proteasome non-ATPase regulatory subunit 8 OS=Alligator mississippiensis GN=PSMD8 PE=4 SV=1 | 14974923,0 | 8 | 6 |
| 797 | \|A0A151MBJ7\|A0A151MBJ7_ALLMI Methylthioribulose-1-phosphate dehydratase OS=Alligator mississippiensis GN=APIP PE=3 SV=1 | 14967292,4 | 5 | 3 |
| 798 | \|A0A1U8D4M1\|A0A1U8D4M1_ALLSI alpha-2-macroglobulin-like protein 1 OS=Alligator sinensis GN=LOC106721822 PE=4 SV=1 | 14956090,8 | 10 | 3 |
| 799 | \|A0A1U7RPL3\|A0A1U7RPL3_ALLSI quinone oxidoreductase isoform X2 OS=Alligator sinensis GN=CRYZ PE=4 SV=1 | 14921303,9 | 5 | 3 |
| 800 | \|A0A151NGQ8\|A0A151NGQ8_ALLMI Protein syndesmos OS=Alligator mississippiensis GN=NUDT16L1 PE=4 SV=1 | 14847401,9 | 4 | 3 |
| 801 | \|A0A151N230\|A0A151N230_ALLMI Eukaryotic translation initiation factor 3 subunit B OS=Alligator mississippiensis GN=EIF3B PE=3 SV=1 | 14842367,6 | 10 | 7 |
| 802 | \|A0A151MMD1\|A0A151MMD1_ALLMI UV excision repair RAD23-like protein A OS=Alligator mississippiensis GN=RAD23A PE=4 SV=1 | 14816587,0 | 3 | 2 |
| 803 | \|A0A151MKS7\|A0A151MKS7_ALLMI cAMP-dependent protein kinase type II-alpha regulatory subunit OS=Alligator mississippiensis GN=PRKAR2A PE=4 SV=1 | 14815551,7 | 6 | 4 |
| 804 | \|A0A151PFQ0\|A0A151PFQ0_ALLMI Eukaryotic translation initiation factor 3 subunit G OS=Alligator mississippiensis GN=EIF3G PE=3 SV=1 | 14813834,3 | 5 | 4 |
| 805 | \|A0A1U7S1Z6\|A0A1U7S1Z6_ALLSI 60S ribosomal protein L32 OS=Alligator sinensis GN=RPL32 PE=4 SV=1 | 14788644,4 | 7 | 4 |
| 806 | \|A0A151MDF7\|A0A151MDF7_ALLMI Protein TFG OS=Alligator mississippiensis GN=TFG PE=4 SV=1 | 14755162,6 | 5 | 1 |
| 807 | \|A0A151PFQ7\|A0A151PFQ7_ALLMI 40S ribosomal protein S16 OS=Alligator mississippiensis GN=RPS16 PE=3 SV=1 | 14737500,8 | 11 | 8 |
| 808 | \|A0A1U8DIJ1\|A0A1U8DIJ1_ALLSI interferon-induced GTP-binding protein Mx1 isoform X1 OS=Alligator sinensis GN=MX1 PE=3 SV=1 | 14648853,1 | 9 | 7 |
| 809 | \|A0A1U8D5Q2\|A0A1U8D5Q2_ALLSI general vesicular transport factor p115 isoform X4 OS=Alligator sinensis GN=USO1 PE=4 SV=1 | 14627900,3 | 13 | 7 |
| 810 | \|A0A151MER1\|A0A151MER1_ALLMI Poly [ADP-ribose] polymerase OS=Alligator mississippiensis GN=PARP3 PE=4 SV=1 | 14621700,5 | 3 | 2 |
| 811 | \|A0A151NC06\|A0A151NC06_ALLMI Methionine--tRNA ligase, cytoplasmic OS=Alligator mississippiensis GN=MARS PE=3 SV=1 | 14597624,0 | 4 | 3 |
| 812 | \|A0A1U7RXF1\|A0A1U7RXF1_ALLSI Sulfotransferase OS=Alligator sinensis GN=LOC102375551 PE=3 SV=1 | 14574965,5 | 2 | 1 |
| 813 | \|A0A1U8D6F9\|A0A1U8D6F9_ALLSI small ubiquitin-related modifier 2 OS=Alligator sinensis GN=SUMO2 PE=4 SV=1 | 14392049,0 | 2 | 2 |
| 814 | \|A0A1U7RR37\|A0A1U7RR37_ALLSI ATP-dependent RNA helicase DDX1 OS=Alligator sinensis GN=DDX1 PE=4 SV=1 | 14391396,9 | 10 | 7 |
| 815 | \|A0A151P816\|A0A151P816_ALLMI 40S ribosomal protein S4 OS=Alligator mississippiensis GN=RPS4X PE=3 SV=1 | 14372963,7 | 9 | 7 |
| 816 | \|A0A151NR69\|A0A151NR69_ALLMI Interferon-inducible GTPase 5-like OS=Alligator mississippiensis GN=Y1Q_0022070 PE=4 SV=1 | 14299962,4 | 6 | 4 |
| 817 | \|A0A151PBQ2\|A0A151PBQ2_ALLMI Synaptic vesicle membrane VAT-1-like protein OS=Alligator mississippiensis GN=VAT1 PE=4 SV=1 | 14294548,4 | 9 | 7 |
| 818 | \|A0A151NYX9\|A0A151NYX9_ALLMI Breast carcinoma-amplified sequence 1 isoform A OS=Alligator mississippiensis GN=BCAS1 PE=4 SV=1 | 14276873,6 | 5 | 2 |
| 819 | \|A0A1U8D364\|A0A1U8D364_ALLSI kynurenine--oxoglutarate transaminase 1 isoform X3 OS=Alligator sinensis GN=CCBL1 PE=4 SV=1 | 14265375,5 | 11 | 6 |
| 820 | \|A0A1U8DIA7\|A0A1U8DIA7_ALLSI isoleucine--tRNA ligase, cytoplasmic isoform X2 OS=Alligator sinensis GN=IARS PE=3 SV=1 | 14254192,4 | 9 | 8 |
| 821 | \|A0A151NL66\|A0A151NL66_ALLMI Tubulin beta chain OS=Alligator mississippiensis GN=Y1Q_0015774 PE=3 SV=1 | 14231563,8 | 10 | 5 |
| 822 | \|A0A1U7SVU4\|A0A1U7SVU4_ALLSI COP9 signalosome complex subunit 4 OS=Alligator sinensis GN=LOC102386320 PE=4 SV=1 | 14221425,9 | 4 | 3 |
| 823 | \|A0A151N6K4\|A0A151N6K4_ALLMI Uncharacterized protein OS=Alligator mississippiensis GN=Y1Q_0008901 PE=4 SV=1 | 14213908,1 | 6 | 3 |
| 824 | \|A0A151NW88\|A0A151NW88_ALLMI Cathepsin B OS=Alligator mississippiensis GN=CTSB PE=3 SV=1 | 14151207,7 | 2 | 1 |
| 825 | \|A0A151N8L6\|A0A151N8L6_ALLMI Mitogen-activated protein kinase OS=Alligator mississippiensis GN=MAPK14 PE=4 SV=1 | 14028933,2 | 7 | 5 |
| 826 | \|A0A151NHW6\|A0A151NHW6_ALLMI Eukaryotic translation initiation factor 3 subunit D OS=Alligator mississippiensis GN=EIF3D PE=3 SV=1 | 13973720,7 | 6 | 3 |
| 827 | \|A0A151MCQ7\|A0A151MCQ7_ALLMI Uncharacterized protein OS=Alligator mississippiensis GN=Y1Q_0002916 PE=3 SV=1 | 13963551,6 | 8 | 3 |
| 828 | \|A0A151N4B7\|A0A151N4B7_ALLMI Kelch-like protein 22 OS=Alligator mississippiensis GN=KLHL22 PE=4 SV=1 | 13798394,0 | 2 | 1 |
| 829 | \|A0A151NIJ6\|A0A151NIJ6_ALLMI Poly [ADP-ribose] polymerase 12 isoform A OS=Alligator mississippiensis GN=PARP12 PE=4 SV=1 | 13704071,3 | 5 | 4 |
| 830 | \|A0A1U7RYZ9\|A0A1U7RYZ9_ALLSI 60S ribosomal protein L12 OS=Alligator sinensis GN=RPL12 PE=3 SV=1 | 13577077,9 | 13 | 6 |
| 831 | \|A0A1U7S834\|A0A1U7S834_ALLSI arf-GAP with dual PH domain-containing protein 1-like isoform X1 OS=Alligator sinensis GN=LOC102386672 PE=4 SV=1 | 13573287,6 | 9 | 6 |
| 832 | \|A0A1U7RM78\|A0A1U7RM78_ALLSI glutaredoxin-3 OS=Alligator sinensis GN=GLRX3 PE=4 SV=1 | 13568708,9 | 14 | 6 |
| 833 | \|A0A151PJ24\|A0A151PJ24_ALLMI Interleukin enhancer-binding factor 2 OS=Alligator mississippiensis GN=ILF2 PE=4 SV=1 | 13502705,4 | 13 | 5 |
| 834 | \|A0A151LY43\|A0A151LY43_ALLMI Drebrin-like protein isoform A OS=Alligator mississippiensis GN=DBNL PE=4 SV=1 | 13497426,1 | 6 | 3 |
| 835 | \|A0A1U8DME2\|A0A1U8DME2_ALLSI cingulin isoform X2 OS=Alligator sinensis GN=CGN PE=4 SV=1 | 13468067,8 | 6 | 5 |
| 836 | \|A0A1U7S048\|A0A1U7S048_ALLSI secernin-2 isoform X1 OS=Alligator sinensis GN=SCRN2 PE=4 SV=1 | 13438335,8 | 5 | 4 |
| 837 | \|A0A151MIY0\|A0A151MIY0_ALLMI Sulfotransferase OS=Alligator mississippiensis GN=Y1Q_0002097 PE=3 SV=1 | 13302455,5 | 5 | 3 |
| 838 | \|A0A151P985\|A0A151P985_ALLMI Disks large-like protein 1 isoform A OS=Alligator mississippiensis GN=DLG1 PE=4 SV=1 | 13258901,6 | 7 | 4 |
| 839 | \|A0A151MML1\|A0A151MML1_ALLMI Chloride intracellular channel protein OS=Alligator mississippiensis GN=CLIC4-1 PE=3 SV=1 | 13168066,3 | 3 | 2 |
| 840 | \|A0A151P3P4\|A0A151P3P4_ALLMI Echinoderm microtubule-associated protein-like 1 isoform B OS=Alligator mississippiensis GN=EML1 PE=4 SV=1 | 13162690,6 | 9 | 7 |
| 841 | \|A0A1U7RXK1\|A0A1U7RXK1_ALLSI protein S100-A13 OS=Alligator sinensis GN=S100A13 PE=3 SV=1 | 13143842,3 | 4 | 3 |
| 842 | \|A0A151MLX2\|A0A151MLX2_ALLMI 40S ribosomal protein S5 OS=Alligator mississippiensis GN=RPS5 PE=3 SV=1 | 13121310,7 | 7 | 5 |
| 843 | \|A0A151PF44\|A0A151PF44_ALLMI Twinfilin-2 OS=Alligator mississippiensis GN=TWF2 PE=4 SV=1 | 13077074,1 | 6 | 3 |
| 844 | \|A0A1U7SR71\|A0A1U7SR71_ALLSI tryptophan--tRNA ligase, cytoplasmic OS=Alligator sinensis GN=WARS PE=3 SV=1 | 13054526,9 | 5 | 3 |
| 845 | \|A0A151NPC7\|A0A151NPC7_ALLMI S-adenosylmethionine synthase OS=Alligator mississippiensis GN=Y1Q_0007449 PE=3 SV=1 | 12907069,8 | 9 | 5 |
| 846 | \|A0A151M519\|A0A151M519_ALLMI 60S ribosomal protein L5 OS=Alligator mississippiensis GN=RPL5 PE=3 SV=1 | 12875715,4 | 6 | 4 |
| 847 | \|A0A151N171\|A0A151N171_ALLMI Eukaryotic peptide chain release factor GTP-binding subunit ERF3A OS=Alligator mississippiensis GN=GSPT1 PE=4 SV=1 | 12812383,6 | 4 | 2 |
| 848 | \|A0A151NHD5\|A0A151NHD5_ALLMI Alpha-N-acetylgalactosaminidase OS=Alligator mississippiensis GN=NAGA PE=3 SV=1 | 12806369,0 | 4 | 2 |
| 849 | \|A0A151NRJ2\|A0A151NRJ2_ALLMI Tripeptidyl-peptidase 2 isoform A OS=Alligator mississippiensis GN=TPP2 PE=4 SV=1 | 12783895,7 | 11 | 10 |
| 850 | \|A0A151ML13\|A0A151ML13_ALLMI Uncharacterized protein OS=Alligator mississippiensis GN=Y1Q_0009014 PE=4 SV=1 | 12725560,0 | 4 | 3 |
| 851 | \|A0A151MYG2\|A0A151MYG2_ALLMI Attractin-like protein 1 isoform B OS=Alligator mississippiensis GN=ATRNL1 PE=4 SV=1 | 12715629,7 | 5 | 4 |
| 852 | \|A0A151NZX5\|A0A151NZX5_ALLMI Prosaposin OS=Alligator mississippiensis GN=PSAP-2 PE=4 SV=1 | 12707288,1 | 4 | 2 |
| 853 | \|A0A151N0X7\|A0A151N0X7_ALLMI SH3 domain-binding glutamic acid-rich-like protein 3 OS=Alligator mississippiensis GN=SH3BGRL3 PE=4 SV=1 | 12707170,0 | 2 | 1 |
| 854 | \|A0A151NE83\|A0A151NE83_ALLMI Fructose-bisphosphate aldolase OS=Alligator mississippiensis GN=ALDOC PE=3 SV=1 | 12693545,9 | 7 | 5 |
| 855 | \|A0A151MQJ9\|A0A151MQJ9_ALLMI Basic leucine zipper and W2 domain-containing protein 1 OS=Alligator mississippiensis GN=BZW1 PE=4 SV=1 | 12654169,5 | 5 | 2 |
| 856 | \|A0A151NAJ3\|A0A151NAJ3_ALLMI Phenylalanine--tRNA ligase beta subunit OS=Alligator mississippiensis GN=FARSB PE=4 SV=1 | 12624123,8 | 5 | 5 |
| 857 | \|A0A151NVU2\|A0A151NVU2_ALLMI Copine-4 OS=Alligator mississippiensis GN=CPNE4 PE=4 SV=1 | 12545453,9 | 5 | 4 |
| 858 | \|A0A151P4Q1\|A0A151P4Q1_ALLMI Alpha-soluble NSF attachment protein OS=Alligator mississippiensis GN=NAPA PE=4 SV=1 | 12432598,4 | 7 | 4 |
| 859 | \|A0A151N5I7\|A0A151N5I7_ALLMI Methylosome subunit pICln OS=Alligator mississippiensis GN=CLNS1A PE=4 SV=1 | 12393935,5 | 2 | 1 |
| 860 | \|A0A151MX15\|A0A151MX15_ALLMI Succinyl-CoA:3-ketoacid-coenzyme A transferase OS=Alligator mississippiensis GN=OXCT1 PE=3 SV=1 | 12380791,6 | 5 | 5 |
| 861 | \|A0A151N7N1\|A0A151N7N1_ALLMI High mobility group protein B3 OS=Alligator mississippiensis GN=HMGB3 PE=4 SV=1 | 12323402,1 | 4 | 2 |
| 862 | \|A0A151M9X3\|A0A151M9X3_ALLMI Proteasome endopeptidase complex OS=Alligator mississippiensis GN=HYKK PE=3 SV=1 | 12315019,3 | 4 | 3 |
| 863 | \|A0A151NX36\|A0A151NX36_ALLMI Uncharacterized protein OS=Alligator mississippiensis GN=Y1Q_0006266 PE=3 SV=1 | 12267394,7 | 6 | 4 |
| 864 | \|A0A151NMN5\|A0A151NMN5_ALLMI Myosin-7B isoform B OS=Alligator mississippiensis GN=MYH7B PE=3 SV=1 | 12089009,2 | 3 | 2 |
| 865 | \|A0A151NNA0\|A0A151NNA0_ALLMI Synaptotagmin-like protein 2 OS=Alligator mississippiensis GN=Y1Q_0015540 PE=4 SV=1 | 12022618,7 | 7 | 4 |
| 866 | \|A0A151P8H7\|A0A151P8H7_ALLMI Eukaryotic translation initiation factor 5 OS=Alligator mississippiensis GN=EIF5 PE=4 SV=1 | 11982991,2 | 9 | 6 |
| 867 | \|A0A151P075\|A0A151P075_ALLMI Vacuolar protein sorting-associated protein 26A OS=Alligator mississippiensis GN=VPS26A PE=4 SV=1 | 11955628,1 | 5 | 2 |
| 868 | \|A0A151NI23\|A0A151NI23_ALLMI N-alpha-acetyltransferase 15, NatA auxiliary subunit OS=Alligator mississippiensis GN=NAA15 PE=4 SV=1 | 11948349,0 | 5 | 5 |
| 869 | \|A0A151LY40\|A0A151LY40_ALLMI Macrophage-capping protein OS=Alligator mississippiensis GN=CAPG PE=4 SV=1 | 11906922,2 | 5 | 2 |
| 870 | \|A0A151MV92\|A0A151MV92_ALLMI Uncharacterized protein OS=Alligator mississippiensis GN=Y1Q_0015996 PE=3 SV=1 | 11838923,8 | 8 | 5 |
| 871 | \|A0A151MTD6\|A0A151MTD6_ALLMI RNA-binding protein FUS OS=Alligator mississippiensis GN=FUS PE=4 SV=1 | 11838259,1 | 7 | 5 |
| 872 | \|A0A151NDL3\|A0A151NDL3_ALLMI N-alpha-acetyltransferase 10 isoform A OS=Alligator mississippiensis GN=NAA10-1 PE=4 SV=1 | 11785007,8 | 6 | 3 |
| 873 | \|A0A151N2U1\|A0A151N2U1_ALLMI Protein HID1 OS=Alligator mississippiensis GN=HID1 PE=4 SV=1 | 11767216,0 | 4 | 3 |
| 874 | \|A0A151M6P3\|A0A151M6P3_ALLMI Dihydropyrimidinase-related protein 2 isoform A OS=Alligator mississippiensis GN=DPYSL2 PE=4 SV=1 | 11662919,4 | 8 | 7 |
| 875 | \|A0A151M6F5\|A0A151M6F5_ALLMI Methionine adenosyltransferase 2 subunit beta OS=Alligator mississippiensis GN=MAT2B PE=3 SV=1 | 11658759,3 | 6 | 6 |
| 876 | \|A0A151MZM7\|A0A151MZM7_ALLMI Clathrin light chain OS=Alligator mississippiensis GN=CLTB-1 PE=3 SV=1 | 11655415,6 | 4 | 3 |
| 877 | \|A0A151N1E7\|A0A151N1E7_ALLMI Aminoacyl tRNA synthase complex-interacting multifunctional protein 2 isoform B OS=Alligator mississippiensis GN=AIMP2-1 PE=4 SV=1 | 11608888,8 | 3 | 2 |
| 878 | \|A0A151MUK9\|A0A151MUK9_ALLMI Coatomer subunit zeta-1 OS=Alligator mississippiensis GN=COPZ1 PE=4 SV=1 | 11585927,3 | 6 | 3 |
| 879 | \|A0A1U7RPC2\|A0A1U7RPC2_ALLSI Glucosamine-6-phosphate isomerase OS=Alligator sinensis GN=GNPDA1 PE=3 SV=1 | 11546730,4 | 16 | 9 |
| 880 | \|A0A151P595\|A0A151P595_ALLMI Glutaredoxin-3 OS=Alligator mississippiensis GN=GLRX3 PE=4 SV=1 | 11545024,3 | 2 | 1 |
| 881 | \|A0A1U8DIS7\|A0A1U8DIS7_ALLSI heterogeneous nuclear ribonucleoprotein D-like OS=Alligator sinensis GN=HNRNPDL PE=4 SV=1 | 11502535,3 | 6 | 3 |
| 882 | \|A0A1U8DHN6\|A0A1U8DHN6_ALLSI protein phosphatase 1 regulatory subunit 7 OS=Alligator sinensis GN=PPP1R7 PE=4 SV=1 | 11450808,2 | 7 | 7 |
| 883 | \|A0A151NWY9\|A0A151NWY9_ALLMI 60S ribosomal protein L31 OS=Alligator mississippiensis GN=RPL31 PE=4 SV=1 | 11447666,2 | 4 | 3 |
| 884 | \|A0A1U7RYG9\|A0A1U7RYG9_ALLSI 60S ribosomal protein L35a OS=Alligator sinensis GN=RPL35A PE=4 SV=1 | 11432268,7 | 5 | 2 |
| 885 | \|A0A151M5C0\|A0A151M5C0_ALLMI Protein FAM63A OS=Alligator mississippiensis GN=FAM63A PE=4 SV=1 | 11397901,3 | 5 | 3 |
| 886 | \|A0A151N360\|A0A151N360_ALLMI Septin-9 isoform B OS=Alligator mississippiensis GN=SEPT9 PE=3 SV=1 | 11289010,2 | 4 | 4 |
| 887 | \|A0A151MXR4\|A0A151MXR4_ALLMI V-type proton ATPase subunit C OS=Alligator mississippiensis GN=Y1Q_0017986 PE=3 SV=1 | 11147758,0 | 4 | 4 |
| 888 | \|A0A151NMP7\|A0A151NMP7_ALLMI Eukaryotic translation initiation factor 4E OS=Alligator mississippiensis GN=EIF4E PE=3 SV=1 | 11107404,4 | 2 | 2 |
| 889 | \|A0A1U7RJ31\|A0A1U7RJ31_ALLSI anterior gradient protein 3 OS=Alligator sinensis GN=AGR3 PE=4 SV=1 | 10975378,9 | 3 | 1 |
| 890 | \|A0A151PG62\|A0A151PG62_ALLMI Uncharacterized protein OS=Alligator mississippiensis GN=Y1Q_0001898 PE=4 SV=1 | 10959000,3 | 7 | 4 |
| 891 | \|A0A151MZJ5\|A0A151MZJ5_ALLMI 60S ribosomal protein L35 OS=Alligator mississippiensis GN=RPL35 PE=3 SV=1 | 10927239,2 | 3 | 2 |
| 892 | \|A0A1U7S9T4\|A0A1U7S9T4_ALLSI keratin, type II cytoskeletal 6A OS=Alligator sinensis GN=LOC102372665 PE=3 SV=1 | 10896560,2 | 4 | 4 |
| 893 | \|A0A151NZC6\|A0A151NZC6_ALLMI Eukaryotic translation initiation factor 2 subunit 2 OS=Alligator mississippiensis GN=EIF2S2 PE=4 SV=1 | 10883034,1 | 2 | 2 |
| 894 | \|A0A151M7T3\|A0A151M7T3_ALLMI Sorting nexin-1 OS=Alligator mississippiensis GN=SNX1 PE=4 SV=1 | 10880748,1 | 4 | 3 |
| 895 | \|A0A1U8DPW7\|A0A1U8DPW7_ALLSI 60S ribosomal protein L23a OS=Alligator sinensis GN=RPL23A PE=3 SV=1 | 10819438,6 | 4 | 3 |
| 896 | \|A0A151NHQ8\|A0A151NHQ8_ALLMI Eukaryotic translation initiation factor 3 subunit L OS=Alligator mississippiensis GN=EIF3L PE=3 SV=1 | 10776352,7 | 4 | 2 |
| 897 | \|A0A1U7SH11\|A0A1U7SH11_ALLSI mucin-16 OS=Alligator sinensis GN=MUC16 PE=4 SV=1 | 10715710,1 | 4 | 4 |
| 898 | \|A0A151MTM9\|A0A151MTM9_ALLMI Putative helicase MOV-10 OS=Alligator mississippiensis GN=MOV10 PE=4 SV=1 | 10711730,7 | 6 | 4 |
| 899 | \|A0A151MSN0\|A0A151MSN0_ALLMI VAC14-like protein OS=Alligator mississippiensis GN=VAC14 PE=4 SV=1 | 10703940,8 | 4 | 3 |
| 900 | \|A0A151NFE7\|A0A151NFE7_ALLMI Uncharacterized protein OS=Alligator mississippiensis GN=Y1Q_0008100 PE=3 SV=1 | 10679095,8 | 3 | 1 |
| 901 | \|A0A151N5B4\|A0A151N5B4_ALLMI Rabankyrin-5 OS=Alligator mississippiensis GN=ANKFY1 PE=4 SV=1 | 10648742,0 | 5 | 5 |
| 902 | \|A0A151MAX9\|A0A151MAX9_ALLMI Enhancer of rudimentary homolog OS=Alligator mississippiensis GN=ERH PE=3 SV=1 | 10629843,4 | 5 | 2 |
| 903 | \|A0A151NVS2\|A0A151NVS2_ALLMI Peptidyl-prolyl cis-trans isomerase OS=Alligator mississippiensis GN=PIN1 PE=4 SV=1 | 10591487,8 | 5 | 3 |
| 904 | \|A0A151MKE3\|A0A151MKE3_ALLMI NEDD8-activating enzyme E1 catalytic subunit isoform A OS=Alligator mississippiensis GN=UBA3 PE=4 SV=1 | 10591361,7 | 12 | 7 |
| 905 | \|A0A151M2M1\|A0A151M2M1_ALLMI Uncharacterized protein OS=Alligator mississippiensis GN=Y1Q_0009199 PE=3 SV=1 | 10501800,8 | 4 | 3 |
| 906 | \|A0A151NV96\|A0A151NV96_ALLMI 6-phosphogluconolactonase OS=Alligator mississippiensis GN=PGLS PE=4 SV=1 | 10497065,5 | 12 | 5 |
| 907 | \|A0A151NS46\|A0A151NS46_ALLMI Coronin OS=Alligator mississippiensis GN=CORO1B PE=3 SV=1 | 10462196,8 | 10 | 8 |
| 908 | \|A0A1U7STL2\|A0A1U7STL2_ALLSI transforming protein RhoA-like OS=Alligator sinensis GN=LOC102383248 PE=3 SV=1 | 10420069,8 | 4 | 1 |
| 909 | \|A0A151MCG6\|A0A151MCG6_ALLMI 40S ribosomal protein S13 OS=Alligator mississippiensis GN=RPS13 PE=3 SV=1 | 10345519,0 | 6 | 4 |
| 910 | \|A0A151NY32\|A0A151NY32_ALLMI Gastric intrinsic factor-like OS=Alligator mississippiensis GN=Y1Q_0006445 PE=4 SV=1 | 10286920,9 | 4 | 3 |
| 911 | \|A0A1U7SQ36\|A0A1U7SQ36_ALLSI Glutathione peroxidase OS=Alligator sinensis GN=GPX2 PE=3 SV=1 | 10252441,4 | 9 | 5 |
| 912 | \|A0A1U8DD25\|A0A1U8DD25_ALLSI vacuolar protein sorting-associated protein 13C isoform X2 OS=Alligator sinensis GN=VPS13C PE=4 SV=1 | 10211190,2 | 6 | 6 |
| 913 | \|A0A151N8S0\|A0A151N8S0_ALLMI Serine--tRNA ligase, cytoplasmic OS=Alligator mississippiensis GN=SARS PE=4 SV=1 | 10164478,1 | 6 | 5 |
| 914 | \|A0A151P285\|A0A151P285_ALLMI 40S ribosomal protein S6 OS=Alligator mississippiensis GN=RPS6 PE=3 SV=1 | 10124011,5 | 4 | 3 |
| 915 | \|A0A1U7SPR5\|A0A1U7SPR5_ALLSI heterogeneous nuclear ribonucleoprotein D0 OS=Alligator sinensis GN=HNRNPD PE=4 SV=1 | 10101825,8 | 4 | 2 |
| 916 | \|A0A151N2H8\|A0A151N2H8_ALLMI E3 ubiquitin/ISG15 ligase TRIM25 isoform A OS=Alligator mississippiensis GN=TRIM25-1 PE=4 SV=1 | 10059579,4 | 4 | 2 |
| 917 | \|A0A1U7RBM6\|A0A1U7RBM6_ALLSI xylulose kinase isoform X1 OS=Alligator sinensis GN=XYLB PE=4 SV=1 | 9967130,1 | 7 | 6 |
| 918 | \|A0A151MSS7\|A0A151MSS7_ALLMI Enoyl-CoA hydratase, mitochondrial isoform A OS=Alligator mississippiensis GN=ECHS1 PE=3 SV=1 | 9963013,0 | 3 | 2 |
| 919 | \|A0A151NQ16\|A0A151NQ16_ALLMI Uncharacterized protein OS=Alligator mississippiensis GN=Y1Q_0023432 PE=4 SV=1 | 9956393,9 | 3 | 2 |
| 920 | \|A0A151NNZ7\|A0A151NNZ7_ALLMI Transcriptional activator protein Pur-beta OS=Alligator mississippiensis GN=PURB PE=4 SV=1 | 9946181,8 | 2 | 2 |
| 921 | \|A0A151M8R3\|A0A151M8R3_ALLMI NEDD8 OS=Alligator mississippiensis GN=NEDD8 PE=4 SV=1 | 9917319,5 | 4 | 2 |
| 922 | \|A0A151MCA1\|A0A151MCA1_ALLMI Cytosolic 5'-nucleotidase 1A-like OS=Alligator mississippiensis GN=Y1Q_0000761 PE=4 SV=1 | 9909687,4 | 2 | 2 |
| 923 | \|A0A151PAG7\|A0A151PAG7_ALLMI Plasma kallikrein isoform B OS=Alligator mississippiensis GN=KLKB1 PE=4 SV=1 | 9814738,7 | 6 | 3 |
| 924 | \|A0A151P9B2\|A0A151P9B2_ALLMI Hepatocyte growth factor activator OS=Alligator mississippiensis GN=HGFAC PE=3 SV=1 | 9777172,2 | 5 | 3 |
| 925 | \|A0A151P856\|A0A151P856_ALLMI Ly6/PLAUR domain-containing protein 2 OS=Alligator mississippiensis GN=LYPD2 PE=4 SV=1 | 9759297,9 | 3 | 1 |
| 926 | \|A0A1U7S3L7\|A0A1U7S3L7_ALLSI leukocyte elastase inhibitor A OS=Alligator sinensis GN=LOC102384348 PE=3 SV=1 | 9718097,6 | 5 | 3 |
| 927 | \|A0A151ND71\|A0A151ND71_ALLMI Putative hydrolase RBBP9 OS=Alligator mississippiensis GN=RBBP9 PE=4 SV=1 | 9711587,0 | 7 | 4 |
| 928 | \|A0A151PC08\|A0A151PC08_ALLMI Signal transducer and activator of transcription OS=Alligator mississippiensis GN=STAT4 PE=3 SV=1 | 9668258,8 | 5 | 3 |
| 929 | \|A0A151PC58\|A0A151PC58_ALLMI Keratin, type I cytoskeletal 14 OS=Alligator mississippiensis GN=KRT14 PE=3 SV=1 | 9666398,5 | 3 | 3 |
| 930 | \|A0A151N9H9\|A0A151N9H9_ALLMI Fibronectin isoform A OS=Alligator mississippiensis GN=FN1 PE=4 SV=1 | 9657455,0 | 5 | 4 |
| 931 | \|A0A151N5K4\|A0A151N5K4_ALLMI Eukaryotic translation initiation factor 4H OS=Alligator mississippiensis GN=EIF4H PE=4 SV=1 | 9597815,3 | 5 | 3 |
| 932 | \|A0A151NYP2\|A0A151NYP2_ALLMI 40S ribosomal protein S21 OS=Alligator mississippiensis GN=RPS21 PE=3 SV=1 | 9552090,4 | 3 | 2 |
| 933 | \|A0A151LZS4\|A0A151LZS4_ALLMI Uncharacterized protein OS=Alligator mississippiensis GN=Y1Q_0011436 PE=4 SV=1 | 9550114,4 | 10 | 8 |
| 934 | \|A0A151MSN3\|A0A151MSN3_ALLMI E3 ubiquitin-protein ligase RFWD3 OS=Alligator mississippiensis GN=RFWD3 PE=4 SV=1 | 9535228,0 | 5 | 3 |
| 935 | \|A0A151MMV7\|A0A151MMV7_ALLMI Eukaryotic translation initiation factor 3 subunit I OS=Alligator mississippiensis GN=EIF3I PE=3 SV=1 | 9523390,1 | 3 | 3 |
| 936 | \|A0A1U7S025\|A0A1U7S025_ALLSI Peptidyl-prolyl cis-trans isomerase OS=Alligator sinensis GN=PPIH PE=3 SV=1 | 9520851,0 | 2 | 1 |
| 937 | \|A0A151NN44\|A0A151NN44_ALLMI Prefoldin subunit 3 OS=Alligator mississippiensis GN=VBP1 PE=3 SV=1 | 9469234,1 | 6 | 4 |
| 938 | \|A0A151P494\|A0A151P494_ALLMI Phospholysine phosphohistidine inorganic pyrophosphate phosphatase OS=Alligator mississippiensis GN=LHPP PE=4 SV=1 | 9432568,9 | 6 | 2 |
| 939 | \|A0A151PDX3\|A0A151PDX3_ALLMI AP-2 complex subunit mu isoform A OS=Alligator mississippiensis GN=AP2M1 PE=3 SV=1 | 9388788,1 | 4 | 3 |
| 940 | \|A0A151P815\|A0A151P815_ALLMI Lambda-crystallin-like protein OS=Alligator mississippiensis GN=CRYL1 PE=4 SV=1 | 9366830,0 | 8 | 3 |
| 941 | \|A0A151P620\|A0A151P620_ALLMI Proliferating cell nuclear antigen OS=Alligator mississippiensis GN=PCNA PE=3 SV=1 | 9317720,5 | 3 | 3 |
| 942 | \|A0A1U7RFC6\|A0A1U7RFC6_ALLSI GTPase-activating protein and VPS9 domain-containing protein 1 isoform X2 OS=Alligator sinensis GN=GAPVD1 PE=4 SV=1 | 9242801,3 | 7 | 6 |
| 943 | \|A0A151P266\|A0A151P266_ALLMI LanC-like protein 2 OS=Alligator mississippiensis GN=LANCL2 PE=4 SV=1 | 9237945,1 | 5 | 2 |
| 944 | \|A0A151PBM4\|A0A151PBM4_ALLMI Nascent polypeptide-associated complex subunit alpha OS=Alligator mississippiensis GN=NACA PE=4 SV=1 | 9231479,9 | 7 | 5 |
| 945 | \|A0A151N0S6\|A0A151N0S6_ALLMI Ribose-phosphate pyrophosphokinase 2 OS=Alligator mississippiensis GN=PRPS2 PE=3 SV=1 | 9214957,9 | 8 | 3 |
| 946 | \|A0A1U8DEB5\|A0A1U8DEB5_ALLSI retinal dehydrogenase 1 OS=Alligator sinensis GN=ALDH1A1 PE=3 SV=1 | 9158267,1 | 9 | 3 |
| 947 | \|A0A151NMA2\|A0A151NMA2_ALLMI Beta-mannosidase OS=Alligator mississippiensis GN=MANBA PE=3 SV=1 | 9107378,3 | 5 | 4 |
| 948 | \|A0A1U7SN93\|A0A1U7SN93_ALLSI Malic enzyme OS=Alligator sinensis GN=ME1 PE=3 SV=1 | 9078878,9 | 4 | 2 |
| 949 | \|A0A151P0J2\|A0A151P0J2_ALLMI Glutathione peroxidase OS=Alligator mississippiensis GN=GPX4 PE=3 SV=1 | 9072077,2 | 8 | 5 |
| 950 | \|A0A151NK46\|A0A151NK46_ALLMI 60S ribosomal protein L13a OS=Alligator mississippiensis GN=RPL13A PE=3 SV=1 | 9062982,2 | 8 | 5 |
| 951 | \|A0A151NJK0\|A0A151NJK0_ALLMI Nuclear transport factor 2 OS=Alligator mississippiensis GN=NUTF2 PE=4 SV=1 | 9052058,3 | 9 | 5 |
| 952 | \|A0A151P508\|A0A151P508_ALLMI Annexin OS=Alligator mississippiensis GN=ANXA4 PE=3 SV=1 | 9044983,5 | 11 | 5 |
| 953 | \|A0A151MSV6\|A0A151MSV6_ALLMI Uncharacterized protein OS=Alligator mississippiensis GN=Y1Q_0005166 PE=4 SV=1 | 8999466,0 | 7 | 6 |
| 954 | \|A0A151M020\|A0A151M020_ALLMI Cytoplasmic dynein 1 intermediate chain 2 isoform A OS=Alligator mississippiensis GN=DYNC1I2 PE=4 SV=1 | 8998042,8 | 7 | 5 |
| 955 | \|A0A151MBZ4\|A0A151MBZ4_ALLMI Uncharacterized protein OS=Alligator mississippiensis GN=Y1Q_0000665 PE=4 SV=1 | 8992814,7 | 6 | 4 |
| 956 | \|A0A151MJX9\|A0A151MJX9_ALLMI RuvB-like helicase OS=Alligator mississippiensis GN=RUVBL1 PE=3 SV=1 | 8963502,0 | 8 | 5 |
| 957 | \|A0A151LYR9\|A0A151LYR9_ALLMI Early endosome antigen 1 OS=Alligator mississippiensis GN=EEA1 PE=4 SV=1 | 8851510,7 | 7 | 7 |
| 958 | \|A0A1U7S9Z6\|A0A1U7S9Z6_ALLSI Coatomer subunit gamma OS=Alligator sinensis GN=COPG2 PE=3 SV=1 | 8821548,0 | 9 | 5 |
| 959 | \|A0A151PIR2\|A0A151PIR2_ALLMI Complement C2 OS=Alligator mississippiensis GN=C2 PE=3 SV=1 | 8812161,8 | 3 | 2 |
| 960 | \|A0A1U7SL53\|A0A1U7SL53_ALLSI tetratricopeptide repeat protein 38-like OS=Alligator sinensis GN=LOC102374927 PE=4 SV=1 | 8809115,1 | 5 | 3 |
| 961 | \|A0A151MK42\|A0A151MK42_ALLMI SEC13-like protein OS=Alligator mississippiensis GN=SEC13 PE=4 SV=1 | 8793655,9 | 8 | 6 |
| 962 | \|A0A151P099\|A0A151P099_ALLMI Prefoldin subunit 4 OS=Alligator mississippiensis GN=PFDN4 PE=3 SV=1 | 8742538,4 | 4 | 4 |
| 963 | \|A0A151N659\|A0A151N659_ALLMI Farnesyl pyrophosphate synthase isoform A OS=Alligator mississippiensis GN=FDPS PE=3 SV=1 | 8720442,6 | 2 | 2 |
| 964 | \|A0A151M5C3\|A0A151M5C3_ALLMI Prune-like protein isoform B OS=Alligator mississippiensis GN=PRUNE PE=4 SV=1 | 8699232,4 | 4 | 2 |
| 965 | \|A0A1U7RAY5\|A0A1U7RAY5_ALLSI rhophilin-2 OS=Alligator sinensis GN=RHPN2 PE=4 SV=1 | 8687680,1 | 3 | 3 |
| 966 | \|A0A151N3I2\|A0A151N3I2_ALLMI Cytosolic acyl coenzyme A thioester hydrolase isoform A OS=Alligator mississippiensis GN=ACOT7 PE=4 SV=1 | 8687124,7 | 5 | 2 |
| 967 | \|A0A1U8DAU9\|A0A1U8DAU9_ALLSI 6-phosphogluconate dehydrogenase, decarboxylating OS=Alligator sinensis GN=PGD PE=3 SV=1 | 8662717,0 | 3 | 2 |
| 968 | \|A0A151MC00\|A0A151MC00_ALLMI Uncharacterized protein OS=Alligator mississippiensis GN=Y1Q_0000658 PE=4 SV=1 | 8655516,2 | 4 | 4 |
| 969 | \|A0A151MJB9\|A0A151MJB9_ALLMI Sulfotransferase OS=Alligator mississippiensis GN=SULT1B1 PE=3 SV=1 | 8632656,0 | 2 | 2 |
| 970 | \|A0A151NVM2\|A0A151NVM2_ALLMI Tubulin-specific chaperone D OS=Alligator mississippiensis GN=TBCD PE=4 SV=1 | 8613507,9 | 7 | 5 |
| 971 | \|A0A151M2G2\|A0A151M2G2_ALLMI Acetyl-CoA acetyltransferase, cytosolic OS=Alligator mississippiensis GN=ACAT2 PE=3 SV=1 | 8583182,8 | 6 | 3 |
| 972 | \|A0A1U8DYZ4\|A0A1U8DYZ4_ALLSI uncharacterized protein LOC102382790 OS=Alligator sinensis GN=LOC102382790 PE=4 SV=1 | 8505976,4 | 2 | 2 |
| 973 | \|A0A151M976\|A0A151M976_ALLMI EGF-like repeat and discoidin I-like domain-containing protein 3 OS=Alligator mississippiensis GN=Y1Q_0001380 PE=4 SV=1 | 8466515,0 | 4 | 3 |
| 974 | \|A0A151NLK4\|A0A151NLK4_ALLMI Protein-L-isoaspartate(D-aspartate) O-methyltransferase OS=Alligator mississippiensis GN=PCMT1 PE=4 SV=1 | 8437163,1 | 5 | 3 |
| 975 | \|A0A1U8CYW7\|A0A1U8CYW7_ALLSI arfaptin-1 isoform X3 OS=Alligator sinensis GN=ARFIP1 PE=4 SV=1 | 8414080,2 | 11 | 6 |
| 976 | \|A0A151MAK7\|A0A151MAK7_ALLMI Extracellular matrix protein 1 OS=Alligator mississippiensis GN=ECM1 PE=4 SV=1 | 8322806,3 | 6 | 5 |
| 977 | \|A0A1U8DJD6\|A0A1U8DJD6_ALLSI syntaxin-binding protein 2 OS=Alligator sinensis GN=STXBP2 PE=3 SV=1 | 8292699,9 | 9 | 5 |
| 978 | \|A0A1U7RR68\|A0A1U7RR68_ALLSI Signal recognition particle subunit SRP72 OS=Alligator sinensis GN=SRP72 PE=3 SV=1 | 8069007,2 | 4 | 2 |
| 979 | \|A0A151PGU4\|A0A151PGU4_ALLMI Uncharacterized protein OS=Alligator mississippiensis GN=Y1Q_0024691 PE=4 SV=1 | 8056981,4 | 4 | 4 |
| 980 | \|A0A151N0P0\|A0A151N0P0_ALLMI Hsp90 co-chaperone Cdc37 OS=Alligator mississippiensis GN=CDC37 PE=4 SV=1 | 8038019,9 | 5 | 4 |
| 981 | \|A0A151NZP5\|A0A151NZP5_ALLMI Uncharacterized protein OS=Alligator mississippiensis GN=Y1Q_0002837 PE=4 SV=1 | 8004905,4 | 3 | 3 |
| 982 | \|A0A151MSR7\|A0A151MSR7_ALLMI Guanine nucleotide-binding protein G(O) subunit alpha OS=Alligator mississippiensis GN=GNAO1 PE=4 SV=1 | 8002584,2 | 4 | 2 |
| 983 | \|A0A1U7RC66\|A0A1U7RC66_ALLSI LOW QUALITY PROTEIN: 60S ribosomal protein L7 OS=Alligator sinensis GN=RPL7 PE=4 SV=1 | 7985837,3 | 5 | 5 |
| 984 | \|A0A151N7I1\|A0A151N7I1_ALLMI Hypoxanthine-guanine phosphoribosyltransferase OS=Alligator mississippiensis GN=HPRT1 PE=4 SV=1 | 7958238,5 | 5 | 2 |
| 985 | \|A0A151NL05\|A0A151NL05_ALLMI Tetraspanin OS=Alligator mississippiensis GN=CD63 PE=3 SV=1 | 7944159,2 | 5 | 2 |
| 986 | \|A0A151NGK1\|A0A151NGK1_ALLMI Carbamoyl-phosphate synthase [ammonia], mitochondrial OS=Alligator mississippiensis GN=CPS1 PE=3 SV=1 | 7921208,9 | 7 | 6 |
| 987 | \|A0A151NUX8\|A0A151NUX8_ALLMI Ubiquitin/ISG15-conjugating enzyme E2 L6 OS=Alligator mississippiensis GN=UBE2L6 PE=3 SV=1 | 7897623,2 | 4 | 3 |
| 988 | \|A0A151PC66\|A0A151PC66_ALLMI 2',3'-cyclic-nucleotide 3'-phosphodiesterase OS=Alligator mississippiensis GN=Y1Q_0018430 PE=4 SV=1 | 7895462,5 | 3 | 2 |
| 989 | \|A0A1U7RBB6\|A0A1U7RBB6_ALLSI carbonic anhydrase 2 OS=Alligator sinensis GN=CA2 PE=4 SV=1 | 7876414,8 | 2 | 2 |
| 990 | \|A0A151N2X7\|A0A151N2X7_ALLMI Uncharacterized protein OS=Alligator mississippiensis GN=Y1Q_0016496 PE=4 SV=1 | 7827746,8 | 9 | 7 |
| 991 | \|A0A1U8DVH3\|A0A1U8DVH3_ALLSI 40S ribosomal protein S17 OS=Alligator sinensis GN=RPS17 PE=3 SV=1 | 7827127,1 | 10 | 6 |
| 992 | \|A0A151MXY3\|A0A151MXY3_ALLMI NudC domain-containing protein 1 OS=Alligator mississippiensis GN=NUDCD1 PE=4 SV=1 | 7805526,0 | 3 | 2 |
| 993 | \|A0A1U7S2M2\|A0A1U7S2M2_ALLSI sorting nexin-12 OS=Alligator sinensis GN=SNX12 PE=4 SV=1 | 7779482,8 | 5 | 2 |
| 994 | \|A0A151M6J3\|A0A151M6J3_ALLMI Guanine nucleotide-binding protein subunit beta-4 OS=Alligator mississippiensis GN=GNB4 PE=4 SV=1 | 7767927,1 | 2 | 2 |
| 995 | \|A0A151M7A8\|A0A151M7A8_ALLMI GRIP1-associated protein 1 isoform A OS=Alligator mississippiensis GN=GRIPAP1 PE=4 SV=1 | 7681939,4 | 2 | 1 |
| 996 | \|A0A151MNB8\|A0A151MNB8_ALLMI Ras-related protein Rab-2A OS=Alligator mississippiensis GN=RAB2A PE=4 SV=1 | 7674193,6 | 8 | 4 |
| 997 | \|A0A1U7SBZ5\|A0A1U7SBZ5_ALLSI homeodomain-only protein OS=Alligator sinensis GN=HOPX PE=4 SV=1 | 7670096,3 | 8 | 4 |
| 998 | \|A0A151NEG3\|A0A151NEG3_ALLMI Phospholipase A-2-activating protein OS=Alligator mississippiensis GN=Y1Q_0001082 PE=4 SV=1 | 7655614,5 | 4 | 4 |
| 999 | \|A0A151NSY6\|A0A151NSY6_ALLMI CAP-Gly domain-containing linker protein 1 isoform A OS=Alligator mississippiensis GN=CLIP1 PE=4 SV=1 | 7642783,1 | 5 | 4 |
| 1000 | \|A0A151M2C0\|A0A151M2C0_ALLMI Plasminogen OS=Alligator mississippiensis GN=PLG PE=3 SV=1 | 7631329,7 | 3 | 2 |
| 1001 | \|A0A151NIT0\|A0A151NIT0_ALLMI Deoxyribose-phosphate aldolase OS=Alligator mississippiensis GN=DERA PE=4 SV=1 | 7625125,5 | 3 | 3 |
| 1002 | \|A0A151MJH3\|A0A151MJH3_ALLMI Sorting nexin-3 OS=Alligator mississippiensis GN=SNX3 PE=4 SV=1 | 7585800,6 | 8 | 3 |
| 1003 | \|A0A151MYZ1\|A0A151MYZ1_ALLMI Uncharacterized protein OS=Alligator mississippiensis GN=Y1Q_0005952 PE=4 SV=1 | 7520463,1 | 6 | 3 |
| 1004 | \|A0A151NL96\|A0A151NL96_ALLMI Formin-binding protein 1-like OS=Alligator mississippiensis GN=FNBP1L PE=4 SV=1 | 7455084,7 | 3 | 3 |
| 1005 | \|A0A151M731\|A0A151M731_ALLMI Heterogeneous nuclear ribonucleoprotein A0 OS=Alligator mississippiensis GN=HNRNPA0 PE=4 SV=1 | 7407951,4 | 4 | 2 |
| 1006 | \|A0A1U7RWB7\|A0A1U7RWB7_ALLSI ubiquitin-like modifier-activating enzyme 7 OS=Alligator sinensis GN=UBA7 PE=3 SV=1 | 7376107,6 | 4 | 4 |
| 1007 | \|A0A151P705\|A0A151P705_ALLMI Peptidylprolyl isomerase OS=Alligator mississippiensis GN=Y1Q_0016801 PE=4 SV=1 | 7313118,1 | 3 | 1 |
| 1008 | \|A0A151P233\|A0A151P233_ALLMI Sulfotransferase OS=Alligator mississippiensis GN=Y1Q_0012731 PE=3 SV=1 | 7288443,5 | 4 | 2 |
| 1009 | \|A0A151N4J5\|A0A151N4J5_ALLMI Translational activator GCN1 OS=Alligator mississippiensis GN=GCN1 PE=4 SV=1 | 7273399,7 | 6 | 6 |
| 1010 | \|A0A151NEW0\|A0A151NEW0_ALLMI mRNA cap guanine-N7 methyltransferase OS=Alligator mississippiensis GN=RNMT PE=3 SV=1 | 7223234,1 | 3 | 2 |
| 1011 | \|A0A151NHH6\|A0A151NHH6_ALLMI Adenylosuccinate lyase OS=Alligator mississippiensis GN=ADSL PE=3 SV=1 | 7190928,3 | 3 | 2 |
| 1012 | \|A0A1U7SCZ5\|A0A1U7SCZ5_ALLSI Glutathione peroxidase OS=Alligator sinensis GN=GPX1 PE=3 SV=1 | 7180251,6 | 6 | 3 |
| 1013 | \|A0A1U7SA63\|A0A1U7SA63_ALLSI rho GTPase-activating protein 1 OS=Alligator sinensis GN=ARHGAP1 PE=4 SV=1 | 7151784,2 | 5 | 4 |
| 1014 | \|A0A151M7I2\|A0A151M7I2_ALLMI tRNA-dihydrouridine(16/17) synthase [NAD(P)(+)]-like isoform A OS=Alligator mississippiensis GN=DUS1L PE=4 SV=1 | 7144098,6 | 7 | 5 |
| 1015 | \|A0A151P5B2\|A0A151P5B2_ALLMI Migration and invasion enhancer 1 OS=Alligator mississippiensis GN=MIEN1 PE=4 SV=1 | 7129401,6 | 3 | 2 |
| 1016 | \|A0A1U7S1V6\|A0A1U7S1V6_ALLSI ras-related protein Rab-8B OS=Alligator sinensis GN=RAB8B PE=4 SV=1 | 7122393,2 | 7 | 3 |
| 1017 | \|A0A151NBW3\|A0A151NBW3_ALLMI Proline synthase co-transcribed-like protein OS=Alligator mississippiensis GN=PROSC PE=3 SV=1 | 7072976,5 | 2 | 2 |
| 1018 | \|A0A151PBI5\|A0A151PBI5_ALLMI 60S ribosomal protein L27 OS=Alligator mississippiensis GN=RPL27 PE=3 SV=1 | 7024496,1 | 6 | 3 |
| 1019 | \|A0A1U7SF25\|A0A1U7SF25_ALLSI charged multivesicular body protein 4a isoform X2 OS=Alligator sinensis GN=CHMP4A PE=3 SV=1 | 7000952,4 | 5 | 4 |
| 1020 | \|A0A151NZ41\|A0A151NZ41_ALLMI AP-1 complex subunit mu-1 OS=Alligator mississippiensis GN=AP1M1 PE=3 SV=1 | 6878679,6 | 6 | 4 |
| 1021 | \|A0A1U7SJU0\|A0A1U7SJU0_ALLSI nicotinamide N-methyltransferase-like OS=Alligator sinensis GN=LOC102377471 PE=4 SV=1 | 6870807,1 | 5 | 3 |
| 1022 | \|A0A151NR82\|A0A151NR82_ALLMI Uncharacterized protein OS=Alligator mississippiensis GN=Y1Q_0008569 PE=3 SV=1 | 6862101,9 | 7 | 5 |
| 1023 | \|A0A151N3M5\|A0A151N3M5_ALLMI 60S ribosomal protein L22 OS=Alligator mississippiensis GN=RPL22 PE=4 SV=1 | 6788095,3 | 3 | 2 |
| 1024 | \|A0A151NMB3\|A0A151NMB3_ALLMI Dynein light chain roadblock OS=Alligator mississippiensis GN=DYNLRB2 PE=3 SV=1 | 6772167,8 | 1 | 1 |
| 1025 | \|A0A151PAY0\|A0A151PAY0_ALLMI Ubiquitin-conjugating enzyme E2 K OS=Alligator mississippiensis GN=UBE2K PE=3 SV=1 | 6769880,4 | 4 | 3 |
| 1026 | \|A0A151MIT4\|A0A151MIT4_ALLMI Hydroxyacyl-coenzyme A dehydrogenase, mitochondrial OS=Alligator mississippiensis GN=HADH PE=4 SV=1 | 6741375,5 | 9 | 6 |
| 1027 | \|A0A151NQU2\|A0A151NQU2_ALLMI Matrin-3 OS=Alligator mississippiensis GN=MATR3 PE=4 SV=1 | 6671286,7 | 8 | 4 |
| 1028 | \|A0A151NVH0\|A0A151NVH0_ALLMI Catenin delta-1 isoform A OS=Alligator mississippiensis GN=CTNND1 PE=4 SV=1 | 6670031,3 | 4 | 3 |
| 1029 | \|A0A151NGE3\|A0A151NGE3_ALLMI Uncharacterized protein OS=Alligator mississippiensis GN=Y1Q_0020549 PE=4 SV=1 | 6573874,4 | 4 | 2 |
| 1030 | \|A0A1U8DCX7\|A0A1U8DCX7_ALLSI LOW QUALITY PROTEIN: beta-enolase OS=Alligator sinensis GN=ENO3 PE=3 SV=1 | 6538365,8 | 2 | 2 |
| 1031 | \|A0A151P1Q4\|A0A151P1Q4_ALLMI Uncharacterized protein OS=Alligator mississippiensis GN=Y1Q_0012903 PE=4 SV=1 | 6500468,7 | 7 | 4 |
| 1032 | \|A0A151M3T2\|A0A151M3T2_ALLMI DNA-(apurinic or apyrimidinic site) lyase OS=Alligator mississippiensis GN=APEX1 PE=3 SV=1 | 6438730,7 | 4 | 2 |
| 1033 | \|A0A151NQR3\|A0A151NQR3_ALLMI Putative ubiquitin carboxyl-terminal hydrolase FAF-X OS=Alligator mississippiensis GN=USP9X PE=3 SV=1 | 6328907,0 | 4 | 3 |
| 1034 | \|A0A151N8Y0\|A0A151N8Y0_ALLMI Adseverin OS=Alligator mississippiensis GN=SCINL PE=4 SV=1 | 6320240,5 | 6 | 3 |
| 1035 | \|A0A151PCW6\|A0A151PCW6_ALLMI Abl interactor 2 OS=Alligator mississippiensis GN=ABI2 PE=4 SV=1 | 6311622,6 | 4 | 2 |
| 1036 | \|A0A151P5R7\|A0A151P5R7_ALLMI Pyridoxine-5'-phosphate oxidase OS=Alligator mississippiensis GN=PNPO PE=3 SV=1 | 6243186,6 | 3 | 3 |
| 1037 | \|A0A151MGB8\|A0A151MGB8_ALLMI Alpha-amylase OS=Alligator mississippiensis GN=Y1Q_0010113 PE=3 SV=1 | 6239207,8 | 4 | 3 |
| 1038 | \|A0A151NQV2\|A0A151NQV2_ALLMI Uncharacterized protein OS=Alligator mississippiensis GN=Y1Q_0004857 PE=3 SV=1 | 6139485,6 | 3 | 3 |
| 1039 | \|A0A151MLC9\|A0A151MLC9_ALLMI Adapter molecule crk OS=Alligator mississippiensis GN=CRK PE=4 SV=1 | 6129208,1 | 5 | 5 |
| 1040 | \|A0A151MHH8\|A0A151MHH8_ALLMI Heterogeneous nuclear ribonucleoprotein U OS=Alligator mississippiensis GN=HNRNPU PE=4 SV=1 | 6080468,0 | 5 | 5 |
| 1041 | \|A0A151NA49\|A0A151NA49_ALLMI Heat shock protein, mitochondrial OS=Alligator mississippiensis GN=HSPD1 PE=3 SV=1 | 6059902,9 | 5 | 2 |
| 1042 | \|A0A151PI83\|A0A151PI83_ALLMI Ras GTPase-activating protein 1 OS=Alligator mississippiensis GN=RASA1 PE=4 SV=1 | 6042261,0 | 3 | 2 |
| 1043 | \|A0A151NI06\|A0A151NI06_ALLMI Uncharacterized protein OS=Alligator mississippiensis GN=Y1Q_0024184 PE=3 SV=1 | 6022566,4 | 4 | 4 |
| 1044 | \|A0A151MG19\|A0A151MG19_ALLMI Ras-related protein Rab-14 OS=Alligator mississippiensis GN=RAB14 PE=4 SV=1 | 5988133,9 | 5 | 4 |
| 1045 | \|A0A1U8DH77\|A0A1U8DH77_ALLSI NAD(P)H-hydrate epimerase OS=Alligator sinensis GN=APOA1BP PE=3 SV=1 | 5972643,6 | 8 | 5 |
| 1046 | \|A0A1U8CUF4\|A0A1U8CUF4_ALLSI ubiquitin-conjugating enzyme E2 variant 1 OS=Alligator sinensis GN=LOC106721866 PE=3 SV=1 | 5919100,5 | 6 | 3 |
| 1047 | \|A0A151NC49\|A0A151NC49_ALLMI Serotriflin OS=Alligator mississippiensis GN=CRISP2 PE=3 SV=1 | 5912273,0 | 2 | 1 |
| 1048 | \|A0A1U8DQI5\|A0A1U8DQI5_ALLSI kynurenine--oxoglutarate transaminase 3 isoform X1 OS=Alligator sinensis GN=CCBL2 PE=4 SV=1 | 5834230,1 | 3 | 2 |
| 1049 | \|A0A1U8DBQ2\|A0A1U8DBQ2_ALLSI BRCA1-A complex subunit BRE OS=Alligator sinensis GN=LOC102371325 PE=4 SV=1 | 5775409,6 | 3 | 2 |
| 1050 | \|A0A1U7RBY2\|A0A1U7RBY2_ALLSI fetuin-B OS=Alligator sinensis GN=LOC102382361 PE=4 SV=1 | 5769333,8 | 3 | 3 |
| 1051 | \|A0A1U8DJK2\|A0A1U8DJK2_ALLSI uncharacterized protein LOC106722952 OS=Alligator sinensis GN=LOC106722952 PE=4 SV=1 | 5764797,3 | 4 | 1 |
| 1052 | \|A0A151MKJ5\|A0A151MKJ5_ALLMI Protein FAM3D OS=Alligator mississippiensis GN=FAM3D PE=4 SV=1 | 5755058,8 | 5 | 1 |
| 1053 | \|A0A1U8DFL4\|A0A1U8DFL4_ALLSI protein kinase C and casein kinase substrate in neurons protein 2 isoform X1 OS=Alligator sinensis GN=PACSIN2 PE=4 SV=1 | 5740002,5 | 5 | 4 |
| 1054 | \|A0A151N8K8\|A0A151N8K8_ALLMI Glutathione S-transferase Mu 3 OS=Alligator mississippiensis GN=GSTM3 PE=4 SV=1 | 5724116,2 | 7 | 2 |
| 1055 | \|A0A151NMI4\|A0A151NMI4_ALLMI Rap1 GTPase-GDP dissociation stimulator 1 isoform B OS=Alligator mississippiensis GN=RAP1GDS1 PE=4 SV=1 | 5702101,2 | 3 | 2 |
| 1056 | \|A0A151MQA4\|A0A151MQA4_ALLMI Desmin OS=Alligator mississippiensis GN=DESMB PE=3 SV=1 | 5681028,1 | 5 | 3 |
| 1057 | \|A0A151PHZ8\|A0A151PHZ8_ALLMI COP9 signalosome complex subunit 6 OS=Alligator mississippiensis GN=COPS6 PE=4 SV=1 | 5662726,0 | 4 | 3 |
| 1058 | \|A0A151N2B7\|A0A151N2B7_ALLMI Serine/threonine-protein phosphatase OS=Alligator mississippiensis GN=PPP5C PE=3 SV=1 | 5641496,1 | 2 | 1 |
| 1059 | \|A0A1U8DEY3\|A0A1U8DEY3_ALLSI arginine--tRNA ligase, cytoplasmic isoform X2 OS=Alligator sinensis GN=RARS PE=3 SV=1 | 5592242,6 | 6 | 4 |
| 1060 | \|A0A151MVQ8\|A0A151MVQ8_ALLMI Mitogen-activated protein kinase OS=Alligator mississippiensis GN=MAPK15 PE=4 SV=1 | 5553534,2 | 4 | 1 |
| 1061 | \|A0A1U7SHT7\|A0A1U7SHT7_ALLSI exocyst complex component 4 OS=Alligator sinensis GN=EXOC4 PE=4 SV=1 | 5533805,7 | 4 | 3 |
| 1062 | \|A0A151N6L5\|A0A151N6L5_ALLMI RNA-binding Musashi-like protein 2 isoform B OS=Alligator mississippiensis GN=MSI2 PE=4 SV=1 | 5473508,2 | 2 | 1 |
| 1063 | \|A0A1U7RXH6\|A0A1U7RXH6_ALLSI lamin-B1 OS=Alligator sinensis GN=LMNB1 PE=3 SV=1 | 5469071,7 | 4 | 4 |
| 1064 | \|A0A151N2F7\|A0A151N2F7_ALLMI Mesothelin OS=Alligator mississippiensis GN=MSLN PE=4 SV=1 | 5395385,3 | 7 | 5 |
| 1065 | \|A0A151MKP1\|A0A151MKP1_ALLMI Ras GTPase-activating-like protein IQGAP2 OS=Alligator mississippiensis GN=IQGAP2 PE=4 SV=1 | 5395318,9 | 3 | 3 |
| 1066 | \|A0A151NHV6\|A0A151NHV6_ALLMI Sulfurtransferase OS=Alligator mississippiensis GN=MPST PE=4 SV=1 | 5393704,7 | 5 | 3 |
| 1067 | \|A0A1U8DBW7\|A0A1U8DBW7_ALLSI creatine kinase U-type, mitochondrial OS=Alligator sinensis GN=LOC102386529 PE=3 SV=1 | 5390678,0 | 7 | 5 |
| 1068 | \|A0A1U8DHN9\|A0A1U8DHN9_ALLSI septin-2 OS=Alligator sinensis GN=SEPT2 PE=3 SV=1 | 5389918,1 | 3 | 3 |
| 1069 | \|A0A1U7SDX6\|A0A1U7SDX6_ALLSI O-acetyl-ADP-ribose deacetylase MACROD2 OS=Alligator sinensis GN=MACROD2 PE=4 SV=1 | 5379946,9 | 3 | 2 |
| 1070 | \|A0A1U7RUB6\|A0A1U7RUB6_ALLSI activated RNA polymerase II transcriptional coactivator p15 OS=Alligator sinensis GN=SUB1 PE=4 SV=1 | 5369160,7 | 7 | 3 |
| 1071 | \|A0A151PFH8\|A0A151PFH8_ALLMI Nucleosome assembly protein 1-like 1 isoform A OS=Alligator mississippiensis GN=NAP1L1 PE=3 SV=1 | 5364299,1 | 6 | 3 |
| 1072 | \|A0A1U7RYZ6\|A0A1U7RYZ6_ALLSI lysozyme g-like OS=Alligator sinensis GN=LOC102379439 PE=4 SV=1 | 5356413,0 | 4 | 3 |
| 1073 | \|A0A151P2M7\|A0A151P2M7_ALLMI Small nuclear ribonucleoprotein Sm D1 OS=Alligator mississippiensis GN=SNRPD1 PE=4 SV=1 | 5355644,5 | 1 | 1 |
| 1074 | \|A0A151NAC6\|A0A151NAC6_ALLMI Leucine-rich repeat flightless-interacting protein 1 OS=Alligator mississippiensis GN=LRRFIP1 PE=4 SV=1 | 5347965,9 | 3 | 2 |
| 1075 | \|A0A151N604\|A0A151N604_ALLMI Clusterin OS=Alligator mississippiensis GN=CLU PE=3 SV=1 | 5269836,5 | 3 | 2 |
| 1076 | \|A0A1U7RQG2\|A0A1U7RQG2_ALLSI protein flightless-1 homolog OS=Alligator sinensis GN=FLII PE=4 SV=1 | 5227760,7 | 7 | 7 |
| 1077 | \|A0A1U8D4S3\|A0A1U8D4S3_ALLSI complement factor I isoform X2 OS=Alligator sinensis GN=CFI PE=3 SV=1 | 5224046,5 | 3 | 2 |
| 1078 | \|A0A1U7RKR2\|A0A1U7RKR2_ALLSI dynein light chain 2, cytoplasmic OS=Alligator sinensis GN=DYNLL2 PE=4 SV=1 | 5182634,7 | 5 | 4 |
| 1079 | \|A0A151NPP5\|A0A151NPP5_ALLMI Optineurin OS=Alligator mississippiensis GN=OPTN PE=4 SV=1 | 5180826,0 | 4 | 3 |
| 1080 | \|A0A1U7RG96\|A0A1U7RG96_ALLSI small glutamine-rich tetratricopeptide repeat-containing protein alpha isoform X2 OS=Alligator sinensis GN=SGTA PE=4 SV=1 | 5171829,8 | 2 | 2 |
| 1081 | \|A0A1U7S996\|A0A1U7S996_ALLSI 60 kDa SS-A/Ro ribonucleoprotein OS=Alligator sinensis GN=TROVE2 PE=4 SV=1 | 5165120,0 | 3 | 3 |
| 1082 | \|A0A151MC56\|A0A151MC56_ALLMI Uncharacterized protein OS=Alligator mississippiensis GN=Y1Q_0000679 PE=3 SV=1 | 5135693,7 | 4 | 3 |
| 1083 | \|A0A1U8DFN7\|A0A1U8DFN7_ALLSI ubiquitin-like modifier-activating enzyme ATG7 isoform X1 OS=Alligator sinensis GN=ATG7 PE=4 SV=1 | 5133089,8 | 7 | 5 |
| 1084 | \|A0A151PCJ1\|A0A151PCJ1_ALLMI Ras-related protein Rab-18 OS=Alligator mississippiensis GN=RAB18 PE=4 SV=1 | 5107988,7 | 5 | 3 |
| 1085 | \|A0A1U7SLT8\|A0A1U7SLT8_ALLSI junctional adhesion molecule A-like OS=Alligator sinensis GN=LOC102383731 PE=4 SV=1 | 5058992,9 | 2 | 2 |
| 1086 | \|A0A151M6X5\|A0A151M6X5_ALLMI PDZ and LIM domain protein 4 OS=Alligator mississippiensis GN=PDLIM4 PE=4 SV=1 | 5014867,9 | 3 | 1 |
| 1087 | \|A0A1U7SFI8\|A0A1U7SFI8_ALLSI LOW QUALITY PROTEIN: elongation factor 1-beta OS=Alligator sinensis GN=EEF1B2 PE=3 SV=1 | 4997389,4 | 7 | 4 |
| 1088 | \|A0A1U7RS44\|A0A1U7RS44_ALLSI phospholipase DDHD2 isoform X1 OS=Alligator sinensis GN=DDHD2 PE=4 SV=1 | 4973023,3 | 7 | 2 |
| 1089 | \|A0A151NRH1\|A0A151NRH1_ALLMI Thimet oligopeptidase OS=Alligator mississippiensis GN=THOP1 PE=3 SV=1 | 4962946,7 | 4 | 3 |
| 1090 | \|A0A151N325\|A0A151N325_ALLMI Uncharacterized protein OS=Alligator mississippiensis GN=Y1Q_0016527 PE=4 SV=1 | 4888700,9 | 3 | 3 |
| 1091 | \|A0A151M4B1\|A0A151M4B1_ALLMI Nardilysin OS=Alligator mississippiensis GN=NRD1 PE=3 SV=1 | 4880771,7 | 3 | 1 |
| 1092 | \|A0A151NFL7\|A0A151NFL7_ALLMI Inosine-5'-monophosphate dehydrogenase OS=Alligator mississippiensis GN=IMPDH PE=3 SV=1 | 4858226,1 | 2 | 2 |
| 1093 | \|A0A151N085\|A0A151N085_ALLMI Spermine synthase OS=Alligator mississippiensis GN=SMS PE=4 SV=1 | 4775215,0 | 2 | 1 |
| 1094 | \|A0A1U8CZF0\|A0A1U8CZF0_ALLSI catechol O-methyltransferase isoform X3 OS=Alligator sinensis GN=COMT PE=4 SV=1 | 4747956,8 | 8 | 3 |
| 1095 | \|A0A151PDZ6\|A0A151PDZ6_ALLMI Uncharacterized protein OS=Alligator mississippiensis GN=Y1Q_0019947 PE=4 SV=1 | 4738888,6 | 6 | 4 |
| 1096 | \|A0A1U7S279\|A0A1U7S279_ALLSI calpain-2 catalytic subunit OS=Alligator sinensis GN=CAPN2 PE=3 SV=1 | 4737587,3 | 7 | 5 |
| 1097 | \|A0A151PIB4\|A0A151PIB4_ALLMI Glutaredoxin-1 OS=Alligator mississippiensis GN=GLRX PE=4 SV=1 | 4696633,4 | 4 | 2 |
| 1098 | \|A0A151MIB1\|A0A151MIB1_ALLMI Sulfhydryl oxidase OS=Alligator mississippiensis GN=Y1Q_0004308 PE=4 SV=1 | 4664964,0 | 2 | 2 |
| 1099 | \|A0A1U7SJG7\|A0A1U7SJG7_ALLSI LOW QUALITY PROTEIN: heterogeneous nuclear ribonucleoprotein L OS=Alligator sinensis GN=HNRNPL PE=4 SV=1 | 4650314,0 | 3 | 3 |
| 1100 | \|A0A1U8DJB1\|A0A1U8DJB1_ALLSI COP9 signalosome complex subunit 5 OS=Alligator sinensis GN=COPS5 PE=4 SV=1 | 4594036,9 | 2 | 2 |
| 1101 | \|A0A151MZX1\|A0A151MZX1_ALLMI Signal-transducing adaptor protein 2 isoform A OS=Alligator mississippiensis GN=STAP2 PE=4 SV=1 | 4556852,6 | 4 | 2 |
| 1102 | \|A0A151NGC3\|A0A151NGC3_ALLMI Citrate synthase OS=Alligator mississippiensis GN=CS PE=3 SV=1 | 4535659,3 | 2 | 2 |
| 1103 | \|A0A151PCX6\|A0A151PCX6_ALLMI Glycylpeptide N-tetradecanoyltransferase OS=Alligator mississippiensis GN=NMT1 PE=3 SV=1 | 4450236,0 | 2 | 2 |
| 1104 | \|A0A1U8D293\|A0A1U8D293_ALLSI Methylthioribose-1-phosphate isomerase OS=Alligator sinensis GN=MRI1 PE=3 SV=1 | 4431955,1 | 3 | 3 |
| 1105 | \|A0A1U8DFP2\|A0A1U8DFP2_ALLSI complement component C6-like OS=Alligator sinensis GN=LOC102376310 PE=4 SV=1 | 4431172,6 | 4 | 3 |
| 1106 | \|A0A1U8DA74\|A0A1U8DA74_ALLSI oxidation resistance protein 1 isoform X5 OS=Alligator sinensis GN=OXR1 PE=4 SV=1 | 4390103,6 | 4 | 3 |
| 1107 | \|A0A151NKP0\|A0A151NKP0_ALLMI 14-3-3 protein sigma OS=Alligator mississippiensis GN=SFN PE=3 SV=1 | 4353169,0 | 3 | 1 |
| 1108 | \|A0A151PA01\|A0A151PA01_ALLMI Uncharacterized protein OS=Alligator mississippiensis GN=Y1Q_0021464 PE=4 SV=1 | 4342374,1 | 8 | 7 |
| 1109 | \|A0A151PJ52\|A0A151PJ52_ALLMI Ras-related protein Rab-6A isoform A OS=Alligator mississippiensis GN=RAB6A-1 PE=4 SV=1 | 4342014,3 | 3 | 2 |
| 1110 | \|A0A151NLR5\|A0A151NLR5_ALLMI LIM domain only protein 7 isoform C OS=Alligator mississippiensis GN=LMO7 PE=4 SV=1 | 4333286,9 | 7 | 4 |
| 1111 | \|A0A1U7S8S7\|A0A1U7S8S7_ALLSI fumarylacetoacetate hydrolase domain-containing protein 2A OS=Alligator sinensis GN=FAHD2A PE=4 SV=1 | 4285916,2 | 4 | 2 |
| 1112 | \|A0A151NDA6\|A0A151NDA6_ALLMI Protein transport protein Sec23B isoform B OS=Alligator mississippiensis GN=SEC23B PE=4 SV=1 | 4284140,7 | 3 | 3 |
| 1113 | \|A0A151MCD8\|A0A151MCD8_ALLMI Mucin-6 OS=Alligator mississippiensis GN=MUC6 PE=4 SV=1 | 4268833,5 | 5 | 4 |
| 1114 | \|A0A151MM48\|A0A151MM48_ALLMI Lipoma-preferred partner OS=Alligator mississippiensis GN=LPP PE=4 SV=1 | 4220613,2 | 3 | 2 |
| 1115 | \|A0A1U7RYA8\|A0A1U7RYA8_ALLSI ankyrin-3 isoform X1 OS=Alligator sinensis GN=ANK3 PE=4 SV=1 | 4190352,9 | 4 | 3 |
| 1116 | \|A0A151M9N8\|A0A151M9N8_ALLMI cAMP-regulated phosphoprotein 19 OS=Alligator mississippiensis GN=ARPP19 PE=3 SV=1 | 4054658,9 | 2 | 1 |
| 1117 | \|A0A151MI49\|A0A151MI49_ALLMI Ubiquitin carboxyl-terminal hydrolase OS=Alligator mississippiensis GN=UCHL5 PE=3 SV=1 | 4051622,6 | 3 | 3 |
| 1118 | \|A0A1U7SPX7\|A0A1U7SPX7_ALLSI Proteasome endopeptidase complex OS=Alligator sinensis GN=PSMA5 PE=3 SV=1 | 4023809,1 | 5 | 1 |
| 1119 | \|A0A151MLK3\|A0A151MLK3_ALLMI Translation initiation factor eIF-2B subunit beta OS=Alligator mississippiensis GN=EIF2B2 PE=3 SV=1 | 4002366,3 | 3 | 1 |
| 1120 | \|A0A151M4H1\|A0A151M4H1_ALLMI Complement component C8 alpha chain OS=Alligator mississippiensis GN=C8A PE=4 SV=1 | 3998575,3 | 4 | 2 |
| 1121 | \|A0A151PJ43\|A0A151PJ43_ALLMI Protein phosphatase methylesterase 1 OS=Alligator mississippiensis GN=PPME1 PE=3 SV=1 | 3995633,2 | 5 | 3 |
| 1122 | \|A0A151ND14\|A0A151ND14_ALLMI Thioredoxin domain-containing protein 5 OS=Alligator mississippiensis GN=TXNDC5 PE=3 SV=1 | 3995239,4 | 3 | 2 |
| 1123 | \|A0A151NS93\|A0A151NS93_ALLMI Serine/threonine-protein phosphatase OS=Alligator mississippiensis GN=Y1Q_0018743 PE=3 SV=1 | 3984138,0 | 2 | 2 |
| 1124 | \|A0A151P3N6\|A0A151P3N6_ALLMI Serine/threonine-protein phosphatase 2A regulatory subunit gamma OS=Alligator mississippiensis GN=PPP2R5C PE=4 SV=1 | 3977177,4 | 4 | 4 |
| 1125 | \|A0A151NZJ2\|A0A151NZJ2_ALLMI Mitochondrial import receptor subunit TOM34 OS=Alligator mississippiensis GN=TOMM34 PE=4 SV=1 | 3945051,8 | 2 | 2 |
| 1126 | \|A0A1U8DD58\|A0A1U8DD58_ALLSI UV excision repair protein RAD23 homolog B OS=Alligator sinensis GN=RAD23B PE=4 SV=1 | 3940637,8 | 2 | 1 |
| 1127 | \|A0A1U8DXM2\|A0A1U8DXM2_ALLSI 26S proteasome non-ATPase regulatory subunit 5 OS=Alligator sinensis GN=PSMD5 PE=4 SV=1 | 3865815,1 | 2 | 2 |
| 1128 | \|A0A1U7SA86\|A0A1U7SA86_ALLSI immunoglobulin J chain OS=Alligator sinensis GN=JCHAIN PE=4 SV=1 | 3844835,5 | 4 | 3 |
| 1129 | \|A0A1U7SDK7\|A0A1U7SDK7_ALLSI LOW QUALITY PROTEIN: nuclease-sensitive element-binding protein 1 OS=Alligator sinensis GN=YBX1 PE=4 SV=1 | 3836919,4 | 2 | 1 |
| 1130 | \|A0A151NL87\|A0A151NL87_ALLMI Copper transport protein ATOX1 OS=Alligator mississippiensis GN=ATOX1 PE=4 SV=1 | 3813589,8 | 3 | 2 |
| 1131 | \|Q90WT4\|Q90WT4_CRONI Putative thrombin (Fragment) OS=Crocodylus niloticus GN=thrombin PE=2 SV=1 | 3812535,1 | 2 | 2 |
| 1132 | \|A0A151N1J1\|A0A151N1J1_ALLMI Mismatch repair endonuclease PMS2 OS=Alligator mississippiensis GN=PMS2 PE=4 SV=1 | 3803756,0 | 4 | 2 |
| 1133 | \|A0A151MUT9\|A0A151MUT9_ALLMI Heterogeneous nuclear ribonucleoprotein A1 isoform B OS=Alligator mississippiensis GN=HNRNPA1-1 PE=4 SV=1 | 3782675,7 | 2 | 1 |
| 1134 | \|A0A151MMN0\|A0A151MMN0_ALLMI Tissue alpha-L-fucosidase OS=Alligator mississippiensis GN=FUCA1 PE=4 SV=1 | 3763939,9 | 3 | 3 |
| 1135 | \|A0A1U8D4J3\|A0A1U8D4J3_ALLSI N-acetylneuraminate lyase isoform X2 OS=Alligator sinensis GN=NPL PE=3 SV=1 | 3758113,4 | 2 | 2 |
| 1136 | \|A0A151LZ13\|A0A151LZ13_ALLMI Histone H1.01-like OS=Alligator mississippiensis GN=Y1Q_0020094 PE=3 SV=1 | 3747806,9 | 3 | 2 |
| 1137 | \|A0A151MR00\|A0A151MR00_ALLMI Cytoplasmic dynein 1 light intermediate chain 1 OS=Alligator mississippiensis GN=DYNC1LI1 PE=4 SV=1 | 3724082,5 | 3 | 2 |
| 1138 | \|A0A151N0Z2\|A0A151N0Z2_ALLMI Interferon regulatory factor 3 isoform B OS=Alligator mississippiensis GN=IRF3 PE=3 SV=1 | 3700092,5 | 4 | 1 |
| 1139 | \|A0A1U7RRY1\|A0A1U7RRY1_ALLSI ribonuclease inhibitor OS=Alligator sinensis GN=LOC102380390 PE=4 SV=1 | 3689720,2 | 2 | 1 |
| 1140 | \|A0A151N5B1\|A0A151N5B1_ALLMI RNA-binding protein EWS isoform B OS=Alligator mississippiensis GN=EWSR1 PE=4 SV=1 | 3683021,0 | 2 | 1 |
| 1141 | \|A0A1U8DK88\|A0A1U8DK88_ALLSI Protein arginine N-methyltransferase 5 OS=Alligator sinensis GN=PRMT5 PE=3 SV=1 | 3673421,5 | 3 | 2 |
| 1142 | \|A0A151MMQ9\|A0A151MMQ9_ALLMI Uncharacterized protein OS=Alligator mississippiensis GN=Y1Q_0023633 PE=4 SV=1 | 3668673,4 | 3 | 3 |
| 1143 | \|A0A151N9I9\|A0A151N9I9_ALLMI Bifunctional purine biosynthesis protein PURH isoform B OS=Alligator mississippiensis GN=ATIC PE=3 SV=1 | 3634266,0 | 3 | 2 |
| 1144 | \|A0A151MA54\|A0A151MA54_ALLMI Uncharacterized protein OS=Alligator mississippiensis GN=Y1Q_0001620 PE=4 SV=1 | 3601550,6 | 3 | 3 |
| 1145 | \|A0A151NEI2\|A0A151NEI2_ALLMI Ubiquitin carboxyl-terminal hydrolase 14 OS=Alligator mississippiensis GN=USP14 PE=3 SV=1 | 3598218,3 | 4 | 3 |
| 1146 | \|A0A151M7F0\|A0A151M7F0_ALLMI Alpha-1-inhibitor 3-like OS=Alligator mississippiensis GN=Y1Q_0016305 PE=4 SV=1 | 3586590,0 | 3 | 2 |
| 1147 | \|A0A151NII2\|A0A151NII2_ALLMI Uncharacterized protein OS=Alligator mississippiensis GN=Y1Q_0024087 PE=3 SV=1 | 3539340,8 | 3 | 3 |
| 1148 | \|A0A151MZY7\|A0A151MZY7_ALLMI Uncharacterized protein OS=Alligator mississippiensis GN=Y1Q_0000362 PE=4 SV=1 | 3513812,6 | 3 | 3 |
| 1149 | \|A0A1U7RU79\|A0A1U7RU79_ALLSI CD166 antigen OS=Alligator sinensis GN=ALCAM PE=4 SV=1 | 3493379,5 | 6 | 6 |
| 1150 | \|A0A1U7RH51\|A0A1U7RH51_ALLSI macrophage migration inhibitory factor OS=Alligator sinensis GN=LOC102383931 PE=4 SV=1 | 3483577,8 | 3 | 2 |
| 1151 | \|A0A151NWQ3\|A0A151NWQ3_ALLMI ATP-dependent (S)-NAD(P)H-hydrate dehydratase OS=Alligator mississippiensis GN=CARKD PE=3 SV=1 | 3481347,6 | 3 | 2 |
| 1152 | \|A0A1U7SNL7\|A0A1U7SNL7_ALLSI protein NDRG2 isoform X2 OS=Alligator sinensis GN=NDRG2 PE=4 SV=1 | 3474523,9 | 5 | 3 |
| 1153 | \|A0A151NYH8\|A0A151NYH8_ALLMI Ras-related protein Rab-3A OS=Alligator mississippiensis GN=RAB3A PE=4 SV=1 | 3439808,3 | 3 | 1 |
| 1154 | \|A0A151NKW9\|A0A151NKW9_ALLMI Sciellin isoform A OS=Alligator mississippiensis GN=SCEL-1 PE=4 SV=1 | 3422235,8 | 3 | 1 |
| 1155 | \|A0A151M1D5\|A0A151M1D5_ALLMI 40S ribosomal protein S19 OS=Alligator mississippiensis GN=RPS19 PE=4 SV=1 | 3416271,0 | 4 | 4 |
| 1156 | \|A0A1U8DW76\|A0A1U8DW76_ALLSI importin-5 OS=Alligator sinensis GN=IPO5 PE=4 SV=1 | 3397771,8 | 3 | 3 |
| 1157 | \|A0A1U7S8K6\|A0A1U7S8K6_ALLSI glyoxalase domain-containing protein 4 OS=Alligator sinensis GN=GLOD4 PE=4 SV=1 | 3353626,7 | 1 | 1 |
| 1158 | \|A0A151LZ07\|A0A151LZ07_ALLMI Histone H2A OS=Alligator mississippiensis GN=HIST1H2A3 PE=3 SV=1 | 3318926,7 | 5 | 3 |
| 1159 | \|A0A151NEW3\|A0A151NEW3_ALLMI Inositol-1-monophosphatase OS=Alligator mississippiensis GN=IMPA2 PE=3 SV=1 | 3237196,1 | 8 | 5 |
| 1160 | \|A0A1U7SP46\|A0A1U7SP46_ALLSI CD2-associated protein OS=Alligator sinensis GN=CD2AP PE=4 SV=1 | 3227285,0 | 4 | 3 |
| 1161 | \|A0A1U7S5T2\|A0A1U7S5T2_ALLSI ras-related protein Rab-5B OS=Alligator sinensis GN=RAB5B PE=4 SV=1 | 3219111,3 | 2 | 1 |
| 1162 | \|A0A151NIB4\|A0A151NIB4_ALLMI Cytidine and dCMP deaminase domain-containing protein 1-like OS=Alligator mississippiensis GN=Y1Q_0024264 PE=4 SV=1 | 3214244,4 | 2 | 2 |
| 1163 | \|A0A151NS47\|A0A151NS47_ALLMI Translation initiation factor eIF-2B subunit alpha OS=Alligator mississippiensis GN=EIF2B1 PE=3 SV=1 | 3191841,2 | 4 | 3 |
| 1164 | \|A0A151MIH1\|A0A151MIH1_ALLMI Rab GTPase-activating protein 1 isoform B OS=Alligator mississippiensis GN=RABGAP1 PE=4 SV=1 | 3180004,1 | 2 | 2 |
| 1165 | \|A0A151P8E5\|A0A151P8E5_ALLMI 60S acidic ribosomal protein P1 OS=Alligator mississippiensis GN=RPLP1 PE=3 SV=1 | 3174938,9 | 3 | 1 |
| 1166 | \|A0A1U8D2F1\|A0A1U8D2F1_ALLSI interferon-induced protein 44-like OS=Alligator sinensis GN=IFI44L PE=4 SV=1 | 3174761,4 | 5 | 4 |
| 1167 | \|A0A151M9H5\|A0A151M9H5_ALLMI COP9 signalosome complex subunit 2 isoform B OS=Alligator mississippiensis GN=COPS2 PE=4 SV=1 | 3161911,4 | 3 | 3 |
| 1168 | \|A0A151MHN5\|A0A151MHN5_ALLMI Uncharacterized protein OS=Alligator mississippiensis GN=Y1Q_0004592 PE=4 SV=1 | 3161702,5 | 4 | 4 |
| 1169 | \|A0A151NZ47\|A0A151NZ47_ALLMI Formin-H-like OS=Alligator mississippiensis GN=Y1Q_0002713 PE=4 SV=1 | 3125486,6 | 2 | 1 |
| 1170 | \|A0A1U8D7Y9\|A0A1U8D7Y9_ALLSI inorganic pyrophosphatase OS=Alligator sinensis GN=PPA1 PE=4 SV=1 | 3095995,2 | 3 | 1 |
| 1171 | \|A0A151NW07\|A0A151NW07_ALLMI Leucine carboxyl methyltransferase 1 OS=Alligator mississippiensis GN=LCMT1 PE=4 SV=1 | 3083494,5 | 1 | 1 |
| 1172 | \|A0A151N9L4\|A0A151N9L4_ALLMI Tubulin alpha chain OS=Alligator mississippiensis GN=TUBAL3 PE=3 SV=1 | 3066101,5 | 10 | 5 |
| 1173 | \|A0A151M5A1\|A0A151M5A1_ALLMI 26S proteasome non-ATPase regulatory subunit 4 isoform A OS=Alligator mississippiensis GN=PSMD4 PE=4 SV=1 | 3065734,0 | 5 | 3 |
| 1174 | \|A0A1U8DL01\|A0A1U8DL01_ALLSI arf-GAP with coiled-coil, ANK repeat and PH domain-containing protein 2 OS=Alligator sinensis GN=ACAP2 PE=4 SV=1 | 3065239,9 | 4 | 3 |
| 1175 | \|A0A151NWZ1\|A0A151NWZ1_ALLMI Uncharacterized protein OS=Alligator mississippiensis GN=Y1Q_0007002 PE=4 SV=1 | 3047155,7 | 3 | 2 |
| 1176 | \|A0A1U7RXU8\|A0A1U7RXU8_ALLSI protein FAM107B OS=Alligator sinensis GN=FAM107B PE=4 SV=1 | 3041592,0 | 2 | 1 |
| 1177 | \|Q9PU79\|Q9PU79_CRONI Pyruvate kinase (Fragment) OS=Crocodylus niloticus PE=2 SV=1 | 2990275,5 | 4 | 1 |
| 1178 | \|A0A1U7RQF3\|A0A1U7RQF3_ALLSI mesothelin-like OS=Alligator sinensis GN=LOC102368124 PE=4 SV=1 | 2982349,4 | 2 | 2 |
| 1179 | \|A0A1U7S530\|A0A1U7S530_ALLSI cysteine-rich secretory protein 2 OS=Alligator sinensis GN=CRISP2 PE=3 SV=1 | 2958575,4 | 2 | 1 |
| 1180 | \|A0A1U8DKB2\|A0A1U8DKB2_ALLSI serrate RNA effector molecule homolog OS=Alligator sinensis GN=SRRT PE=4 SV=1 | 2898284,0 | 2 | 2 |
| 1181 | \|A0A151MCJ7\|A0A151MCJ7_ALLMI Perilipin OS=Alligator mississippiensis GN=PLIN4 PE=3 SV=1 | 2894003,2 | 1 | 1 |
| 1182 | \|A0A1U7RIM8\|A0A1U7RIM8_ALLSI costars family protein ABRACL OS=Alligator sinensis GN=ABRACL PE=4 SV=1 | 2883674,7 | 7 | 5 |
| 1183 | \|A0A151MKW5\|A0A151MKW5_ALLMI AP-1 complex subunit beta-1 OS=Alligator mississippiensis GN=Y1Q_0002595 PE=4 SV=1 | 2857420,6 | 2 | 2 |
| 1184 | \|A0A151NUE0\|A0A151NUE0_ALLMI Pleckstrin-like proteiny domain-containing family S member 1 isoform B OS=Alligator mississippiensis GN=PLEKHS1-1 PE=4 SV=1 | 2856512,3 | 7 | 3 |
| 1185 | \|A0A1U7S8G5\|A0A1U7S8G5_ALLSI mucin-1 OS=Alligator sinensis GN=MUC1 PE=4 SV=1 | 2847428,9 | 3 | 1 |
| 1186 | \|A0A151MY86\|A0A151MY86_ALLMI Uncharacterized protein OS=Alligator mississippiensis GN=Y1Q_0022713 PE=3 SV=1 | 2837631,6 | 3 | 3 |
| 1187 | \|A0A1U8DGY3\|A0A1U8DGY3_ALLSI ADP-ribosylation factor-binding protein GGA1 OS=Alligator sinensis GN=GGA1 PE=4 SV=1 | 2831669,1 | 3 | 3 |
| 1188 | \|A0A1U7S934\|A0A1U7S934_ALLSI X-ray repair cross-complementing protein 6 isoform X2 OS=Alligator sinensis GN=XRCC6 PE=4 SV=1 | 2826905,9 | 5 | 4 |
| 1189 | \|A0A151ME35\|A0A151ME35_ALLMI Uncharacterized protein OS=Alligator mississippiensis GN=Y1Q_0003272 PE=3 SV=1 | 2820698,1 | 4 | 2 |
| 1190 | \|A0A151MJT7\|A0A151MJT7_ALLMI Uncharacterized protein OS=Alligator mississippiensis GN=Y1Q_0014088 PE=4 SV=1 | 2820027,6 | 3 | 3 |
| 1191 | \|A0A1U8DXL1\|A0A1U8DXL1_ALLSI Biliverdin reductase A OS=Alligator sinensis GN=BLVRA PE=3 SV=1 | 2800585,5 | 3 | 2 |
| 1192 | \|A0A1U8DB17\|A0A1U8DB17_ALLSI protein phosphatase 1 regulatory subunit 1B isoform X1 OS=Alligator sinensis GN=PPP1R1B PE=4 SV=1 | 2788662,4 | 1 | 1 |
| 1193 | \|A0A151N471\|A0A151N471_ALLMI Glutathione S-transferase theta-1-like OS=Alligator mississippiensis GN=GSTT1L PE=3 SV=1 | 2782522,8 | 5 | 3 |
| 1194 | \|A0A1U8DDJ6\|A0A1U8DDJ6_ALLSI LOW QUALITY PROTEIN: alpha-2-macroglobulin-like OS=Alligator sinensis GN=LOC106721825 PE=4 SV=1 | 2765135,4 | 3 | 1 |
| 1195 | \|A0A151NDE2\|A0A151NDE2_ALLMI Corticosteroid 11-beta-dehydrogenase isozyme 2 isoform A OS=Alligator mississippiensis GN=HSD11B2 PE=4 SV=1 | 2697548,9 | 2 | 2 |
| 1196 | \|A0A151LZC1\|A0A151LZC1_ALLMI E3 ubiquitin-protein ligase HECTD3 OS=Alligator mississippiensis GN=HECTD3 PE=4 SV=1 | 2687453,6 | 3 | 3 |
| 1197 | \|A0A1U7SLP3\|A0A1U7SLP3_ALLSI LOW QUALITY PROTEIN: fibrinogen alpha chain OS=Alligator sinensis GN=FGA PE=4 SV=1 | 2686586,5 | 4 | 2 |
| 1198 | \|A0A1U8D9Q5\|A0A1U8D9Q5_ALLSI ubiquitin fusion degradation protein 1 homolog isoform X1 OS=Alligator sinensis GN=UFD1L PE=4 SV=1 | 2676844,4 | 3 | 2 |
| 1199 | \|A0A151MWD8\|A0A151MWD8_ALLMI Katanin p60 ATPase-containing subunit A-like 2 OS=Alligator mississippiensis GN=KATNAL2 PE=3 SV=1 | 2662231,4 | 2 | 1 |
| 1200 | \|A0A151M9V6\|A0A151M9V6_ALLMI Alpha-mannosidase OS=Alligator mississippiensis GN=MAN2C1 PE=3 SV=1 | 2620200,0 | 5 | 4 |
| 1201 | \|A0A151M1K3\|A0A151M1K3_ALLMI Uncharacterized protein OS=Alligator mississippiensis GN=Y1Q_0008516 PE=4 SV=1 | 2560878,4 | 1 | 1 |
| 1202 | \|A0A151P502\|A0A151P502_ALLMI Cathelicidin-related peptide Oh-Cath-like OS=Alligator mississippiensis GN=Y1Q_0010284 PE=3 SV=1 | 2537012,2 | 2 | 1 |
| 1203 | \|A0A151NE39\|A0A151NE39_ALLMI Protein farnesyltransferase/geranylgeranyltransferase type-1 subunit alpha isoform A OS=Alligator mississippiensis GN=FNTA PE=4 SV=1 | 2509111,7 | 2 | 1 |
| 1204 | \|A0A1U7SPV1\|A0A1U7SPV1_ALLSI phosphorylase b kinase regulatory subunit beta isoform X3 OS=Alligator sinensis GN=PHKB PE=4 SV=1 | 2498385,9 | 5 | 5 |
| 1205 | \|A0A151N217\|A0A151N217_ALLMI Mesothelin-like protein OS=Alligator mississippiensis GN=MSLNL PE=4 SV=1 | 2463999,6 | 3 | 2 |
| 1206 | \|A0A151N3F8\|A0A151N3F8_ALLMI ADP-ribosylation factor 6 OS=Alligator mississippiensis GN=ARF6 PE=3 SV=1 | 2446754,4 | 2 | 2 |
| 1207 | \|A0A1U7RZ28\|A0A1U7RZ28_ALLSI UDP-N-acetylhexosamine pyrophosphorylase-like protein 1 OS=Alligator sinensis GN=UAP1L1 PE=4 SV=1 | 2445925,8 | 2 | 2 |
| 1208 | \|A0A151PD42\|A0A151PD42_ALLMI Eukaryotic translation initiation factor 1 OS=Alligator mississippiensis GN=EIF1 PE=4 SV=1 | 2410549,7 | 5 | 3 |
| 1209 | \|A0A151MBW9\|A0A151MBW9_ALLMI Eukaryotic translation initiation factor 4 gamma 2 isoform A OS=Alligator mississippiensis GN=EIF4G2 PE=4 SV=1 | 2396994,3 | 2 | 1 |
| 1210 | \|A0A1U8DQH4\|A0A1U8DQH4_ALLSI BUB3-interacting and GLEBS motif-containing protein ZNF207 isoform X1 OS=Alligator sinensis GN=ZNF207 PE=4 SV=1 | 2384020,4 | 1 | 1 |
| 1211 | \|A0A151N7Y2\|A0A151N7Y2_ALLMI 1,2-dihydroxy-3-keto-5-methylthiopentene dioxygenase OS=Alligator mississippiensis GN=ADI1 PE=3 SV=1 | 2356617,4 | 3 | 1 |
| 1212 | \|A0A1U8E0N3\|A0A1U8E0N3_ALLSI DAZ-associated protein 1 isoform X3 OS=Alligator sinensis GN=DAZAP1 PE=4 SV=1 | 2353253,2 | 2 | 1 |
| 1213 | \|A0A1U7SAF6\|A0A1U7SAF6_ALLSI cytochrome b5 OS=Alligator sinensis GN=LOC102370405 PE=3 SV=1 | 2263252,4 | 1 | 1 |
| 1214 | \|A0A151NDI1\|A0A151NDI1_ALLMI Uncharacterized protein OS=Alligator mississippiensis GN=Y1Q_0016610 PE=3 SV=1 | 2227173,5 | 2 | 2 |
| 1215 | \|A0A1U7S2T9\|A0A1U7S2T9_ALLSI AP-1 complex subunit mu-2 OS=Alligator sinensis GN=AP1M2 PE=3 SV=1 | 2189668,0 | 3 | 2 |
| 1216 | \|A0A151M3P2\|A0A151M3P2_ALLMI Dynactin subunit 4 OS=Alligator mississippiensis GN=DCTN4 PE=4 SV=1 | 2179705,6 | 2 | 2 |
| 1217 | \|A0A151MGD4\|A0A151MGD4_ALLMI Eukaryotic translation initiation factor 5A OS=Alligator mississippiensis GN=EIF5A2 PE=3 SV=1 | 2099324,8 | 2 | 2 |
| 1218 | \|A0A151N0R7\|A0A151N0R7_ALLMI Trafficking protein particle complex subunit 2 OS=Alligator mississippiensis GN=TRAPPC2 PE=4 SV=1 | 2093994,2 | 2 | 2 |
| 1219 | \|A0A151MJA3\|A0A151MJA3_ALLMI Adaptin ear-binding coat-associated protein 2 OS=Alligator mississippiensis GN=NECAP2 PE=4 SV=1 | 2076035,3 | 3 | 2 |
| 1220 | \|A0A151P2N9\|A0A151P2N9_ALLMI Catenin beta-1 isoform B OS=Alligator mississippiensis GN=CTNNB1 PE=4 SV=1 | 2048798,4 | 2 | 1 |
| 1221 | \|A0A151MCR1\|A0A151MCR1_ALLMI Retinol-binding protein 5 isoform A OS=Alligator mississippiensis GN=RBP5 PE=3 SV=1 | 1984868,0 | 2 | 1 |
| 1222 | \|A0A151NAX0\|A0A151NAX0_ALLMI Eukaryotic translation initiation factor 2A OS=Alligator mississippiensis GN=EIF2A PE=3 SV=1 | 1976534,9 | 2 | 1 |
| 1223 | \|A0A1U7SDR9\|A0A1U7SDR9_ALLSI interferon regulatory factor 6 OS=Alligator sinensis GN=IRF6 PE=3 SV=1 | 1968497,6 | 1 | 1 |
| 1224 | \|A0A151P2M3\|A0A151P2M3_ALLMI Tetranectin OS=Alligator mississippiensis GN=CLEC3B PE=4 SV=1 | 1937932,3 | 3 | 2 |
| 1225 | \|A0A151MUW8\|A0A151MUW8_ALLMI Adenine phosphoribosyltransferase-like OS=Alligator mississippiensis GN=APRT PE=3 SV=1 | 1937248,7 | 1 | 1 |
| 1226 | \|A0A1U7SQX1\|A0A1U7SQX1_ALLSI selenium-binding protein 1-like OS=Alligator sinensis GN=LOC102388298 PE=4 SV=1 | 1915786,0 | 3 | 2 |
| 1227 | \|A0A151MNR0\|A0A151MNR0_ALLMI Acyl-protein thioesterase 1 OS=Alligator mississippiensis GN=LYPLA1 PE=4 SV=1 | 1905390,8 | 2 | 1 |
| 1228 | \|A0A151MWA4\|A0A151MWA4_ALLMI N-alpha-acetyltransferase 50 isoform B OS=Alligator mississippiensis GN=NAA50-1 PE=4 SV=1 | 1905061,5 | 2 | 2 |
| 1229 | \|A0A151M446\|A0A151M446_ALLMI 3-hydroxyacyl-CoA dehydrogenase type-2 OS=Alligator mississippiensis GN=HSD17B10 PE=3 SV=1 | 1902572,9 | 3 | 2 |
| 1230 | \|A0A151P6N4\|A0A151P6N4_ALLMI Tetraspanin OS=Alligator mississippiensis GN=CD9 PE=3 SV=1 | 1880526,4 | 4 | 1 |
| 1231 | \|A0A151MSP4\|A0A151MSP4_ALLMI Golgi apparatus protein 1 OS=Alligator mississippiensis GN=GLG1 PE=4 SV=1 | 1874460,5 | 3 | 2 |
| 1232 | \|A0A151MTA8\|A0A151MTA8_ALLMI Glutamate dehydrogenase OS=Alligator mississippiensis GN=GLUD1 PE=3 SV=1 | 1869021,3 | 3 | 3 |
| 1233 | \|A0A151PCR8\|A0A151PCR8_ALLMI Cullin-2 OS=Alligator mississippiensis GN=CUL2 PE=3 SV=1 | 1867036,9 | 1 | 1 |
| 1234 | \|A0A1U7RM31\|A0A1U7RM31_ALLSI immunoglobulin-binding protein 1 OS=Alligator sinensis GN=IGBP1 PE=4 SV=1 | 1858608,1 | 1 | 1 |
| 1235 | \|A0A151MCV4\|A0A151MCV4_ALLMI Omega-amidase NIT2 OS=Alligator mississippiensis GN=NIT2 PE=4 SV=1 | 1841926,9 | 3 | 3 |
| 1236 | \|A0A151MYG3\|A0A151MYG3_ALLMI SET and MYND domain-containing protein 5 OS=Alligator mississippiensis GN=SMYD5 PE=4 SV=1 | 1833918,7 | 2 | 2 |
| 1237 | \|A0A1U7SA28\|A0A1U7SA28_ALLSI calcyphosin-like protein isoform X2 OS=Alligator sinensis GN=CAPSL PE=4 SV=1 | 1830097,0 | 4 | 2 |
| 1238 | \|A0A1U7RBG4\|A0A1U7RBG4_ALLSI aldehyde dehydrogenase family 16 member A1 isoform X2 OS=Alligator sinensis GN=ALDH16A1 PE=4 SV=1 | 1809259,5 | 3 | 2 |
| 1239 | \|A0A1U7S8Z9\|A0A1U7S8Z9_ALLSI cysteine and glycine-rich protein 1 OS=Alligator sinensis GN=CSRP1 PE=4 SV=1 | 1805872,2 | 2 | 1 |
| 1240 | \|A0A151P8G5\|A0A151P8G5_ALLMI Apolipoprotein D OS=Alligator mississippiensis GN=Y1Q_0011214 PE=3 SV=1 | 1797896,4 | 2 | 2 |
| 1241 | \|A0A151N0N7\|A0A151N0N7_ALLMI Synapse-associated protein 1 OS=Alligator mississippiensis GN=SYAP1 PE=4 SV=1 | 1790191,0 | 2 | 2 |
| 1242 | \|A0A151MC97\|A0A151MC97_ALLMI Ubiquitin-conjugating enzyme E2 variant 3 OS=Alligator mississippiensis GN=UEVLD PE=4 SV=1 | 1787888,5 | 2 | 1 |
| 1243 | \|A0A151NB67\|A0A151NB67_ALLMI Programmed cell death protein 10 OS=Alligator mississippiensis GN=PDCD10 PE=4 SV=1 | 1783051,4 | 2 | 2 |
| 1244 | \|A0A151M1I6\|A0A151M1I6_ALLMI Uncharacterized protein OS=Alligator mississippiensis GN=Y1Q_0008495 PE=3 SV=1 | 1778070,2 | 2 | 2 |
| 1245 | \|A0A151MJF5\|A0A151MJF5_ALLMI Autophagy protein 5 OS=Alligator mississippiensis GN=ATG5 PE=3 SV=1 | 1763313,2 | 1 | 1 |
| 1246 | \|A0A151N1A2\|A0A151N1A2_ALLMI THUMP domain-containing protein 1 OS=Alligator mississippiensis GN=THUMPD1 PE=4 SV=1 | 1726680,5 | 2 | 2 |
| 1247 | \|A0A151NBY3\|A0A151NBY3_ALLMI Ubiquitin-fold modifier 1 OS=Alligator mississippiensis GN=UFM1 PE=4 SV=1 | 1722246,0 | 4 | 1 |
| 1248 | \|A0A1U7RUH2\|A0A1U7RUH2_ALLSI serine/threonine-protein phosphatase 6 regulatory subunit 3 isoform X3 OS=Alligator sinensis GN=PPP6R3 PE=4 SV=1 | 1695464,5 | 1 | 1 |
| 1249 | >sp\|Q9W790\|TCPA_PALPA T-complex protein 1 subunit alpha OS=Paleosuchus palpebrosus GN=TCP1 PE=2 SV=1 | 1686290,5 | 2 | 2 |
| 1250 | \|A0A151MD07\|A0A151MD07_ALLMI Autophagy-related protein 3 OS=Alligator mississippiensis GN=ATG3 PE=3 SV=1 | 1668868,2 | 3 | 3 |
| 1251 | \|A0A151PEJ8\|A0A151PEJ8_ALLMI Uncharacterized protein OS=Alligator mississippiensis GN=Y1Q_0001247 PE=3 SV=1 | 1643124,5 | 1 | 1 |
| 1252 | \|A0A151NHX6\|A0A151NHX6_ALLMI 2-amino-3-ketobutyrate coenzyme A ligase, mitochondrial OS=Alligator mississippiensis GN=GCAT PE=3 SV=1 | 1637818,7 | 1 | 1 |
| 1253 | \|A0A1U8CV70\|A0A1U8CV70_ALLSI serine/threonine-protein phosphatase 2A 56 kDa regulatory subunit epsilon isoform isoform X2 OS=Alligator sinensis GN=PPP2R5E PE=4 SV=1 | 1626512,4 | 2 | 2 |
| 1254 | >sp\|Q98SL2\|LDHA_CAICA L-lactate dehydrogenase A chain OS=Caiman crocodilus apaporiensis GN=LDHA PE=2 SV=3 | 1621680,7 | 1 | 1 |
| 1255 | \|A0A151N5S7\|A0A151N5S7_ALLMI Serum albumin OS=Alligator mississippiensis GN=ALB PE=3 SV=1 | 1611702,4 | 3 | 1 |
| 1256 | \|A0A151M2A7\|A0A151M2A7_ALLMI Superoxide dismutase OS=Alligator mississippiensis GN=SOD2 PE=3 SV=1 | 1610681,3 | 2 | 1 |
| 1257 | \|A0A1U7RX47\|A0A1U7RX47_ALLSI heat shock protein beta-8 OS=Alligator sinensis GN=HSPB8 PE=3 SV=1 | 1540364,8 | 2 | 1 |
| 1258 | \|A0A151M759\|A0A151M759_ALLMI RUN and FYVE domain-containing protein 1 isoform A OS=Alligator mississippiensis GN=RUFY1 PE=4 SV=1 | 1522098,5 | 3 | 2 |
| 1259 | \|V9TNY5\|V9TNY5_CAICR Complement component 4 (Fragment) OS=Caiman crocodilus GN=C4 PE=2 SV=1 | 1522085,3 | 5 | 3 |
| 1260 | \|A0A151MSW0\|A0A151MSW0_ALLMI Uncharacterized protein OS=Alligator mississippiensis GN=Y1Q_0005172 PE=4 SV=1 | 1518890,7 | 5 | 3 |
| 1261 | \|A0A151N607\|A0A151N607_ALLMI Dehydrogenase/reductase SDR family member 11 OS=Alligator mississippiensis GN=DHRS11 PE=3 SV=1 | 1486937,0 | 2 | 2 |
| 1262 | \|A0A151NYS0\|A0A151NYS0_ALLMI Uncharacterized protein OS=Alligator mississippiensis GN=Y1Q_0002442 PE=4 SV=1 | 1425097,6 | 2 | 1 |
| 1263 | \|A0A151MK90\|A0A151MK90_ALLMI U6 snRNA-associated Sm-like protein LSm3 OS=Alligator mississippiensis GN=LSM3 PE=4 SV=1 | 1423931,7 | 2 | 1 |
| 1264 | \|A0A151NS04\|A0A151NS04_ALLMI Vacuolar protein sorting-associated protein 37B OS=Alligator mississippiensis GN=VPS37B PE=4 SV=1 | 1334846,0 | 1 | 1 |
| 1265 | \|A0A1U8DZG5\|A0A1U8DZG5_ALLSI adapter molecule crk OS=Alligator sinensis GN=CRK PE=4 SV=1 | 1333247,0 | 1 | 1 |
| 1266 | \|A0A151NL85\|A0A151NL85_ALLMI Ganglioside GM2 activator OS=Alligator mississippiensis GN=GM2A PE=4 SV=1 | 1324738,4 | 1 | 1 |
| 1267 | \|A0A151N6P8\|A0A151N6P8_ALLMI Cytosolic Fe-S cluster assembly factor NUBP2 OS=Alligator mississippiensis GN=NUBP2 PE=3 SV=1 | 1316045,1 | 1 | 1 |
| 1268 | \|A0A151M494\|A0A151M494_ALLMI UMP-CMP kinase isoform B OS=Alligator mississippiensis GN=CMPK1 PE=3 SV=1 | 1315579,6 | 7 | 5 |
| 1269 | \|A0A1U7SH21\|A0A1U7SH21_ALLSI reticulocalbin-1 OS=Alligator sinensis GN=RCN1 PE=4 SV=1 | 1281193,6 | 2 | 2 |
| 1270 | \|A0A1U7RQ36\|A0A1U7RQ36_ALLSI L-lactate dehydrogenase OS=Alligator sinensis GN=LOC102383352 PE=3 SV=1 | 1275103,0 | 1 | 1 |
| 1271 | \|A0A151P0B8\|A0A151P0B8_ALLMI Amino-terminal enhancer of split OS=Alligator mississippiensis GN=AES PE=4 SV=1 | 1267107,5 | 2 | 1 |
| 1272 | \|A0A1U7RT33\|A0A1U7RT33_ALLSI Sulfotransferase OS=Alligator sinensis GN=LOC102376769 PE=3 SV=1 | 1264842,6 | 3 | 2 |
| 1273 | \|A0A151NUS7\|A0A151NUS7_ALLMI General transcription factor IIF subunit 1 OS=Alligator mississippiensis GN=GTF2F1 PE=3 SV=1 | 1260506,4 | 2 | 1 |
| 1274 | \|A0A151NQ69\|A0A151NQ69_ALLMI Vacuolar protein sorting-associated protein 26B OS=Alligator mississippiensis GN=VPS26B PE=4 SV=1 | 1246326,3 | 2 | 2 |
| 1275 | \|A0A151N7J8\|A0A151N7J8_ALLMI Coagulation factor IX OS=Alligator mississippiensis GN=F9 PE=3 SV=1 | 1231276,8 | 2 | 2 |
| 1276 | \|A0A151MP05\|A0A151MP05_ALLMI Uncharacterized protein OS=Alligator mississippiensis GN=Y1Q_0000208 PE=4 SV=1 | 1214249,9 | 2 | 1 |
| 1277 | \|A0A1U7SDH7\|A0A1U7SDH7_ALLSI Tubulin alpha chain OS=Alligator sinensis GN=LOC102384635 PE=3 SV=1 | 1207275,9 | 5 | 2 |
| 1278 | \|A0A151PBB9\|A0A151PBB9_ALLMI Poliovirus receptor-related protein 2 OS=Alligator mississippiensis GN=PVRL2 PE=4 SV=1 | 1206337,1 | 1 | 1 |
| 1279 | \|A0A1U7R8X9\|A0A1U7R8X9_ALLSI protein archease OS=Alligator sinensis GN=ZBTB8OS PE=4 SV=1 | 1194704,7 | 6 | 3 |
| 1280 | \|A0A151NR35\|A0A151NR35_ALLMI Drebrin-like OS=Alligator mississippiensis GN=Y1Q_0019911 PE=4 SV=1 | 1177367,5 | 1 | 1 |
| 1281 | \|A0A151M890\|A0A151M890_ALLMI Kinesin-like protein KIF27 OS=Alligator mississippiensis GN=KIF27 PE=3 SV=1 | 1174480,0 | 2 | 1 |
| 1282 | \|A0A1U7SK08\|A0A1U7SK08_ALLSI neuroblast differentiation-associated protein AHNAK isoform X1 OS=Alligator sinensis GN=AHNAK PE=4 SV=1 | 1113038,9 | 2 | 2 |
| 1283 | \|A0A151N542\|A0A151N542_ALLMI Calumenin isoform C OS=Alligator mississippiensis GN=CALU PE=4 SV=1 | 1105498,4 | 1 | 1 |
| 1284 | \|A0A151PJ76\|A0A151PJ76_ALLMI 40S ribosomal protein S27 OS=Alligator mississippiensis GN=RPS27 PE=3 SV=1 | 1082877,9 | 1 | 1 |
| 1285 | \|A0A1U8D4Q1\|A0A1U8D4Q1_ALLSI alpha-1-antitrypsin-like OS=Alligator sinensis GN=LOC102370638 PE=3 SV=1 | 1049057,0 | 1 | 1 |
| 1286 | \|A0A151MQD9\|A0A151MQD9_ALLMI Acyl-CoA-binding domain-containing protein 7 OS=Alligator mississippiensis GN=ACBD7 PE=4 SV=1 | 1003786,0 | 2 | 2 |
| 1287 | \|A0A151N6B8\|A0A151N6B8_ALLMI Myosin light polypeptide 6 isoform A OS=Alligator mississippiensis GN=MYL6-1 PE=4 SV=1 | 987776,8 | 1 | 1 |
| 1288 | \|A0A1U8DQ78\|A0A1U8DQ78_ALLSI guanylate-binding protein 1-like isoform X2 OS=Alligator sinensis GN=LOC102385888 PE=4 SV=1 | 972103,4 | 1 | 1 |
| 1289 | \|A0A151NM91\|A0A151NM91_ALLMI Ubiquitin carboxyl-terminal hydrolase 10 OS=Alligator mississippiensis GN=USP10 PE=3 SV=1 | 958971,5 | 2 | 2 |
| 1290 | \|A0A151P9W8\|A0A151P9W8_ALLMI Protein NOXP20 isoform B OS=Alligator mississippiensis GN=FAM114A1 PE=4 SV=1 | 958576,4 | 1 | 1 |
| 1291 | \|A0A151NHV0\|A0A151NHV0_ALLMI NHP2-like protein 1 OS=Alligator mississippiensis GN=SNU13 PE=4 SV=1 | 956835,8 | 2 | 1 |
| 1292 | \|A0A1U7SD72\|A0A1U7SD72_ALLSI acidic leucine-rich nuclear phosphoprotein 32 family member E OS=Alligator sinensis GN=ANP32E PE=4 SV=1 | 956387,6 | 1 | 1 |
| 1293 | \|A0A151NC03\|A0A151NC03_ALLMI GMP synthase [glutamine-hydrolyzing] isoform B OS=Alligator mississippiensis GN=GMPS PE=4 SV=1 | 947837,8 | 2 | 2 |
| 1294 | \|A0A1U7S3L6\|A0A1U7S3L6_ALLSI Peptidylprolyl isomerase OS=Alligator sinensis GN=FKBP1A PE=4 SV=1 | 934475,2 | 2 | 1 |
| 1295 | \|A0A151MRM3\|A0A151MRM3_ALLMI Ras-related protein Rab-10 OS=Alligator mississippiensis GN=RAB10 PE=4 SV=1 | 920484,1 | 1 | 1 |
| 1296 | \|A0A1U8DJZ1\|A0A1U8DJZ1_ALLSI uncharacterized protein LOC102379723 OS=Alligator sinensis GN=LOC102379723 PE=4 SV=1 | 914688,7 | 3 | 2 |
| 1297 | \|A0A1U7RQG4\|A0A1U7RQG4_ALLSI Tubulin beta chain OS=Alligator sinensis GN=LOC102388337 PE=3 SV=1 | 905722,7 | 3 | 2 |
| 1298 | \|A0A1U7S0Y1\|A0A1U7S0Y1_ALLSI nitrilase homolog 1 OS=Alligator sinensis GN=NIT1 PE=4 SV=1 | 905657,4 | 3 | 1 |
| 1299 | \|A0A1U7RY69\|A0A1U7RY69_ALLSI heat shock factor-binding protein 1 OS=Alligator sinensis GN=HSBP1 PE=4 SV=1 | 882079,6 | 2 | 2 |
| 1300 | \|A0A151MG62\|A0A151MG62_ALLMI Splicing factor U2AF subunit OS=Alligator mississippiensis GN=U2AF2 PE=4 SV=1 | 877942,6 | 3 | 2 |
| 1301 | \|A0A1U8D4Z0\|A0A1U8D4Z0_ALLSI 40S ribosomal protein S28 OS=Alligator sinensis GN=RPS28 PE=4 SV=1 | 866315,8 | 3 | 1 |
| 1302 | \|A0A151P3Z7\|A0A151P3Z7_ALLMI Developmentally-regulated GTP-binding protein 2 OS=Alligator mississippiensis GN=DRG2 PE=4 SV=1 | 855182,1 | 1 | 1 |
| 1303 | \|A0A151PG67\|A0A151PG67_ALLMI Uroporphyrinogen decarboxylase OS=Alligator mississippiensis GN=UROD PE=3 SV=1 | 854038,6 | 1 | 1 |
| 1304 | \|A0A151MEM0\|A0A151MEM0_ALLMI Mesencephalic astrocyte-derived neurotrophic factor isoform A OS=Alligator mississippiensis GN=MANF PE=4 SV=1 | 853869,7 | 1 | 1 |
| 1305 | \|A0A151NJ44\|A0A151NJ44_ALLMI Vitellogenin-1 OS=Alligator mississippiensis GN=VTG1 PE=4 SV=1 | 851026,6 | 1 | 1 |
| 1306 | \|A0A151NVW3\|A0A151NVW3_ALLMI Protein NDRG3 isoform B OS=Alligator mississippiensis GN=NDRG3 PE=4 SV=1 | 848760,1 | 3 | 2 |
| 1307 | \|A0A151NY26\|A0A151NY26_ALLMI Pre-mRNA-processing factor 19 OS=Alligator mississippiensis GN=PRPF19 PE=4 SV=1 | 836159,4 | 4 | 2 |
| 1308 | \|A0A1U7SSL6\|A0A1U7SSL6_ALLSI kunitz-type protease inhibitor 4 OS=Alligator sinensis GN=SPINT4 PE=4 SV=1 | 823601,2 | 1 | 1 |
| 1309 | \|A0A1U7SKA7\|A0A1U7SKA7_ALLSI alanine aminotransferase 1 OS=Alligator sinensis GN=GPT PE=4 SV=1 | 812294,6 | 3 | 3 |
| 1310 | \|A0A151MUX2\|A0A151MUX2_ALLMI Protein-L-isoaspartate O-methyltransferase OS=Alligator mississippiensis GN=PCMT1L PE=3 SV=1 | 804128,7 | 2 | 2 |
| 1311 | \|A0A1U7RRT8\|A0A1U7RRT8_ALLSI protein FAM49B isoform X2 OS=Alligator sinensis GN=FAM49B PE=4 SV=1 | 792097,3 | 2 | 2 |
| 1312 | \|A0A151MYW1\|A0A151MYW1_ALLMI Ig epsilon chain C region OS=Alligator mississippiensis GN=Y1Q_0005958 PE=4 SV=1 | 774101,2 | 3 | 1 |
| 1313 | \|A0A151NTG5\|A0A151NTG5_ALLMI Dynein light chain Tctex-type 3 OS=Alligator mississippiensis GN=DYNLT3 PE=4 SV=1 | 771928,8 | 1 | 1 |
| 1314 | \|A0A151MPN3\|A0A151MPN3_ALLMI DNA-directed RNA polymerase subunit beta OS=Alligator mississippiensis GN=POLR2B PE=3 SV=1 | 771901,6 | 1 | 1 |
| 1315 | \|A0A151P734\|A0A151P734_ALLMI Polypeptide N-acetylgalactosaminyltransferase OS=Alligator mississippiensis GN=GALNT8 PE=3 SV=1 | 749483,9 | 3 | 2 |
| 1316 | \|A0A151NAI8\|A0A151NAI8_ALLMI Cullin-3 OS=Alligator mississippiensis GN=CUL3 PE=3 SV=1 | 732773,6 | 2 | 2 |
| 1317 | \|A0A151PBZ2\|A0A151PBZ2_ALLMI Granulins isoform B OS=Alligator mississippiensis GN=GRN-1 PE=4 SV=1 | 727222,6 | 1 | 1 |
| 1318 | \|A0A151MTQ4\|A0A151MTQ4_ALLMI Methylosome protein 50 OS=Alligator mississippiensis GN=WDR77 PE=4 SV=1 | 722085,1 | 1 | 1 |
| 1319 | \|A0A151NFQ9\|A0A151NFQ9_ALLMI Uncharacterized protein OS=Alligator mississippiensis GN=Y1Q_0008055 PE=4 SV=1 | 710636,0 | 1 | 1 |
| 1320 | \|A0A151P2H4\|A0A151P2H4_ALLMI Proteasome inhibitor PI31 subunit OS=Alligator mississippiensis GN=PSMF1 PE=4 SV=1 | 698063,5 | 1 | 1 |
| 1321 | \|A0A151NVU3\|A0A151NVU3_ALLMI Tankyrase-1-binding protein isoform C OS=Alligator mississippiensis GN=TNKS1BP1-1 PE=4 SV=1 | 694693,1 | 1 | 1 |
| 1322 | \|A0A151P978\|A0A151P978_ALLMI Ribose-5-phosphate isomerase OS=Alligator mississippiensis GN=RPIA PE=3 SV=1 | 658693,2 | 3 | 3 |
| 1323 | \|A0A1U7S1Y2\|A0A1U7S1Y2_ALLSI Phosphoinositide phospholipase C OS=Alligator sinensis GN=PLCD1 PE=4 SV=1 | 639400,7 | 1 | 1 |
| 1324 | \|A0A151MM69\|A0A151MM69_ALLMI Carbonic anhydrase 15-like OS=Alligator mississippiensis GN=Y1Q_0000151 PE=4 SV=1 | 631712,9 | 1 | 1 |
| 1325 | \|A0A151MVM2\|A0A151MVM2_ALLMI Uncharacterized protein OS=Alligator mississippiensis GN=Y1Q_0005349 PE=3 SV=1 | 629953,5 | 1 | 1 |
| 1326 | \|A0A151NSC6\|A0A151NSC6_ALLMI N(G),N(G)-dimethylarginine dimethylaminohydrolase 2 OS=Alligator mississippiensis GN=DDAH2 PE=4 SV=1 | 606882,4 | 2 | 2 |
| 1327 | \|A0A1U7RRG0\|A0A1U7RRG0_ALLSI scaffold attachment factor B1-like OS=Alligator sinensis GN=LOC102372028 PE=4 SV=1 | 592281,9 | 2 | 2 |
| 1328 | \|A0A151MZH4\|A0A151MZH4_ALLMI 26S proteasome non-ATPase regulatory subunit 1 OS=Alligator mississippiensis GN=PSMD1 PE=4 SV=1 | 590280,9 | 1 | 1 |
| 1329 | \|A0A151MUD9\|A0A151MUD9_ALLMI Glutathione peroxidase OS=Alligator mississippiensis GN=GPX3 PE=3 SV=1 | 578506,0 | 1 | 1 |
| 1330 | \|A0A1U8DKE2\|A0A1U8DKE2_ALLSI cystathionine gamma-lyase OS=Alligator sinensis GN=CTH PE=3 SV=1 | 573536,9 | 2 | 1 |
| 1331 | \|A0A151PEL1\|A0A151PEL1_ALLMI Translation initiation factor eIF-2B subunit epsilon OS=Alligator mississippiensis GN=EIF2B5 PE=4 SV=1 | 573500,9 | 1 | 1 |
| 1332 | \|A0A151PHZ6\|A0A151PHZ6_ALLMI Protein FAM172A OS=Alligator mississippiensis GN=FAM172A PE=4 SV=1 | 556263,0 | 2 | 2 |
| 1333 | \|A0A1U7R8Z0\|A0A1U7R8Z0_ALLSI MARCKS-related protein OS=Alligator sinensis GN=MARCKSL1 PE=4 SV=1 | 516407,5 | 1 | 1 |
| 1334 | \|A0A151MSY3\|A0A151MSY3_ALLMI Adenosine kinase OS=Alligator mississippiensis GN=ADK-2 PE=4 SV=1 | 512912,2 | 1 | 1 |
| 1335 | \|A0A151MZP5\|A0A151MZP5_ALLMI Actin-related protein 2/3 complex subunit 5 OS=Alligator mississippiensis GN=ARPC5L PE=3 SV=1 | 426390,3 | 1 | 1 |
| 1336 | \|A0A151N4F7\|A0A151N4F7_ALLMI Ras-related protein Rab-35 OS=Alligator mississippiensis GN=RAB35 PE=4 SV=1 | 399357,9 | 1 | 1 |
| 1337 | \|A0A1U7R8A2\|A0A1U7R8A2_ALLSI aldehyde dehydrogenase, mitochondrial OS=Alligator sinensis GN=ALDH2 PE=3 SV=1 | 378959,8 | 2 | 1 |
| 1338 | \|A0A1U8DS91\|A0A1U8DS91_ALLSI alpha-2-macroglobulin-like protein 1 OS=Alligator sinensis GN=LOC102379584 PE=4 SV=1 | 371922,0 | 1 | 1 |
| 1339 | \|A0A1U7SKU7\|A0A1U7SKU7_ALLSI COP9 signalosome complex subunit 8 OS=Alligator sinensis GN=COPS8 PE=4 SV=1 | 357410,2 | 1 | 1 |
| 1340 | \|A0A151MWR2\|A0A151MWR2_ALLMI Uncharacterized protein OS=Alligator mississippiensis GN=Y1Q_0009778 PE=4 SV=1 | 353242,1 | 1 | 1 |
| 1341 | \|A0A1U7SL22\|A0A1U7SL22_ALLSI U6 snRNA-associated Sm-like protein LSm2 OS=Alligator sinensis GN=LSM2 PE=3 SV=1 | 304700,3 | 1 | 1 |
| 1342 | \|A0A151M4B9\|A0A151M4B9_ALLMI Transcription factor BTF3 OS=Alligator mississippiensis GN=BTF3L4 PE=3 SV=1 | 300099,3 | 1 | 1 |
| 1343 | \|A0A151N7P5\|A0A151N7P5_ALLMI Uncharacterized protein OS=Alligator mississippiensis GN=Y1Q_0009370 PE=4 SV=1 | 292796,1 | 1 | 1 |
| 1344 | \|A0A151MVS5\|A0A151MVS5_ALLMI Programmed cell death 4a OS=Alligator mississippiensis GN=PDCD4A PE=4 SV=1 | 289296,8 | 1 | 1 |
| 1345 | \|A0A151NHS8\|A0A151NHS8_ALLMI Galectin OS=Alligator mississippiensis GN=LGALS1 PE=4 SV=1 | 260024,2 | 1 | 1 |
| 1346 | \|A0A151MJ47\|A0A151MJ47_ALLMI F-actin-capping protein subunit beta OS=Alligator mississippiensis GN=CAPZB PE=4 SV=1 | 259068,9 | 1 | 1 |
| 1347 | \|A0A151MLL5\|A0A151MLL5_ALLMI Sulfotransferase OS=Alligator mississippiensis GN=SULT1ST6 PE=3 SV=1 | 234559,9 | 1 | 1 |
| 1348 | \|A0A151LZ63\|A0A151LZ63_ALLMI ADP-ribosylation factor-like protein 1 OS=Alligator mississippiensis GN=ARL1 PE=3 SV=1 | 217577,3 | 2 | 2 |
| 1349 | \|A0A151PCD6\|A0A151PCD6_ALLMI ERI1 exoribonuclease 3 OS=Alligator mississippiensis GN=ERI3 PE=4 SV=1 | 213250,5 | 2 | 1 |
| 1350 | \|A0A151PAP3\|A0A151PAP3_ALLMI Superoxide dismutase [Cu-Zn] OS=Alligator mississippiensis GN=SOD3 PE=3 SV=1 | 206984,4 | 1 | 1 |
| 1351 | \|A0A1U7SKR8\|A0A1U7SKR8_ALLSI S-methyl-5'-thioadenosine phosphorylase OS=Alligator sinensis GN=MTAP PE=3 SV=1 | 199578,8 | 1 | 1 |
| 1352 | \|A0A151MSR1\|A0A151MSR1_ALLMI Cleavage and polyadenylation specificity factor subunit 5 OS=Alligator mississippiensis GN=NUDT21 PE=4 SV=1 | 171784,6 | 1 | 1 |
| 1353 | \|A0A151M582\|A0A151M582_ALLMI Tissue factor OS=Alligator mississippiensis GN=F3 PE=4 SV=1 | 155295,7 | 1 | 1 |
| 1354 | \|Q8JIW5\|Q8JIW5_CAICR Glyceraldehyde-3-phosphate dehydrogenase (Fragment) OS=Caiman crocodilus GN=GAPDH PE=2 SV=1 | 151988,1 | 2 | 1 |
| 1355 | \|A0A151NWQ9\|A0A151NWQ9_ALLMI Protein phosphatase 1 regulatory subunit 21 OS=Alligator mississippiensis GN=PPP1R21 PE=4 SV=1 | 132750,6 | 1 | 1 |
| 1356 | \|A0A151N9R5\|A0A151N9R5_ALLMI Arginyl aminopeptidase-like 1 OS=Alligator mississippiensis GN=RNPEPL1 PE=4 SV=1 | 130337,2 | 1 | 1 |
| 1357 | \|A0A151P7F2\|A0A151P7F2_ALLMI Uncharacterized protein OS=Alligator mississippiensis GN=Y1Q_0022981 PE=4 SV=1 | 109155,7 | 1 | 1 |
| 1358 | \|A0A151M0V9\|A0A151M0V9_ALLMI Glycerol-3-phosphate dehydrogenase [NAD(+)] OS=Alligator mississippiensis GN=Y1Q_0011724 PE=3 SV=1 | 94201,0 | 1 | 1 |
