# Supplementary material for: The proteomics of roadside hawk (Rupornis magnirostris), broad-snouted caiman (Caiman latirostris) and loggerhead sea turtle (Caretta caretta) tears
Source: BMC Vet Res. 2020 Aug 7;16:276. doi: 10.1186/s12917-020-02495-0 (PMC7412644; doi:10.1186/s12917-020-02495-0)
Supplement: Supplementary file 3 — Additional file 3 Table 2. Proteins identified in caiman (Caiman latirostris) tears. An Orbitrap platform was used to identify the peptides, and the results were analyzed using the Alligatoridae family database . [file 12917_2020_2495_MOESM3_ESM.docx]

**Table 3. Proteins identified in sea turtle (Caretta caretta) tears.** An Orbitrap platform was used to identify the peptides, and the results were analyzed using the Testudines order database (UNIPROT KB).

| **n.** | **Description** | **Total Intensity** | **n. of spectra** | **n. of unique peptides** |
| --- | --- | --- | --- | --- |
| 1 | \|M7CCE6\|M7CCE6_CHEMY Ovotransferrin OS=Chelonia mydas GN=UY3_04363 PE=3 SV=1 | 5367463592,7 | 321 | 33 |
| 2 | \|M7BVN8\|M7BVN8_CHEMY Uncharacterized protein OS=Chelonia mydas GN=UY3_02910 PE=4 SV=1 | 1698718641,5 | 54 | 8 |
| 3 | \|M7BJL4\|M7BJL4_CHEMY Vitelline membrane outer layer protein 1 (Fragment) OS=Chelonia mydas GN=UY3_06923 PE=4 SV=1 | 1208607659,9 | 50 | 7 |
| 4 | \|M7B0H7\|M7B0H7_CHEMY Myeloperoxidase (Fragment) OS=Chelonia mydas GN=UY3_12337 PE=4 SV=1 | 755654251,1 | 26 | 14 |
| 5 | \|M7BYB4\|M7BYB4_CHEMY Deleted in malignant brain tumors 1 protein OS=Chelonia mydas GN=UY3_00607 PE=4 SV=1 | 491171293,2 | 27 | 3 |
| 6 | \|M7CCQ8\|M7CCQ8_CHEMY Ig mu chain C region OS=Chelonia mydas GN=UY3_04248 PE=4 SV=1 | 487365433,6 | 75 | 11 |
| 7 | \|M7AJL3\|M7AJL3_CHEMY Actin, cytoplasmic 2 OS=Chelonia mydas GN=UY3_17663 PE=3 SV=1 | 228152622,3 | 62 | 22 |
| 8 | \|M7BTZ2\|M7BTZ2_CHEMY Protein-glutamine gamma-glutamyltransferase 4 (Fragment) OS=Chelonia mydas GN=UY3_11308 PE=4 SV=1 | 188068665,5 | 46 | 22 |
| 9 | \|M7B4N9\|M7B4N9_CHEMY Serum albumin OS=Chelonia mydas GN=UY3_12388 PE=3 SV=1 | 168132787,7 | 71 | 29 |
| 10 | \|M7ANA5\|M7ANA5_CHEMY Alpha-enolase OS=Chelonia mydas GN=UY3_17006 PE=3 SV=1 | 151099077,7 | 63 | 22 |
| 11 | \|M7B9P6\|M7B9P6_CHEMY Keratin, type I cytoskeletal 19 OS=Chelonia mydas GN=UY3_09153 PE=3 SV=1 | 150940909,1 | 45 | 13 |
| 12 | \|M7ARI6\|M7ARI6_CHEMY Extracellular matrix protein 1 OS=Chelonia mydas GN=UY3_14918 PE=4 SV=1 | 146772765,7 | 40 | 7 |
| 13 | \|M7C118\|M7C118_CHEMY Peroxiredoxin-1 OS=Chelonia mydas GN=UY3_08776 PE=4 SV=1 | 137980304,7 | 63 | 16 |
| 14 | \|M7BMM5\|M7BMM5_CHEMY Keratin, type II cytoskeletal cochleal OS=Chelonia mydas GN=UY3_05867 PE=3 SV=1 | 129715619,5 | 34 | 18 |
| 15 | \|M7AYN0\|M7AYN0_CHEMY Clusterin OS=Chelonia mydas GN=UY3_18044 PE=3 SV=1 | 111659294,4 | 51 | 21 |
| 16 | \|M7BTE6\|M7BTE6_CHEMY Keratin, type I cytoskeletal 14 OS=Chelonia mydas GN=UY3_02222 PE=3 SV=1 | 99504493,2 | 22 | 10 |
| 17 | \|M7CBJ9\|M7CBJ9_CHEMY Niban-like protein 1 OS=Chelonia mydas GN=UY3_04665 PE=4 SV=1 | 91728989,3 | 6 | 2 |
| 18 | \|M7BHQ5\|M7BHQ5_CHEMY Desmocollin-1 OS=Chelonia mydas GN=UY3_06125 PE=4 SV=1 | 83285973,0 | 30 | 11 |
| 19 | \|M7B4J3\|M7B4J3_CHEMY Annexin OS=Chelonia mydas GN=UY3_12431 PE=3 SV=1 | 67334448,3 | 37 | 15 |
| 20 | \|M7BY28\|M7BY28_CHEMY Keratin, type II cytoskeletal 75 OS=Chelonia mydas GN=UY3_05865 PE=3 SV=1 | 59309912,2 | 19 | 8 |
| 21 | \|M7B7E9\|M7B7E9_CHEMY Keratin, type I cytoskeletal 12 OS=Chelonia mydas GN=UY3_09150 PE=3 SV=1 | 54607613,9 | 16 | 6 |
| 22 | \|M7BZ06\|M7BZ06_CHEMY Ovostatin OS=Chelonia mydas GN=UY3_01648 PE=4 SV=1 | 53323153,4 | 11 | 9 |
| 23 | \|M7CH40\|M7CH40_CHEMY Glutathione S-transferase OS=Chelonia mydas GN=UY3_02529 PE=4 SV=1 | 51523337,7 | 9 | 1 |
| 24 | \|M7AVN3\|M7AVN3_CHEMY Resistin OS=Chelonia mydas GN=UY3_19129 PE=4 SV=1 | 48051798,7 | 15 | 1 |
| 25 | \|M7ASY4\|M7ASY4_CHEMY NADH dehydrogenase [ubiquinone] flavoprotein 1, mitochondrial OS=Chelonia mydas GN=UY3_16906 PE=3 SV=1 | 45009251,1 | 16 | 5 |
| 26 | \|M7C1D2\|M7C1D2_CHEMY Elongation factor 1-alpha OS=Chelonia mydas GN=UY3_08660 PE=3 SV=1 | 44607890,1 | 26 | 16 |
| 27 | \|M7BB29\|M7BB29_CHEMY Actin-related protein 2/3 complex subunit 2 OS=Chelonia mydas GN=UY3_07651 PE=3 SV=1 | 43221933,8 | 31 | 12 |
| 28 | \|M7AIT5\|M7AIT5_CHEMY Uncharacterized protein OS=Chelonia mydas GN=UY3_18651 PE=4 SV=1 | 39167280,8 | 19 | 6 |
| 29 | \|M7BB95\|M7BB95_CHEMY Villin-1 OS=Chelonia mydas GN=UY3_13461 PE=4 SV=1 | 38032149,0 | 22 | 15 |
| 30 | \|M7BS42\|M7BS42_CHEMY SH3 domain-binding glutamic acid-rich-like protein OS=Chelonia mydas GN=UY3_02717 PE=4 SV=1 | 37099062,7 | 17 | 4 |
| 31 | \|M7B1U2\|M7B1U2_CHEMY Gastric intrinsic factor OS=Chelonia mydas GN=UY3_16859 PE=4 SV=1 | 35576182,8 | 18 | 7 |
| 32 | \|M7BBQ2\|M7BBQ2_CHEMY Gelsolin OS=Chelonia mydas GN=UY3_08180 PE=4 SV=1 | 34353542,0 | 11 | 6 |
| 33 | \|M7CB04\|M7CB04_CHEMY 14-3-3 protein epsilon OS=Chelonia mydas GN=UY3_05121 PE=3 SV=1 | 34132396,5 | 4 | 3 |
| 34 | \|M7BYT0\|M7BYT0_CHEMY Ig kappa chain V-III region HIC OS=Chelonia mydas GN=UY3_01734 PE=4 SV=1 | 31676565,3 | 13 | 4 |
| 35 | \|M7BVR9\|M7BVR9_CHEMY Diacylglycerol kinase beta OS=Chelonia mydas GN=UY3_00770 PE=4 SV=1 | 31142356,1 | 11 | 7 |
| 36 | \|M7C6T2\|M7C6T2_CHEMY Glutathione S-transferase 3 OS=Chelonia mydas GN=UY3_02530 PE=4 SV=1 | 31087977,0 | 16 | 8 |
| 37 | \|M7BQD0\|M7BQD0_CHEMY Condensin complex subunit 1 OS=Chelonia mydas GN=UY3_08551 PE=3 SV=1 | 30563621,5 | 18 | 10 |
| 38 | \|M7BPE5\|M7BPE5_CHEMY Keratin, type I cytoskeletal 15 OS=Chelonia mydas GN=UY3_09152 PE=3 SV=1 | 29489443,0 | 16 | 9 |
| 39 | \|M7BW96\|M7BW96_CHEMY Annexin OS=Chelonia mydas GN=UY3_01282 PE=3 SV=1 | 29282269,9 | 27 | 12 |
| 40 | \|M7AIT6\|M7AIT6_CHEMY RNA-binding protein FUS OS=Chelonia mydas GN=UY3_17972 PE=4 SV=1 | 29176970,5 | 9 | 5 |
| 41 | \|M7BG29\|M7BG29_CHEMY Glucose-6-phosphate isomerase OS=Chelonia mydas GN=UY3_05935 PE=3 SV=1 | 27690896,3 | 22 | 10 |
| 42 | \|M7CMB5\|M7CMB5_CHEMY Protein S100 OS=Chelonia mydas GN=UY3_00405 PE=3 SV=1 | 26241505,7 | 10 | 2 |
| 43 | \|M7BD06\|M7BD06_CHEMY Peptidyl-prolyl cis-trans isomerase OS=Chelonia mydas GN=UY3_12864 PE=3 SV=1 | 26208004,7 | 9 | 4 |
| 44 | \|M7BCZ3\|M7BCZ3_CHEMY Intelectin-1a OS=Chelonia mydas GN=UY3_12878 PE=4 SV=1 | 24455697,2 | 6 | 6 |
| 45 | \|M7C5W0\|M7C5W0_CHEMY Brain acid soluble protein 1 like protein OS=Chelonia mydas GN=UY3_02913 PE=4 SV=1 | 23470035,4 | 12 | 5 |
| 46 | \|M7CKD0\|M7CKD0_CHEMY 6-phosphogluconate dehydrogenase, decarboxylating (Fragment) OS=Chelonia mydas GN=UY3_01240 PE=3 SV=1 | 23310831,1 | 14 | 8 |
| 47 | \|M7B445\|M7B445_CHEMY Histone H2B OS=Chelonia mydas GN=UY3_08577 PE=3 SV=1 | 23142053,1 | 10 | 8 |
| 48 | \|M7AZ21\|M7AZ21_CHEMY Uncharacterized protein OS=Chelonia mydas GN=UY3_12869 PE=4 SV=1 | 22625163,5 | 7 | 2 |
| 49 | \|M7B6U8\|M7B6U8_CHEMY Transketolase OS=Chelonia mydas GN=UY3_15021 PE=4 SV=1 | 22397449,1 | 8 | 4 |
| 50 | \|M7ANH2\|M7ANH2_CHEMY Complement C3 OS=Chelonia mydas GN=UY3_16816 PE=4 SV=1 | 22317032,6 | 8 | 7 |
| 51 | \|M7B5M9\|M7B5M9_CHEMY 6-phosphofructo-2-kinase/fructose-2, 6-biphosphatase 2 OS=Chelonia mydas GN=UY3_12005 PE=4 SV=1 | 21547701,1 | 11 | 5 |
| 52 | \|M7BA41\|M7BA41_CHEMY Heat shock cognate 71 kDa protein OS=Chelonia mydas GN=UY3_13863 PE=3 SV=1 | 20631840,4 | 8 | 6 |
| 53 | \|M7C289\|M7C289_CHEMY Neuroblast differentiation-associated protein AHNAK OS=Chelonia mydas GN=UY3_04293 PE=4 SV=1 | 19926416,9 | 10 | 6 |
| 54 | \|M7B5Z7\|M7B5Z7_CHEMY Ovoinhibitor (Fragment) OS=Chelonia mydas GN=UY3_11953 PE=4 SV=1 | 19513614,3 | 8 | 5 |
| 55 | \|M7BZU3\|M7BZU3_CHEMY Actin, cytoplasmic 2 OS=Chelonia mydas GN=UY3_01386 PE=4 SV=1 | 18139306,5 | 4 | 1 |
| 56 | \|M7B4R1\|M7B4R1_CHEMY Creatine kinase B-type OS=Chelonia mydas GN=UY3_09975 PE=3 SV=1 | 17814200,9 | 13 | 7 |
| 57 | \|M7AN25\|M7AN25_CHEMY Prostate stem cell antigen (Fragment) OS=Chelonia mydas GN=UY3_16273 PE=4 SV=1 | 17798994,7 | 7 | 1 |
| 58 | >sp\|P80174\|SODC_CARCR Superoxide dismutase [Cu-Zn] OS=Caretta caretta PE=1 SV=2 | 17610938,3 | 13 | 6 |
| 59 | \|M7B8K7\|M7B8K7_CHEMY Tubulin beta chain (Fragment) OS=Chelonia mydas GN=UY3_18346 PE=3 SV=1 | 17479081,1 | 5 | 4 |
| 60 | \|M7BDI1\|M7BDI1_CHEMY Lactadherin OS=Chelonia mydas GN=UY3_07592 PE=4 SV=1 | 17217772,1 | 10 | 5 |
| 61 | \|M7AT00\|M7AT00_CHEMY Histone H2A type 2-C OS=Chelonia mydas GN=UY3_16895 PE=3 SV=1 | 17111343,7 | 15 | 6 |
| 62 | \|M7BGR6\|M7BGR6_CHEMY Ezrin (Fragment) OS=Chelonia mydas GN=UY3_06376 PE=4 SV=1 | 17039093,8 | 11 | 9 |
| 63 | \|M7BD58\|M7BD58_CHEMY Connective tissue growth factor OS=Chelonia mydas GN=UY3_09337 PE=4 SV=1 | 16868695,3 | 4 | 1 |
| 64 | \|M7BGI6\|M7BGI6_CHEMY Transaldolase OS=Chelonia mydas GN=UY3_08107 PE=4 SV=1 | 16714258,8 | 11 | 7 |
| 65 | \|M7B3K3\|M7B3K3_CHEMY Deleted in malignant brain tumors 1 protein OS=Chelonia mydas GN=UY3_11171 PE=4 SV=1 | 15082817,6 | 9 | 6 |
| 66 | \|M7ARL2\|M7ARL2_CHEMY Selenium-binding protein 1 OS=Chelonia mydas GN=UY3_14948 PE=4 SV=1 | 14493943,8 | 7 | 5 |
| 67 | \|M7AJ90\|M7AJ90_CHEMY L-lactate dehydrogenase OS=Chelonia mydas GN=UY3_18507 PE=3 SV=1 | 14107678,4 | 8 | 3 |
| 68 | \|M7AZ98\|M7AZ98_CHEMY Adenylyl cyclase-associated protein OS=Chelonia mydas GN=UY3_14481 PE=3 SV=1 | 14063146,1 | 9 | 7 |
| 69 | \|M7AVJ3\|M7AVJ3_CHEMY Envoplakin OS=Chelonia mydas GN=UY3_19225 PE=4 SV=1 | 12965600,7 | 6 | 6 |
| 70 | \|M7BNE3\|M7BNE3_CHEMY Mucin-16 OS=Chelonia mydas GN=UY3_09330 PE=4 SV=1 | 12522572,8 | 6 | 4 |
| 71 | \|M7BBD4\|M7BBD4_CHEMY Sulfhydryl oxidase OS=Chelonia mydas GN=UY3_08313 PE=4 SV=1 | 12264558,0 | 9 | 4 |
| 72 | \|M7BAM9\|M7BAM9_CHEMY Destrin OS=Chelonia mydas GN=UY3_10197 PE=3 SV=1 | 12063371,6 | 15 | 7 |
| 73 | \|M7C911\|M7C911_CHEMY Anaphase-promoting complex subunit 7 OS=Chelonia mydas GN=UY3_05623 PE=4 SV=1 | 11692730,9 | 5 | 3 |
| 74 | \|M7AHH1\|M7AHH1_CHEMY Ovostatin OS=Chelonia mydas GN=UY3_18518 PE=4 SV=1 | 11471855,2 | 9 | 7 |
| 75 | \|M7B6A7\|M7B6A7_CHEMY Palmitoyltransferase OS=Chelonia mydas GN=UY3_11759 PE=3 SV=1 | 11126152,1 | 4 | 4 |
| 76 | \|M7B7P1\|M7B7P1_CHEMY Purine nucleoside phosphorylase OS=Chelonia mydas GN=UY3_18654 PE=4 SV=1 | 10755597,4 | 3 | 3 |
| 77 | \|M7BLZ6\|M7BLZ6_CHEMY Nucleoside diphosphate kinase OS=Chelonia mydas GN=UY3_09820 PE=3 SV=1 | 10652200,4 | 7 | 4 |
| 78 | \|M7ASD0\|M7ASD0_CHEMY Transcobalamin-2 OS=Chelonia mydas GN=UY3_15316 PE=4 SV=1 | 10086042,7 | 8 | 6 |
| 79 | \|M7BMF4\|M7BMF4_CHEMY Mesothelin OS=Chelonia mydas GN=UY3_05875 PE=4 SV=1 | 9618998,2 | 5 | 2 |
| 80 | \|M7AZ92\|M7AZ92_CHEMY Putative chitinase 3 OS=Chelonia mydas GN=UY3_12056 PE=4 SV=1 | 9062809,2 | 13 | 7 |
| 81 | \|M7AVI1\|M7AVI1_CHEMY Histone H1.0 OS=Chelonia mydas GN=UY3_15886 PE=3 SV=1 | 8988787,0 | 6 | 3 |
| 82 | \|M7B4U2\|M7B4U2_CHEMY ATP-dependent RNA helicase DDX51 OS=Chelonia mydas GN=UY3_10709 PE=4 SV=1 | 8889698,9 | 7 | 5 |
| 83 | \|M7AX29\|M7AX29_CHEMY Alkaline phosphatase OS=Chelonia mydas GN=UY3_12821 PE=3 SV=1 | 8743380,2 | 2 | 2 |
| 84 | \|M7API4\|M7API4_CHEMY Lysozyme g OS=Chelonia mydas GN=UY3_15775 PE=3 SV=1 | 8670697,8 | 10 | 4 |
| 85 | \|M7AMU4\|M7AMU4_CHEMY Ubiquitin-like modifier-activating enzyme 1 OS=Chelonia mydas GN=UY3_16360 PE=3 SV=1 | 8644134,9 | 4 | 3 |
| 86 | \|M7B7Q5\|M7B7Q5_CHEMY Protein S100 OS=Chelonia mydas GN=UY3_14669 PE=3 SV=1 | 8475878,9 | 4 | 2 |
| 87 | \|M7AS14\|M7AS14_CHEMY Tubulin alpha-1B chain OS=Chelonia mydas GN=UY3_15478 PE=3 SV=1 | 8197398,8 | 4 | 3 |
| 88 | \|M7BSD2\|M7BSD2_CHEMY Myosin light polypeptide 6 OS=Chelonia mydas GN=UY3_11854 PE=4 SV=1 | 7920905,3 | 7 | 3 |
| 89 | \|M7BUP8\|M7BUP8_CHEMY Vitelline membrane outer layer protein 1 OS=Chelonia mydas GN=UY3_03227 PE=4 SV=1 | 7758891,2 | 6 | 3 |
| 90 | \|M7AT53\|M7AT53_CHEMY Alpha-actinin-4 OS=Chelonia mydas GN=UY3_15086 PE=4 SV=1 | 7446882,2 | 7 | 6 |
| 91 | \|M7B4U1\|M7B4U1_CHEMY Malate dehydrogenase, cytoplasmic OS=Chelonia mydas GN=UY3_09941 PE=4 SV=1 | 7429818,7 | 4 | 3 |
| 92 | \|M7BF29\|M7BF29_CHEMY Ig kappa chain C region OS=Chelonia mydas GN=UY3_16111 PE=4 SV=1 | 7283442,6 | 5 | 4 |
| 93 | \|M7BMM1\|M7BMM1_CHEMY Keratin, type II cytoskeletal 8 OS=Chelonia mydas GN=UY3_05862 PE=3 SV=1 | 7016956,1 | 8 | 5 |
| 94 | \|M7AWX2\|M7AWX2_CHEMY Sorcin OS=Chelonia mydas GN=UY3_13641 PE=4 SV=1 | 6996530,8 | 6 | 3 |
| 95 | \|M7BW30\|M7BW30_CHEMY Uncharacterized protein OS=Chelonia mydas GN=UY3_01327 PE=4 SV=1 | 6933393,6 | 2 | 1 |
| 96 | >sp\|Q10732\|HBA_CARCR Hemoglobin subunit alpha-A OS=Caretta caretta GN=HBAA PE=1 SV=1 | 6897985,0 | 3 | 3 |
| 97 | \|M7BPZ7\|M7BPZ7_CHEMY Alcohol dehydrogenase [NADP+] OS=Chelonia mydas GN=UY3_08777 PE=4 SV=1 | 6800209,6 | 3 | 3 |
| 98 | \|M7BIG6\|M7BIG6_CHEMY Copper-transporting ATPase 1 OS=Chelonia mydas GN=UY3_07408 PE=3 SV=1 | 6782074,4 | 4 | 4 |
| 99 | \|M7B7B6\|M7B7B6_CHEMY Complement factor D OS=Chelonia mydas GN=UY3_18809 PE=3 SV=1 | 6390106,0 | 4 | 3 |
| 100 | \|M7C028\|M7C028_CHEMY NMDA receptor-regulated protein 2 OS=Chelonia mydas GN=UY3_01283 PE=4 SV=1 | 6316343,1 | 4 | 2 |
| 101 | \|M7ATN3\|M7ATN3_CHEMY Protein AHNAK2 OS=Chelonia mydas GN=UY3_14827 PE=4 SV=1 | 5865992,2 | 10 | 6 |
| 102 | \|M7B6A9\|M7B6A9_CHEMY Kallikrein-11 OS=Chelonia mydas GN=UY3_15202 PE=3 SV=1 | 5697990,7 | 9 | 3 |
| 103 | \|M7AYG9\|M7AYG9_CHEMY Ig epsilon chain C region OS=Chelonia mydas GN=UY3_12302 PE=4 SV=1 | 5523912,0 | 1 | 1 |
| 104 | \|M7ANT8\|M7ANT8_CHEMY Ovostatin OS=Chelonia mydas GN=UY3_18517 PE=4 SV=1 | 5473481,9 | 5 | 4 |
| 105 | \|M7AR42\|M7AR42_CHEMY Phosphatidylethanolamine-binding protein 4 OS=Chelonia mydas GN=UY3_15123 PE=4 SV=1 | 5328793,2 | 5 | 1 |
| 106 | \|M7B436\|M7B436_CHEMY Histone H4 OS=Chelonia mydas GN=UY3_11022 PE=3 SV=1 | 5002529,5 | 3 | 1 |
| 107 | \|M7BUQ1\|M7BUQ1_CHEMY Histone H3 OS=Chelonia mydas GN=UY3_11020 PE=3 SV=1 | 5002529,5 | 3 | 1 |
| 108 | \|M7C1G7\|M7C1G7_CHEMY 14-3-3 protein theta (Fragment) OS=Chelonia mydas GN=UY3_00725 PE=3 SV=1 | 4758067,1 | 2 | 2 |
| 109 | \|M7CG64\|M7CG64_CHEMY Calmodulin OS=Chelonia mydas GN=UY3_02901 PE=4 SV=1 | 4633201,6 | 4 | 3 |
| 110 | \|M7BEA9\|M7BEA9_CHEMY Toll-like receptor 9 OS=Chelonia mydas GN=UY3_08989 PE=4 SV=1 | 4555847,8 | 3 | 1 |
| 111 | \|M7B658\|M7B658_CHEMY Ig kappa chain V-I region Walker OS=Chelonia mydas GN=UY3_10239 PE=4 SV=1 | 4527248,9 | 3 | 1 |
| 112 | \|M7B5B9\|M7B5B9_CHEMY Sulfurtransferase OS=Chelonia mydas GN=UY3_09703 PE=4 SV=1 | 4371094,6 | 4 | 3 |
| 113 | \|M7B1V3\|M7B1V3_CHEMY Histone H1.01 OS=Chelonia mydas GN=UY3_11004 PE=3 SV=1 | 4010654,3 | 6 | 3 |
| 114 | \|M7B8I3\|M7B8I3_CHEMY Epiplakin OS=Chelonia mydas GN=UY3_08593 PE=4 SV=1 | 3894956,0 | 2 | 1 |
| 115 | \|M7BEI7\|M7BEI7_CHEMY Ly6/PLAUR domain-containing protein 2 OS=Chelonia mydas GN=UY3_16274 PE=4 SV=1 | 3884710,1 | 5 | 2 |
| 116 | \|M7BYX1\|M7BYX1_CHEMY WD repeat and FYVE domain-containing protein 1 OS=Chelonia mydas GN=UY3_05515 PE=3 SV=1 | 3565186,2 | 3 | 2 |
| 117 | \|M7BZ74\|M7BZ74_CHEMY Coatomer subunit delta OS=Chelonia mydas GN=UY3_09464 PE=3 SV=1 | 3507970,1 | 2 | 1 |
| 118 | \|M7API8\|M7API8_CHEMY Leucine-rich repeat-containing protein 23 OS=Chelonia mydas GN=UY3_16433 PE=4 SV=1 | 3275660,0 | 7 | 4 |
| 119 | \|M7BNG3\|M7BNG3_CHEMY Ras-related protein Rab-3D OS=Chelonia mydas GN=UY3_05574 PE=4 SV=1 | 3266677,6 | 5 | 3 |
| 120 | >sp\|P00993\|IBP_CARCR Chelonianin OS=Caretta caretta PE=1 SV=1 | 2959240,0 | 2 | 2 |
| 121 | \|M7AS58\|M7AS58_CHEMY Protein S100 (Fragment) OS=Chelonia mydas GN=UY3_14667 PE=3 SV=1 | 2718684,8 | 3 | 1 |
| 122 | \|M7BBQ4\|M7BBQ4_CHEMY Ras-related protein Rab-10 (Fragment) OS=Chelonia mydas GN=UY3_09873 PE=4 SV=1 | 2692092,4 | 1 | 1 |
| 123 | \|M7ATL5\|M7ATL5_CHEMY 14-3-3 protein beta/alpha (Fragment) OS=Chelonia mydas GN=UY3_14181 PE=3 SV=1 | 2689636,4 | 5 | 4 |
| 124 | \|M7BM18\|M7BM18_CHEMY Pyruvate kinase OS=Chelonia mydas GN=UY3_03883 PE=3 SV=1 | 2579991,6 | 4 | 4 |
| 125 | \|M7BU13\|M7BU13_CHEMY Vitelline membrane outer layer protein 1 like protein OS=Chelonia mydas GN=UY3_03497 PE=4 SV=1 | 2206821,7 | 3 | 1 |
| 126 | \|M7CM00\|M7CM00_CHEMY Disintegrin and metalloproteinase domain-containing protein 9 OS=Chelonia mydas GN=UY3_00605 PE=4 SV=1 | 2171233,2 | 2 | 2 |
| 127 | \|M7BD95\|M7BD95_CHEMY Cell division control protein 42 like protein OS=Chelonia mydas GN=UY3_12764 PE=3 SV=1 | 2051004,1 | 2 | 1 |
| 128 | \|M7AXP6\|M7AXP6_CHEMY Calpain-2 catalytic subunit OS=Chelonia mydas GN=UY3_18483 PE=3 SV=1 | 1867621,9 | 1 | 1 |
| 129 | \|M7C2G9\|M7C2G9_CHEMY 40S ribosomal protein S14 OS=Chelonia mydas GN=UY3_00373 PE=3 SV=1 | 1728763,2 | 2 | 1 |
| 130 | \|M7BNZ4\|M7BNZ4_CHEMY Uncharacterized protein OS=Chelonia mydas GN=UY3_05406 PE=4 SV=1 | 1542436,9 | 1 | 1 |
| 131 | \|M7AS96\|M7AS96_CHEMY Steroid hormone receptor ERR1 OS=Chelonia mydas GN=UY3_17071 PE=3 SV=1 | 1528336,8 | 5 | 3 |
| 132 | \|M7ANF1\|M7ANF1_CHEMY Neuroendocrine protein 7B2 (Fragment) OS=Chelonia mydas GN=UY3_16161 PE=4 SV=1 | 1498508,3 | 1 | 1 |
| 133 | \|M7BCR4\|M7BCR4_CHEMY Putative RNA-binding protein 3 OS=Chelonia mydas GN=UY3_16887 PE=4 SV=1 | 1497391,3 | 4 | 3 |
| 134 | \|M7B3C0\|M7B3C0_CHEMY Transgelin (Fragment) OS=Chelonia mydas GN=UY3_12904 PE=3 SV=1 | 1459265,7 | 3 | 2 |
| 135 | \|M7AQD6\|M7AQD6_CHEMY Ig kappa chain V-III region SIE OS=Chelonia mydas GN=UY3_16108 PE=4 SV=1 | 1426139,5 | 1 | 1 |
| 136 | \|M7BSG2\|M7BSG2_CHEMY SH3 domain-binding glutamic acid-rich-like protein 3 OS=Chelonia mydas GN=UY3_11751 PE=4 SV=1 | 1373702,7 | 3 | 2 |
| 137 | \|M7AMF8\|M7AMF8_CHEMY Cyclic AMP-responsive element-binding protein 3-like protein 1 OS=Chelonia mydas GN=UY3_16540 PE=4 SV=1 | 1305270,5 | 1 | 1 |
| 138 | \|M7B0L3\|M7B0L3_CHEMY Uncharacterized protein OS=Chelonia mydas GN=UY3_11585 PE=3 SV=1 | 1296446,6 | 3 | 3 |
| 139 | \|M7BPC3\|M7BPC3_CHEMY Hemopexin OS=Chelonia mydas GN=UY3_12838 PE=4 SV=1 | 1178648,6 | 2 | 2 |
| 140 | \|M7B6Q8\|M7B6Q8_CHEMY Beta-1,4-galactosyltransferase 4 OS=Chelonia mydas GN=UY3_09193 PE=4 SV=1 | 1175454,0 | 1 | 1 |
| 141 | \|M7BHA4\|M7BHA4_CHEMY Mucin-1 OS=Chelonia mydas GN=UY3_06231 PE=4 SV=1 | 1096283,4 | 1 | 1 |
| 142 | \|M7AKH6\|M7AKH6_CHEMY Ly6/PLAUR domain-containing protein 3 OS=Chelonia mydas GN=UY3_18028 PE=4 SV=1 | 1064956,0 | 2 | 2 |
| 143 | \|M7C5J8\|M7C5J8_CHEMY Breast carcinoma-amplified sequence 1 OS=Chelonia mydas GN=UY3_07014 PE=4 SV=1 | 986058,5 | 1 | 1 |
| 144 | \|M7BHG2\|M7BHG2_CHEMY Glutathione peroxidase OS=Chelonia mydas GN=UY3_15242 PE=3 SV=1 | 948309,9 | 8 | 6 |
| 145 | \|M7BW25\|M7BW25_CHEMY IgW heavy chain V region W26 OS=Chelonia mydas GN=UY3_01356 PE=4 SV=1 | 915865,9 | 2 | 1 |
| 146 | \|M7AU05\|M7AU05_CHEMY Protein S100 OS=Chelonia mydas GN=UY3_14675 PE=3 SV=1 | 851080,1 | 1 | 1 |
| 147 | \|M7AU42\|M7AU42_CHEMY Ephrin-A1 OS=Chelonia mydas GN=UY3_14710 PE=3 SV=1 | 849787,4 | 2 | 2 |
| 148 | \|M7BNB0\|M7BNB0_CHEMY 60S ribosomal protein L30 OS=Chelonia mydas GN=UY3_03418 PE=4 SV=1 | 688897,2 | 2 | 2 |
| 149 | \|M7BU52\|M7BU52_CHEMY Nucleoside diphosphate kinase OS=Chelonia mydas GN=UY3_02071 PE=3 SV=1 | 613059,9 | 1 | 1 |
| 150 | \|M7BGW9\|M7BGW9_CHEMY Ras-related protein Rab-11A OS=Chelonia mydas GN=UY3_11542 PE=4 SV=1 | 515761,4 | 1 | 1 |
| 151 | \|M7AMM1\|M7AMM1_CHEMY Uncharacterized protein (Fragment) OS=Chelonia mydas GN=UY3_18985 PE=4 SV=1 | 514474,8 | 2 | 2 |
| 152 | \|M7AP69\|M7AP69_CHEMY Ig heavy chain V-II region COR OS=Chelonia mydas GN=UY3_18364 PE=4 SV=1 | 490266,8 | 1 | 1 |
| 153 | \|M7AIX8\|M7AIX8_CHEMY Isocitrate dehydrogenase [NADP] OS=Chelonia mydas GN=UY3_18539 PE=3 SV=1 | 477907,7 | 1 | 1 |
| 154 | \|M7BRX7\|M7BRX7_CHEMY Cytosolic non-specific dipeptidase OS=Chelonia mydas GN=UY3_02126 PE=4 SV=1 | 452127,5 | 1 | 1 |
| 155 | \|M7AXY9\|M7AXY9_CHEMY Dual oxidase 2 OS=Chelonia mydas GN=UY3_18286 PE=4 SV=1 | 445391,8 | 1 | 1 |
| 156 | \|M7C1F4\|M7C1F4_CHEMY Ig mu chain C region (Fragment) OS=Chelonia mydas GN=UY3_00748 PE=4 SV=1 | 443362,5 | 4 | 1 |
| 157 | \|M7CF07\|M7CF07_CHEMY Calmodulin, striated muscle OS=Chelonia mydas GN=UY3_03374 PE=4 SV=1 | 349066,8 | 1 | 1 |
| 158 | \|M7B5K9\|M7B5K9_CHEMY Tubulin alpha chain OS=Chelonia mydas GN=UY3_15477 PE=3 SV=1 | 272405,5 | 1 | 1 |
| 159 | \|M7B739\|M7B739_CHEMY Pyruvate kinase OS=Chelonia mydas GN=UY3_14905 PE=3 SV=1 | 263674,6 | 1 | 1 |
| 160 | \|M7BSY4\|M7BSY4_CHEMY Peripherin (Fragment) OS=Chelonia mydas GN=UY3_11669 PE=3 SV=1 | 242960,6 | 2 | 1 |
| 161 | \|M7AU69\|M7AU69_CHEMY Polyubiquitin-C (Fragment) OS=Chelonia mydas GN=UY3_16404 PE=4 SV=1 | 235843,0 | 2 | 2 |
| 162 | \|M7BKH2\|M7BKH2_CHEMY Dual specificity protein phosphatase 14 OS=Chelonia mydas GN=UY3_04985 PE=3 SV=1 | 215711,6 | 1 | 1 |
| 163 | \|M7BRM9\|M7BRM9_CHEMY Actin-related protein 3 (Fragment) OS=Chelonia mydas GN=UY3_08082 PE=3 SV=1 | 144098,6 | 1 | 1 |
